# Synthetic strategies towards benzothiazole-based compounds of therapeutic potency: experimental, investigational and approved drugs

**DOI:** 10.1039/d5ra03993b

**Published:** 2025-10-31

**Authors:** Reham A. Mohamed-Ezzat, Galal H. Elgemeie

**Affiliations:** a Chemistry of Natural and Microbial Products Department, Pharmaceutical and Drug Industries Research Institute, National Research Centre Dokki Cairo Egypt; b Chemistry Department, Faculty of Science, Helwan University Cairo Egypt elgemeie@yahoo.com

## Abstract

Benzothiazoles play significant roles in modern therapeutical chemistry owing to their significant effects and their integration into various pharmaceutical agents for treating critical diseases. Several approved, investigational, and experimental benzothiazole-based drugs have been accomplished. Novel strategies targeting therapeutical benzothiazoles and up-to-date synthetic strategies of anti-neurodegenerative, anti-inflammatory, antitumor, anti-microbial, and anti-viral benzothiazoles are well-recognized and reviewed. The biological investigations of the newly synthesized compounds are emphasized. This comprehensive review is considered as a valuable source in drug discovery and development.

## Abstract 2

Benzothiazoles play significant roles in modern therapeutical chemistry owing to their significant effects and their integration into various pharmaceutical agents. Novel strategies targeting therapeutic benzothiazoles have been developed*.*

## Graphical abstract

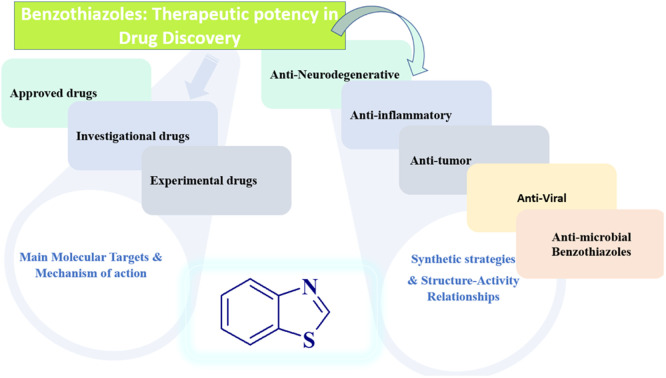

## 1. Introduction

The wide range of biological and pharmacological actions of benzothiazole-based compounds, such as their anti-cancer, anti-inflammatory, anti-viral, anti-malarial, anti-tubercular, anti-diabetic, and neuroprotective activities, have garnered a lot of interest.^1–5^ They are useful candidates in drug discovery because of their adaptable heterocyclic framework, which allows interaction with a variety of molecular targets. The therapeutic value of benzothiazole derivatives in addressing important signaling pathways and disease mechanisms has been shown in recent research. Novel benzothiazole derivatives, for example, have shown promise as GPR183 antagonists for the treatment of inflammatory bowel disease (IBD)^6^ and as strong inhibitors of phosphoinositide 3-kinase γ (PI3Kγ), which is linked to inflammatory and autoimmune diseases.^7^ Additional derivatives have the potential to treat cell death-related disorders as they preferentially inhibit RIPK1, a regulator of necroptosis.^8^

Benzothiazole scaffolds have been investigated for the synthesis of agents that target Huntington's and Alzheimer's diseases in neurodegenerative diseases.^9–15^ By blocking Hsp90, TRPC3/6, androgen receptors, glutathione peroxidase, and kinases including SCD and CLK, other benzothiazole analogs have also demonstrated effectiveness against a variety of cancer types.^16–24^

Compounds that target HSV-1, HCV, USP7, NS3/4A, and SARS-CoV-2 are used in antiviral applications; many inhibitors have been found to block Mpro and spike protein interactions.^25–29^ Other derivatives have showed anti-trypanosomal activity, with enhanced *in vivo* efficacy through optimal structural alterations, and benzothiazole-based DHFR inhibitors have shown promise in bacterial infections.^30–40^ Benzothiazoles have other applications, including molecular imaging and photodynamic treatment.^41,42^ Notably, boron-containing benzothiazoles have demonstrated promise in glioma treatment using boron neutron capture therapy (BNCT),^43^ while 68Ga-labeled benzothiazole derivatives have been developed for imaging amyloid β plaques in cerebral amyloid angiopathy (CAA).^44^ On the whole, benzothiazole derivatives are effective molecules in both therapeutic and diagnostic medicine due to their structural adaptability and pharmacological range. Clinically relevant molecules for a variety of disorders may result from their ongoing optimization and evaluation.^45–51^

## 2. Benzothiazole-based approved drugs

### 2.1. Riluzole

**Fig. 1:**
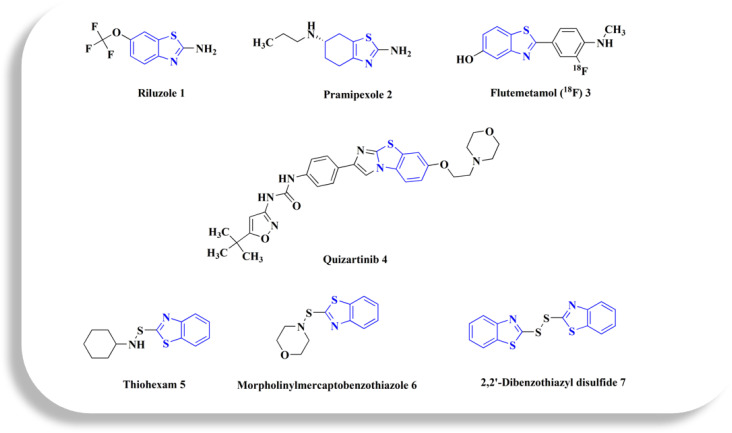
Benzothiazole-based drugs.

Riluzole 1 (Rilutek as marketed by Sanofi) (Fig. 1) is a glutamate antagonist utilized as anti-convulsants and used to treat to amyotrophic lateral sclerosis.^52^ Riluzole is an activator of the TREK-1 channel and has been used clinically for a long time to treat almost all individuals with amyotrophic lateral sclerosis. It has a strong analgesic impact in a number of models of inflammatory and neuropathic pain. It's interesting to note that riluzole inhibits proliferation as well. The riluzole's effects on bone pain that induced by prostate cancer were analyzed. Prostate cancer (PCa) cell viability has also been shown to be dramatically reduced *in vitro* by riluzole treatment. In addition, riluzole's antiproliferative action causes cancer cells to express more TREK-1 channels.^53^

**Scheme 1:**
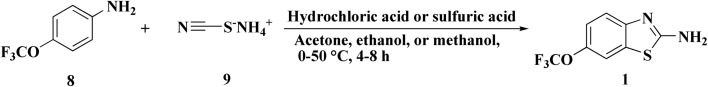
Synthesis of riluzole 1.

Riluzole (1) was prepared through reacting 4-trifluoromethoxyaniline (8) with ammonium thiocyanate (9) in acidic condition (Scheme 1).^54^

### 2.2. Pramipexole

Pramipexole 2 (Fig. 1) is an anti-parkinsonian drug used in treating parkinsonism and restless leg syndrome.^55^

**Scheme 2:**
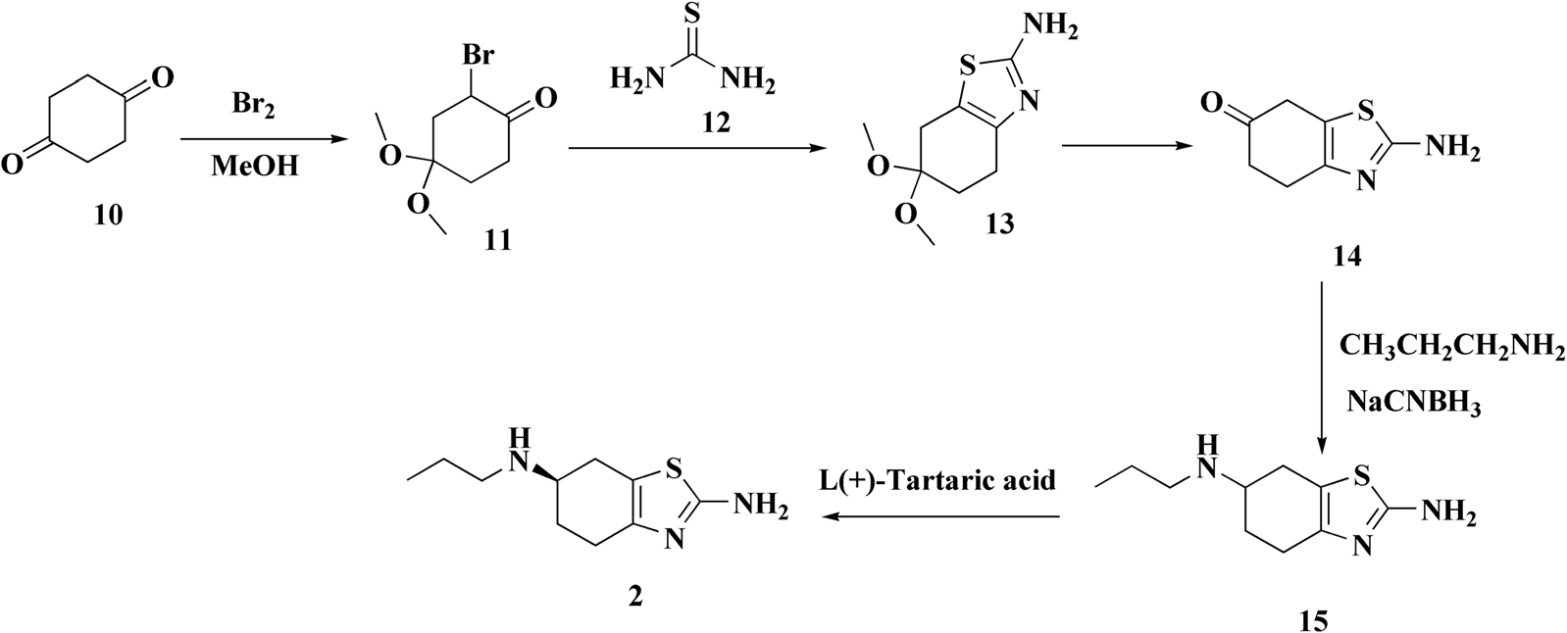
Synthesis of pramipexole 2.

The preparation of the pramipexole dihydrochloride proceeds from the 4-acetamidocyclohexanone and starts *via* bromination then thiourea cycliation and eventually transformation to generate the pramipexole (Scheme 2). An enhanced process uses aminocyclohexanol as the starting material and incorporates safer chemicals and fewer steps to increase efficiency and lessen environmental impact.^56^

### 2.3. Flutemetamol (^18^F)

Flutemetamol (^18^F) 3 (Fig. 1) is a PET scanning radiopharmaceutical containing the radionuclide fluorine-18. Estimating the density of β amyloid neuritic plaque in the brain by Positron Emission Tomography (PET) imaging is recommended for adult patients experiencing cognitive impairment who are being assessed for Alzheimer's disease (AD) or other potential causes of cognitive loss.^57^ As a radiotracer, the ^18^F-flumetamol has a strong affinity for brain amyloid plaques, is capable of differentiating AD cases from controls, and has strong histopathological neuritic amyloid plaque density concordance.^58^

**Fig. 2:**
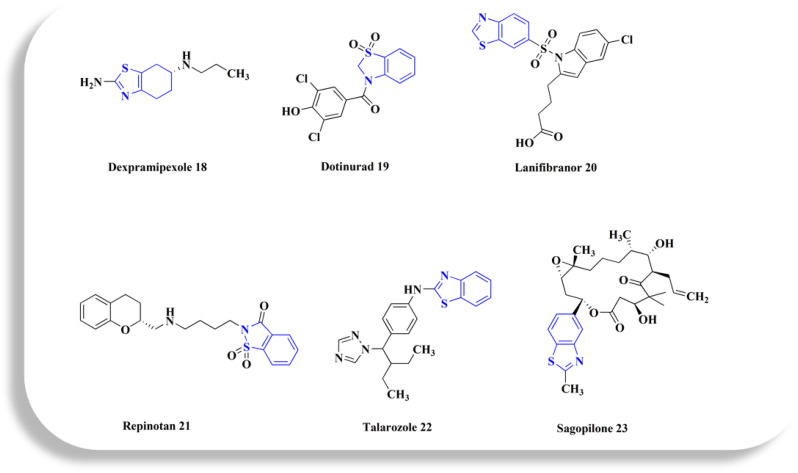
Benzothiazole-based investigational drugs.

**Fig. 3:**
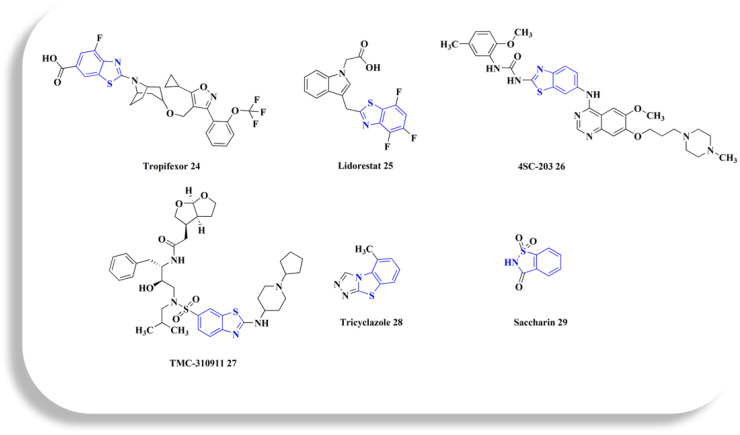
Benzothiazole-based investigational drugs.

**Scheme 3:**
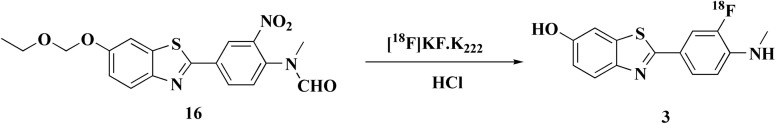
Synthesis of flutemetamol (^18^F) 3.

Flutemetamol (^18^F) is produced by a nucleophilic aromatic substitution reaction between [^18^F]fluoride and a nitro precursor. The synthesis pathway is described in detail below (Scheme 3).^59^

### 2.4. Quizartinib

Quizartinib 4 (Fig. 1) is an oral and active fms-like tyrosine kinase 3 inhibitor (FLT3 inhibitor) and it is the 1^st^ drug developed precisely targeting FLT3. Quizartinib is a highly potent, 2^nd^-generation, selective, type 2 FLT3 inhibitor. In patients with newly diagnosed AML that was FLT3-ITD-positive, phase-1/2 trials revealed that quizartinib plus chemotherapy demonstrated anti-leukaemic effectiveness with a tolerable safety profile. The drug was also effective when used separately following allo-HCT31. Additionally, in the phase 3 QuANTUM-R trial, quizartinib monotherapy increased overall survival compared to salvage chemotherapy in the relapsed or refractory circumstances.^60^

### 2.5. Thiohexam

Thiohexam 5, 2-(cyclohexylaminothio)benzothiazole (Fig. 1), is considered as a rubber cure accelerator. It is an identified dermatological sensitizer and allergen as well.^52^

### 2.6. Morpholinylmercaptobenzothiazole

The 2-(morpholin-4-ylsulfanyl)-1,3-benzothiazole 6 (Fig. 1)^52^ an be synthesized *via* the oxidative condensation of the 2-mercaptobenzothiazole and morpholine. This drug also known as the 2-(morpholinothio)-benothiazole. Sodium hypochlorite (NaOCl) is frequently used as the oxidizing agent in this reaction.

2-(Morpholinothio)-benothiazole is a sealant has demonstrated efficacy against cancerous cells *in vitro*. By interfering with the formation of disulfide bonds, 2-(morpholinothio)-benothiazole interacts with the cell nuclei and stops DNA synthesis. According to *in vivo* research, 2-(morpholinothio)-benzothiazole can be utilized as a sealant for brain injuries because it is not absorbed into the bloodstream. Additionally, it might have antiangiogenic properties, which could explain why it inhibits EGF.^61^

### 2.7. 2,2′-Dibenzothiazyl disulfide

2,2′-Dibenzothiazyl disulfide 7 (Fig. 1) is an accelerator utilized in processing plastic regeneration and the process of synthetic rubber & natural rubber. It is also a dermatological sensitizer and allergen.^52^

**Scheme 4:**
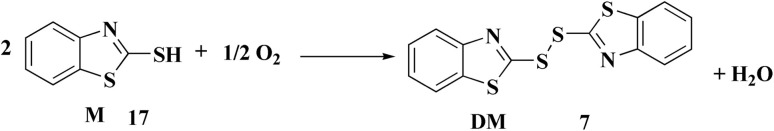
Synthesis of 2,2′-dibenzothiazyl disulfide 7.

The process for synthesizing 2,2′-dithiodibenzothiazole is accomplished *via* catalyzing the oxidation of the molecular oxygen (Scheme 4). A transition metal salt and molecule with N or O acting as a ligand make up the procedure. In order to synthesize 2,2′-dithiobis(benzothiazole), the complex is utilized as a catalyst to react benzothiazole-2-thiol with a solvent for one to thirty hours in an oxygen or air atmosphere at a pressure. The catalyst utilized in this invention is free of precious metals, has multiple uses, produces less waste, is eco-friendly, and has promising industrial applications. Its synthesis process is straightforward, and its catalytic activity and reaction efficiency are high. The synthesis reaction product has a high degree of selectivity and minimal by-products, and no base, acid, or other additives are required.^62^

Another method for preparing dibenzothiazyl disulfide involves using hydrogen peroxide to oxidize mercaptobenzothiazole in an aqueous suspension. The oxidation is carried out with the use of ethylenediaminetetraacetic acid (EDTA) or a derived alkali metal salt.^63^

## 3. Benzothiazole-based investigational drugs

### 3.1. Dexpramipexole

Dexpramipexole 18, (Fig. 2) (6*R*)-*N*6-propyl-4,5,6,7-tetrahydro-1,3-benzothiazole-2,6-diamine, is under clinical investigation (trial no. NCT01511029).

**Scheme 5:**
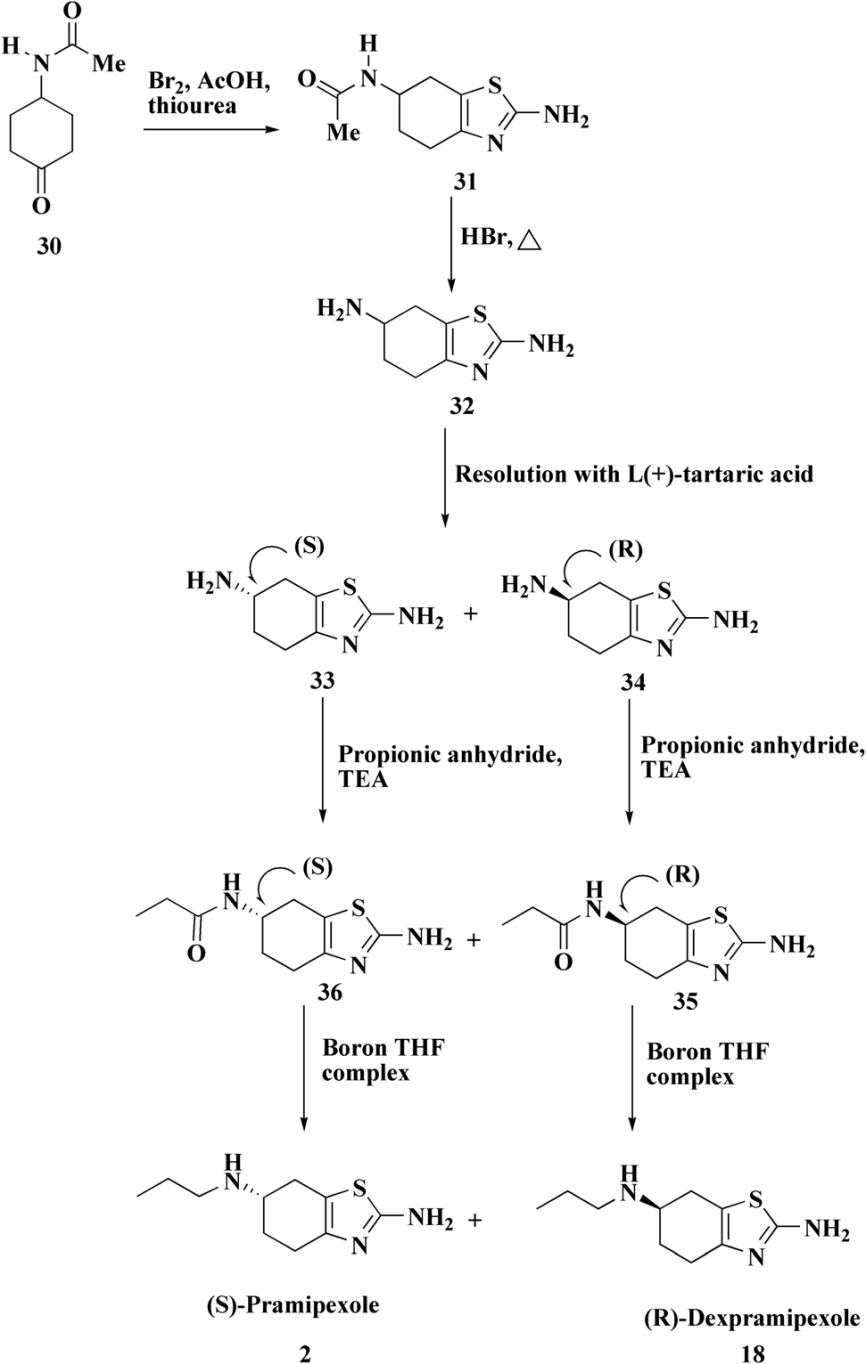
Synthesis of dexpramipexole 18.

Dexpramipexole, an orally bio-available aminobenzothiazole that was originally developed for treating amyotrophic lateral sclerosis, verified an important dose dependent eosinophil lowering effect.^64^ Synthesis of dexpramipexole 18 is accomplished as depicted in Scheme 5.^65^

### 3.2. Dotinurad

Dotinurad 19 (Fig. 2) is under clinical trial NCT03372200. It is considered as a selective urate reabsorption inhibitor that specifically inhibits URAT1. Recently developed as a potent uricosuric drug.^66,67^ It is widely utilized in clinical practice in Japan to treat hyperuricemia in Japan.^68–70^

**Scheme 6:**
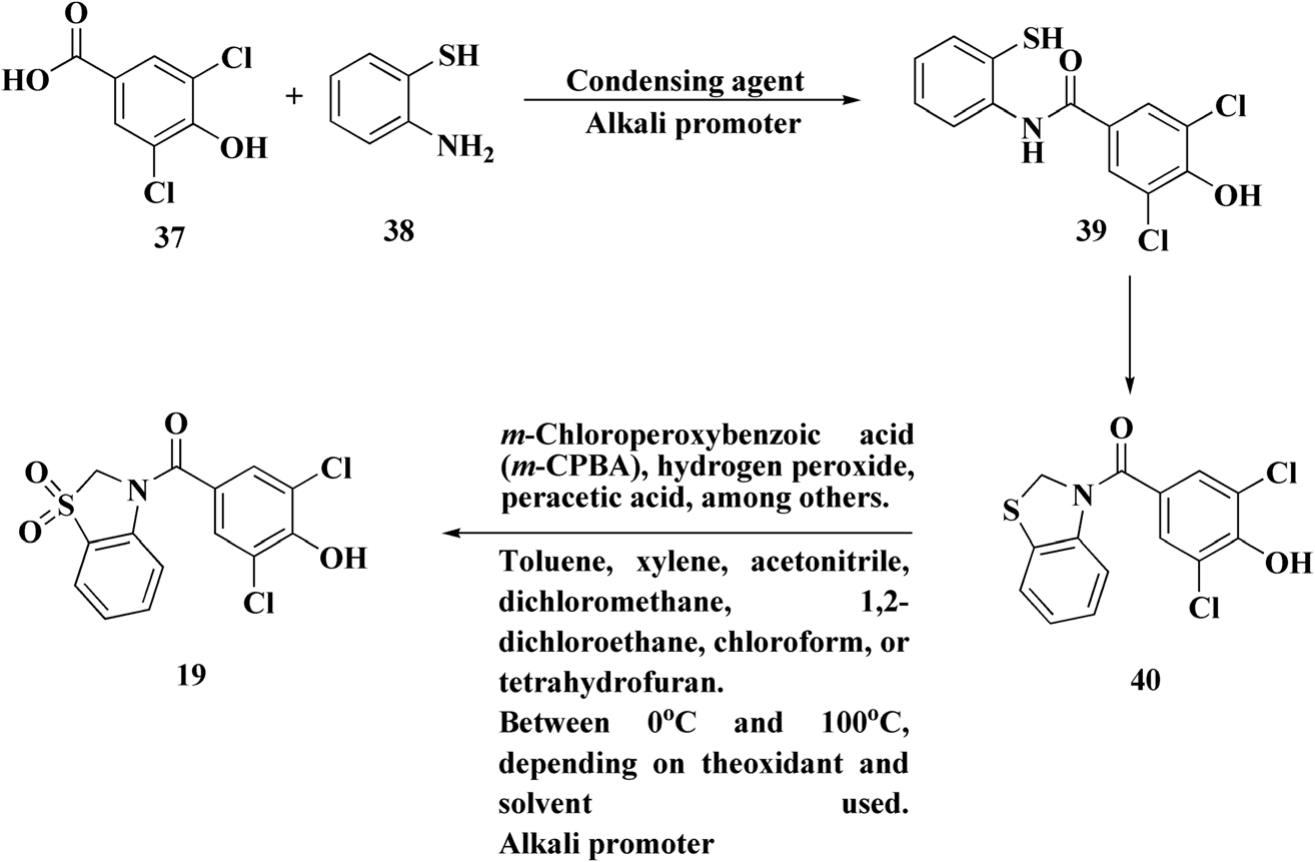
Synthesis of dotinurad 19.

The invention of dotinurad preparation reveals the technique that starts with 2-aminobenzenethiol and proceeds to produce a target product dortinode *via* a series of reactions, including condensation, cyclization, oxidation, and similar processes (Scheme 6). A benzoxazole intermediate is synthesized by cyclizing a 2-aminophenol derivative with an acid chloride or a benzoic acid derivative. Introducing substituents necessary for dotinurad's action by functionalizing the benzoxazole ring by halogenation or alkylation. The whole dotinurad molecule is furnished by coupling the functionalized benzoxazole intermediate with other elements.

Easy access to raw materials, speed, ease of use, cost effectiveness, environmental preservation, and adaptability for large scale industrial production are all advantages of the preparation process.^71^

### 3.3. Lanifibranor

Lanifibranor (Fig. 2), 20 4-[1-(1,3-benzothiazole-6-sulfonyl)-5-chloro-1*H*-indol-2-yl]butanoic acid, is under clinical trial's investigations under number NCT03008070.^52^ Inflammatory, metabolic, and hepatic fibrotic pathways are modulated by the panperoxisome proliferator activated receptor (PPAR) agonist lanifibranor in the pathogenesis of non-alcoholic steatohepatitis (NASH).^72,73^

### 3.4. Repinotan

Repinotan 21 (Fig. 2) is a selective, high affinity, full agonist of the 5HT1A receptor sub-type with neuroprotective features.^52^

Repinotan, also known as BA X3702, is a highly effective agonist for the 5-hydroxytryptamine1A (5HT1A) receptor sub-type, which is extensively expressed in cortical tissue. In a rat permanent middle cerebral artery occlusion model, repinotan has been demonstrated to lower extracellular glutamate by approximately 50% when compared to untreated animals. Repinotan has currently been studied in a number of clinical investigations for acute ischemic stroke due to the positive experimental results.^74^

**Scheme 7:**
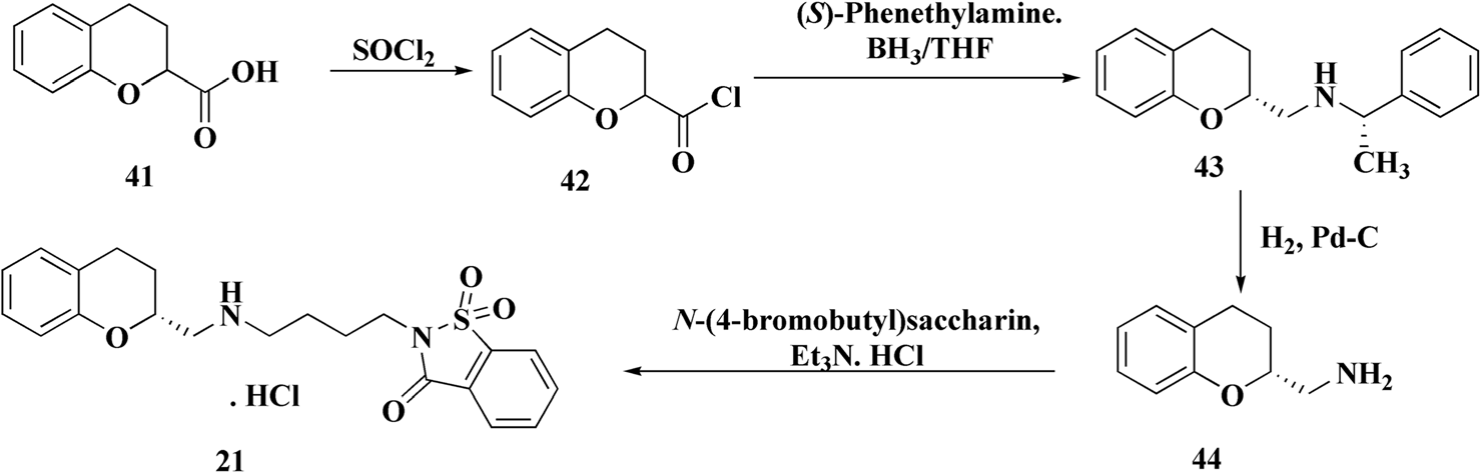
Synthesis of repinotan 21.

Synthesis of repinotan (21) is depicted, as shown in Scheme 7, chroman-2-carboxylic acid (42) is treated with thionyl chloride to create acid chloride (34). When 34 reacts with (*S*)-phenethylamine, a 1 : 1 mixture of diastereomers is produced. The mixture is separated (fractional crystallized) to provide the required epimer with high optical purity. The amide is then reduced with diborane to yield benzyl amine (43), which is then hydrogenolyzed to provide (*R*)-2-(aminomethyl)chroman (44). Repinotan (21) is obtained by alkylating 44 with *N*-(4-bromobutyl)saccharin and is separated as the hydrochloride salt.^75^

### 3.5. Talarozole

Talarozole 22 (Fig. 2) has been clinically investigated for treating cutaneous inflammation and psoriasis.^52^ Under a license from Johnson & Johnson, it was developed *via* Barrier Therapeutics Inc., as a selective and potent inhibitor of cytochrome P450 26-mediated breakdown of endogenous all *trans*-retinoic acid for treating acne and psoriasis.^76^

### 3.6. Sagopilone

Sagopilone 23 (ZK219477, Fig. 2) is an epothilone analogue^77^ (16 member ring macrolides) that works against tumor cell lines resistant to many drugs. It has shown therapeutically effective in treating a number of solid tumors, including melanoma and ovarian cancer.^78^ Also, it has been used in trials for treating of CNS disease, neoplasms, and breast cancer.

### 3.7. Tropifexor

Tropifexor 24 (Fig. 3), 3-[2-(trifluoromethoxy)phenyl]-1,2-oxazol-4-yl}methoxy)-8-azabicyclo[3.2.1]octan-8-yl]-4-fluoro-1,3-benzothiazole, is under clinical investigation (trial no. NCT02516605).

Tropifexor, LJN452, is a nonbile acid FXR agonist with subnanomolar activity due to a special bicyclic nortropine-substituted benzothiazole carboxylic acid moiety that has been optimized for improved fit into the ligand-binding domain of FXR.^79^ In rodent models of non-alcoholic steatohepatitis (NASH)^79,80^ and cholestasis,^81^ tropifexor shown greater effectiveness than OCA and effectively regulated FXR-target genes in the intestine and liver. Tropifexor exhibits a pharmacokinetic profile in humans^82^ and has demonstrated active FXR target engagement through transient & dose dependent rises in fibroblast growth factor 19 in healthy volunteers^82^ and patients with NASH^83,84^ and primary bile acid diarrhea.^85,86^

**Scheme 8:**
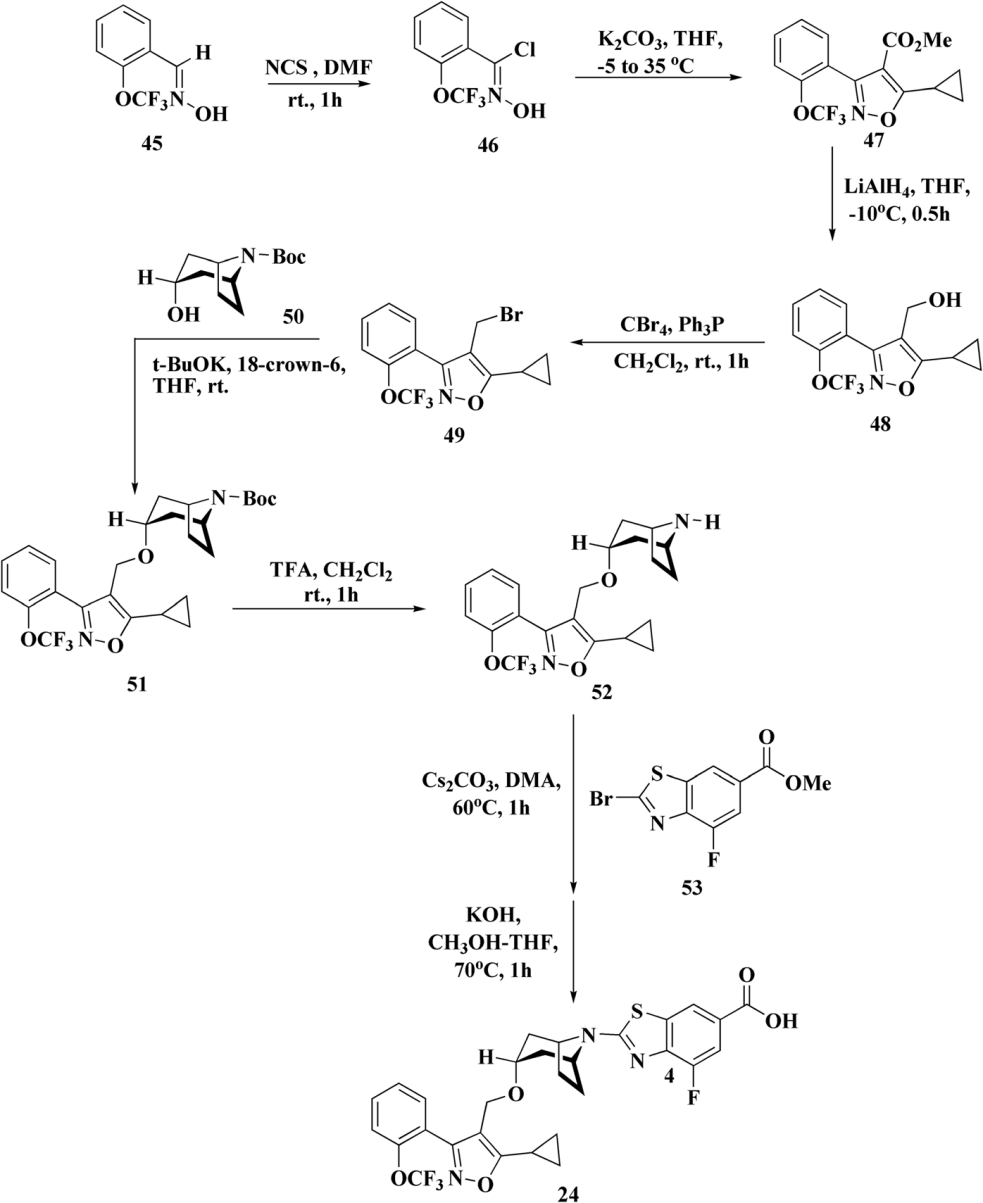
Synthesis of tropifexor 24.

One important modulator of bile acid synthesis, conjugation, and transport is the farnesoid X receptor (FXR). An FXR antagonist called tropifexor (LJN452) is presently undergoing phase 2 trials to treat nonalcoholic steatohepatitis and cholestatic liver disorders. The reaction of the nitrile oxide obtained from the removal of HCl from 46 with *β*-keto ester to produce isoxazole 47 in a 52% yield is a crucial step in the synthesis shown in Scheme 8.^87,88^

### 3.8. Lidorestat

Investigations to identify therapy for the compliations of chronic diabetes lead to the discovery of a new series of highly effective and selective 3-[(benzothiazol-2-yl)methyl]indole-*N*-alkanoic acid aldose reductase inhibitors. 3-[(4,5,7-Trifluorobenzothiazol-2-yl)methyl]indole-*N*-acetic acid (lidorestat 25, Fig. 3) as a lead compound inhibits aldose reductase (IC_50_ of 5 nM), while being 5400 fold less potent against aldehyde reductase which is an associated enzyme elaborated in the detoxification of reactive aldehydes. In the five-day STZ induced diabetic rat model, it reduces nerve & lens sorbitol levels (ED50s of 1.9 & 4.5 mg per kg per d po).^89,90^

**Scheme 9:**
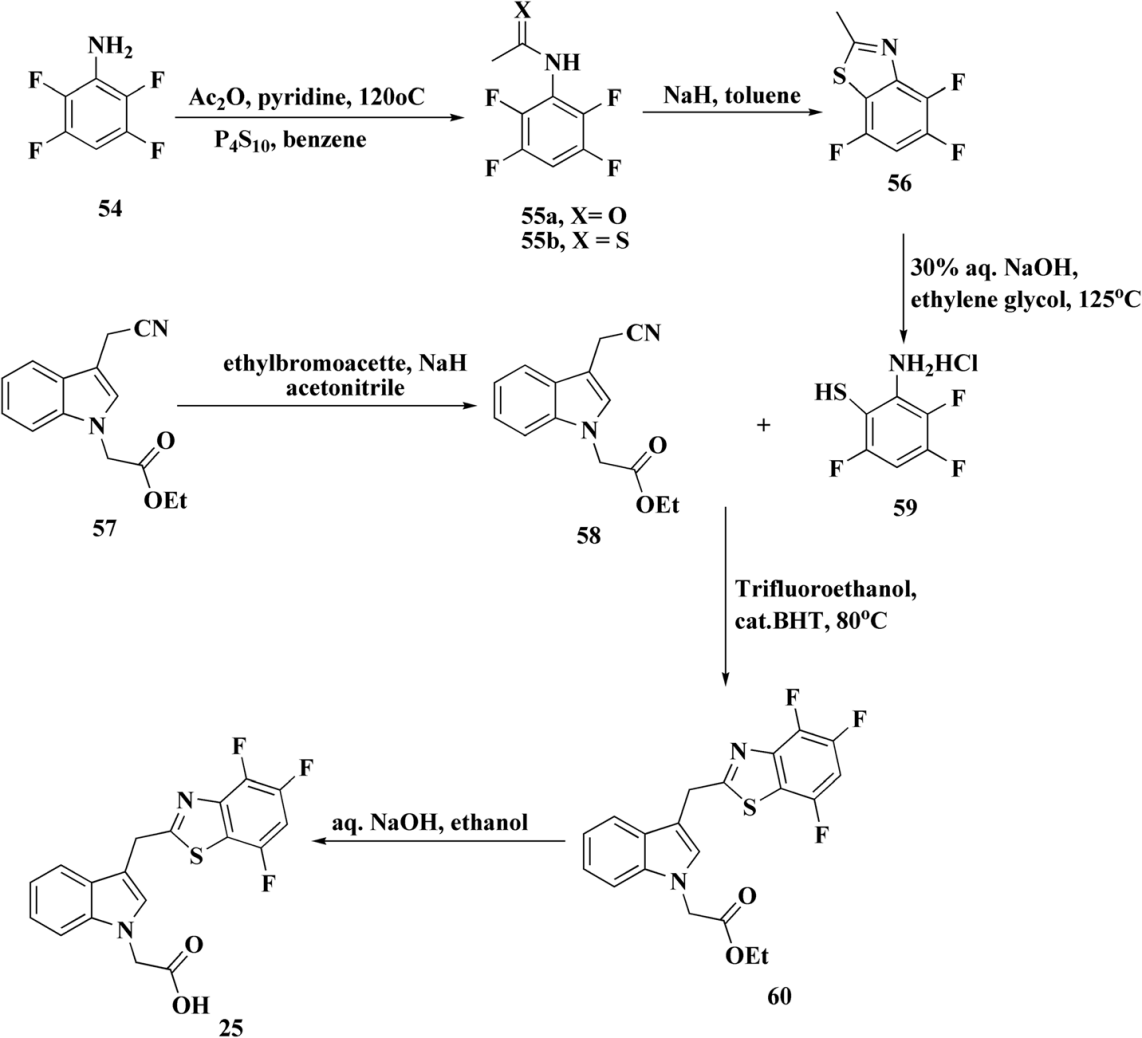
Synthesis of lidorestat 25.

The synthesis of lidorestat comprises the construction to the benothiazole scaffold, then linking it with indole core are accomplished in Scheme 9.^91^

### 3.9. 4SC-203

4SC-203 26 (Fig. 3) has been utilized in investigational trials for treating acute myeloid leukemia.

4SC-203 is a new small molecule selective spectrum kinase inhibitor of the benzothiazole chemical class that exhibits a unique selectivity profile against ALK, AXL, VEGF-R2, FAK, FLT3, FLT3 mutants, & TRK receptors in both *in vitro* estimations with an inhibitory potency on cell line growth in the low nM range. Furthermore, 4SC-203 has demonstrated a strong antitumor impact in acute myeloid leukemia (AML) related *in vivo* models in preclinical investigations. 4SC-203 demonstrated favorable pharmacokinetic characteristics and was well tolerated over the whole concentration range tested in a first-in-man study with healthy volunteers.^92^

### 3.10. TMC-310911

TMC-310911 27 (ASC-09; furan-3-yl *N*-[(2*S*,3*R*)-3-hydroxy-4-[*N*-(2-methylpropyl)2-[(1-cyclopentylpiperidin-4-yl)amino]-1,3-benzothiazole, Fig. 3) is an investigational protease inhibitor. Its potential application to HIV-1 infections is being researched. Significant efficacy against many HIV-1 strains, comprising multi PI-resistant strains, has been demonstrated by TMC-310911.^52^ TMC310911 is a new protease inhibitor (PI) for HIV-1 that shares structural similarities with darunavir (DRV) but has enhanced virological properties.^93^

### 3.11. Tricyclazole

The 5-methyl-1,2,4-triazolo(3,4-*b*)benzothiazole, tricyclazole 28 (Fig. 3) is synthesized from 2-hyrazino 4-methyl benzothiazole.

**Scheme 10:**
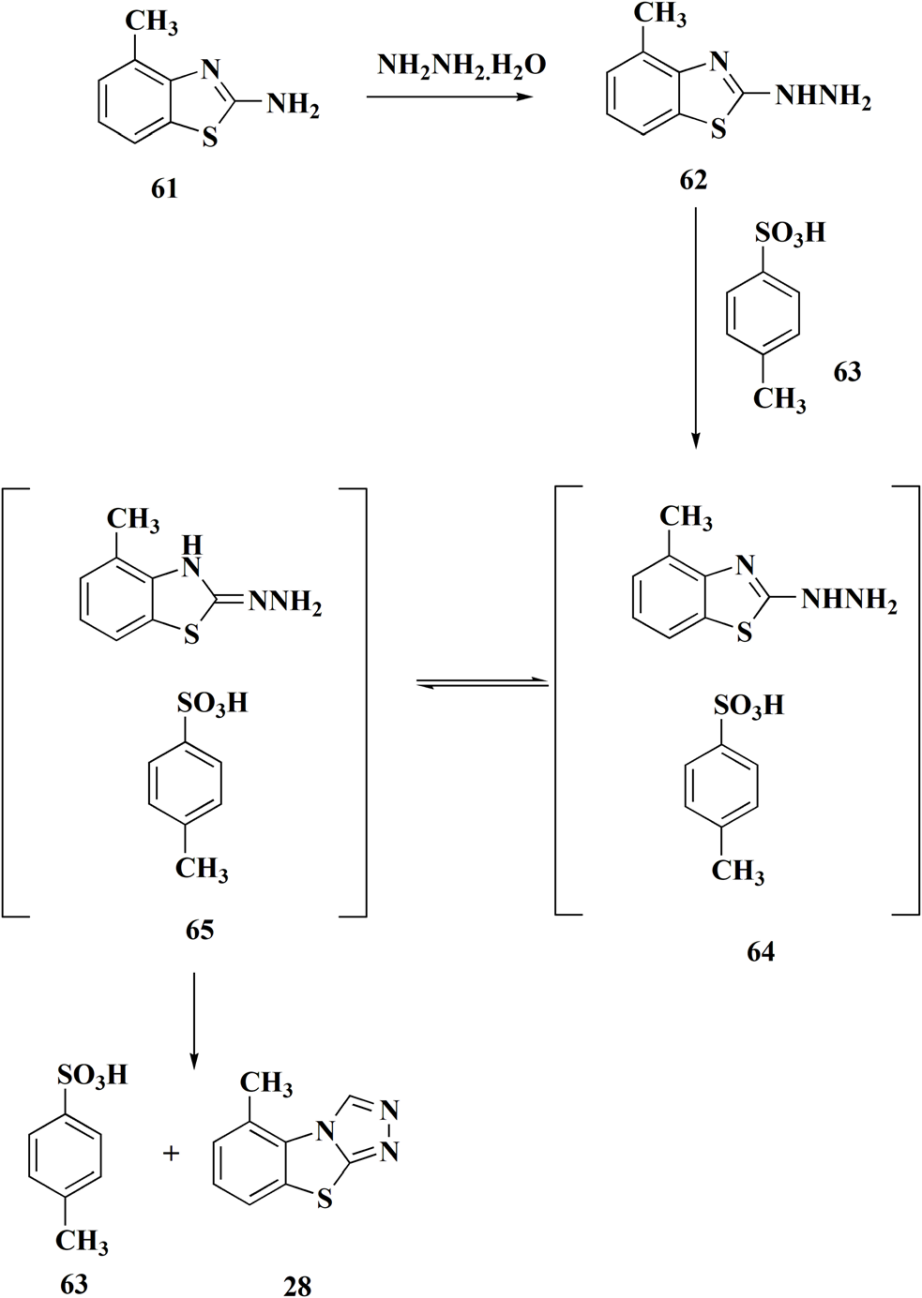
Synthesis of tricyclazole 28.

2-Hydrazine 4-methyl benzothiazole is prepared by reacting amino compound (61) with hydrazine hydrate in the presence of MEG and hydrochloric acid (Scheme 10). The condensation process with formic acid is carried out in the presence of acid promotor catalyst. The final product is isolated in aqueous slurry form by dumping solvent-free molton stirrable mass in precooled water. This is an improved, efficient, and environmentally safe method for producing tricyclazole useful fungicide for rice blast in aqueous slurry form.^94^

### 3.12. Saccharin

Saccharin 29 (Fig. 3), 2,3-dihydro-1lambda6,2-benzothiazole-1,1,3-trione, has been investigated for treating hyperglycemia and hypertension.^95^

**Scheme 11:**
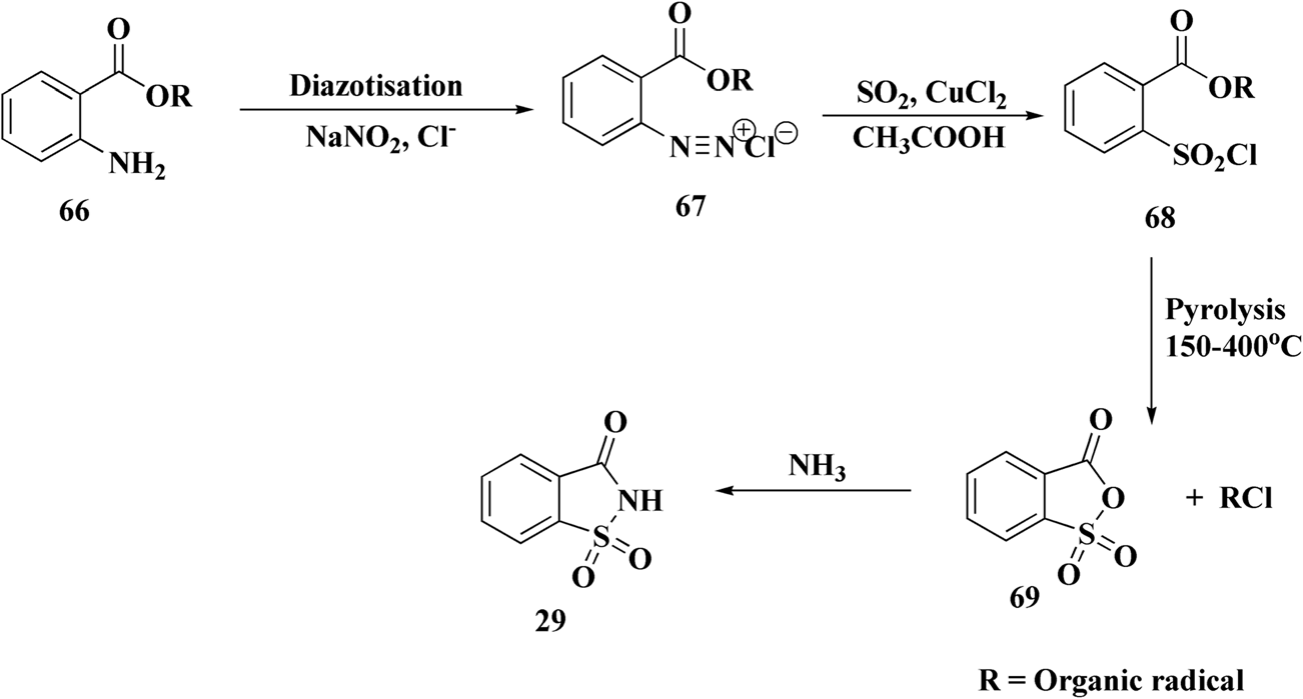
Synthesis of saccharin 29.

A process for the preparation of saccharin by reacting an aqueous hydrochloric acid solution of *o*-methoxycarbonylbenzenediazonium chloride with sulfur dioxide. Oxidation then reaction with ammonia afforded saccharin (Scheme 11).^96^

## 4. Benzothiazole-based experimental drugs

**Fig. 4:**
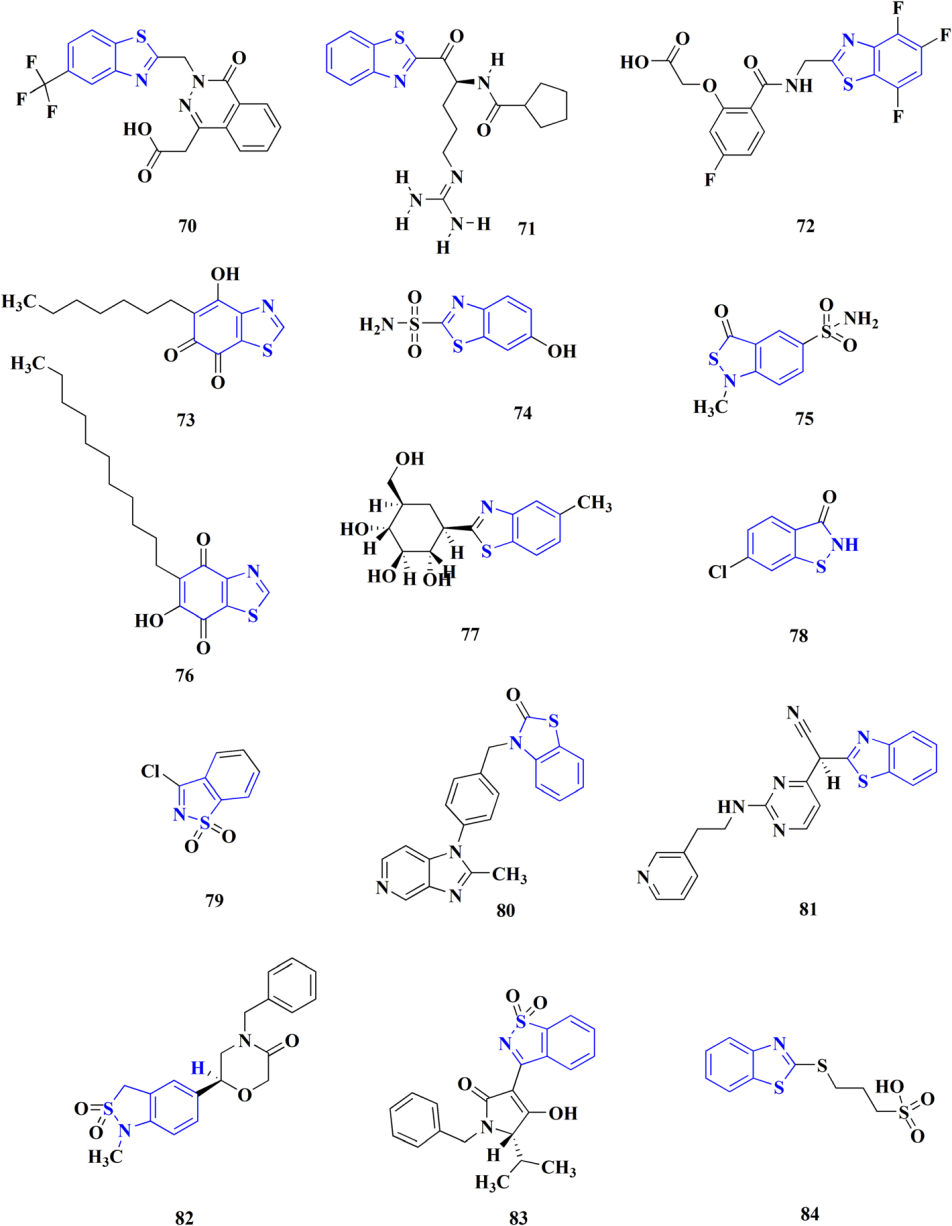
Benzothiazole-based experimental drugs.

**Fig. 5:**
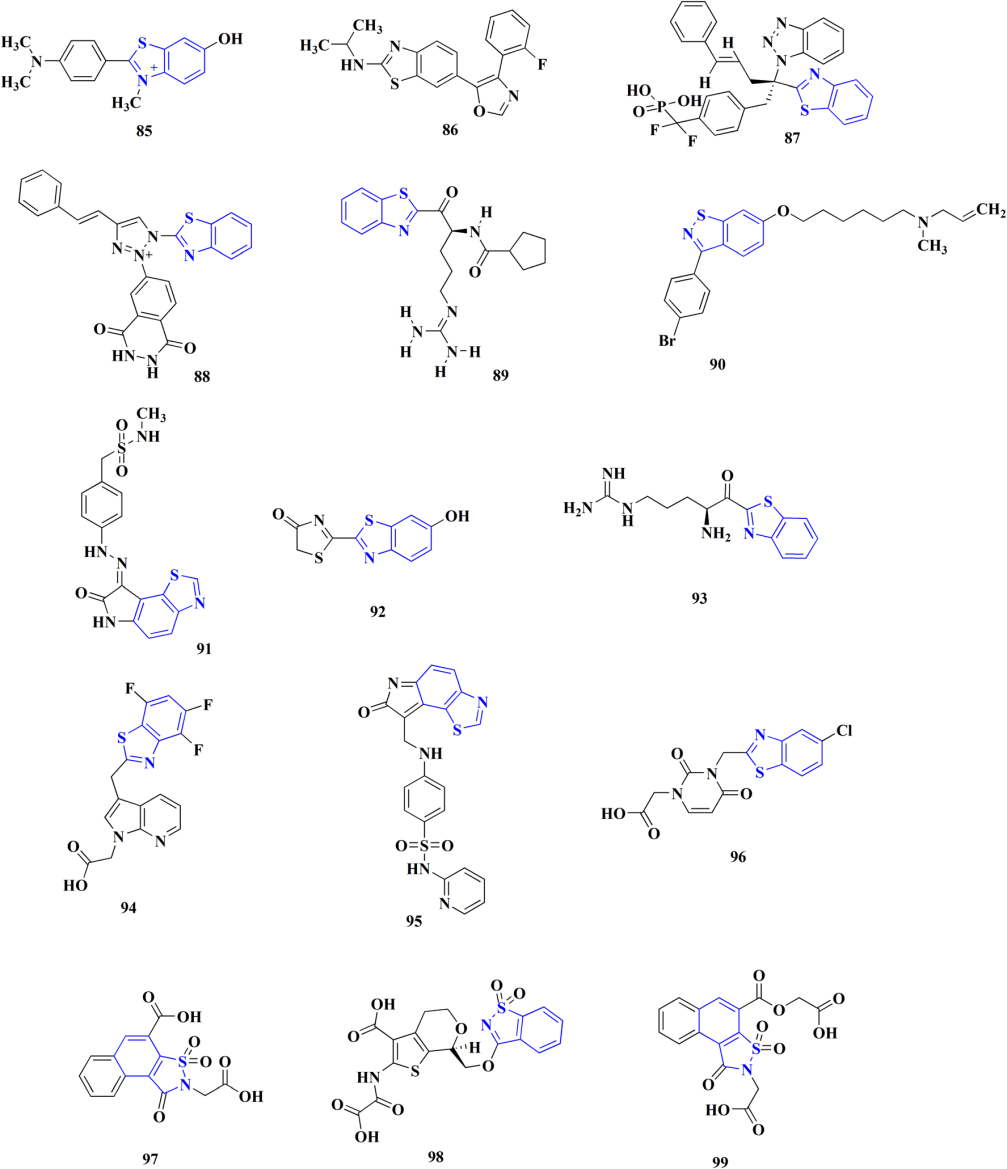
Benzothiazole-based experimental drugs.

Many structures containing benzothiazoles were designed and synthesized, they revealed remarkble biological activities and are under pre-clinical and clinical investigations such as Zopolrestat 70 which acts as an active competitive GLO1 inhibitor which is currently in phase-III clinical trials.^97^ Many other experimental drugs were developed which are depicted in Fig. 4 and 5 such as RWJ-56423,^98^ IDD552^99^ 6-hydroxy-5-undecyl-4,7-benzothiazoledione, 5-heptyl-6-hydroxy-1,3-benzothiazole-4,7-dione, 2-(beta-d-glucopyranosyl)-5-methyl-1,3,4-benzothiazole, pseudosaccharin chloride, 1-methyl-3-oxo-1,3-dihydro-benzo[C]isothiazole-5-sulfonic acid amide, ticlatone, CP-271485, CP-94707, (2*S*)-1,3-benzothiazol-2-yl{2-[(2-pyridin-3-ylethyl)amino]pyrimidin-4-yl}ethanenitrile, 3-(2-benzothiazolylthio)-1-propanesulfonic acid, RWJ-51084.^52^

## 5. Main molecular targets and mechanism of benzothiazole-based drugs (investigational & approved drugs)

**Table:** 

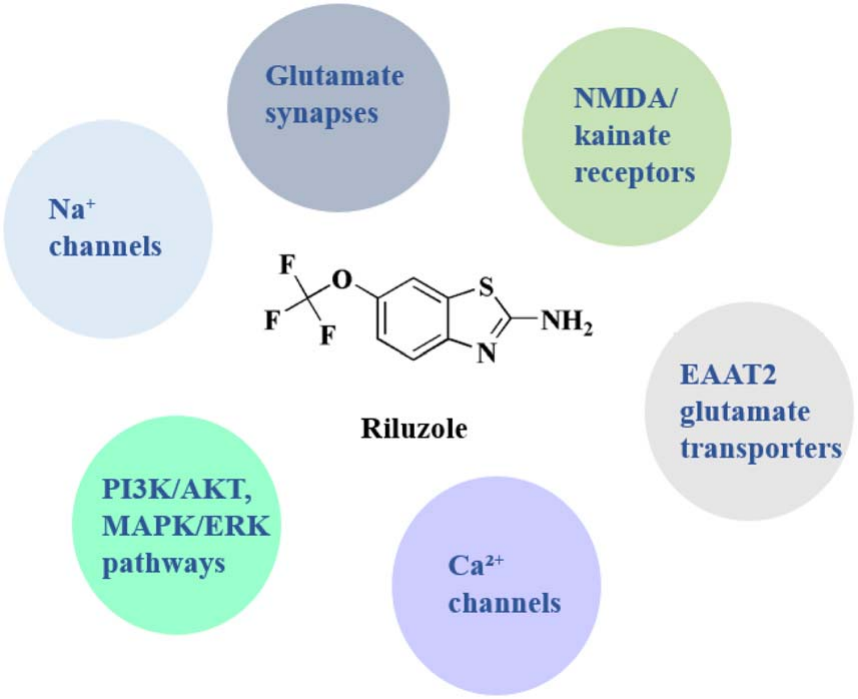	**Riluzole** • Riluzole targeting presynaptic voltage-gated sodium channels, it inhibits Nav channels, which lowers neuronal excitability. Its effect is to limit excitotoxicity and decrease glutamate release^100,101^
	• It targeted glutamate synapses, it reduces synaptic glutamate release through Na^+^ channel blocking; it indirectly targeting the glutamate synapses; through preventing glutamate-induced excitotoxicity, a major contributing factor to ALS, in neurons^102^
	• It modulates NMDA & kainate receptors targeting postsynaptic NMDA and kainate-type glutamate receptors; *via* non-competitive antagonism; riluzole diminishes excitatory toxicity and calcium influx^103^
	• It enhances the glutamate clearance through targeting the glial glutamate transporters (*e.g.*, EAAT2/GLT-1). It acts indirectly to upregulate the transporter expression, promoting the glutamate reuptake, to reduce the extracellular excitotoxin buildup^104^
	• It modulate calcium channels *via* targeting the high voltage-activated calcium channels, it inhibits calcium influx during the depolarization, reducing the neurotransmitter exocytosis^105^
	• Concerneing the neurotrophic/survival pathways (PI3K/AKT, MAPK/ERK), riluzole targeting the intracellular pro-survival signaling indirectly through stimulating pathways supporting the neuronal viability & plasticity^106^
	
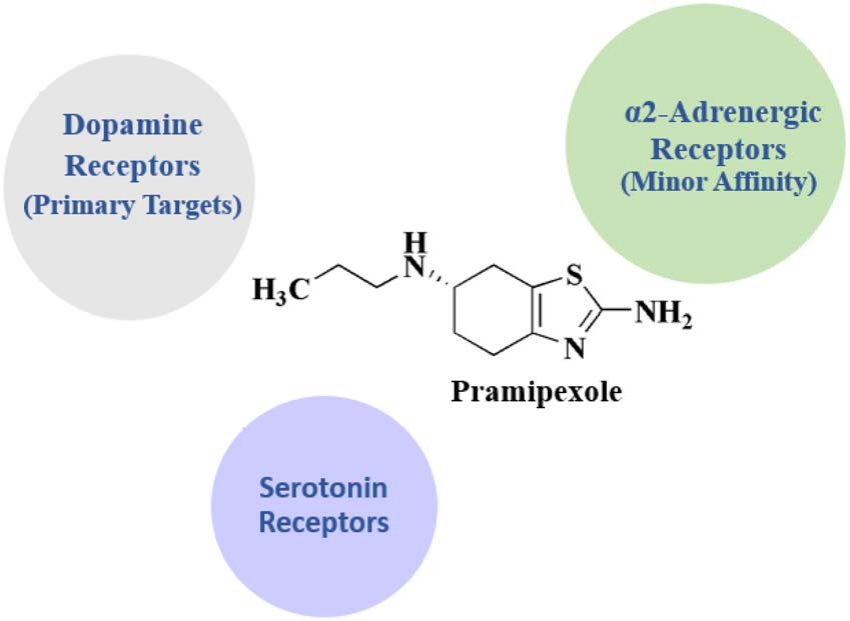	**Pramipexole** • Pramipexole binds with high affinity to the dopamine *D*_3_ receptor (DRD3), making it its primary receptor target
	• It also considered as an agonist at *D*_2_ receptors (DRD2), with some extent lower affinity
	• Pramipexole displays moderate affinity for *D*_4_ receptor (DRD4)
	• Pramipexole indicated very low to negligible binding to serotonin (5-HT_1_A/1B/1D) & α_2_-adrenergic receptors
	• The main mechanism that pramipexole reduces motor symptoms in Parkinson's disease and restless legs syndrome (RLS) is by activating *D*_2_-like dopamine receptors, particularly *D*_3_ and *D*_2_. Its *D*_3_ predilection might also have neuroprotective and mood-regulating benefits, although *D*_4_ and other minor affinities have little therapeutic value^107^
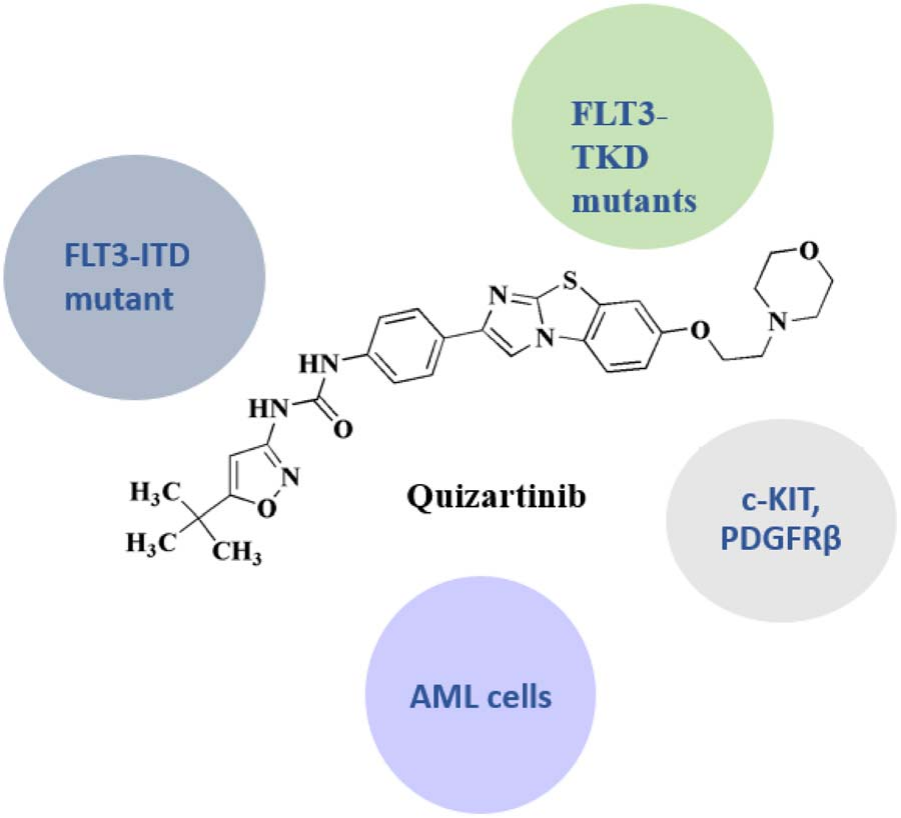	**Quizartinib** • FLT3 (FMS-like tyrosine kinase 3) is the primary target of Quizartinib. Its mechanism is as follows: It binds to the inactive conformation of FLT3. It inhibits autophosphorylation and downstream signaling. It blocks pathways, and causes FLT3-ITD-positive AML cells to undergo apoptosis^108^
	• FLT3 mutations associated with resistance
	Mechanisms of resistance: mutations in FLT3-TKD, such as D835Y and F691L, decrease binding affinity. Alternative signaling pathways are upregulated. The effectiveness of monotherapy is limited by resistance; combination therapy is being researched^109^
	• Low-level off-target kinase inhibition: Limited inhibition of other kinases such as c-KIT, PDGFRβ, RET. It may have mild off-target effects and contribute to myelosuppression^110^
	• Clinically used in treating acute myeloid leukemia (AML) with FLT3-ITD mutation; FDA clearance (2023): To treat newly diagnosed FLT3-ITD AML in conjunction with chemotherapy (brand name Vanflyta®). It is orally administrated^111,112^
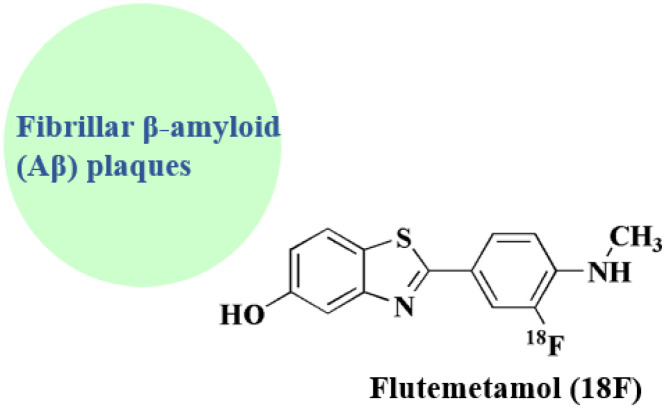	**Flutemetamol (** ^**18**^ **F)** • The principal molecular target of flutemetamol (^18^F) is the fibrillar *β*-amyloid (*Aβ*) plaques. The structure that is being targeted is the cross *β*-sheet conformation in insoluble *Aβ* aggregates
	• There is no discernible binding to soluble or monomeric Aβ forms; instead, there is high-affinity, selective binding to dense-core amyloid plaques^113^
	Imaging mechanism and brain uptake:
	• PET imaging is made possible by the radiotracer fluorine-18 isotope. It efficiently penetrates the blood–brain barrier (BBB)^114^
	It does not interact with neurotransmitter receptors or intracellular signaling pathways; solely used for diagnostic purposes; not therapeutic
	• It is used in amyloid pathology detection and measurement in mild cognitive impairment, Alzheimer's disease, and indeterminate etiology dementia^115^
	
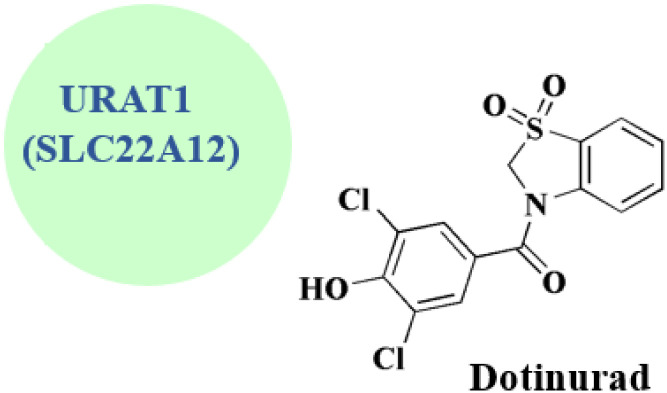	**Dotinurad** • URAT1 (SLC22A12) is the primary molecular target of Dotinurad. It is encoded by the SLC22A12 gene and is located in the proximal renal tubular cells' apical membrane. It acts as a selective inhibitor and promotes uricosuria, or increased excretion of uric acid, by preventing the renal tubule from reabsorbing uric acid into the bloodstream. It has the effect of lowering serum uric acid levels without substantially altering other renal transporters^116^
	• It increases its effectiveness and decreases off-target effects by specifically inhibiting URAT1 without substantially altering other renal urate transporters like OAT1/3 or ABCG2 (ref. 117)
	• Pharmacodynamic and clinical impacts:
	It reduces blood uric acid levels in both hyperuricemia patients and healthy individuals in a dose-dependent manner
	It is favorable, with a low incidence of hepatic or renal side effects compared to previous uricosurics like benzbromarone; half-life: around 10 hours, permitting once-daily dosing^118,119^
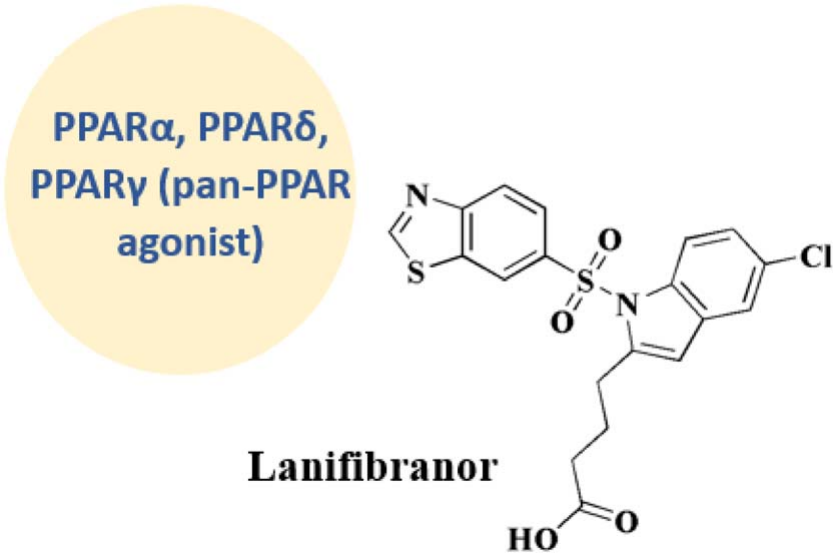	**Lanifibranor** • Lanifibranor has a triple agonist action on PPARα, PPARδ, and PPARγ, and impact on metabolic and inflammatory pathways^120^ Lanifibranor modulates gene expression *via* PPARs^121,122^
	
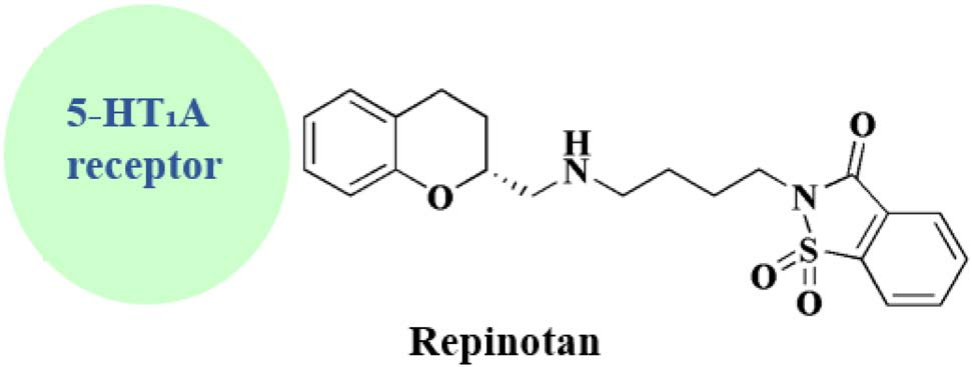	**Repinotan** • Mechanism of action & preclinical neuroprotection: Up to five hours after a stroke, repinotan dramatically decreased the infarct volume in rats
	• Mechanism: excitotoxic damage is decreased by high-affinity 5 HT_1_A receptor agonism^123^
	• In a clinical trial in early stage: BRAINS study
	IV repinotan (0.5–2.5 mg per day) is being evaluated in a phase II trial for acute stroke. It displayed some non-significant favorable trends and tolerability^124^
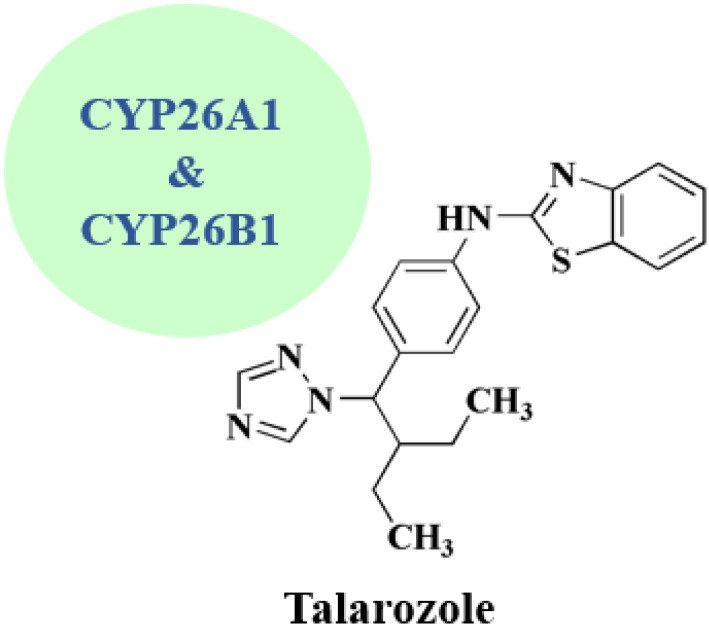	**Talarozole** • Talarozole (R115866) is consiered as a selective and active CYP26A1/B1 inhibitor with nanomolar potency^125^
	• Talarozole exhibits anti-inflammatory effects of in joint cartilage injury models^126^
	
	
	
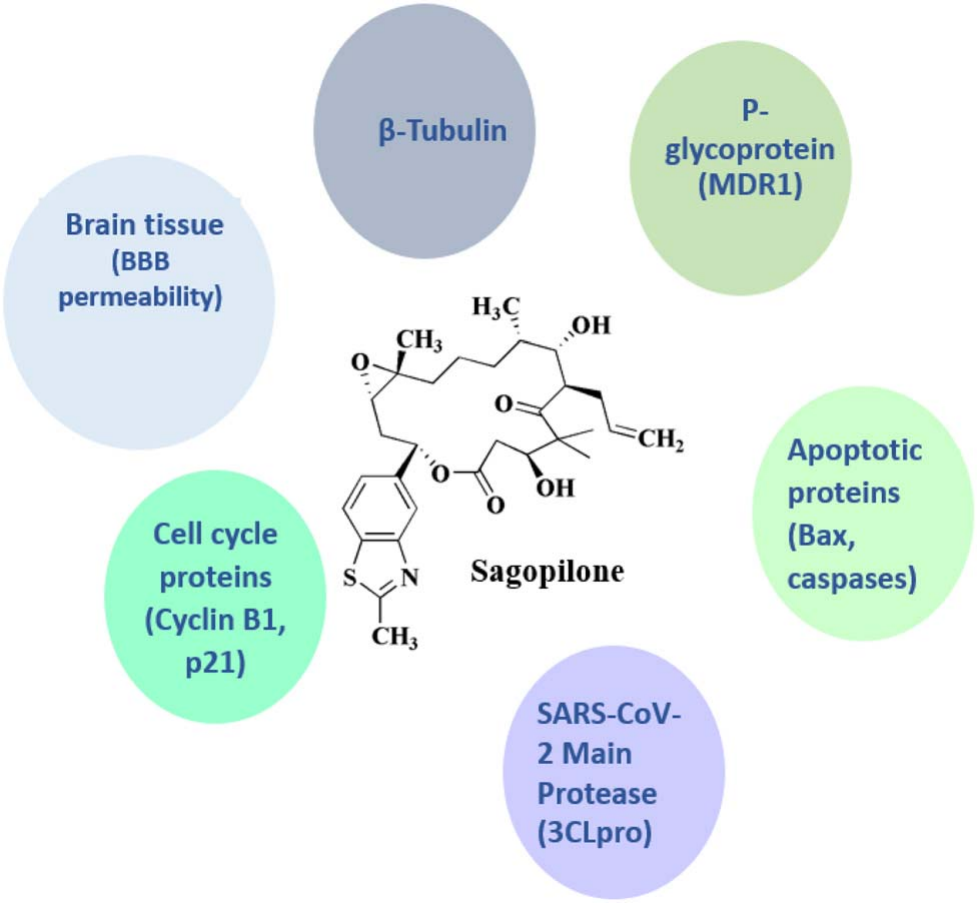	**Sagopilone** • Sagopilone's primary target is the *β*-tubulin (microtubules). *β*-Tubulin is a key structural protein of microtubules. Its mechanism is to attach to the taxane-binding site on *β*-tubulin, stimulate tubulin polymerization, and stabilize microtubules against depolymerization. Cell cycle arrest at the G_2_/M phase and apoptosis in proliferating tumor cells are the results of this disturbance of mitotic spindle dynamics^127^
	• Regarding the multidrug resistance proteins (*e.g.*, *P*-glycoprotein/MDR1) sagopilone is not a target, although *P*-glycoprotein (ABCB1) recognizes it poorly
	• Sagopilone circumvents a major chemoresistance mechanism by maintaining cytotoxic activity in MDR1-overexpressing cancer cells, in contrast to taxanes^128^
	• Even though *β*-tubulin is the only direct molecular target, sagopilone has downstream effects that include indirect modulation of the pro-apoptotic pathways, cell cycle regulators, and the activation of mitotic checkpoints^129^
	• Blood–brain barrier penetration: Unlike paclitaxel and other microtubule drugs, sagopilone penetrates the brain. This enables the targeting of brain malignancies like glioblastoma
	• This impacts therapeutic value even though it is a pharmacokinetic characteristic rather than a receptor interaction^130^
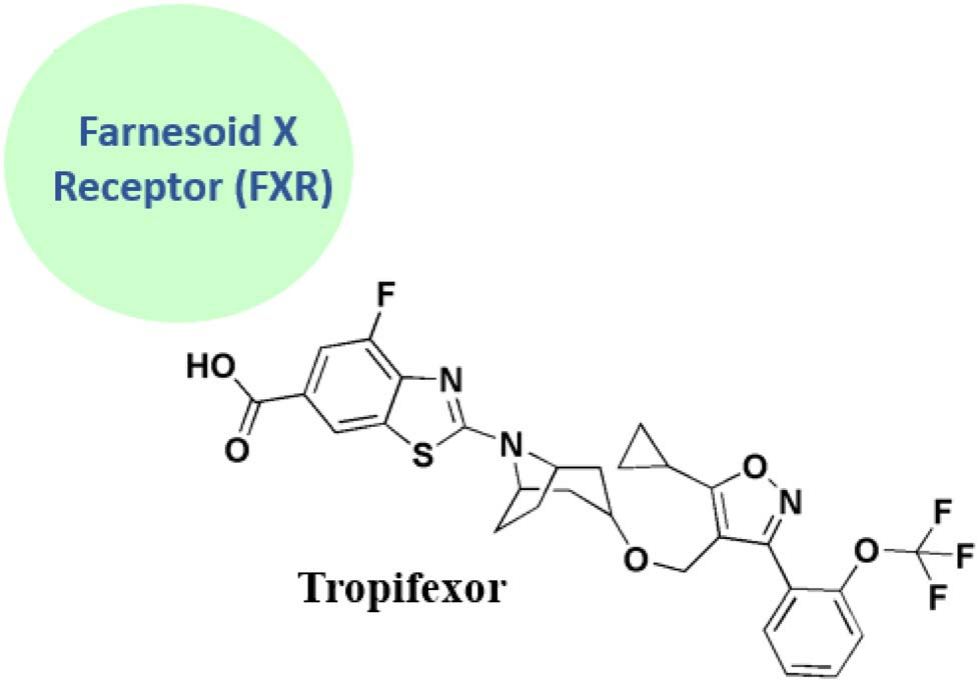	**Tropifexor** • Tropifexor is considered as a highly selective FXR agonist^131^
	• According to a pre-clinical study in NASH models it demonstrates anti-fibrotic & anti-steatotic effects through FXR activation in rodents^132^
	• FXR-tropifexor structural interaction is detected as its crystal structure insights into FXR-ligand binding specificity is provided^133^
	• Tropifexor's dose-dependent reduction in liver fat and ALT, along with adverse effects (LDL rise, pruritus), is reported in the FLIGHT-FXR trial, a clinical study in NASH^134^
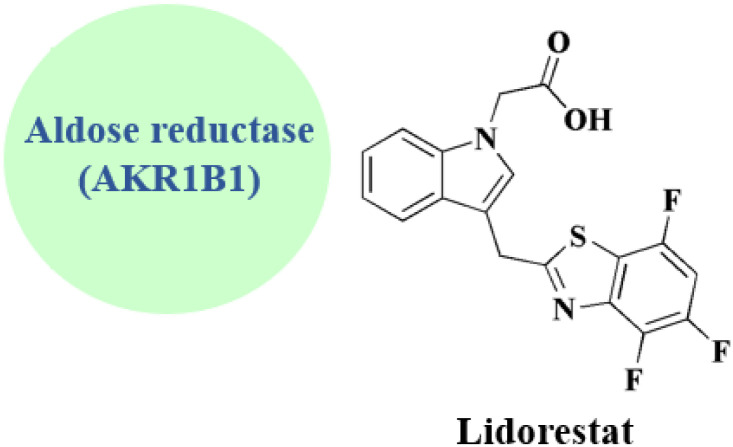	**Lidorestat** • Lidorestat is considered as an aldose reductase (AKR1B1) selective competitive inhibitor
	• Lidorestat binds to the active site of aldose reductase (AKR1B1), preventing the conversion of glucose to sorbitol, the initial and rate-limiting step in the polyol pathway. Aldose reductase (AKR1B1) is the primary and direct target. It is a member of the aldo-keto reductase superfamily. It acts as a selective and strong competitive inhibitor. When this route is overactivated under hyperglycemic settings, tissues like the heart, kidney, retina, and nerves experience oxidative stress, sorbitol buildup, osmotic damage, and secondary inflammation
	• Aldose reductase inhibition aims to stop the development of diabetes complications, including cardiomyopathy and nephropathy, without interfering with regular glucose metabolism^135^
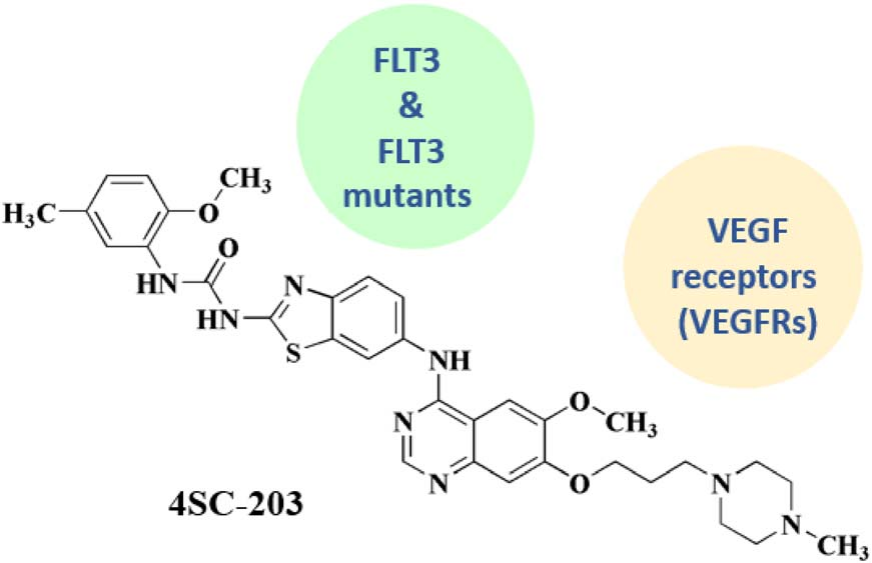	**4SC-203** • Official declaration of the start of a clinical trial characterizes 4SC 203 as a selective kinase inhibitor that primarily targets VEGF receptors and FLT3 (including mutant versions). Pharmacokinetics, safety, and tolerability were evaluated in a clinical trial^136^
	
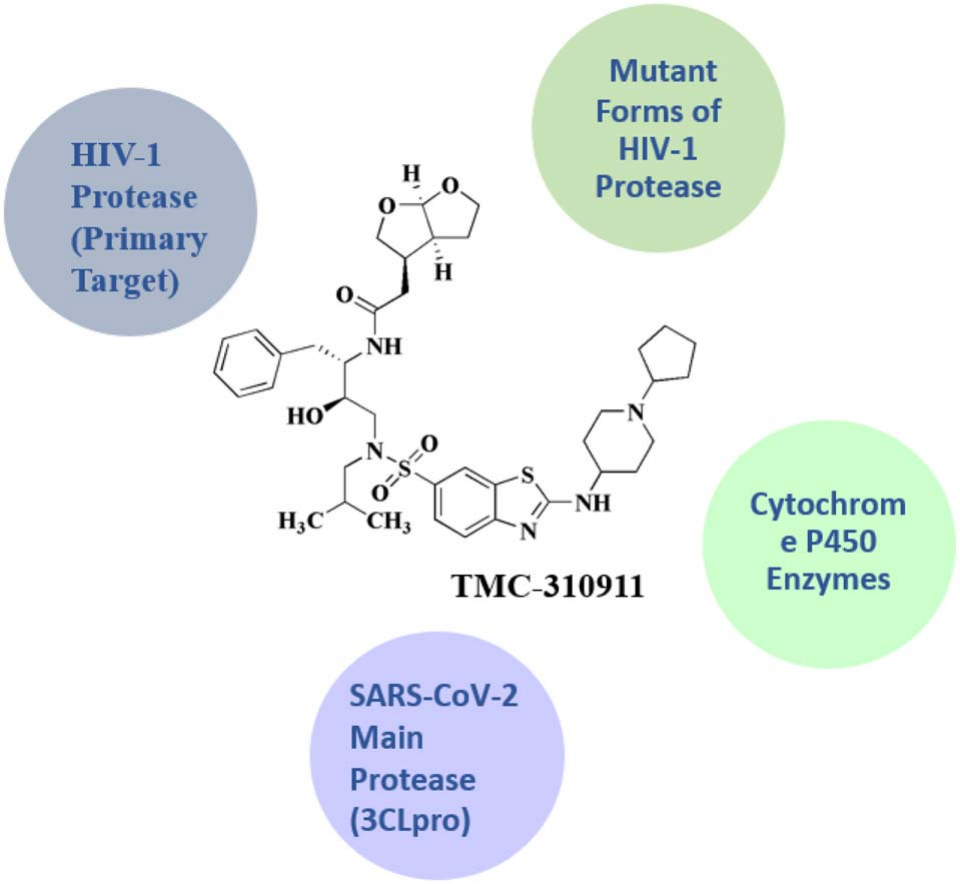	**TMC-310911** • HIV-1 aspartyl protease (EC 3.4.23.16) is the target of TMC-310911. It acts as a competitive inhibitor
	• TMC-310911 binds to the active site of the HIV-1 protease enzyme, preventing the cleavage of viral gag and gag-pol polyproteins into functional proteins (*e.g.*, capsid, reverse transcriptase, integrase, protease).When this process is disrupted, immature, non-infectious virions are formed^137^
	• It targets HIV-1 protease variants with resistance mutations; it preserves inhibitory activity against numerous drug-resistant HIV strains; TMC-310911 was designed especially to preserve effectiveness against HIV strains that are resistant to multiple drugs along with an enhanced genetic barrier to resistance^138^
	• It targeted the cytochrome P450 enzymes; they act as a substrate and a mild inhibitor; it is relevant because similar to darunavir, ritonavir or cobicistat boosting is necessary for TMC-310911 to enhance plasma exposure through CYP3A4-mediated metabolic inhibition. It might also impact how other drugs that are co-administered and processed by CYP3A4 are metabolized^139^
	• The main protease of SARS-CoV-2 (3CLpro): a potential or investigative target
	• TMC-310911 targets SARS-CoV-2's 3-chymotrypsin-like protease (Mpro, 3CLpro); it ats as a weak inhibitor (*in vitro* and *in silico* research)
	• TMC-310911's protease-inhibitory scaffold led to its evaluation as a repurposing candidate during the COVID-19 pandemic. Nevertheless, there was no compelling preclinical or clinical data to support substantial anti-SARS-CoV-2 efficacy^140^
	
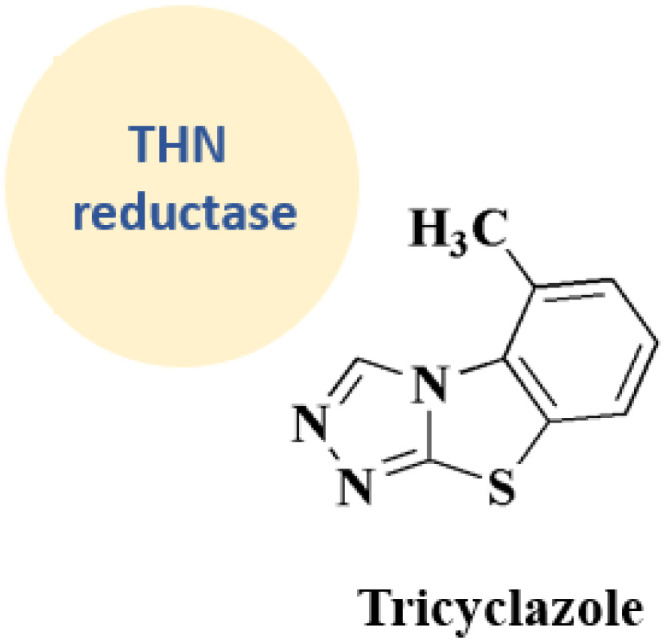	**Tricyclazole** • Tricyclazole's known enzyme target is tetrahydroxynaphthalene reductase (THN reductase)
	• Its mechanism of action depend on the 1,8-dihydroxynaphthalene melanin route, or DHN-melanin production pathway; primarily in fungi like alternaria alternata and magnaporthe oryzae
	• A crucial stage in the biosynthesis of fungal melanin is the reduction of 1,3,6,8-tetrahydroxynaphthalene (THN) to scytalone, which is catalyzed by the enzyme THN reductase, which is inhibited by tricyclazole. The following functions of this melanin are essential: protection against oxidative stress and host immunological responses; appressorium turgor pressure, which is necessary for host penetration
	Because THN reductase is blocked, fungi are unable to synthesize melanin and are unable to effectively penetrate plant tissue, which reduces their virulence and pathogenicity^141,142^
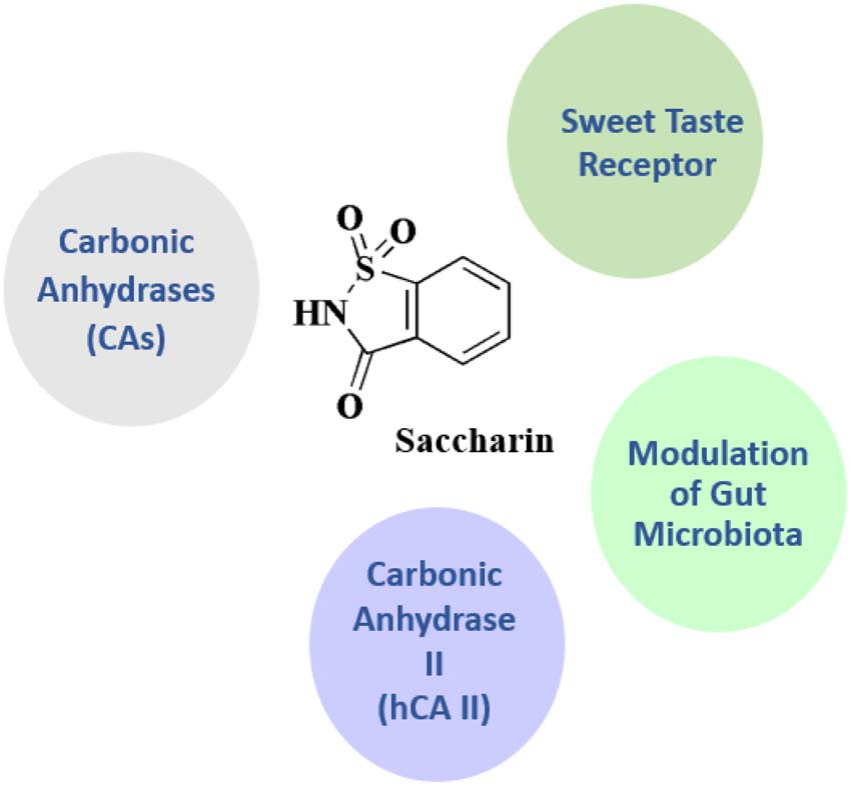	**Saccharin** • Saccharin targets the carbonic anhydrases (CAs) particularly the human carbonic anhydrase isoenzymes, hCA IX and XII
	• CAs are zinc metalloenzymes that regulate pH and are overexpressed in cancers. Saccharin and its derivatives have shown specific inhibition of tumor-associated CAs, especially CA IX, making them intriguing anticancer leads^143,144^
	• It targets sweet taste receptor, saccharin binds and activates the sweet taste receptor complex (T1R2 + T1R3), specifically connecting with extracellular Venus flytrap domains. It targets the G-protein coupled receptor (GPCR) T1R2/T1R3. Its sweetening ability stems from this.^145,146^ It target human carbonic anhydrase II (hCA II) as a weak inhibitor; saccharin can weakly inhibit hCA II, suggesting a broad CA interaction, although less selectively than for hCA IX/XII^147^
	• Saccharin modulate the gut microbiota, it indirect interacts with gut microbial species (*e.g.*, clostridium, bacteroides); it has modulatory effect on microbiota composition and function; long-term exposure to saccharin can result in dysbiosis of the gut microbiota, which is associated with metabolic changes and glucose intolerance^148,149^

## 6. Synthetic strategies for novel anti neurodegenerative benzothiazoles

### 6.1. Oxadiazole-benzothiazole based analogues

**Scheme 12:**
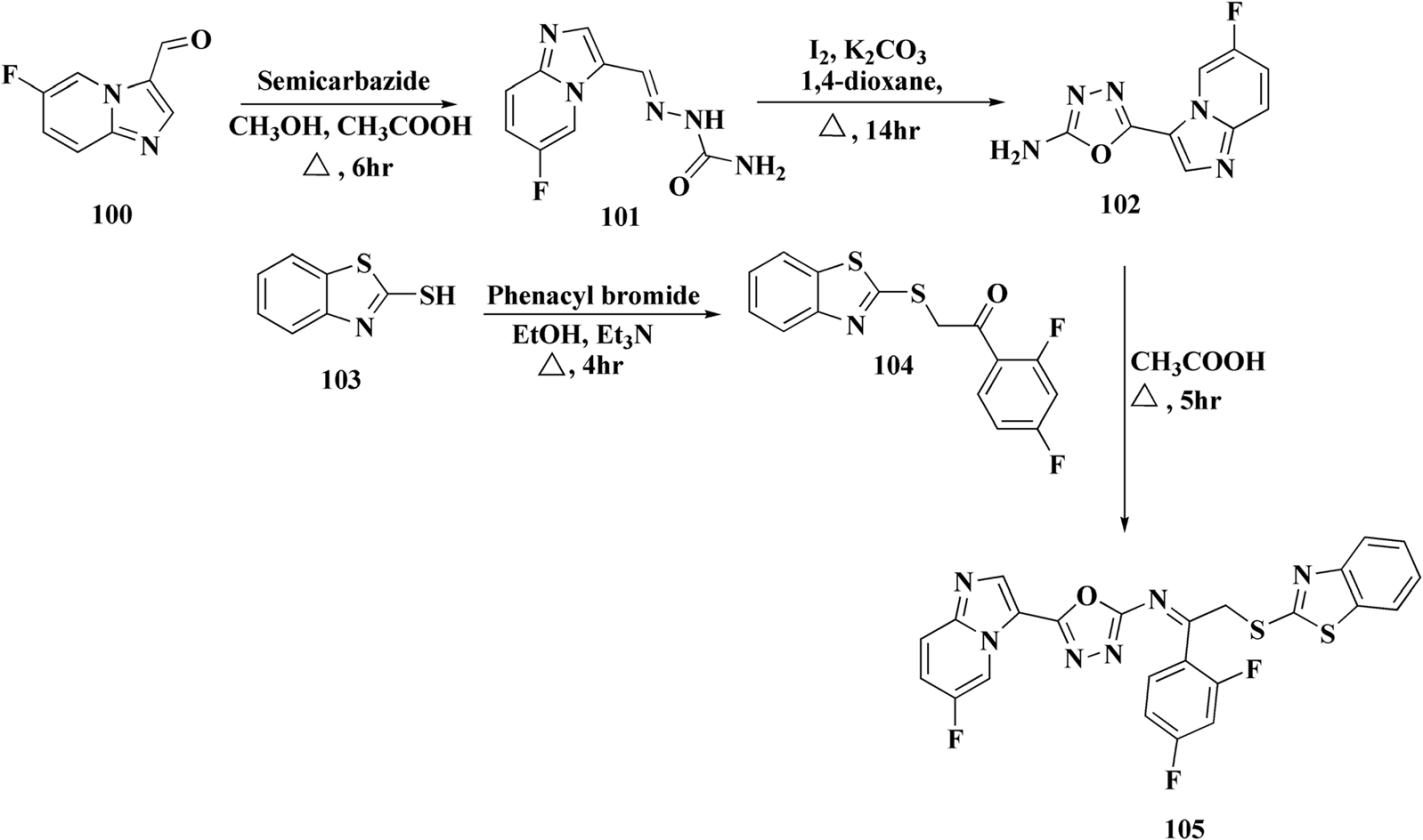
Synthesis of imidazopyridine-based benzothiazole–oxadiazole.

The synthesis of the imidazopyridine-based benzothiazole–oxadiazole analogs as anti-Alzheimer's agents was accomplished in Scheme 12. Treatment of 6-fluoroimidazo[1,2-*a*]pyridine-3-carbaldehyde 100 with carbamylhydrazine in methylalchlol in the presence of ethanoic acid, under reflux yielded the hydrazine based intermediate 101. Further reaction of the latter intermediate with potassium carbonate and iodine to access the cyclized intermediate 102, under reflux condition. Synthesizing the imidazopyridine-based benzothiazole–oxadiazole hybrid 105 was accomplished *via* treating intermediate 102 with the substituted benzothiazole. These analogs were accessed *via* reacting 2-benzothiazolethiol (MBT) with several substituted 2-bromo-1-phenylethanone.^150^

The efficiency of these compounds was estimated comparable to donepezil as a standard drug (IC_50_ value 19.90 ± 2.40 μM for BuChE & 14.47 ± 1.20 μM for AChE). The new scaffolds displayed potency in a range of IC_50_ value 6.40 ± 1.80 μM for AChE and 6.70 ± 1.65 μM & 41.65 ± 7.20 μM for AChE & 44.65 ± 7.40 μM for BuChE. Compound 105 with IC_50_ value 6.40 ± 1.80 μM for BuChE and 6.70 ± 1.65 μM for AChE was considered as the strutural optimization of the compounds with the maximum inhibition as the result of the presence of small-sized and the highly electro-negative fluoro scaffolds that inhibite the enzymes *via* the formation of hydrogen bonds.

### 6.2. Piprazine–benzothiazole based analogues

**Scheme 13:**
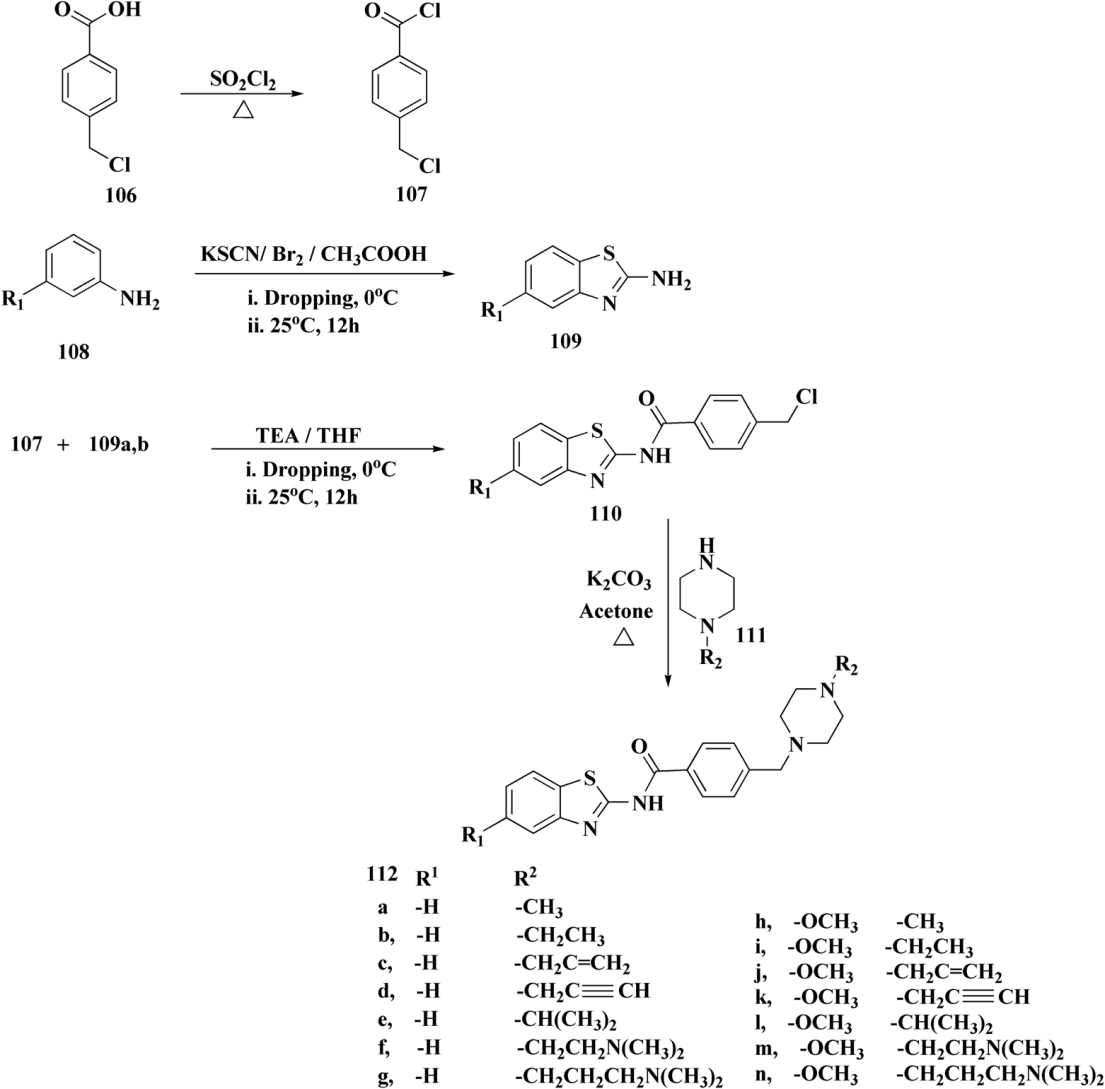
Synthesis of substituted benzamides.

The usage of dual acetylcholinesterase (AChE) monoamine oxidase B inhibitors is a novel strategy in treating Alzheimer's disease. New compounds were designed and synthesized to target those enzymes. Novel compounds comprising piperazine and benzothiazole was synthesized as depicted in Scheme 2. 4-Chloromethylbenzoyl chloride 107 was synthesized utilizing sulfinyl chloride. 2-Benzothiazolamine 109a–b analogs were synthesized *via* ring closure reaction utilizing brome soln. The substituted benzamides 110a–b were accessed *via* reacting 4-chloromethyl benzoyl chloride 107 and 2-benzothiazolamine 110a–b. Compounds 110a,b & piperazine analogs were allowed to react to afford compounds 112a–n (Scheme 13).^151^

**Fig. 6:**
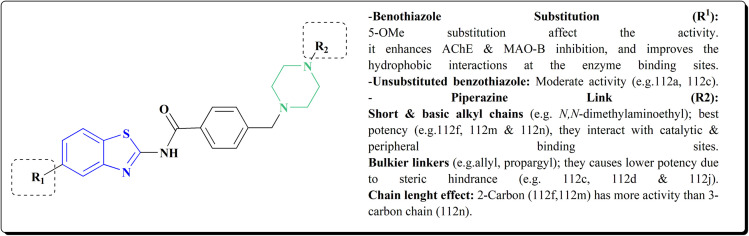
Structure–activity relationship of compound 112.

The inhibitory potential of all compounds against butyrylcholinesterase (BChE), AChE, MAO-A & MAO-B was estimated utilizing an *in vitro* fluorometric procedure. In addition, the inhibitory effect of the potent compounds on amyloid-beta (Ab) aggregation was estimated *in vitro*. Biological investigation revealed that compounds 112a, 112d, 112f, 112h, 112k & 112m exhibited significant potency against MAO-B & AChE enzymes (Fig. 6). Compound 112f inhibited MAO-B and AChE enzyme with IC_50_ values of 23.4 to 1.1 nM & 40.3 to 1.7 nM. It was found that compound 112f may inhibit AChE & MAO-B enzymes potentially, besides the capability to inhibit the beta amyloid plaques's formation accumulated in the brains of AD patients. *In silico* investigations correspondingly support the resultant bioactivity. Compound 112f induced a strong interaction with the active site of the two enzymes. The interaction of flavin adenine dinucleotide (FAD) with compound 112f in the active site of the MAO-B enzyme active site is an exciting discovery.

It is presumed that multi-targeted directed ligands (MTDLs) which interact with various targets related to Alzheimer's disease (AD), might offer an enhanced therapeutic alternative than utilizing the “one-target, one-molecule” strategy. Novel benzothiazole-based analogs were described as a privileged moiety for histamine H3 receptor ligands (H3R). The most affine compound, the propyloxy-linked benzothiazole analog 112b, exhibited a *K*_i_ value of 0.012 lM. The multi-targeting potential of these H3R ligands towards AChE, BuChE & MAO-B enzymes was estimated to afford compound 112f as the furthermost promising MTDL with a Ki value of 0.036 lM at H3R and IC_50_ values of 6.7 mM, 2.35 mM, & 1.6 mM towards AChE, BuChE, & MAO-B. These results propose that compound 112f can be a structural optimization for novel multitargeting anti-AD agents.^151^

**Scheme 14:**
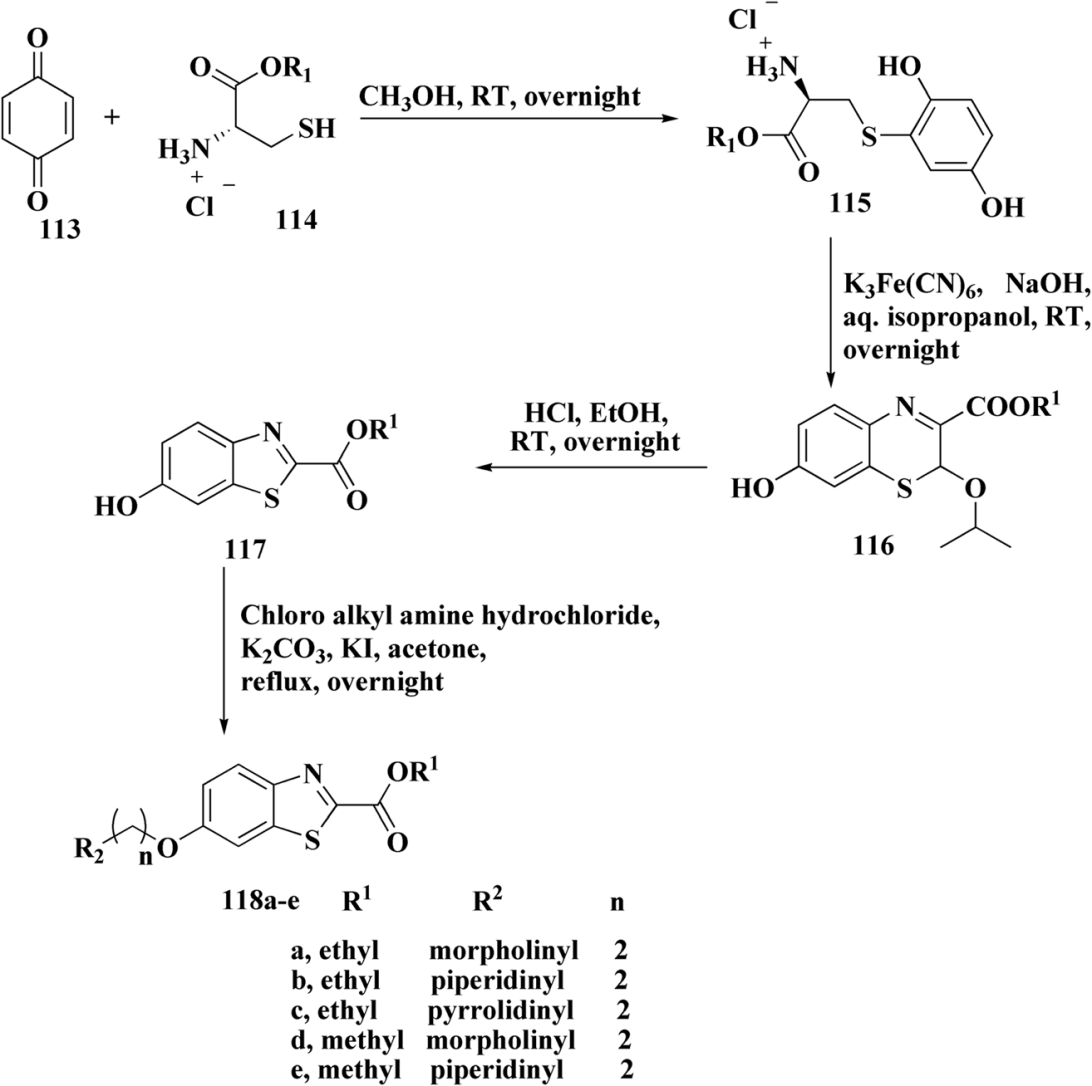
Synthesis of methyl/ethyl 6-hydroxybenzothiazole-2-carboxylates.

**Scheme 15:**
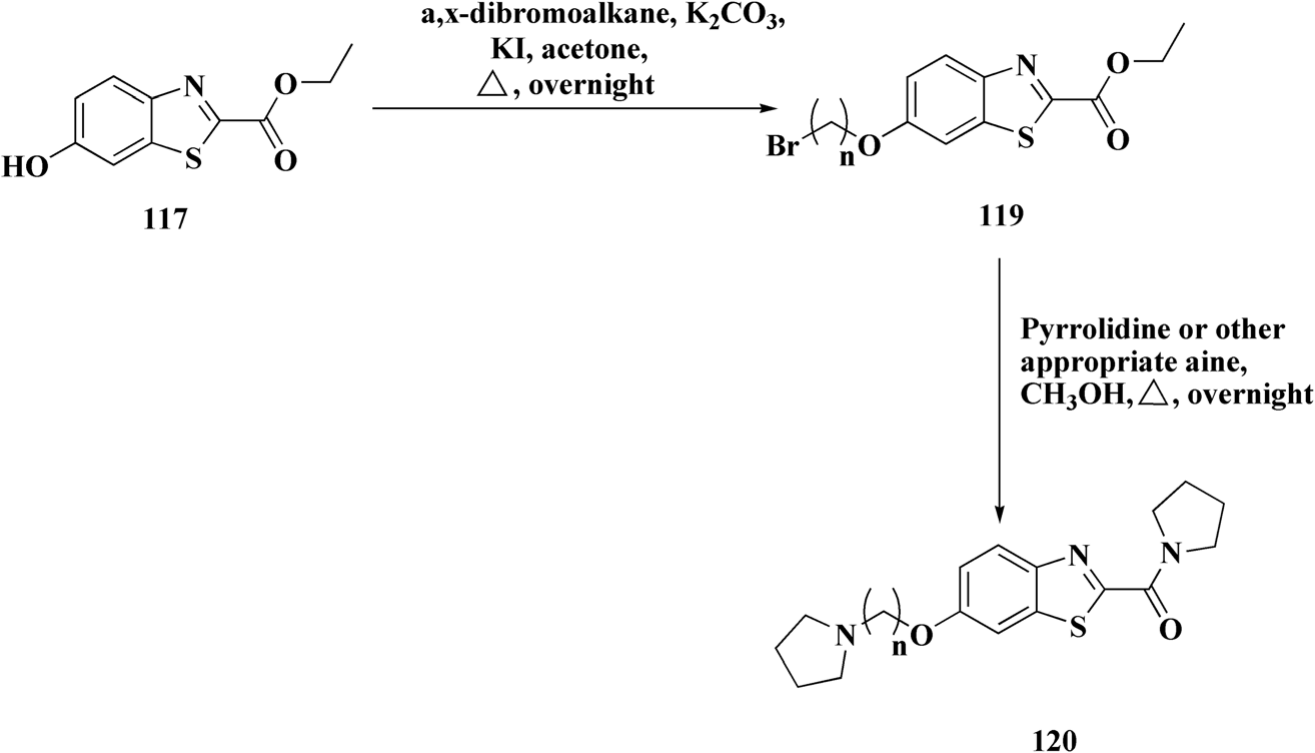
Synthesis of methyl/ethyl 6-hydroxybenzothiazole-2-carboxylates.

The methyl/ethyl 6-hydroxybenzothiazole-2-carboxylate was synthesized as depicted in Schemes 14 & 15. The quinone was reacted with cysteine methyl/ethyl ester hydrochloride *via* Michael addition to give the hydroquinone. Then, the oxidation of the hydroquinone intermediate using potassium ferric hexacyanoferrate afforded the benzothiazine analog. The further contraction to benzothiazole ring in acidic medium gave compounds 117a & 117b. This mechanism was suggested to occur through benzothiazine ring hydrolysis to afford a mercaptoaldehyde analog, intramolecular attack inorder to form the contracted ring, which was followed by oxidation and decarboxylation of the aldehyde group. Compounds 117a (R^1^ = CH_3_) & 117b (R^1^ = CH_2_CH_3_) were allowed to reflux with various chloroalkyl amines to afford compounds 118. The 6 hydroxybenzothiazole-2-carboxylate methyl ester (117a) was reacted with the α,ω-dibromoalkane to yield an ether in the presence of potassium iodide and potassium carbonate to give compounds 119, which were then reacted with the methyl ester of various aromatic amino acids under reflux to afford compounds 120.^152^

**Fig. 7:**
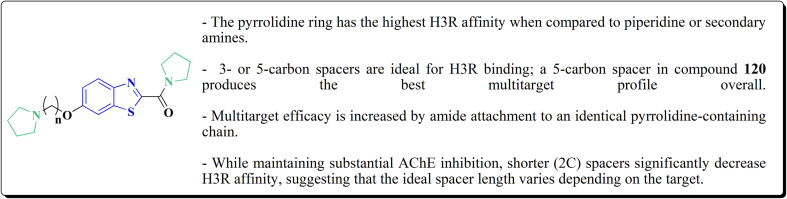
Structure–activity relationship of compound 120.

The struture–activity relationship of compound 120 is shown in Fig. 7. Regarding the H3R binding model, the Glu176 and protonated pyrrolidine nitrogen form a salt bridge. Phe163-CH–π interaction adds extra CH–π with Trp341. Glu176 (CH⋯O), Cys88 (CH⋯S), and Arg351 (carbonyl group) are hydrogen bonds. With an essential ionic connection to Glu176, the binding mode is similar to those of known inverse agonists (ABT-239, JNJ5207852).

Crystal structure of AChE complexed with PDB ID: 4EY5 indicated that the pyrrolidine ring is near the anionic site and catalytic triad. The molecule is held in place by CH–π interactions with Tyr337, cation–π interactions with Trp86, and hydrogen bonds with Glu202 and Phe295.

In case of Inhibition of MAO-B the compound probably entails binding inside the hydrophobic substrate cavity; compound 120 demonstrates MAO-B selectivity over MAO-A, which could be related to amide orientation and spacer length.^152^

### 6.3. Benzothiazoles linked with substituted aromatics

α-Synuclein (α-syn) & Tau aggregates are the key histopathological hallmarks in Parkinson's disease (PD), Alzheimer's disease (AD), and many other neurodegenerative diseases. Alternatively, mis-folding of α-synuclein is considered as a distinguishing feature in PD & dementia with Lewy bodies (DLB). Targeting of proteinaceous oligomers & aggregates remains challenging.

**Scheme 16:**
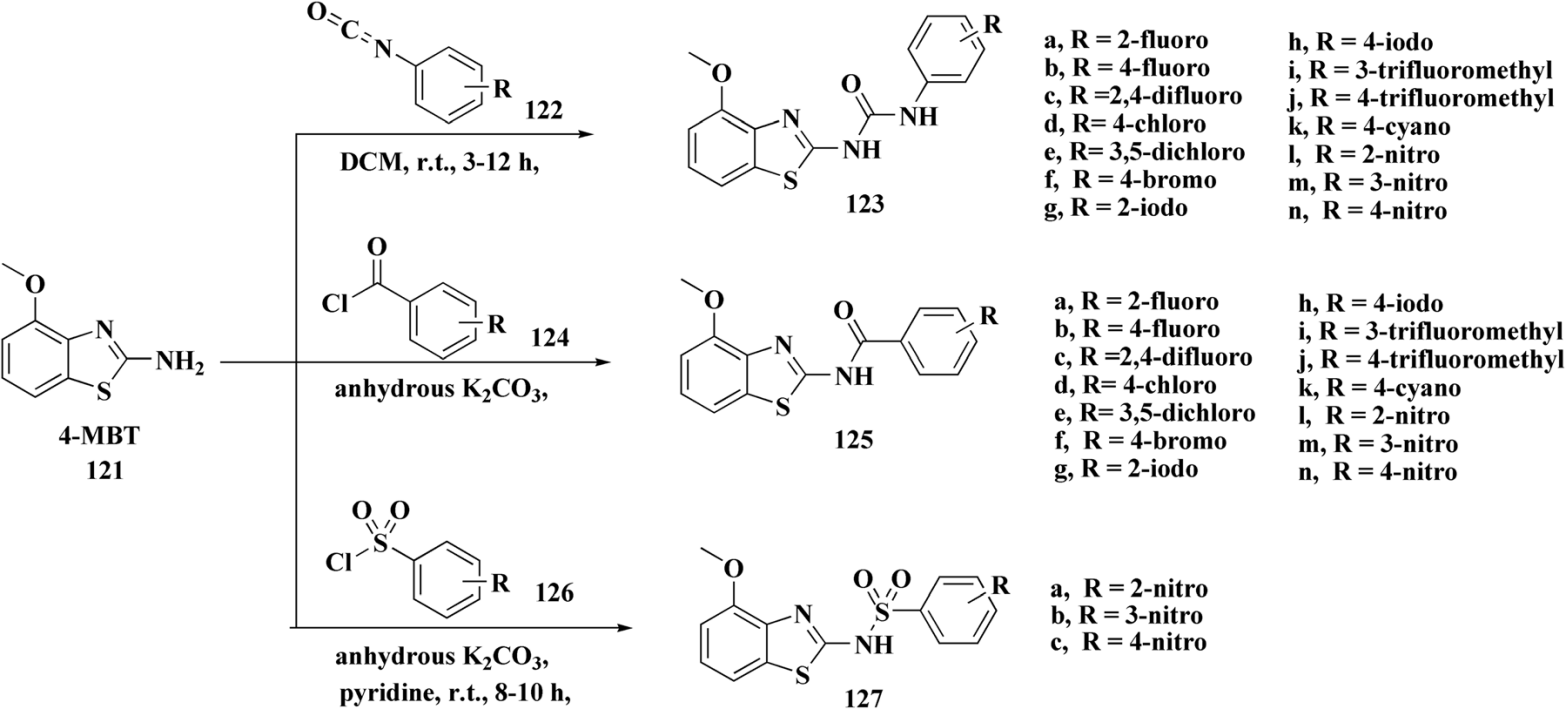
Synthesis of substituted benzothiazol-2-amine.

The benzothiazole-based compounds were synthesized utilizing the structural hybridization approach among the benzothiazole cyanine dye & the diphenyl pyrazole analog that revealed anti-aggregation potency towards α-syn & 2N4R tau. The targetedcompounds were synthesized utilizing 4-methoxy-1,3-benzothiazol-2-amine (4-MBT). To synthesize compounds comprising the urea linker, the phenyl isocyanate in a quantitative amount was utilized in methylene chloride. The sulfonamide or the amido counterparts were furnished using benzene sulfonyl chloride or benzoyl chloride, in the presence of pyridine and anhydrous potassium carbonate (Scheme 16).^153^

The antiaggregation impact of the compounds was investigated utilizing the thioflavin-T fluorescence assay, whereas transmission electron microscopy was used for the detection of fibrils utilizing the ThT assay *via* the accomplishment of a time course research. Meanwhile, the photoinduced cross-linking of the unmodified protein assay (PICUP assay) was utilized to identify the formation of oligomers.

## 7. Synthetic strategies for novel anti-inflammatory benzothiazoles

### 7.1. Thiazole–benzothiazole based analogues

Non-Steroidal bioactive heterocyclic compounds 132, 134 & 140 were synthesized starting from the 2-chloro-1-(2-(4-chlorophenyl)benzothiazol-3(2*H*)-yl) ethanone 128. These novel compounds were estimated for their analgesic, anti-inflammation, ulcerogenic, acute toxicity & free-radical scavenging action as compared to reference drugs in the albino rats. At a dose of 50 mg per kg p.o. compound 132c showed more potency than reference drug.

**Scheme 17:**
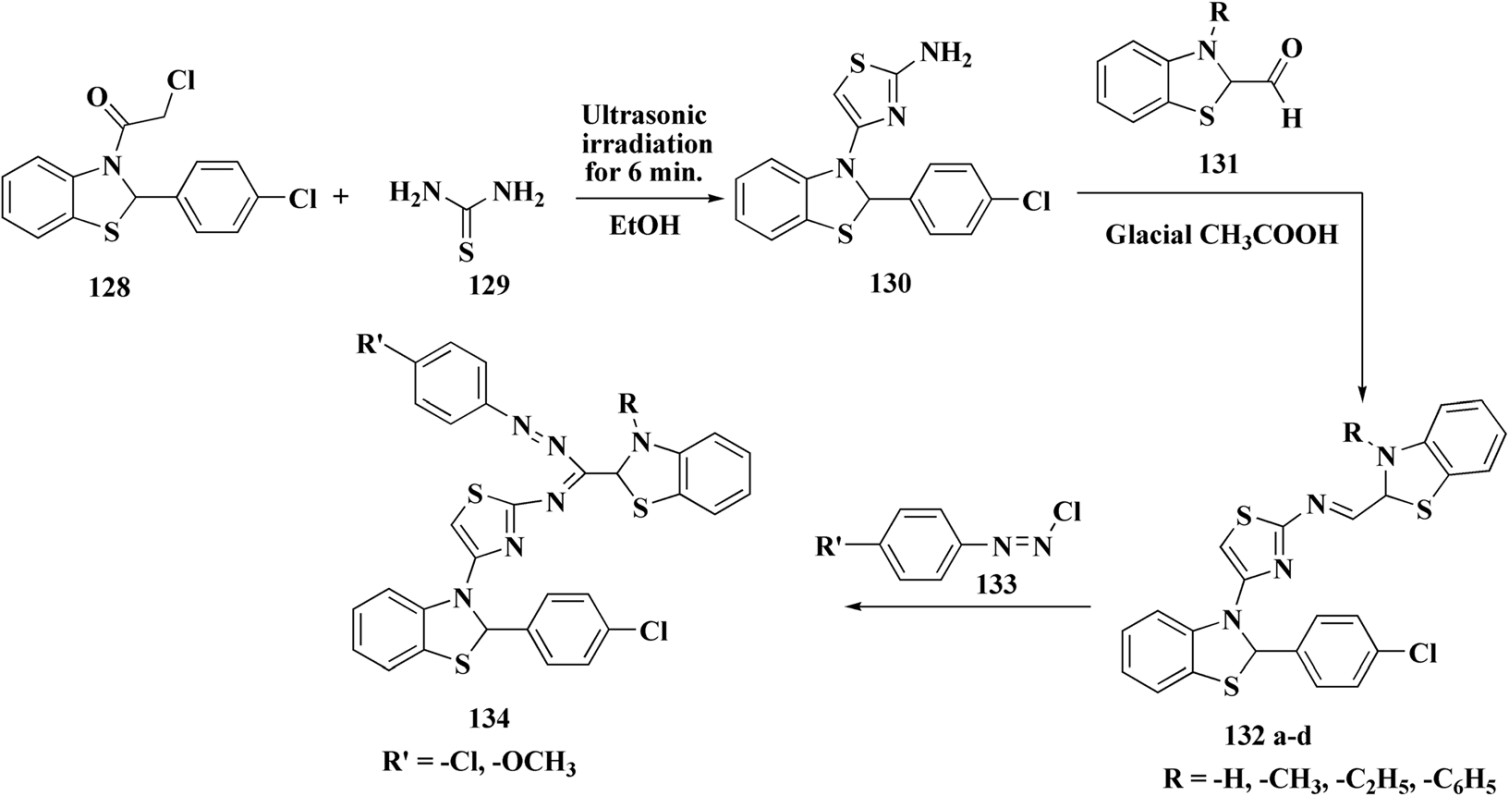
Synthesis of the substituted 2-hydrobenzothiazole-2-carboxamidines.

The synthesis of 2-chloro-1-(2-(4-chlorophenyl)benzothiazol-3(2*H*)-yl) ethenone (128) was started *via* reacting 2-(4-chlorophenyl)-2,3-dihydrobenzothiazole with chloroacetyl chloride. The latter compound was reacted with thiourea to generate the substituted (benzothiazol-3(2*H*)-yl)thiazol-2-amine (130). Further reaction of compounds 130 with the substituted hydrobenzothiazole-2-carboxaldehyde to generate compound 132 which on further reaction with diazonium salts 133 furnished the substituted 2-hydrobenzothiazole-2-carboxamidine 134 (Scheme 17).^154^

### 7.2. Oxazole–benzothiazole based analogues

**Scheme 18:**
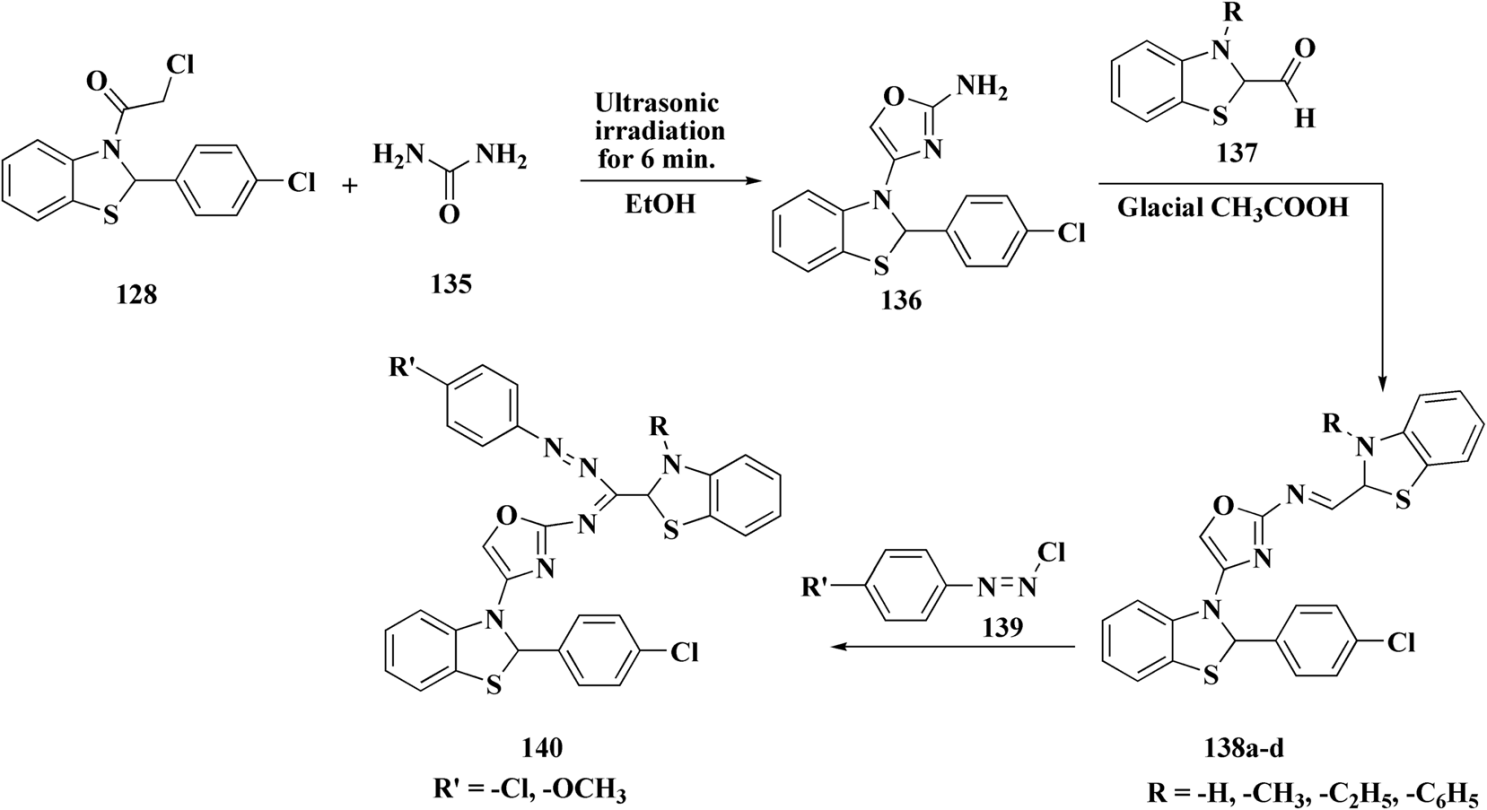
Synthesis of benzothiazole-2-carboxamidine derivatives.

**Fig. 8:**
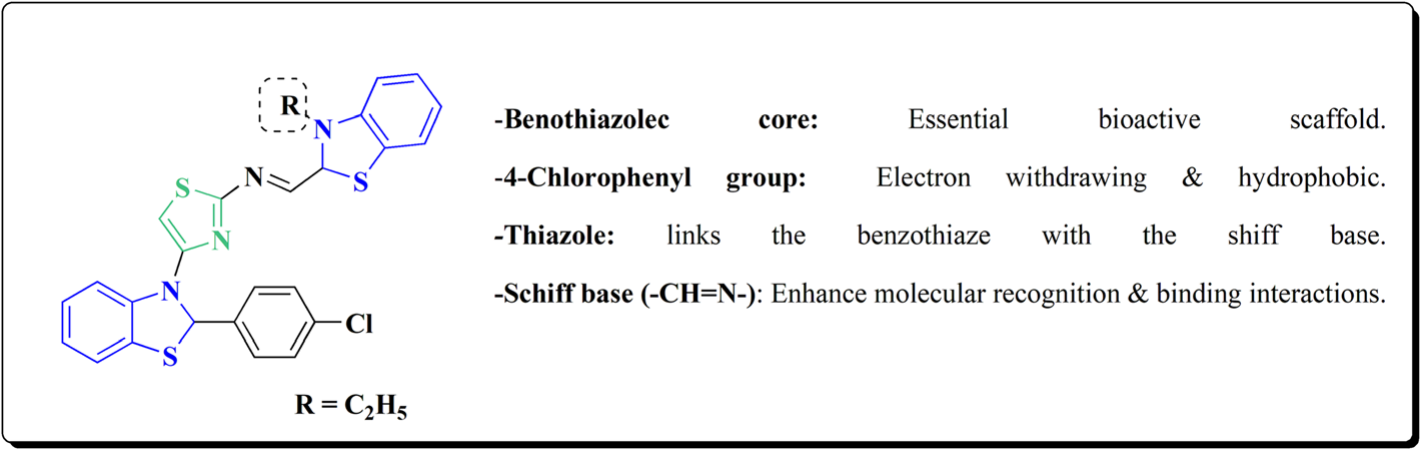
Structure–activity relationship of compound 132c.

The reaction of compound 77 with urea afforded compound 136. The latter compound was reacted with 3-substituted-2-hydrobenzothiazole-2-carboxaldehyde to produce the substituted (benzothiazol-2-yl)methylene)oxazol-2-amine 138 which on further reaction with the substituted aniline synthesized the benzothiazole-2-carboxamidine derivative 140 (Scheme 18, Fig. 8).^154^

### 7.3. Oxadiazole–benzothiazole based analogues

**Scheme 19:**
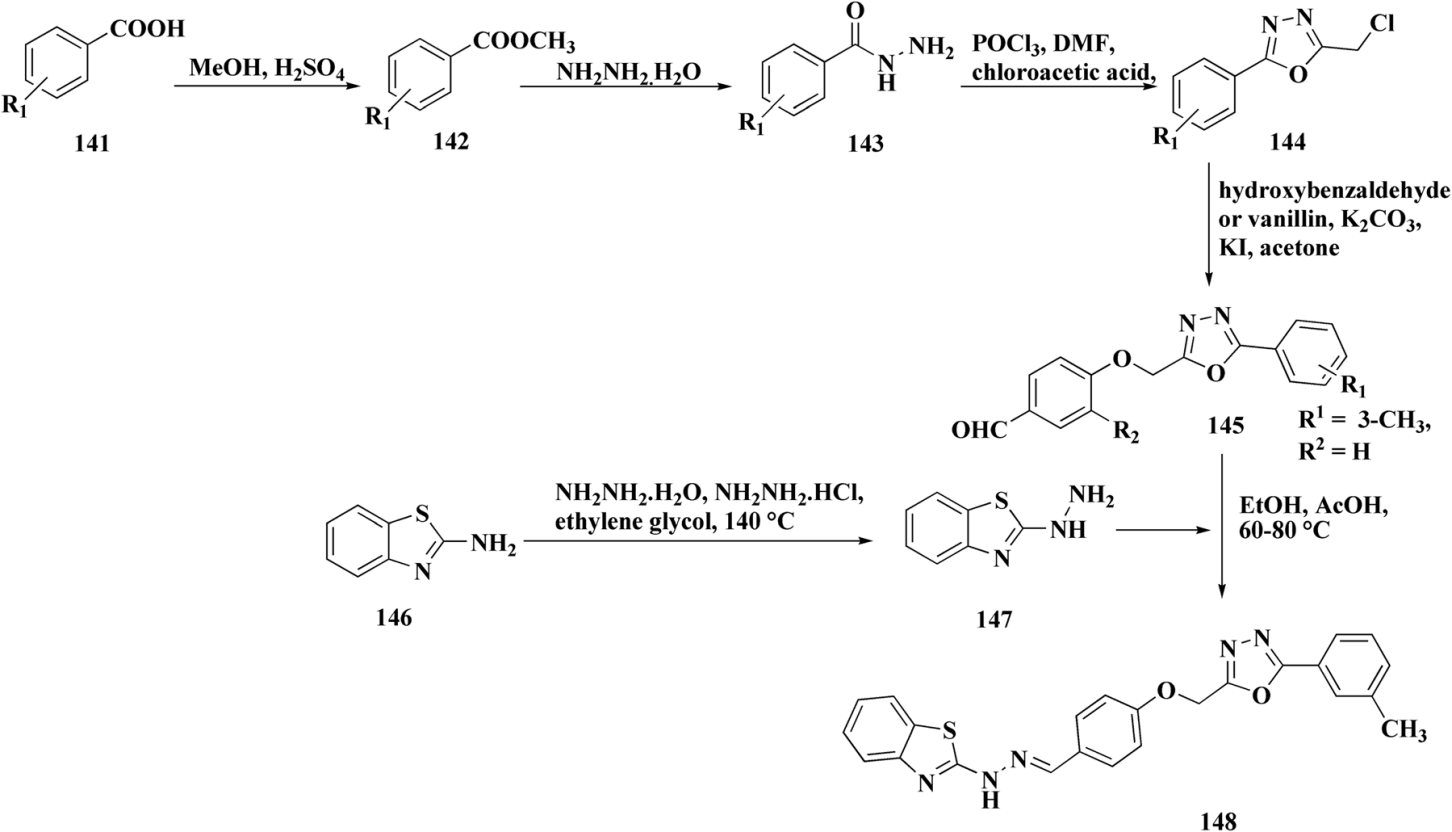
Synthesis of benzothiazole-1,3,4-oxadiazole conjugates.

**Fig. 9:**
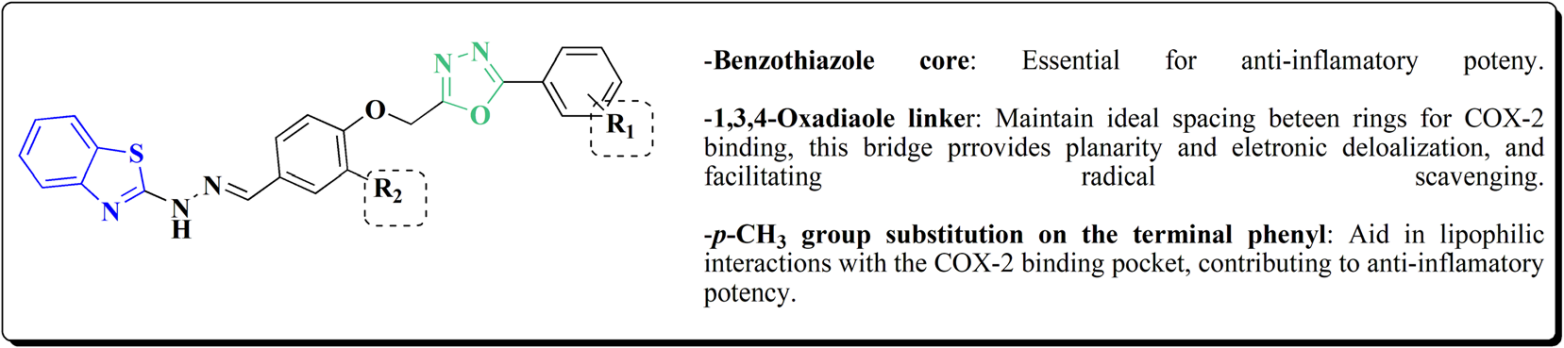
Structure–activity relationship of compound 148.

Benzothiazole analogs bearing a 1,3,4-oxadiazole scaffold were synthesized (Scheme 19) and investigated for their anti-inflammatory and anti-oxidant potencies (Fig. 9). Compound 148 possessed high radical scavenging efficacies using the ABTS+% bioassay. Upon anti-inflammatory assessments, compound 148 exhibited good potency with 57.35% inhibition after intraperitoneal administration, which was more active than indomethacin as a reference drug. Molecular modeling investigations were carried out to estimate the binding mode of compound 148 as a representative structure into COX-2 enzyme. *In vitro* enzyme investigation indicated that compound 148 displayed its anti-inflammatory potency *via* COX-2 inhibition.^155^

### 7.4. Benzothiazoles linked with substituted aromatics

**Scheme 20:**
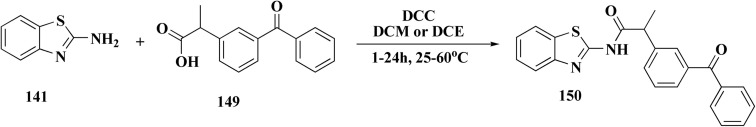
Synthesis of 2-aminobenzothiazole analogs.

**Fig. 10:**
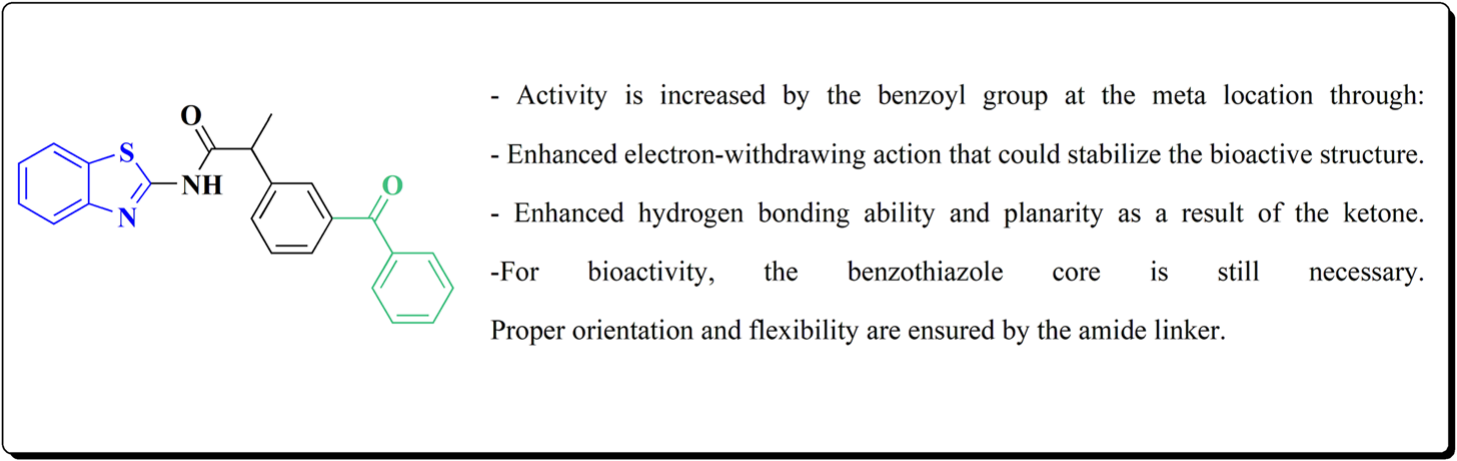
Structure–activity relationship of compound 150.

Several benzothiazoles have utility as anti-inflammatory agents.^156,157^ It was also reported that 2-aminobenzothiazole was combined with a variety of profens to synthesize a number of novel compounds 150 (Scheme 20, Fig. 10). When their biological activities were examined *in vitro*, they had strong antioxidant and anti-inflammatory properties that were on scale with those of common reference compounds. The findings demonstrate the compounds' potent affinity for HSA and encouraging biological activity. The experimental findings and *in silico* calculations support the new hybrid compound between 2-ABT and ketoprofen 150, which shows great promise. With an IC_50_ of 60.24 μg mL^−1^, compound 150 has the highest hydrogen peroxide scavenging activity of all the compounds examined. Additionally, compound 150 has better anti-inflammatory action than the conventional ibuprofen (76.05 μg mL^−1^), with an IC_50_ of 54.64 μg mL^−1^.^158^

## 8. Synthetic strategies for novel antitumor benzothiazoles

### 8.1. Benzothiazoles linked with heterocyclic compounds

#### 8.1.1. Deaza-pyrimidine–benzothiazole based analogues

**Scheme 21:**
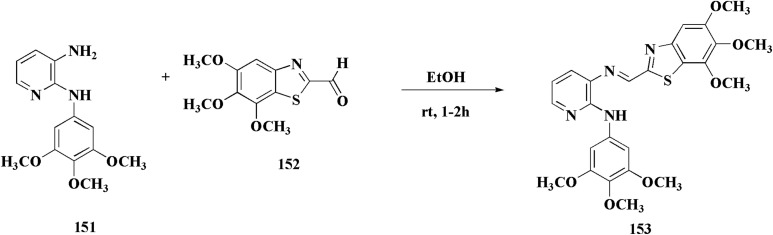
Synthesis of 2-anilinopyridinyl-benzothiazoles.

Compound 151 was reacted with compound 152 to generate compounds 153 (Scheme 21). The anticancer potential of the compounds was evaluated. The compound 153 displayed high antiproliferative potency (GI_50_ value of 3.8 μM) against the cell line DU145.^159^

**Fig. 11:**
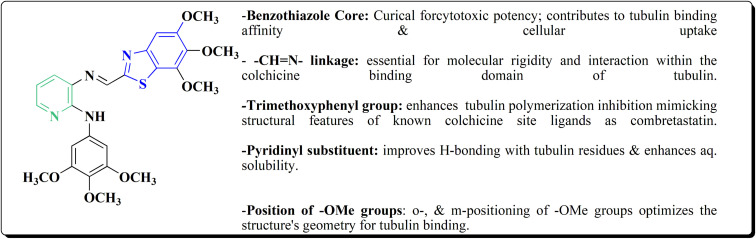
Structure–activity relationship of compound 153.

In this study, a series of 2-anilinopyridinyl-benzothiazole Schiff bases were rationally designed by molecular modeling, and key compounds—particularly 153, featuring a trimethoxy substituent on the benzothiazole ring—exhibited superior binding energies at the tubulin colchicine binding site (even outperforming the reference ligand E7010). These derivatives disrupted mitotic spindle assembly by inhibiting tubulin polymerization, causing a cell-cycle arrest at the G_2_/M phase. Biologically, compound 153 (Fig. 11) induced apoptosis through mitochondrial pathways (*e.g.* loss of mitochondrial membrane potential, caspase-3 activation, and Annexin V binding), consistent with microtubule destabilization leading to programmed cell death.

**Scheme 22:**
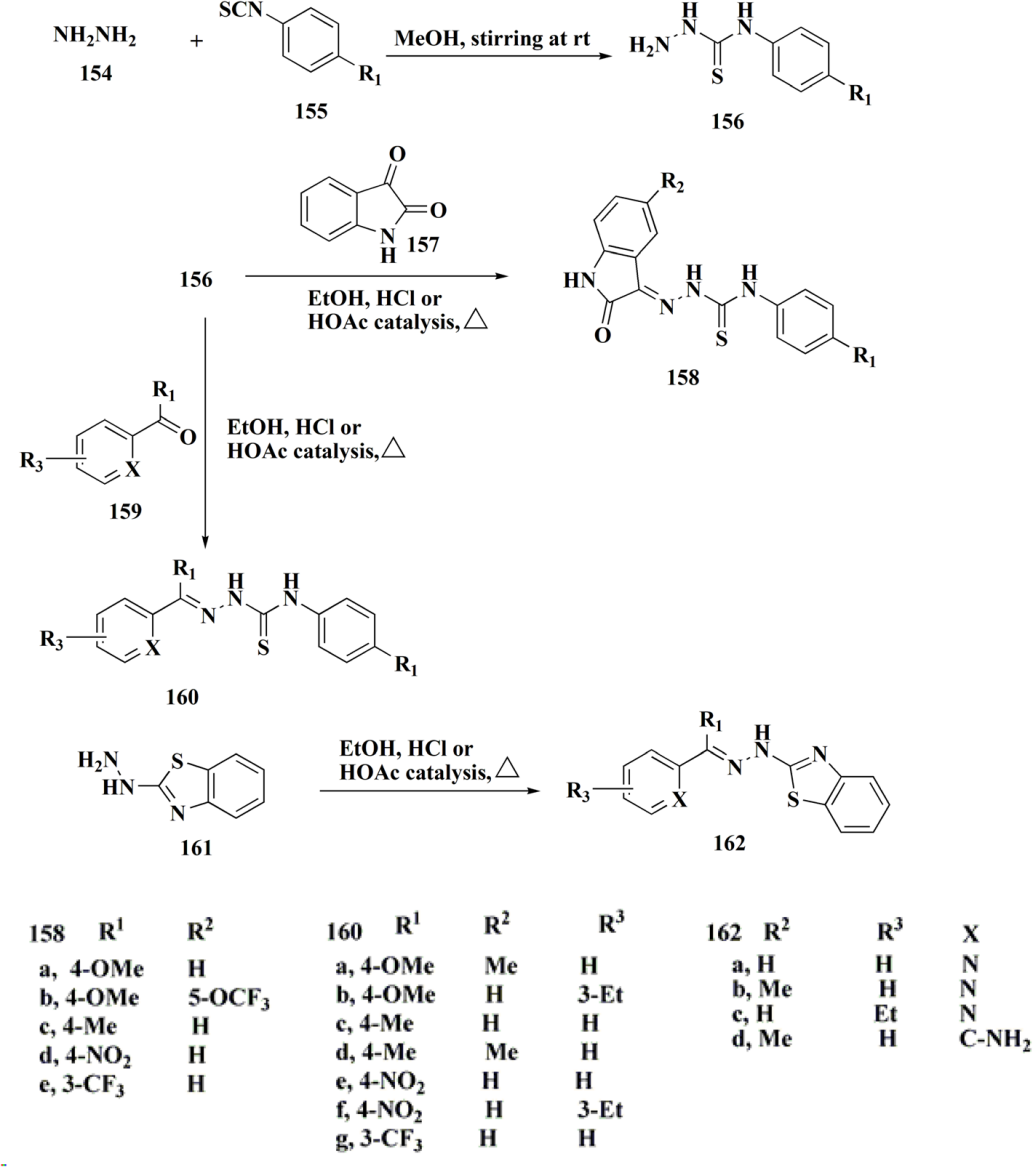
Synthesis of hydrazinobenzothiazoles.

**Fig. 12:**
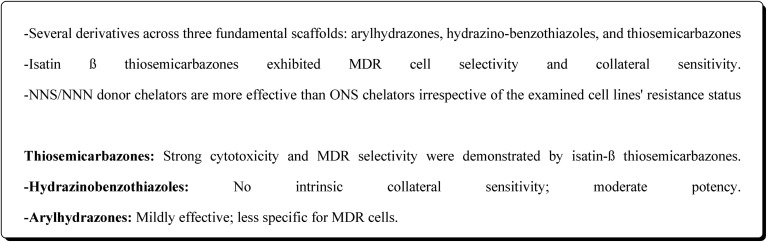
Structure–activity relationship of compounds 158–162.

In another approach, the thiosemicarbazones were generated through an acid catalyzed Schiff base condensation of the thiosemicarbazide 156 with the corresponding ketone. Compound 156 were synthesized from the isothiocyanates upon reaction with hydrazine. The picolinylidene, acetaniline and salicylidene hydrazinobenzothiazoles 162 were accessed by reacting 2-hydrazinobenzothiazole with the keto components under acid catalyzed conditions (Scheme 22; Fig. 12).^160^

**Scheme 23:**
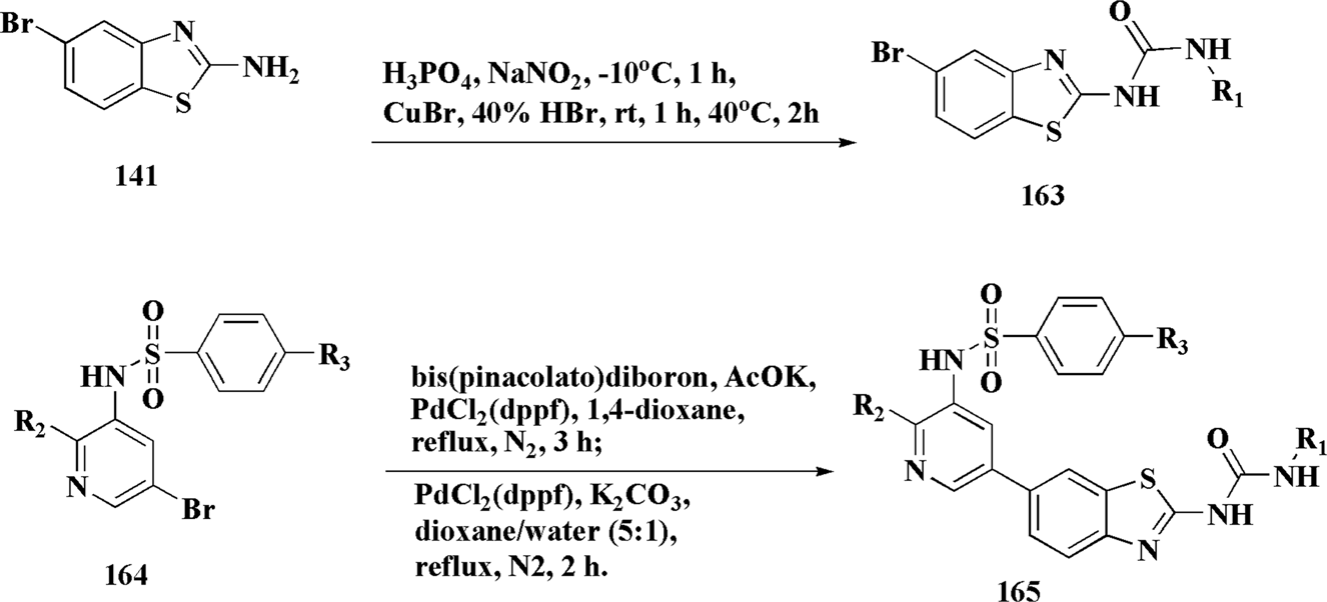
Synthesis of substituted sulfonylaminopyridin-(5-yl)benzo[*d*]thiazol-2-yl)urea.

**Scheme 24:**
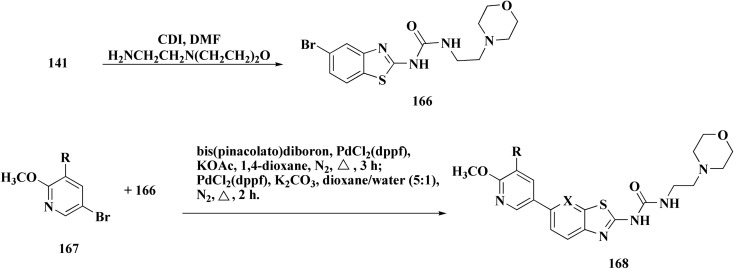
Synthesis of substituted (benzo[*d*]thiazol-2-yl)urea.

2-Amino-6-bromobenzothiazole was allowed to react with alkylamine and CDI to afford compounds 163. The intermediate 164 was reacted with intermediate 163 and bis(pinacolato)diboron to generate compounds 165 (Schemes 23 & 24).^161,162^

**Fig. 13:**
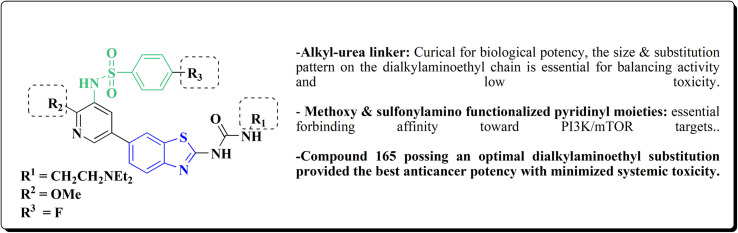
Structure–activity relationship of compound 165.

The anti-proliferative potencies of the compounds were tested *in vitro* against MCF-7, HCT116, A549 and U87 MG cell lines. The compounds with high anti-proliferative potency were examined for their oral toxicity and their inhibitory effect against mTORC1and PI3Ks. Compound 165 (Fig. 13) can efficiently inhibit tumor growth in a study using a mice S180 homograft model. These results propose that this compound can act as potent anticancer agents and PI3K inhibitors.^162^

#### 8.1.2. Pyrazole–benzothiazole based analogues

**Scheme 25:**
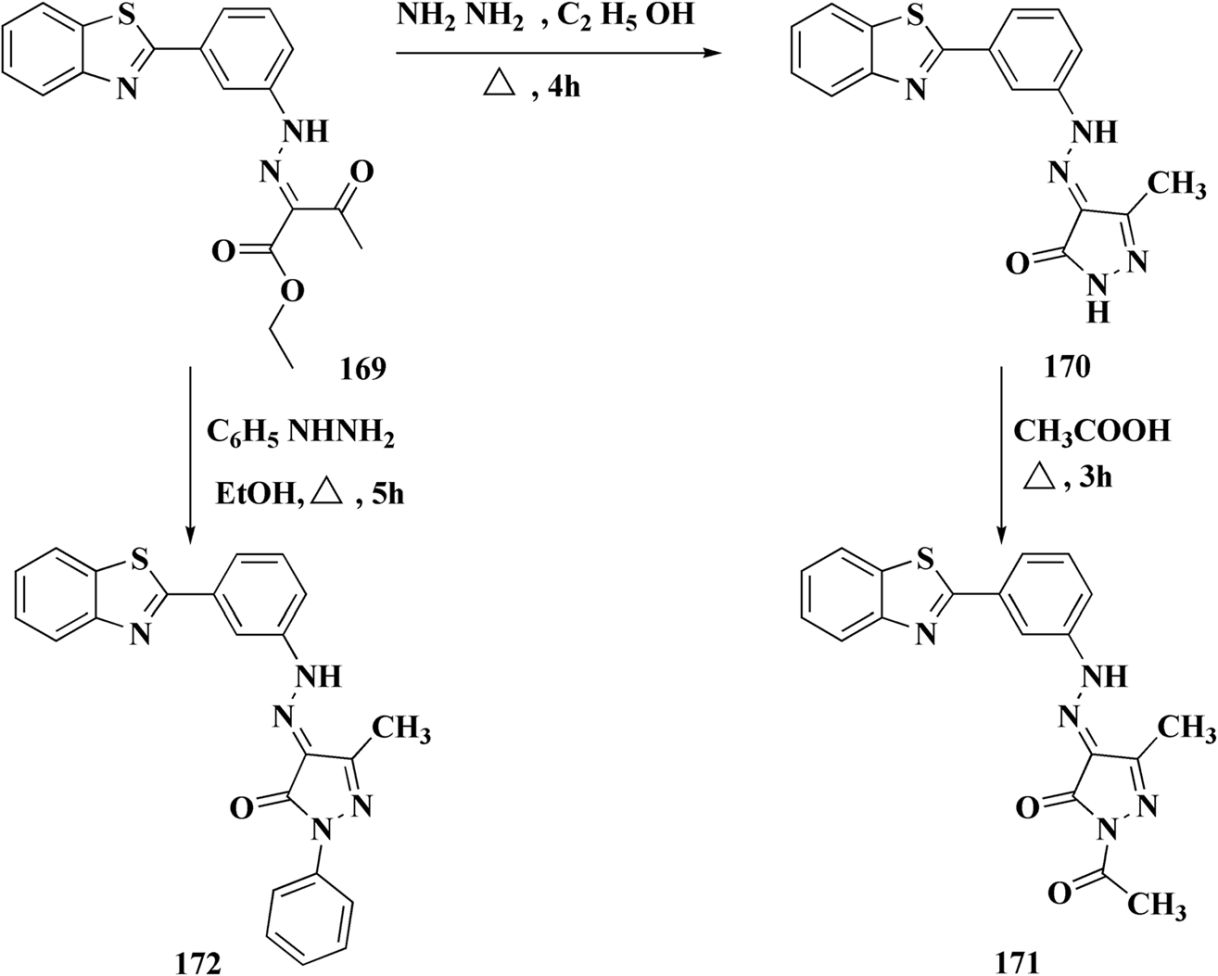
Synthesis of pyrazole–benzothiazole conjugates.

Pyrazoles have various biological potencies,^163–166^ its conjugations with benzothiazoles enhance their activities.^167^ Novel benzothiazole substituted pyrazole derivatives were synthesized. Compound 169 was furnished as accomplished in Scheme 25. Then the synthesis of the pyrazolone derivatives 171 was generated by refluxing compound 169 with hydrazine hydrate. Acetylation of compounds 170 yielded the substituted acetyl pyrazolone 171. Additionally, reacting compound 169 with phenylhydrazine afforded the phenylpyrazolone 172.^167^

**Fig. 14:**
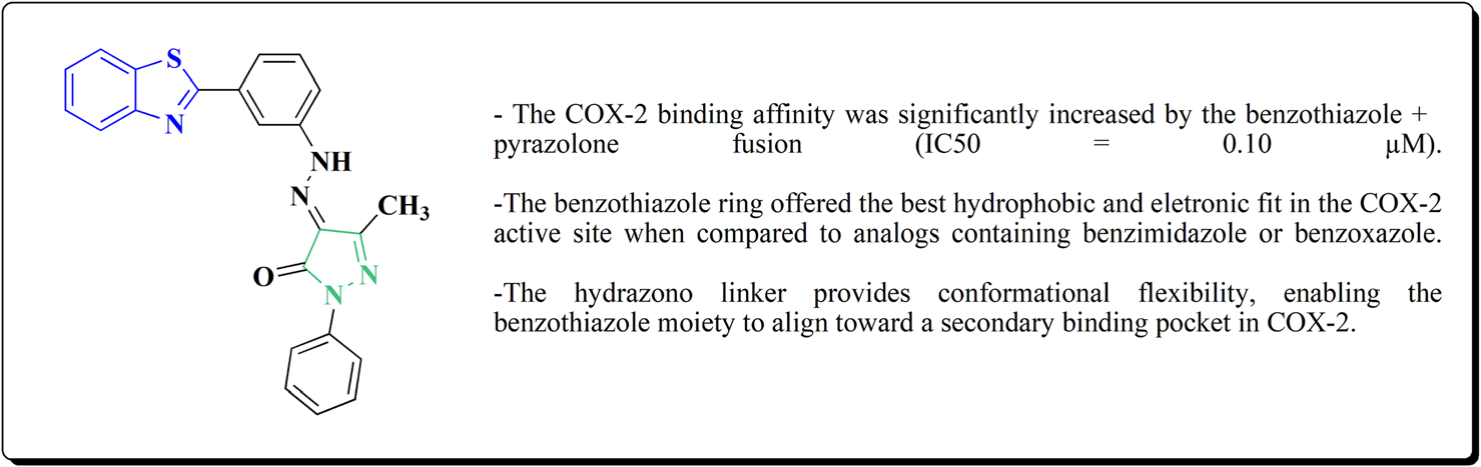
Structure–activity relationship of compound 172.

The compounds were examined for their anti-proliferative potency against A549 and MCF-7 cell lines. The obtained findings revealed that the benzothiazolopyrazoloylpyrazolone derivative 172 was the most active COX-2 inhibitor as comparable to celecoxib (Fig. 14).^167^

**Scheme 26:**
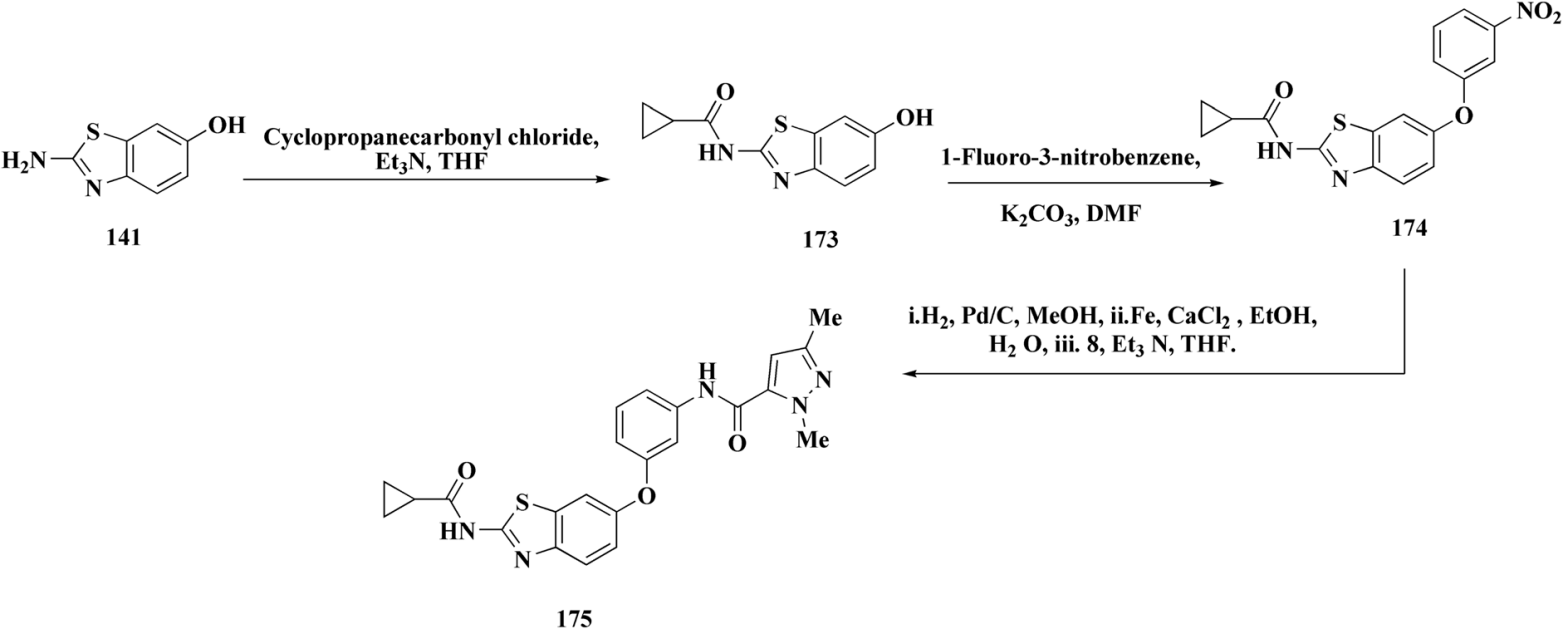
Synthesis of substituted dimethyl-1*H*-pyrazole-5-carboxamide.

Furthermore, the synthesis of the 1,3-benzothiazole derivative 175 was outlined in Scheme 26. Acylation of compound 30 with the cyclopropanecarboxylic acid chloride generated 173, which was reacted with the substituted nitrobenzene to afford compound 174. Reduction of the latter compound 174 and further acylation with acid chloride yielded compound 175. The compounds indicated strong inhibitory activity against VEGFR2 kinase.^168^

#### 8.1.3. Imidazole–benzothiazole based analogues

**Scheme 27:**
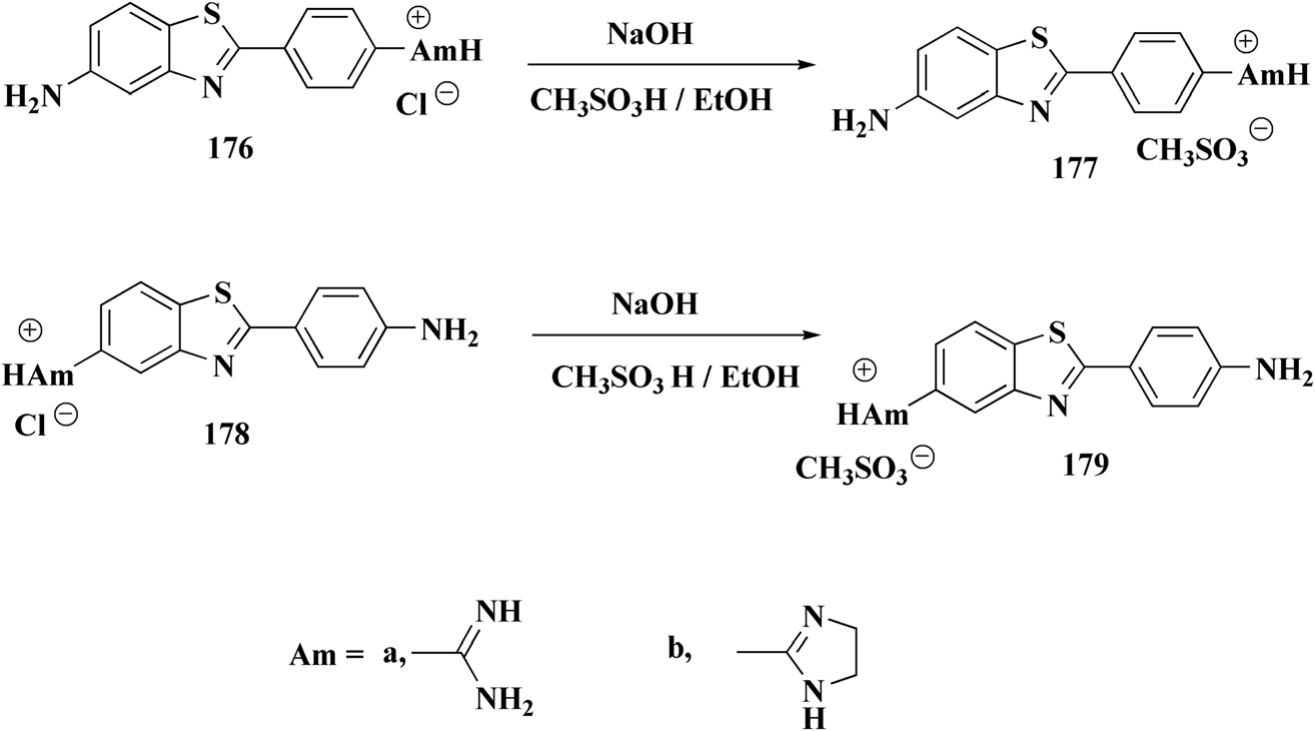
Synthesis of benzothazole mesylate salts 177b–179b.

Hydrochloride salts 176 were transformed to mesylate salts 177, as accomplished in Scheme 27. Mesylate salts 177 were synthesized through the corresponding free bases. The free bases as intermediates were isolated after the addition of sodium hydroxide solution, and transformed into mesylates *via* treating their ethanolic solutions with mesylic acid. The amidine scafold was then protonated and the salts 177b–179b were accessed as monocationic mesylates.

The *in vitro* investigations of benzothiazoles revealed antiproliferative potency on a panel of human cancer cell lines.^169^

**Fig. 15:**
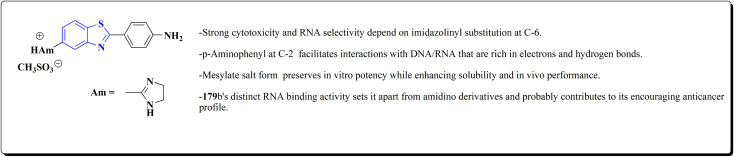
Structure–activity relationship of compound 179.

Without compromising *in vitro* potency, conversion from hydrochloride to mesylate salt significantly increases water solubility and bioavailability. The mesylate derivative of 179b (Fig. 15) is better for *in vivo* application because it has substantial cytotoxic effects on tumor cells while having little acute oral toxicity.

Mode of DNA/RNA binding: -dual binding is indicated in 179b: ds-RNA intercalation, binding of minor grooves to AT-rich DNA, unique among the derivatives under study in terms of distinct single-stranded RNA (ss-RNA) binding.

Its cytotoxic selectivity is probably influenced by this selective binding profile.

Correlation of biological activity: using a variety of cancer cell lines, 179b consistently shown cytotoxic action. Its capacity to bind RNA more effectively than amidino analogs coincides with its action, indicating a molecular connection between RNA recognition and antitumor efficacy.^169^

**Scheme 28:**
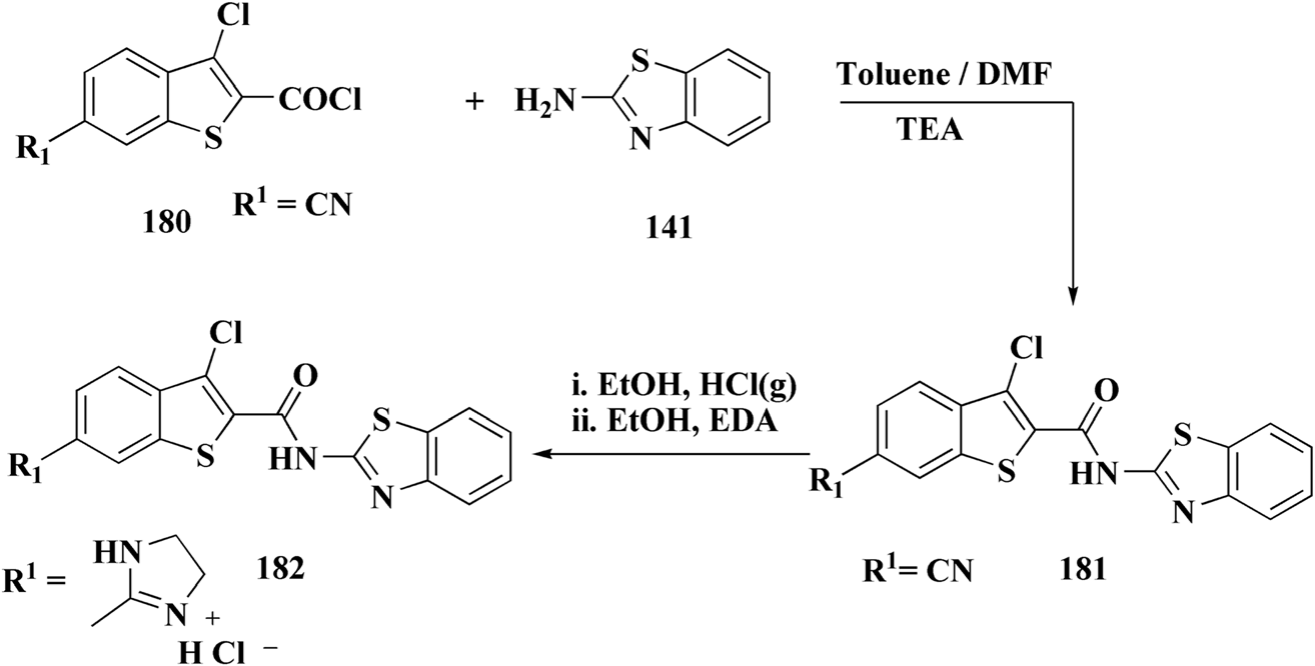
Synthesis of benzothiazolebenzo[*b*]thieno-2-carboxamides.

In parallel, the benzothiazolebenzo[*b*]thieno-2-carboxamides 181 were generated. Compound 180 were reacted with 2-aminobenzothiazoles to afford the corresponding carboxamides 181. Compounds 181 underwent an acidic Pinner reaction to yield the imidazolinyl substituted derivatives 182. Monosubstituted carboxamides bearing 2imidazolinyl group on benzothiazole nuclei were synthesized through the condensation of compound 180 with 2-amino-6-(2imidazolinyl)benzothiazole 141 (Scheme 28).

**Fig. 16:**
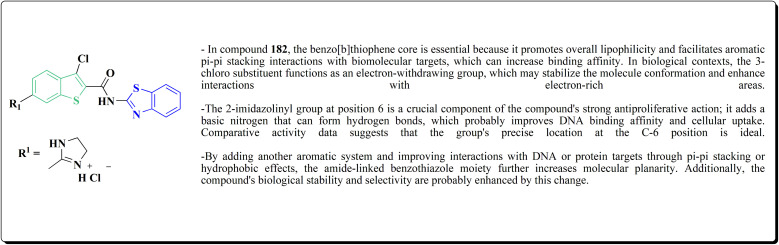
Structure–activity relationship of compound 182.

The antiproliferative activities were estimated *in vitro* against human cancer cell lines. The effective potency against HeLa cells was showed for compound 182 (IC_50_ = 1.16 μM) (Fig. 16).^170^

The bioactive benzothiazole and 1,2,3-triazole moieties were integrated into a single molecular framework for the investigation of their anticancer potential using an effective Cu(i)-catalyzed alkyne–azide cycloaddition (CuAAC) technique.

**Scheme 29:**
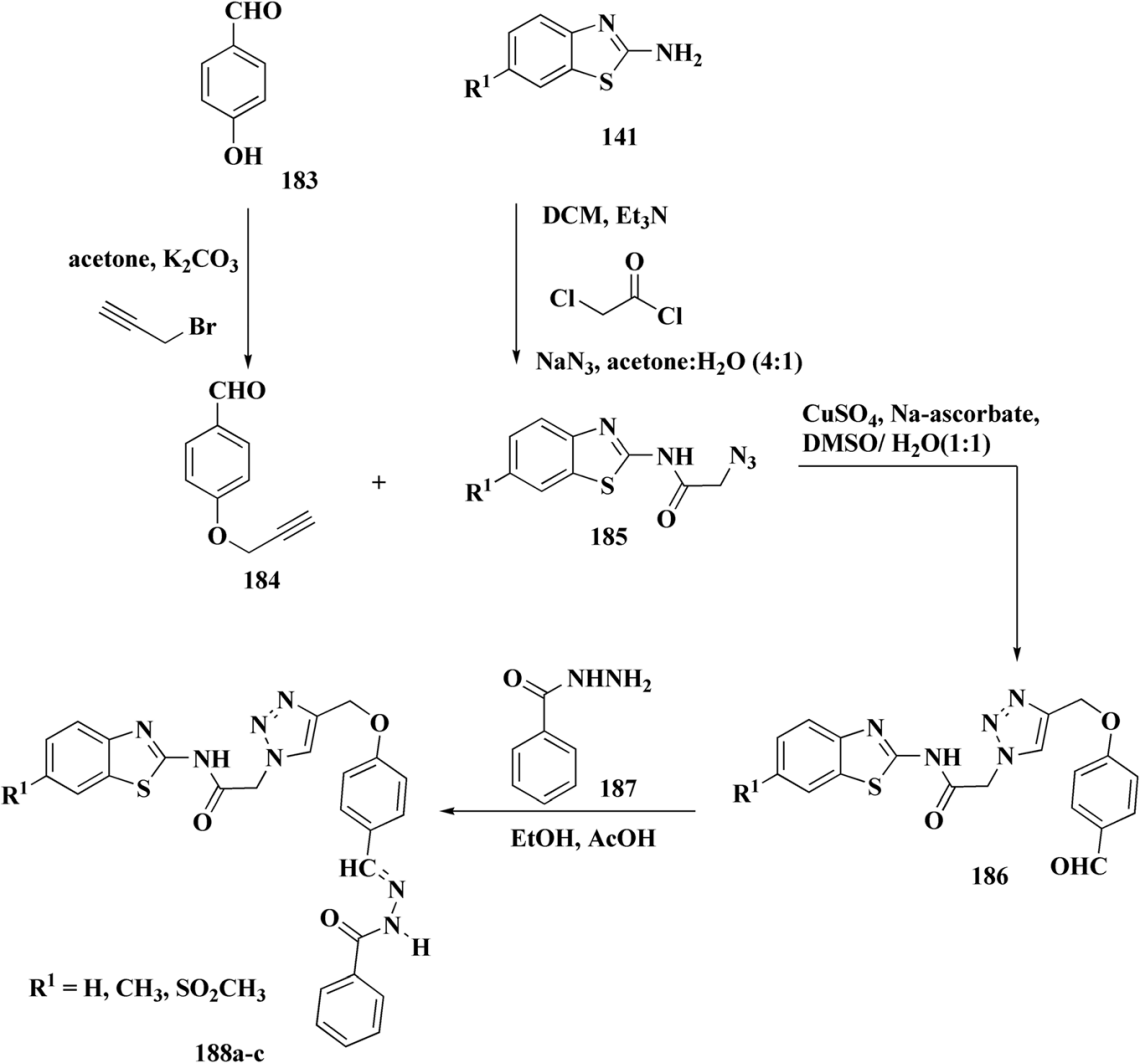
Synthesis of thiosemicarbazone based benzothiazole-1,2,3-triazole derivatives 188a–c.

Therefore, by 1,3-dipolar cycloaddition of different substituted and unsubstituted benzothiazole azides 185 with corresponding *O*-propargylated benzylidene derivatives, a novel series of 1,2,3-triazole–benzothiazole conjugates 188 with hydrazone or thiosemicarbazone linkages was successfully designed and synthesized (Scheme 29).

Compounds 188a–c demonstrated high anticancer activity, especially against the T47D breast cancer cell line, while maintaining acceptable safety profiles in normal cells, according to a biological study of these compounds against three human cancer cell lines. Additionally, compounds 188a and 188b showed remarkable anti-proliferative potential by dramatically inhibiting the two-dimensional migration of lung cancer cells in a concentration-dependent manner. Interestingly, the IC_50_ values of compounds 188a, 188b, and 188c were 0.69, 1.16, and 4.82 μM, respectively.

These results were corroborated by molecular docking experiments, which demonstrated that 188a and 188b have substantial binding affinities to the EGFR active site. These findings collectively imply that compounds 188a and 188b are excellent candidates for the synthesis of chemotherapeutic drugs that target EGFR.^171^

**Fig. 17:**
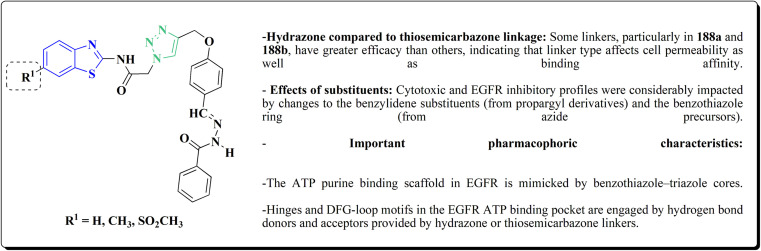
Structure–activity relationship of compound 188.

Against T47 D cells, compounds 188a–188c (Fig. 17) demonstrated the strongest cytotoxic effects: IC_50_ values: ≈0.69 μM for 188a, 1.16 μM for 188b, and 4.82 μM for 188c, at matched concentration, it demonstrated >96% inhibition in a favorable comparison to erlotinib (IC_50_ = 1.3 μM). Safety profiling on normal (non-cancerous) cells found IC_50_ values >500 μM, indicating low toxicity and a high therapeutic window. The mechanism of 188a and 188b as EGFR inhibitors was supported by molecular docking, which revealed that they have substantial binding affinities for the EGFR active site.

#### 8.1.4. Tetrazole–benzothiazole based analogues

**Scheme 30:**
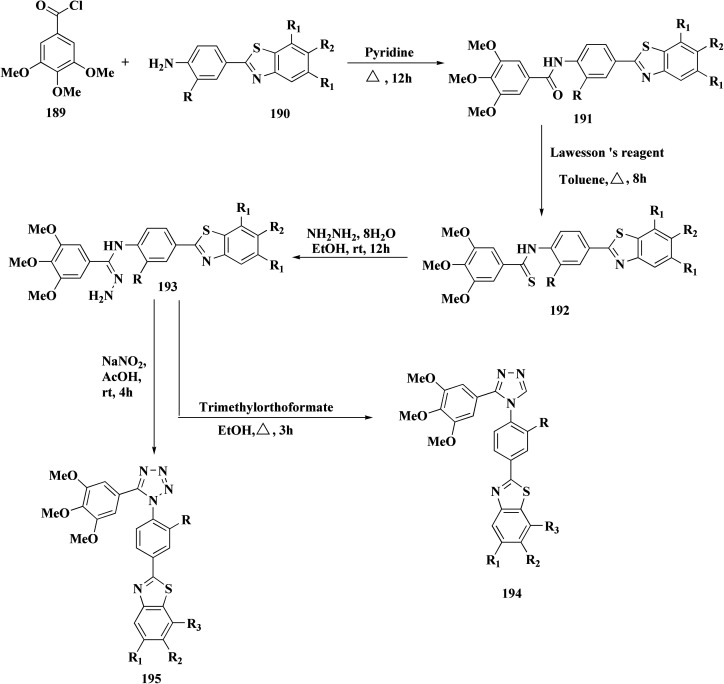
Synthesis of amidrazones 193 & combretastatin-benzothiazole analogs.

Colchicine site binding tubulin inhibitors were prepared as outlined in Scheme 30. Synthesis of the tetrazoles 194 and 195. The 2-(4-aminophenyl) benzothiazoles 190 were reacted with the benzoyl chloride 189 to afford the substituted benzothiazole amides 191 which upon further treatment with Lawesson's reagent affords thioamides 192. The reaction of thioamides with hydrazine hydrate yields amidrazones 193. The intramolecular cyclization was carried out using trimethylorthoformate in the presence of catalytic amount of sulphuric acid giving the targeted cis restricted 1,2,4-triazoles 194, whereas 1,2,3,4-tetrazoles 195 were obtained as shown in Scheme 30.

**Fig. 18:**
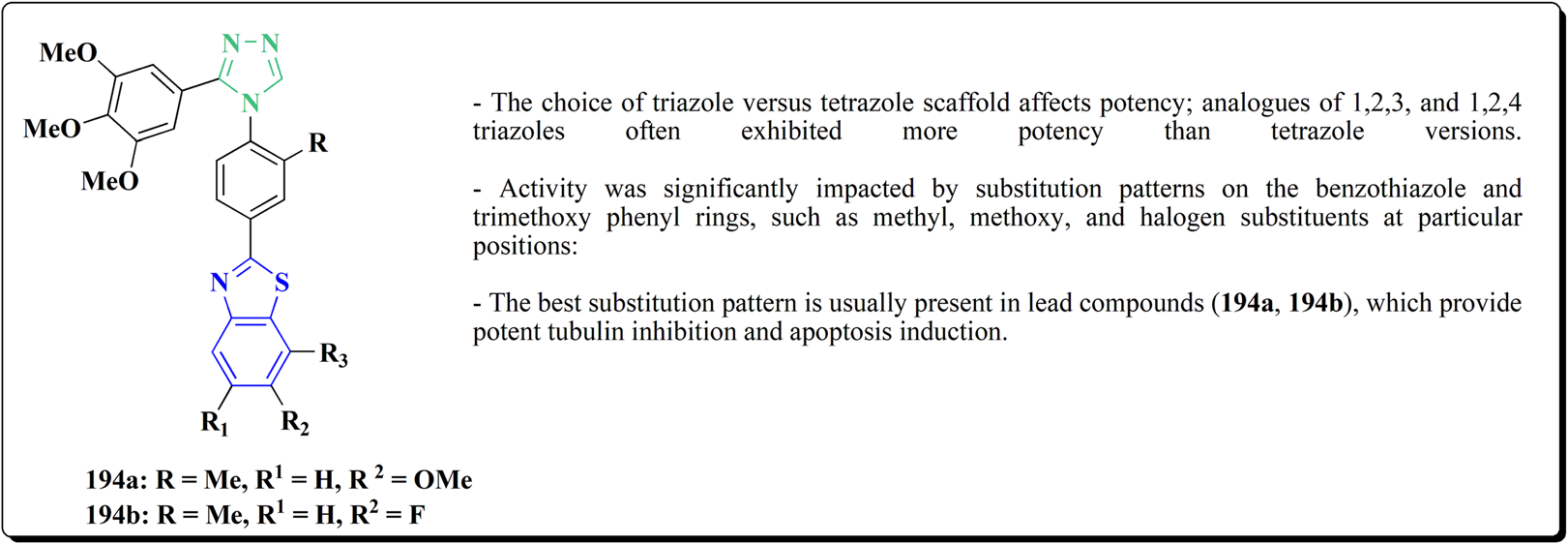
Structure–activity relationship of compound 194.

These compounds were tested for their antiproliferative potency against cancer cell lines. The most active compounds 194a,b (Fig. 18) indicated an antiproliferative effect as compared to that of Combretastatin A-4 (CA-4).^172^

They induced cell-cycle arrest (mitotic arrest) in the G-/M phase. Immunocytochemistry and tubulin polymerization tests validate the disruption of microtubule dynamics. Western blot experiments showed increased tubulin in the soluble fraction, which is consistent with depolymerization. Molecular docking and colchicine-competitive binding tests confirm that they bind at the colchicine site in a manner comparable to Combretastatin A-4 (CA 4).

After mitotic arrest, these compounds cause apoptotic cell death, as demonstrated by these compounds induce apoptotic cell death following mitotic arrest, confirmed by: hoechst nuclear staining, mitochondrial membrane potential loss, Annexin-V-FITC positivity, activation of caspase-3, ROS generation.

#### 8.1.5. Thiazole–benzothiazole based analogues

**Scheme 31:**
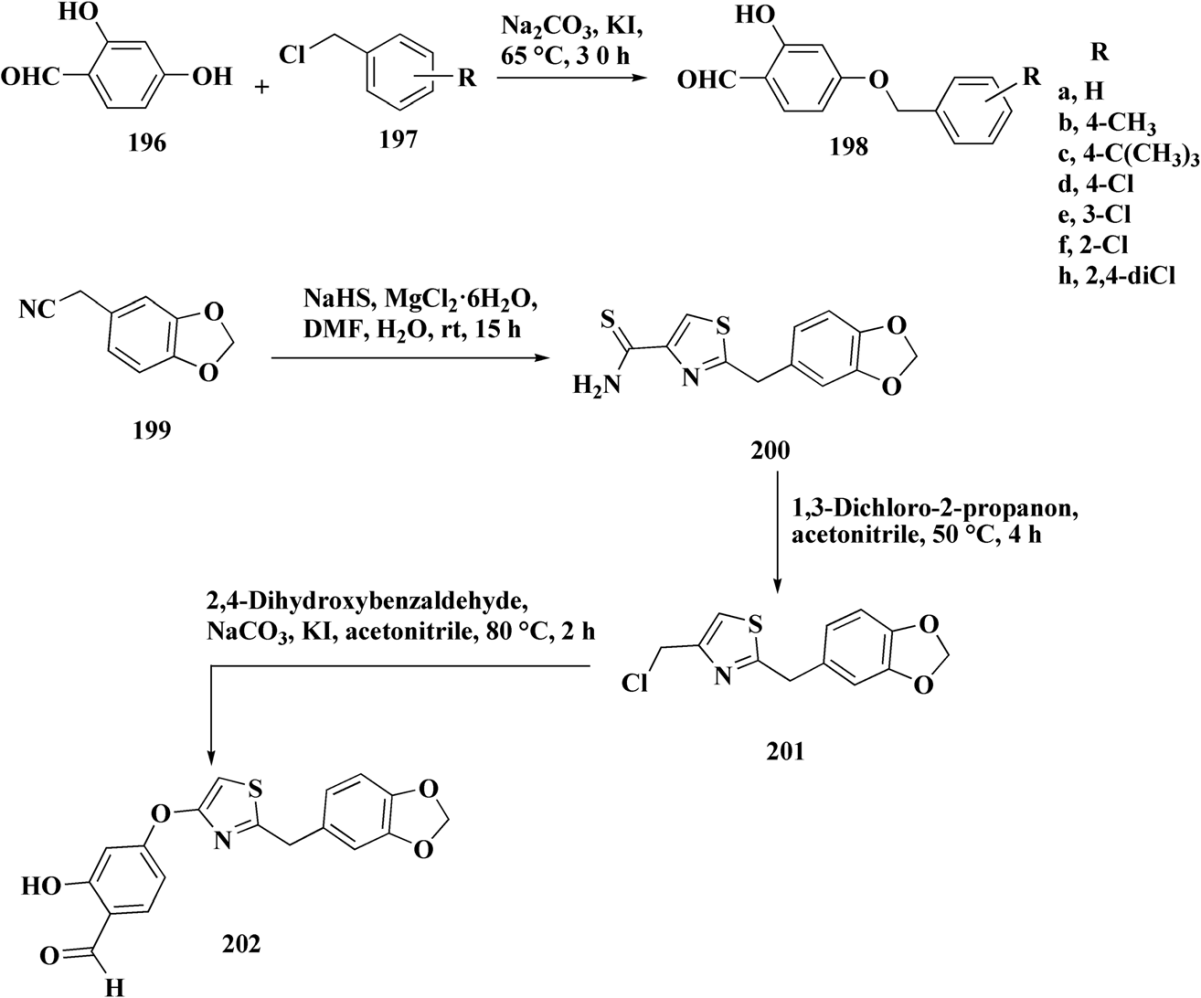
Synthesis substituted thiazole derivative 202.

**Scheme 32:**
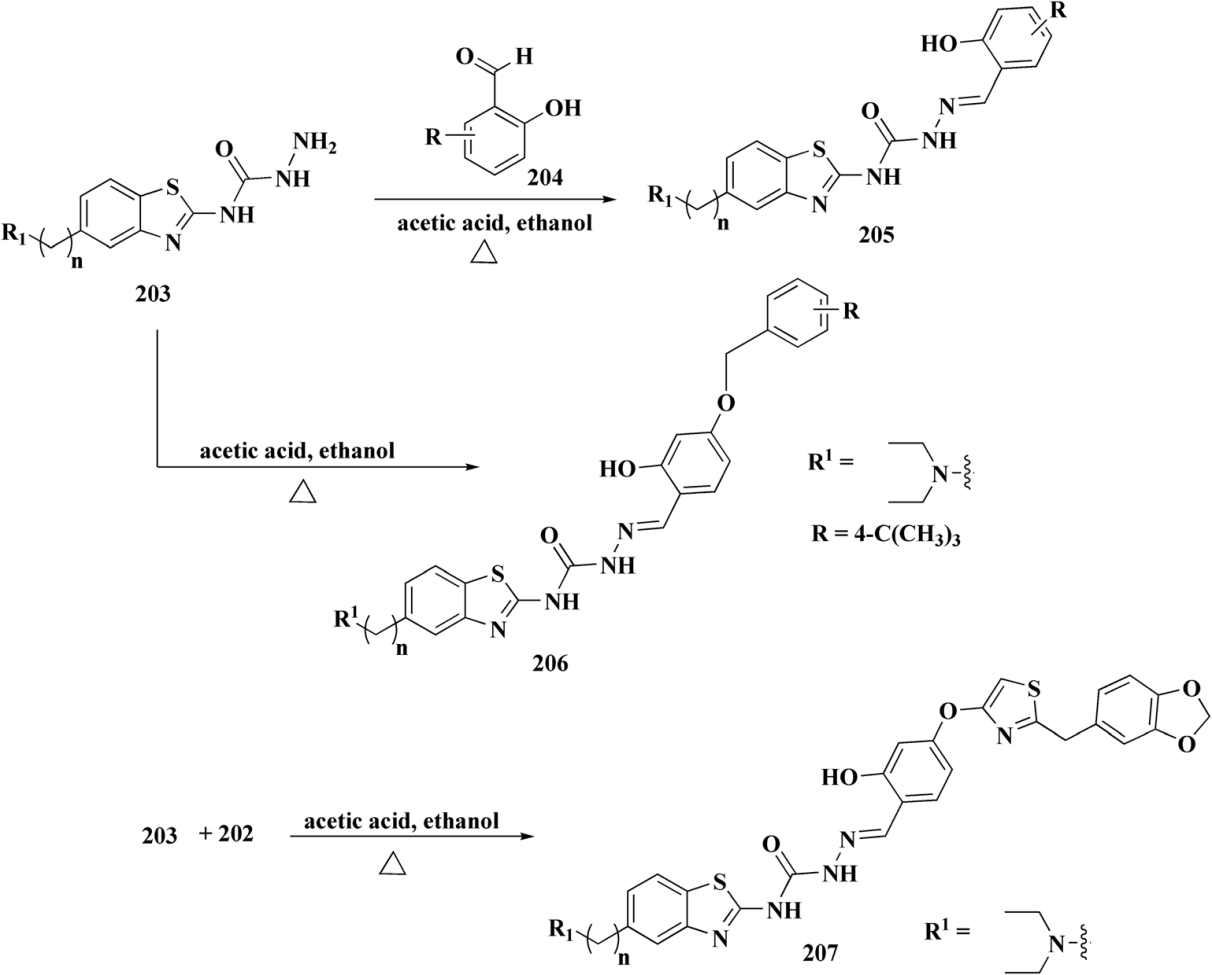
Synthesis of substituted hydrazinecarboxamides.

The benzyloxy 2-hydroxybenzaldehydes 198 were furnished through the reaction of 2,4-dihydroxy benzaldehyde with substituted benzylchloride. 1,3-Benzodioxole-5-acetonitrile was allowed to react with sodium sulfide to access compound 200, which was then reacted with 1,3-dichloroacetone to give compound 201.^173^ Compound 202 was synthesized through the reaction of 4-hydroxysalicylaldehyde with compound 201. Its worthy to note that the salicylaldehyde analogs have anticancer potencies.^174,175^ The desired compounds 205, 206 and 207 were furnished through the reaction of compound 203 with 2-hydroxy aromatic aldehydes (Schemes 31 and 32).^173^

**Fig. 19:**
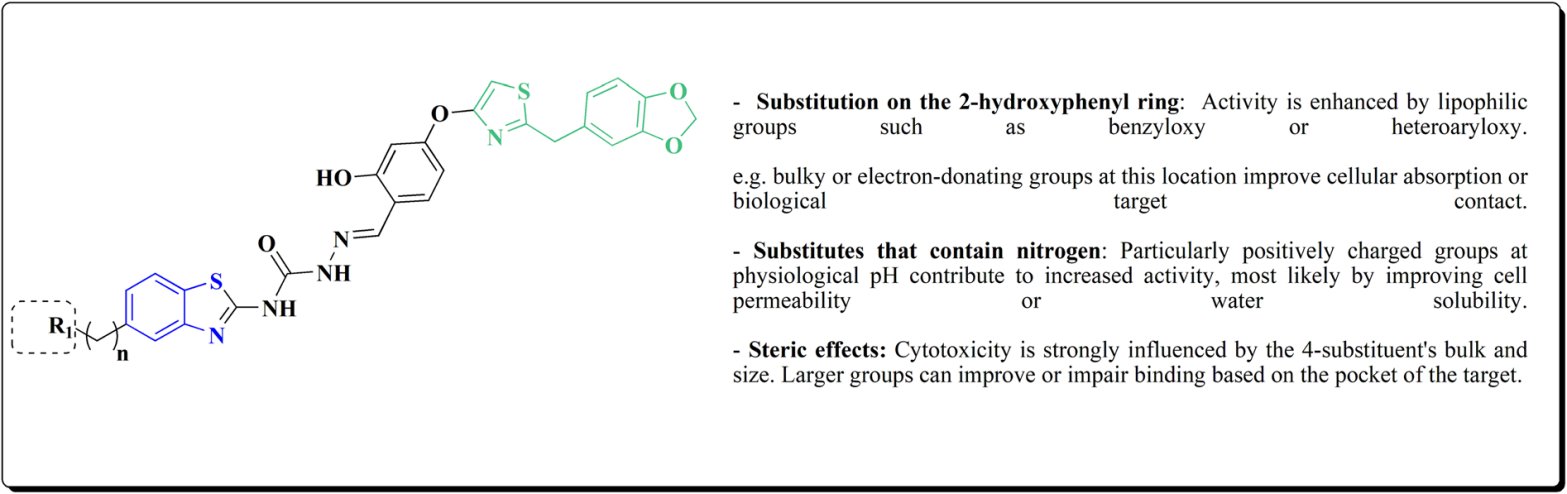
Structure–activity relationship of compound 207.

The cytotoxic activities were evaluated and screened *in vitro* against cancer cell lines (SK-N-SH, NCI-H226, HT29, MDA-MB-231, and MKN45). Compounds 206 (procaspase-3 EC_50_ = 1.42 μM) and 207 (procaspase-3 EC_50_ = 0.25 μM) showed anti-tumor potency with IC_50_ values in the rang of 0.14 μM to 0.98 μM on a panel of cancer cell lines (Fig. 19).^173^

These compounds cause increased apoptosis in cancer cells, which explains the observed cytotoxicity. Procaspase-3, a pro-enzyme version of caspase-3, is a crucial apoptotic executioner. Although the novel compounds have noticeably more potency, this mechanism is identical to that of PAC-1.

Strong procaspase-3 activating properties are provided by the study's powerful and structurally unique anticancer agents. SAR analysis provides precise guidelines for further optimization, emphasizing steric tuning, charge distribution, and lipophilicity.^173^

**Scheme 33:**
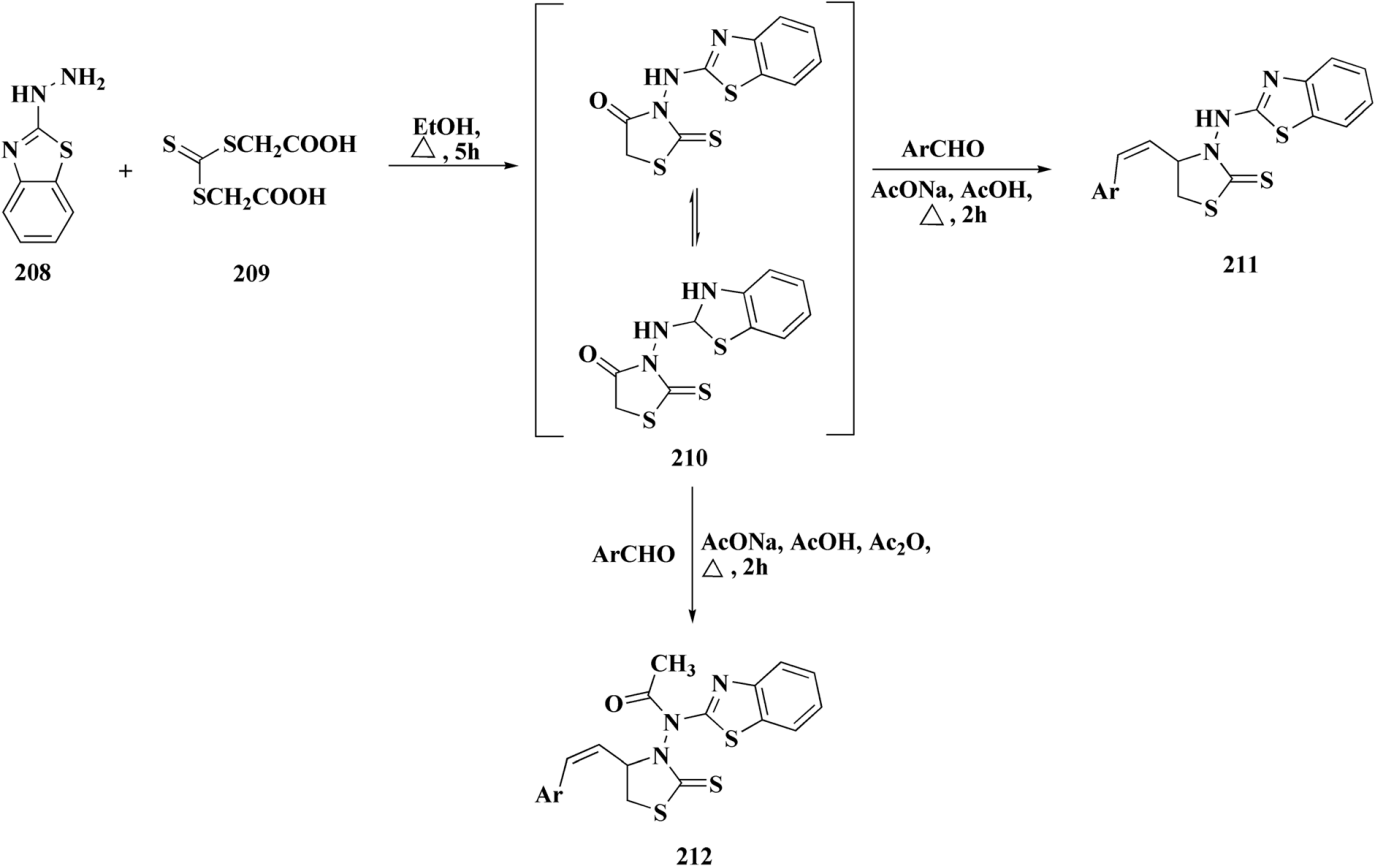
Synthesis of benzothiazole substituted 4-thiazolidinones derivatives.

In continuation, the synthesis of benzothiazole substituted 4-thiazolidinones derivatives were accomplished in Scheme 33. The thiazolidone 210 was synthesized from 2-hydrazino-1,3-benzothiazole and trithiocarbonyldiglycolic acid in ethyl alchol under reflux. Compound 210 was allowed to react with the aromatic aldehydes to yield 5-arylidene derivatives 211*via* a knoevenagel condensation's reaction. The acetylation of exocyclic nitrogen was observed 212 following acetanhydride addition to reactive mixture.

**Fig. 20:**
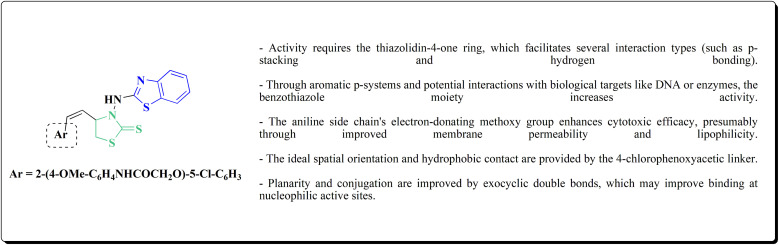
Structure–activity relationship of compound 211.

*In vitro* anti-cancer potency of the compounds was evaluated. The compounds have showed the anticancer activity on cancers cell lines. Among examined compounds, the acetamide 211 was the most potent candidate with average logTGI and logGI50 values −4.45 and −5.38 (Fig. 20).^176^

The cell cycle arrest and apoptosis induction are likely the mechanism, albeit precise enzyme targets have not been established. The possible DNA intercalation or interaction with cysteine-rich enzymatic pockets (such as kinases or reductases) is suggested by structural characteristics.

Strong cytotoxicity was demonstrated by compound 211 against a panel of human carcinoma cell lines.

#### 8.1.6. Indole–benzothiazole based analogues

By combining two active scaffolds, a novel series of benzothiazole–indole hybrid compounds was designed to target important cancer pathways: • benzothiazole, which interacts with kinase domains, tubulin, and DNA. • Indole, a heterocycle compound frequently found in antiproliferative medications and bioactive natural compounds.

**Scheme 34:**
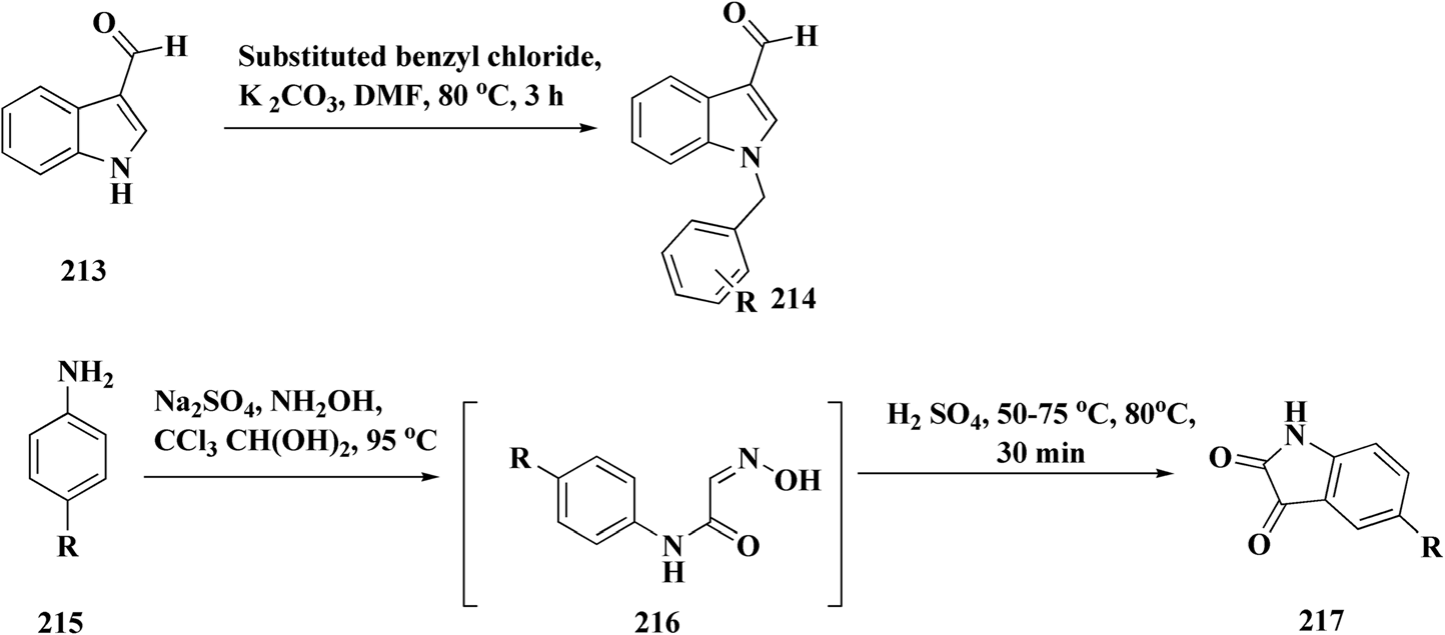
Synthesis of substituted indoles and isatins.

Compounds 214 were prepared *via* nucleophilic substitution of different α-chlorotoluene with 3-formylindole 213. The substituted isatins 217 were synthesized through condensation of phenylamine with trichloroacetaldehyde followed by cyclization in dihydrogen sulfate (Scheme 34).

**Scheme 35:**
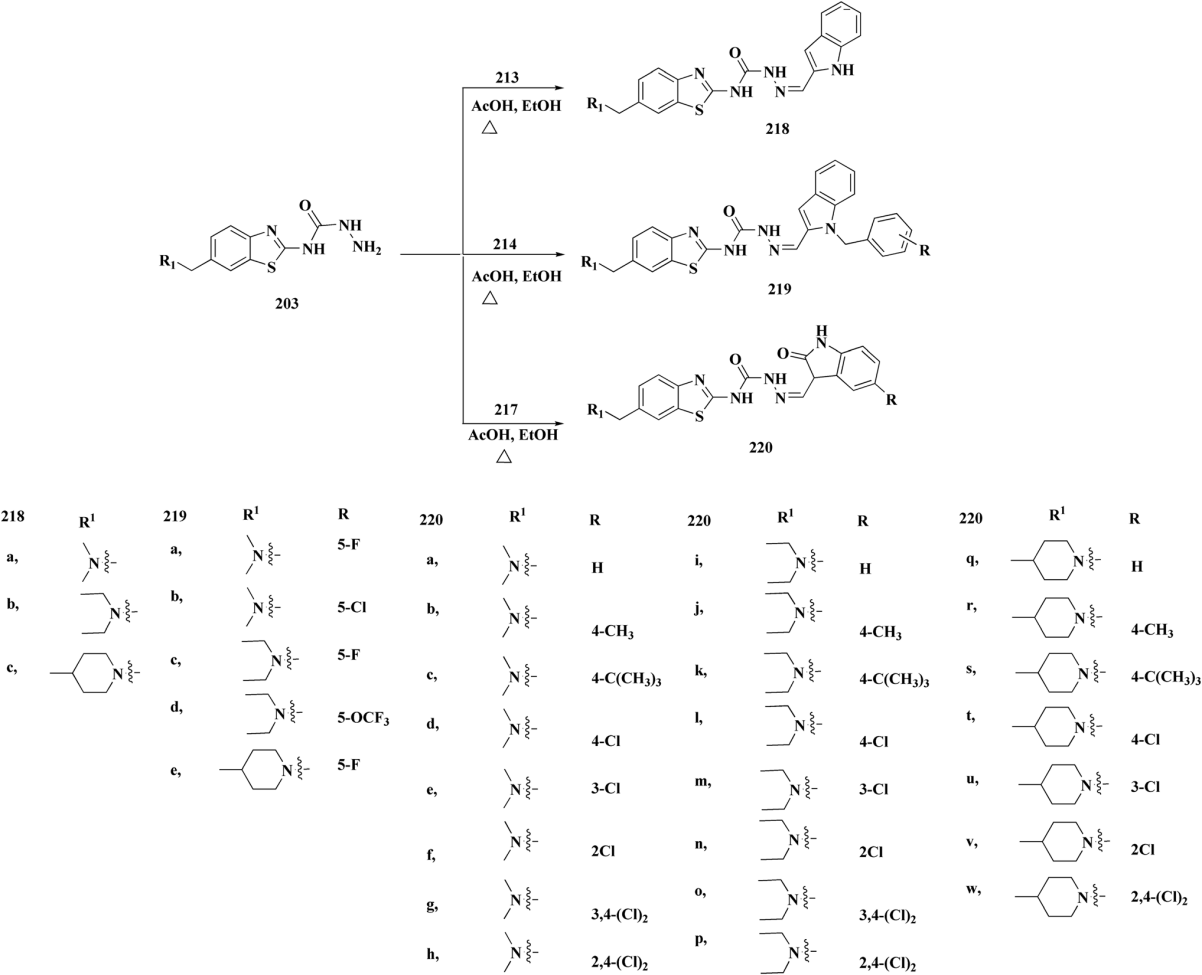
Synthesis of benzothiazole-isatin & benothiazole–indole conjugates.

The desired compounds 218, 219 and 220 were obtained through the reaction of 203 with indole-3-carbaldehyde analogs 213 & 214 or 5-substituted isatins 217 (Scheme 35). The compounds were then screened for *in vitro* antitumor potency on a panel of cancer cell lines. Compounds 219 displayed good selectivity against HT29 cancer cell line. Compound 219 showed high antitumor potency against H460, A549, HT29, and MDA-MB-231.^177^

**Fig. 21:**
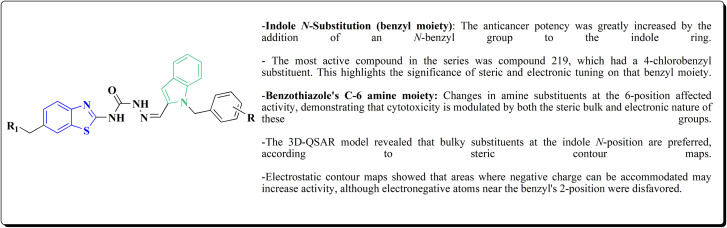
Structure–activity relationship of compound 219.

Strong anticancer potency was demonstrated by the newly synthesized benzothiazole–indole hybrids, especially compound 219 (Fig. 21), which activated caspase and disrupted tubulin to cause mitotic arrest and apoptosis. A prospective lead chemical for more preclinical research and mechanistic investigation is compound 219.^177^

**Scheme 36:**
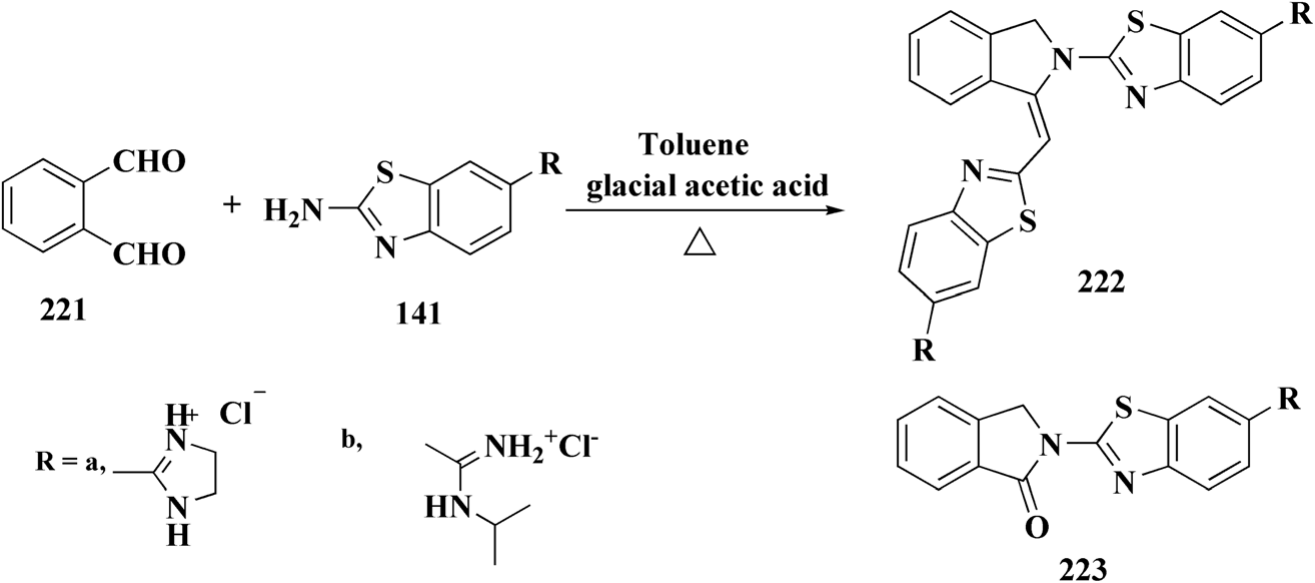
Synthesis of iminoindolines and isoindolin-1-ones-benzothiazoles conjugates.

**Fig. 22:**
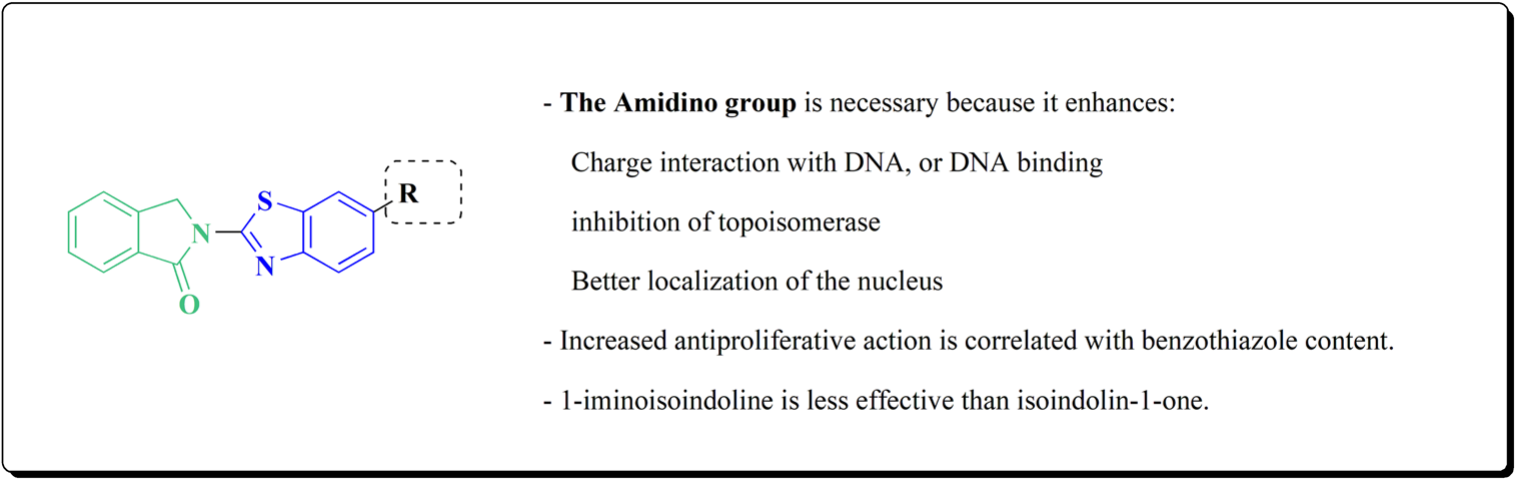
Structure–activity relationship of compound 223.

Following this line of the research of conjugating the indoles with benothiaoles, the isoindolines substituted with amidino- and cyano-benzothiazoles were prepared as novel anticancer agents. Amidino substituted benzo[*d*]thiazol-2-amine 141a–b was synthesized from 6-cyano-1,3-benzothiazol-2-amine as outlined in Scheme 27. The reaction of compounds 141 with *o*-phthalaldehyde 221 afforded the 1-iminoindolines 222 and isoindolin-1-ones 223 (Scheme 36, Fig. 22).^178^

**Scheme 37:**
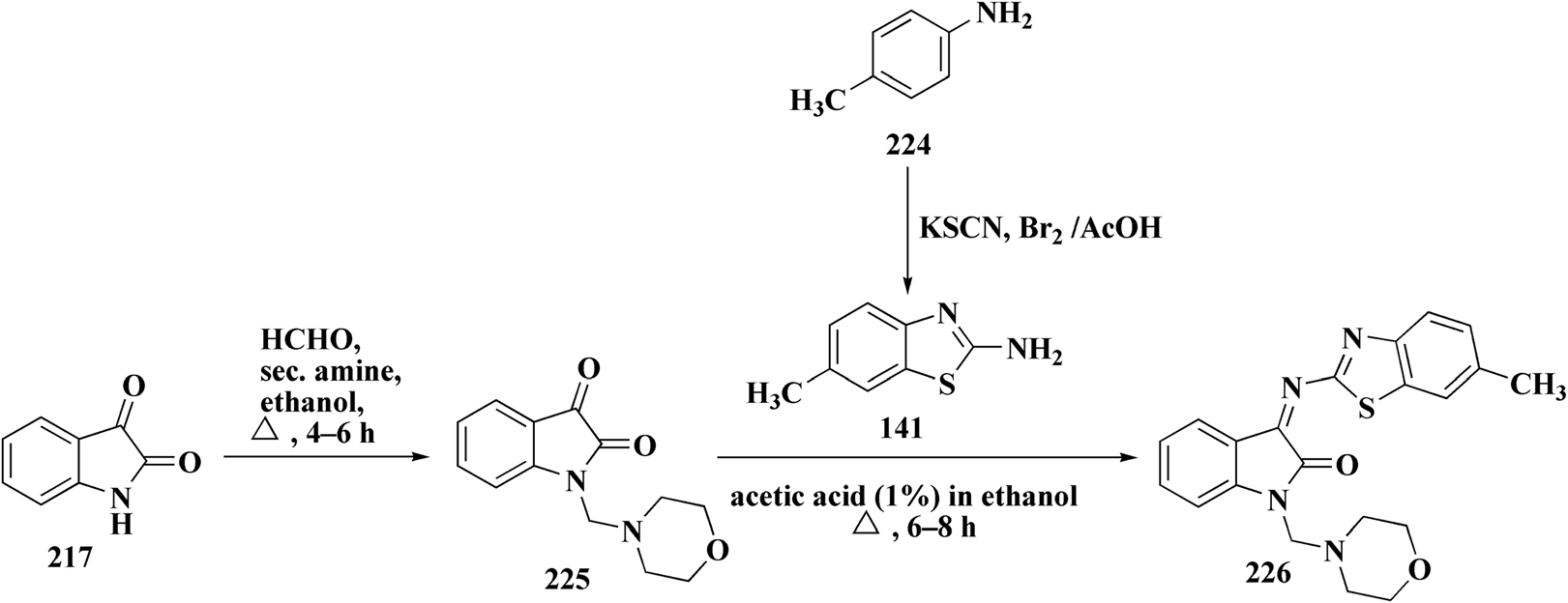
Synthesis of isatin-benothiaole analogs.

The isatin-benzothiazole analogs were synthesized as outlined in scheme. Compounds 225 were synthesized through condensing the isatain with formaldehyde and secondary amino function of amino component. The paraformaldehyde (PFA) and an amino component were dissolved in ethylalchol and iminium ion yielded *in situ* was then reacted with 1*H*-indole-2,3-dione derivatives to generate compounds 225. The intermediate compound 2-amino-6-methylbenzothiazole was prepared from 4-toluidine, in which the substituted aniline was reacted with potassium thiocyanate and bromine in ethanoic acid. Compounds 226 were produced *via* condensation of compound 225 with 6-methyl-benzothiazol-2-ylamine (Scheme 37).

The cytotoxicity of these compounds was exhibited using three human breast tumor cell lines, and two non-cancer breast epithelial cell lines. Compound 226 is considered as the most potent compounds of this series.^179^

**Fig. 23:**
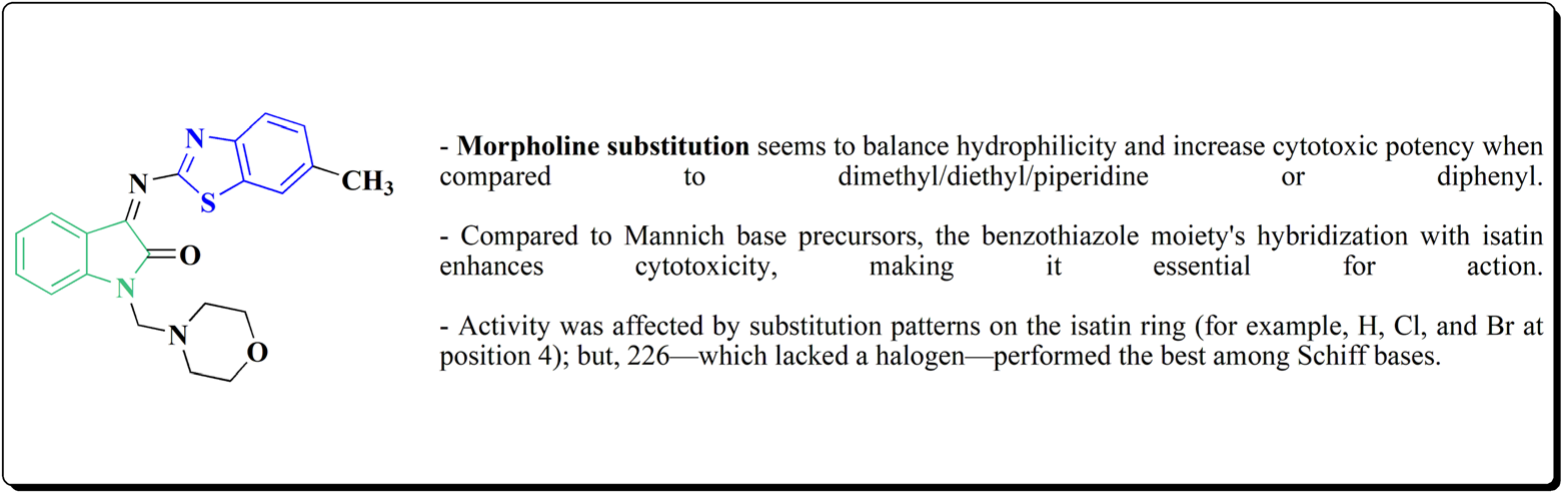
Structure–activity relationship of compound 226.

Among the most effective compounds against the three examined cell lines of breast cancer was compound 226, GI_50_ values: MDA-MB231: 17.61 μM, MDA-MB468: 19.76 μM, & MCF7: 14.56 μM. GI_50_ for MCF7 is 25.77 μM, which is higher than cisplatin. Compared to non-cancerous breast cells, compound 226 (Fig. 23) exhibited more cytotoxicity toward cancer cells.

There is little correlation between cell cycle arrest and 5e's cytotoxic impact. In MCF7 or MCF10A cells, 226 did not stop the cell cycle at 20 μM. Only MCF7 cells showed a little accumulation in the G_2_/M phase at 40 μM. This implies that 226 causes cytotoxicity by a mechanism other than cell cycle arrest, potentially involving: oxidative stress, dysfunction of mitochondria, and non-cell-cycle mechanisms leading to apoptosis (not verified in this study).^179^

#### 8.1.7. Piprazine–benzothiazole based analogues

**Scheme 38:**
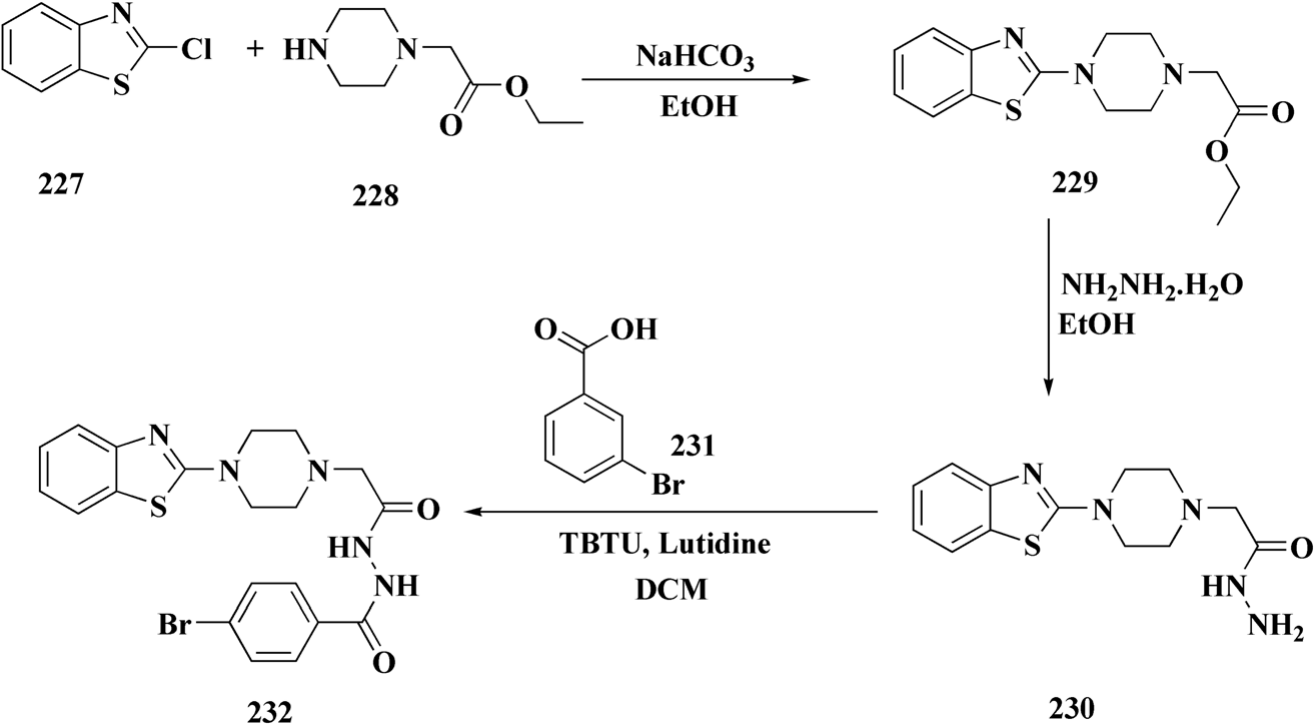
Synthesis of piprazine–benzothiazoe analogs.

2-Chlorobenzo[*d*]thiazole 227 was reacted with the piperazinyl acetate 228 to afford compound 229. The latter compound 229 was reacted with hydrazine hydrate to afford the hydrazide 230. Further treatment of compound 230 with benzoic acids 231 in the presence of TBTU and 2,6-dimethyl pyridine (lutidine), yielded the targeted compounds 232 (Scheme 38).

**Fig. 24:**
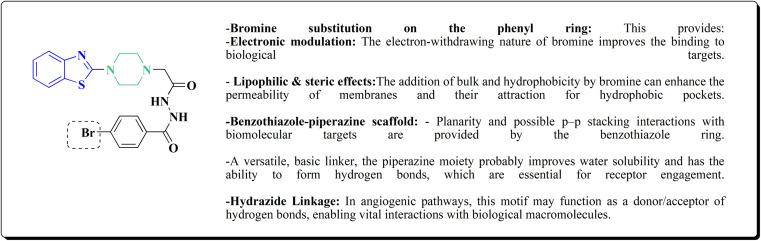
Structure–activity relationship of compound 232.

The cell viability assay and *in vitro* cytotoxicity of compounds 232 were tested against Dalton's lymphoma ascites (DLA) cells. Compound 232 indicated promising antiproliferative efficacy (Fig. 24). Subsequent investigation of compound 232 on *in vivo* treatment model indicated increased tumor suppression through inhibition of angiogenesis.^180^

### 8.2. Benzothiazoles linked with substituted aromatics

**Scheme 39:**
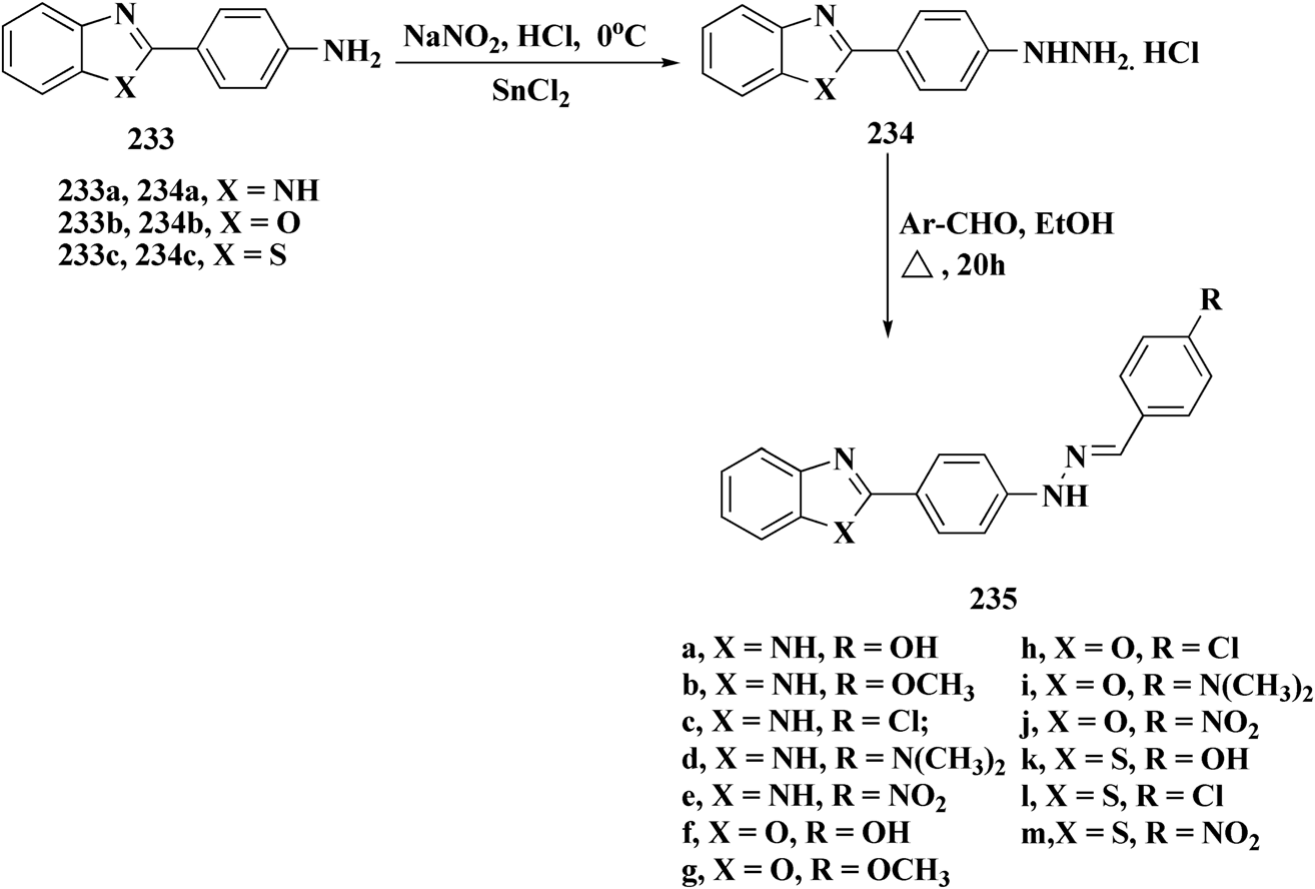
Synthesis of 2-substituted benzothiazoles 235k–m.

The phenyl hydrazine hydrochlorides 234a–c were synthesized *via* the diazotization of aromatic amines of the benzothiazoles 233a–c followed by their reduction. Condensation of compound 2a–c with various aromatic aldehydes yielded the desired compounds 235a–m (Scheme 39).

**Fig. 25:**
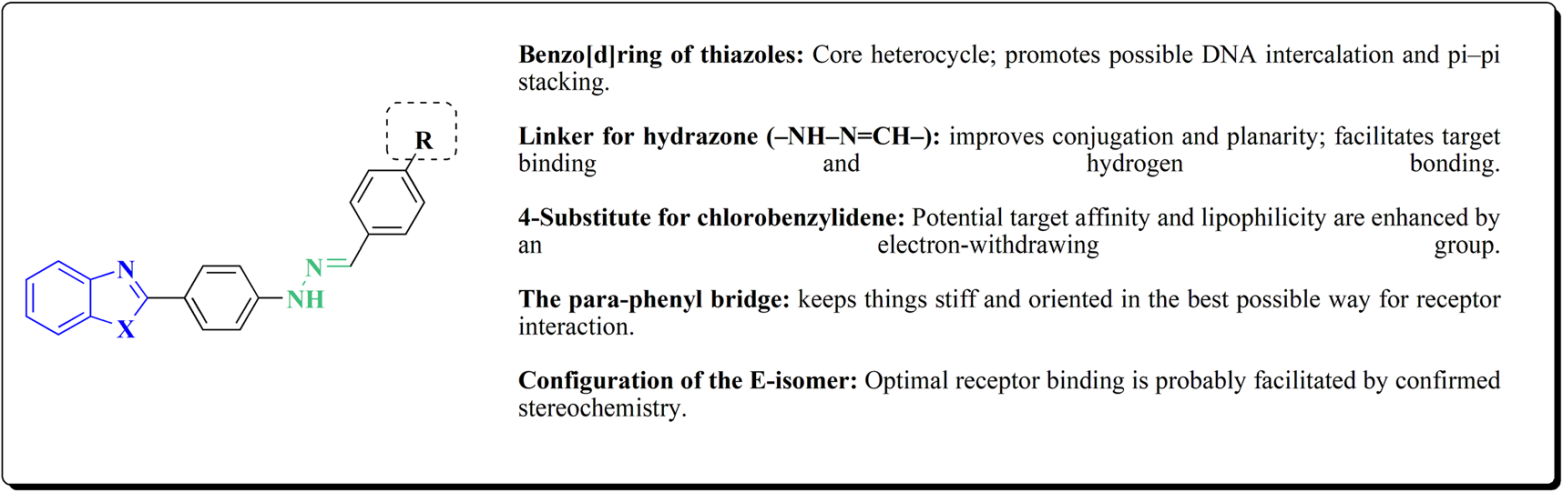
Structure–activity relationship of compound 235.

The novel compounds were examined *in vitro* against both human hepatic adenocarcinoma (HepG2) and human breast adenocarcinoma (MCF-7) cell lines. The most potent compounds 235h (IC_50_ = 0.067 μm against MCF-7) and 235l (IC_50_ = 0.027 μM against HepG2) were tested subsequently for EGFR inhibitory activity (Fig. 25).^181^

**Scheme 40:**
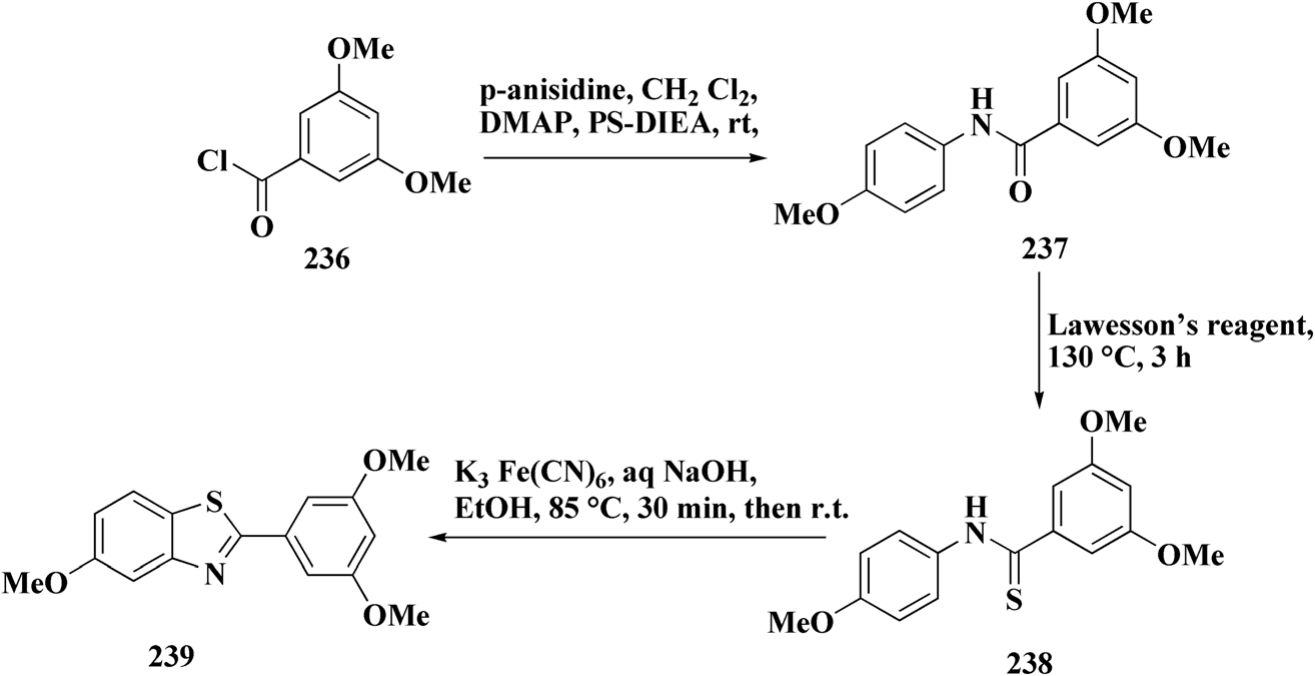
Synthesis of resveratrol (RES) analogs.

Novel resveratrol (RES) analogs were developed. The amide 237 was afforded through condensing of 3,5-dimethoxybenzoyl chloride 236 with *p*-anisidine. Treating this amide with the Lawesson's reagent (LR) afforded the thioamide 238, which was then exposed to an oxidative cyclization (Scheme 40).

**Scheme 41:**
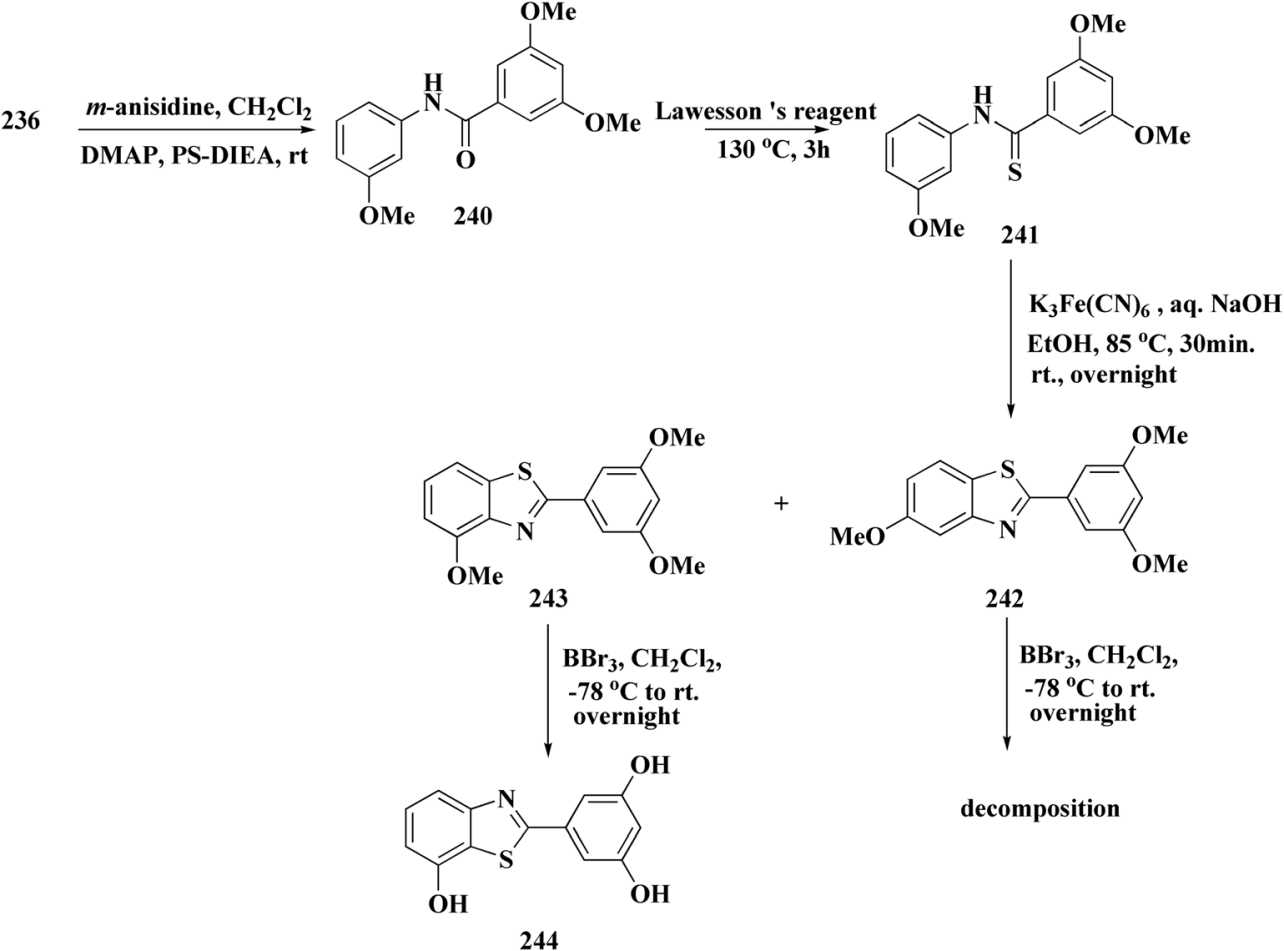
Synthesis of resveratrol (RES) analogs.

**Scheme 42:**
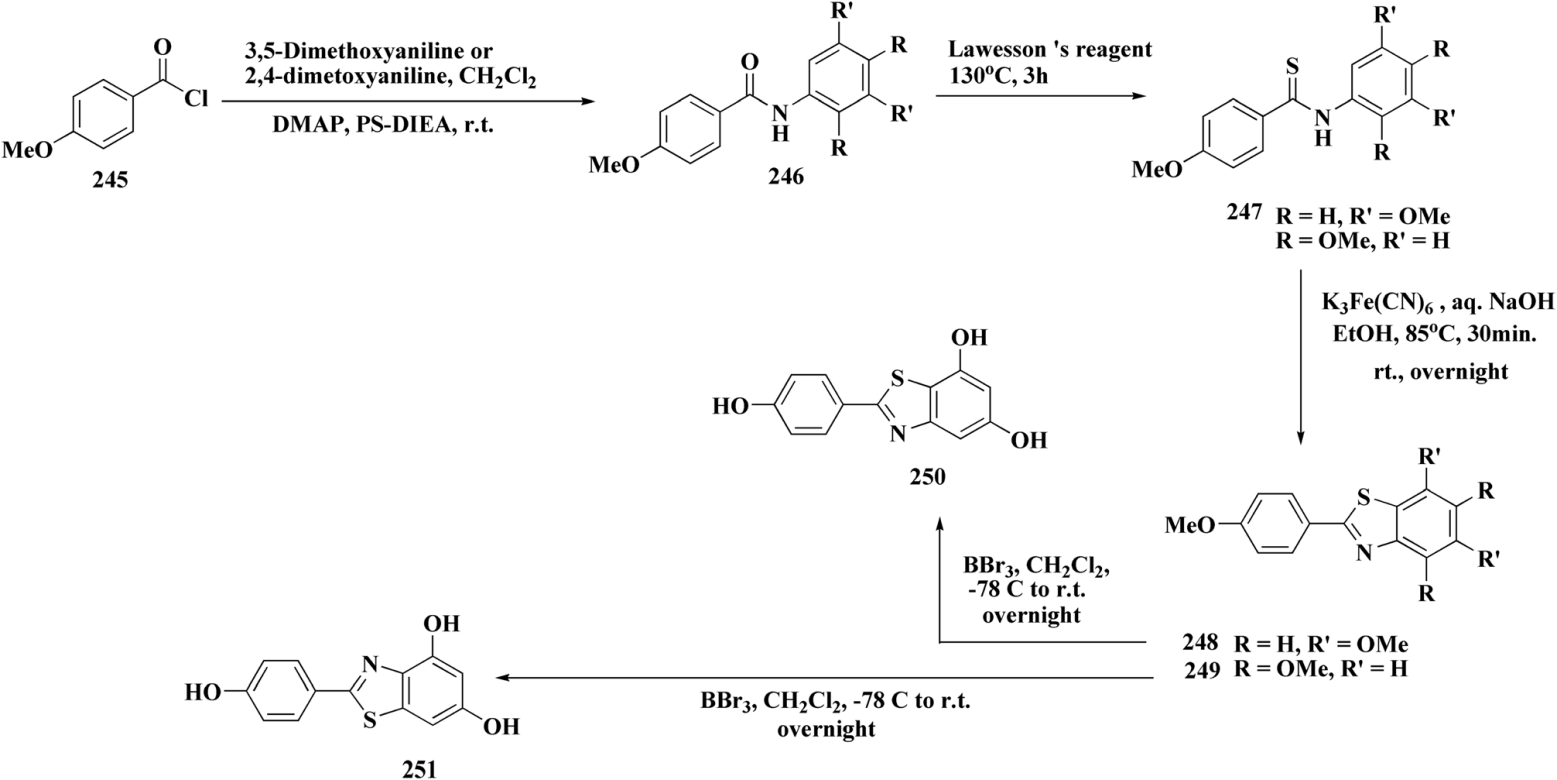
Synthesis of resveratrol (RES) analogs.

The acid chloride 236 was reacted with *m*-anisidine to afford amide 240. Treating the latter with Lawesson's reagent furnished the thioamide 241. The oxidative cyclization step produced, a mixture of the isomeric compounds 242 & 243. The BBr_3_-promoted *O*-demethylation of 243 led to compound 250. The synthesis of benzothiazoles 250 and 251 was accomplished in Scheme 41. Condensation of 3,5-dimethoxyaniline with 4-methoxybenzoyl chloride 245 generated the amide 206. The thioamide 248 was generated using Lawesson's reagent then the latter compound was submitted to the ferricyanide-promoted cyclization. Compound 248 was then deprotected to generate compound 250. Compound 251 was accessed subsequent an indistinguishable synthetic approach, with the only modification consisting in condensing 245 with 2,4-dimethoxyphenylamine (Scheme 42).

The resulting analogs were estimated for their antiproliferative and vasorelaxing effect.

**Fig. 26:**
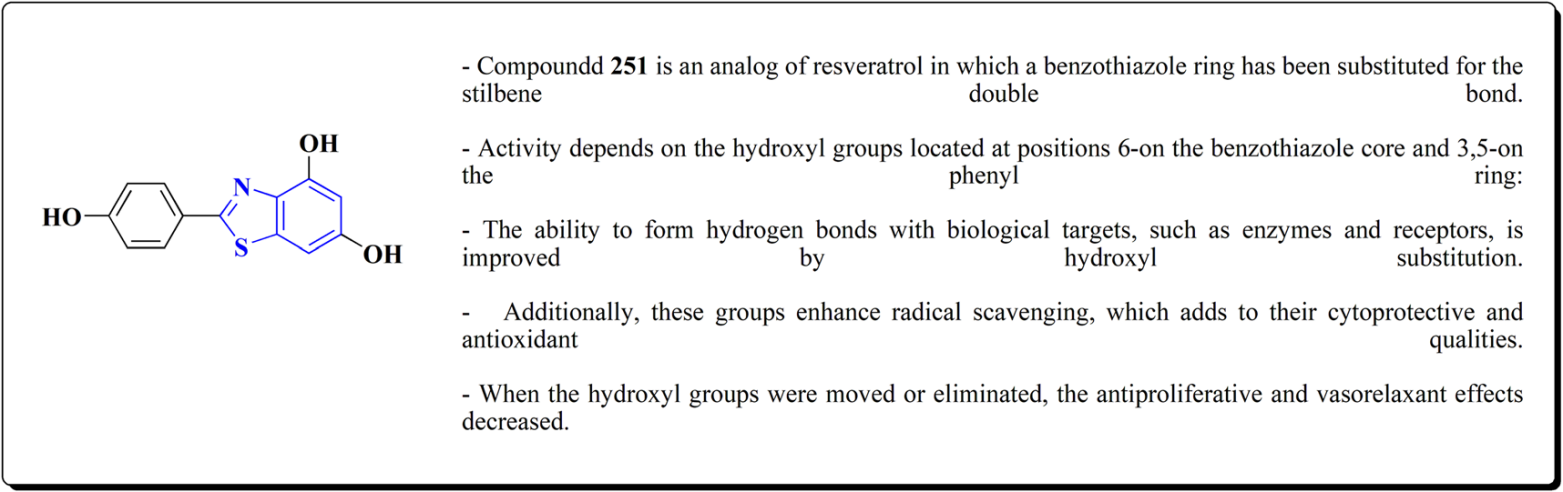
Structure–activity relationship of compound 251.

2-(3,5-Dihydroxyphenyl)-6-hydroxybenzothiazole had the highest levels of potency (pIC 50 = 4.92) and efficacy (*E*_max_ = 88.2%) in vascular testing (Fig. 26).^182^

**Scheme 43:**
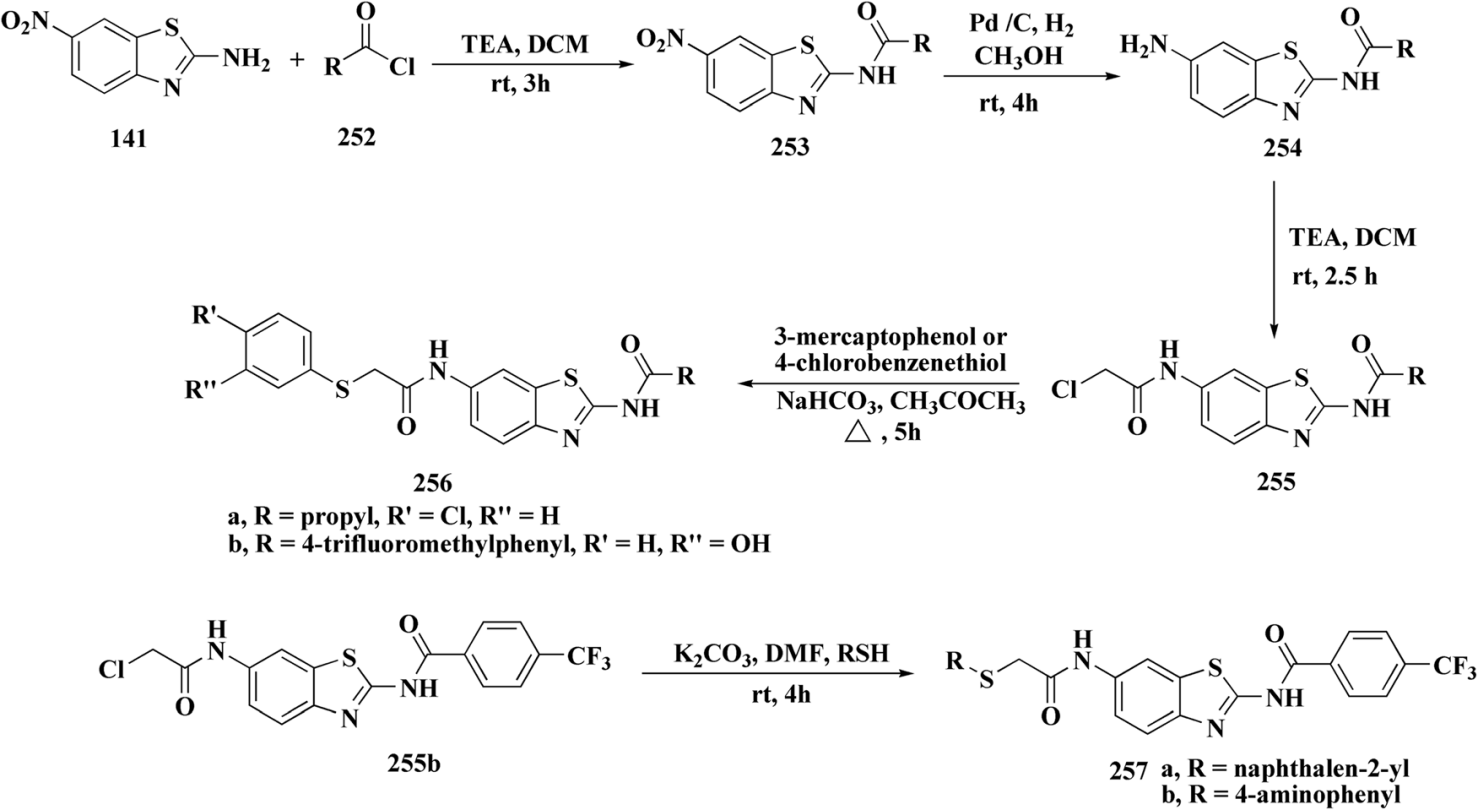
Synthesis of heterocycle-based analogs of resveratrol.

Compound 256a with best anti-tumor potencies was selected as a lead structure. The desired compounds were prepared *via* a multi-step substitution reaction using the benzothiazole 141. On the amide side, phenyl replacements were inserted while the thioether with benzyl substitution was remained. Compounds 256 were afforded in good yield. Compounds 257 were generated by substitution with compound 255 (Scheme 43).

Compounds 256b, 257a, 257b showed antitumor potencies. 257b indicated high anticancer potency against HCT116 colon cancer cells.^183^

**Fig. 27:**
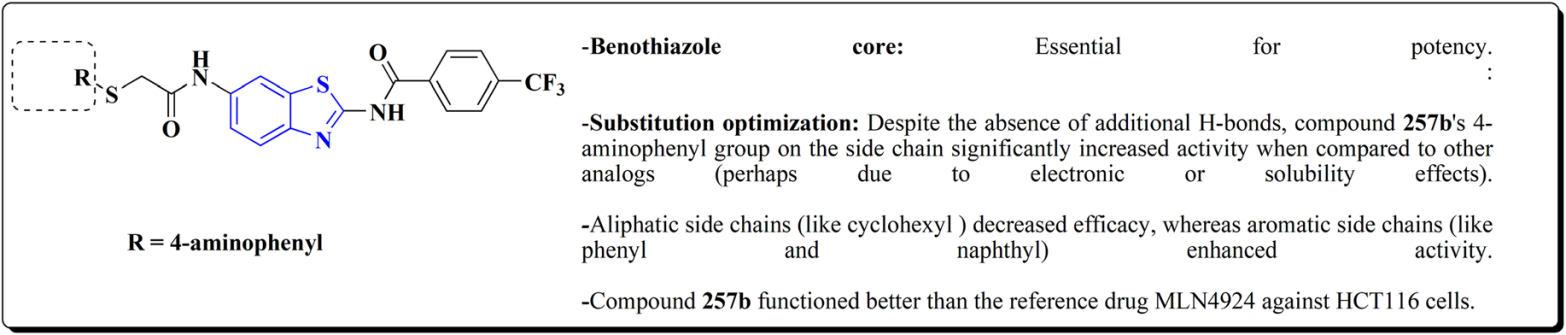
Structure–activity relationship of compound 257.

The non-sulfamide NEDD8-activating enzyme (NAE) is inhibited by compound 257b (Fig. 27). The mechanism can be summed up as follows: inhibition of the NEDD8 pathway-NAE plays a critical role in triggering the NEDD8 cascade, a ubiquitin-like post-translational modification that is necessary for controlling cullin-RING ligases (CRLs), which determine how important cell cycle proteins degrade.

Compound 257b forms three hydrogen bonds (Asp100, Ile148, and Gln149) and one extra H-bond with Asp167 by binding non-covalently to NAE and imitating the main hydrogen-bonding interactions observed with the natural AMP substrate. This binding effectively blocks the NEDD8 pathway, causing cancer cells to undergo apoptosis by lowering cellular levels of NEDD8 and accumulating UBC12, a downstream target. The mechanism at the molecular level was validated by a dose-dependent drop in NEDD8 levels and a matching increase in UBC12 levels.^183^

**Scheme 44:**
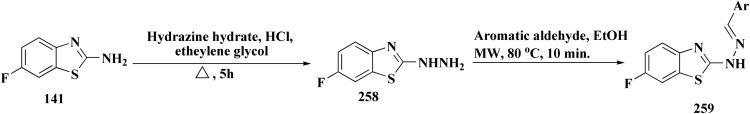
Synthesis of substituted fluorobenzothiazole.

Hydrazine derivatives have several biological potencies.^184–186^ 2-Amino-6-fluorobenzothiazole 141 was allowed to react with hydrazine hydrate to give the hydrazine derivative 258. Reacting compound 258 with aromatic aldehyde using microwave irradiation afforded compounds 259 (Scheme 44).

**Fig. 28:**
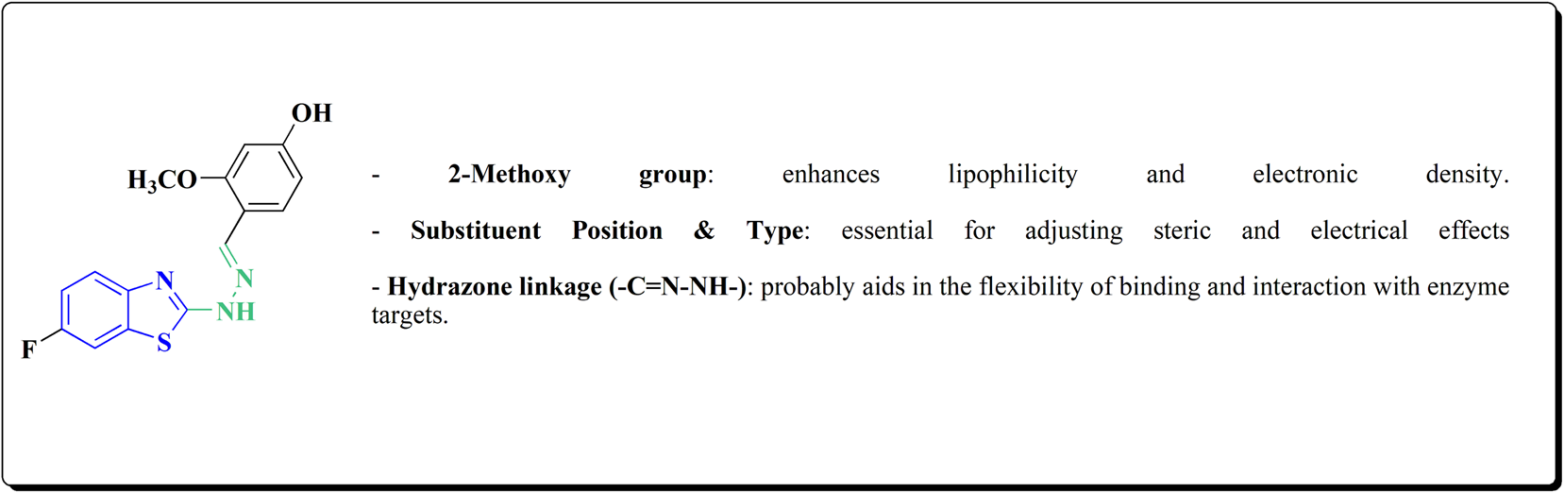
Structure–activity relationship of compound 259.

The antitumor potency of compounds 259 (Fig. 28) against kidney fibroblast cancer (COS-7) and cervical cancer (HeLa) cell lines was tested. Compound 259 revealed excellent potency against COS-7 cell line (IC_50_ value = 4.31 μmol L^−1^) as compared to doxorubicin (IC_50_ value = 3.04 μmol L^−1^).^187^

**Scheme 45:**
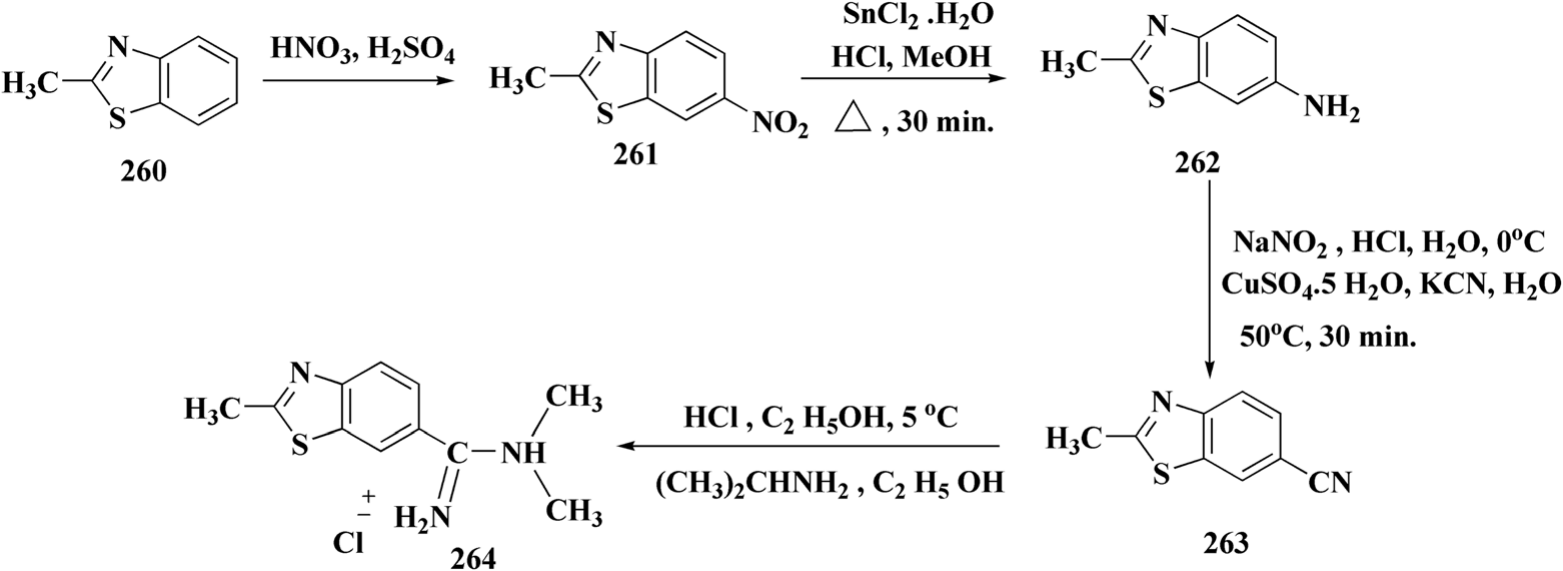
Synthesis of substituted benzothiazole derivatives.

**Scheme 46:**
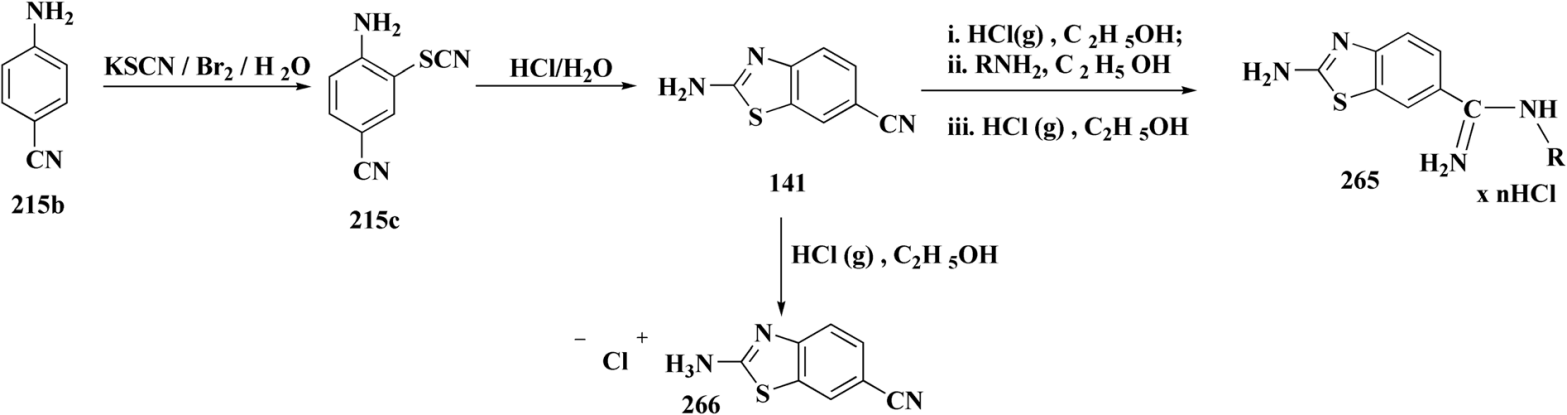
Synthesis of substituted benzothiazole derivatives.

The preparation of the compound 264 was started from compound. Then compound 264 was reduced to reduced to the amino analog which was transformed in the compound 263. Cyano compound 263 in the Pinner reaction was transformed into the amidino compound 264. Similarily, compound 266 was synthesized from 6-cyano-2-aminobenzothiazole. Amidino compounds 265 were obtained as outlined in Schemes 45 & 46.

**Scheme 47:**
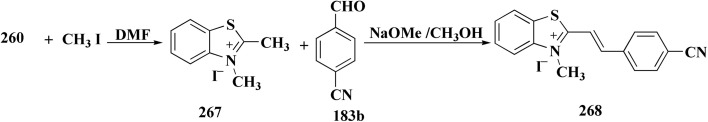
Synthesis of *N*-methyl iodide salts of the substituted benzothiazole.

*N*-Methyl iodide salts of the substituted benzothiazole 267 were synthesized *via* quaternization of the corresponding benzothiazole followed by condensation (Scheme 47).

**Scheme 48:**
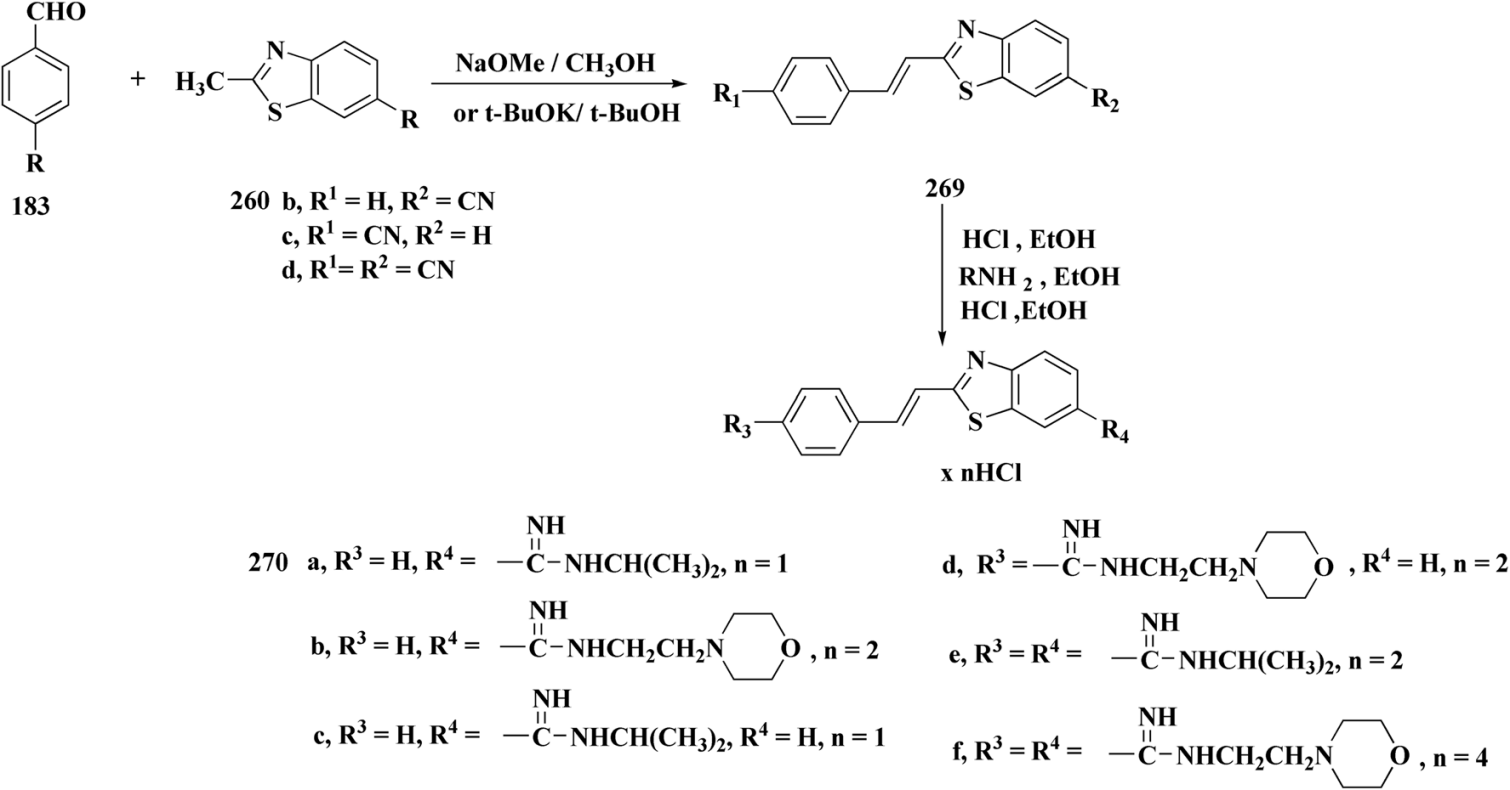
Synthesis of amidino substituted styryl benzothiazoles.

Amidino substituted styryl benzothiazoles 270 were furnished from the corresponding benzothiazole through the condensation with benzaldehyde and subsequent Pinner reaction (Scheme 48).

**Fig. 29:**
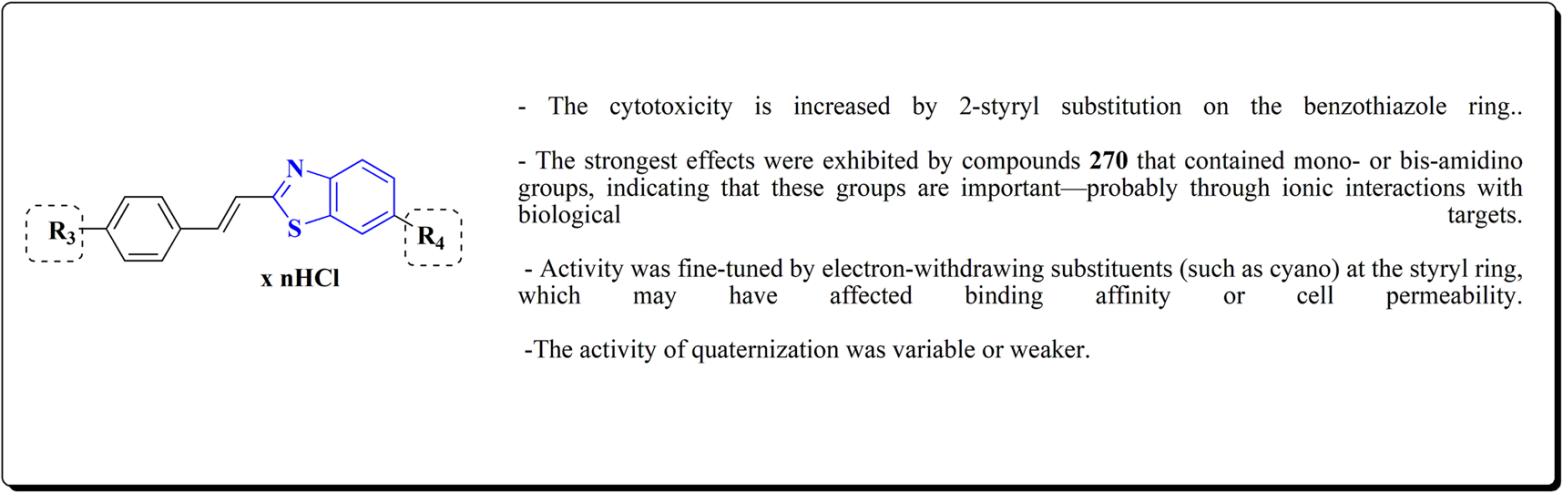
Structure–activity relationship of compound 270.

The compounds were examined on the cytostatic potencies against malignant cell lines. The best inhibitory effect was obtained with compounds 270 (Fig. 29). All of them inhibited the growth of the tested tumor cell lines and similarly normal fibroblasts. Other tested compounds displayed a moderate inhibitory activity, reliant on the type of the cells. Most of them inhibited the growth of WI38 and HeLa cells.^188^

**Scheme 49:**
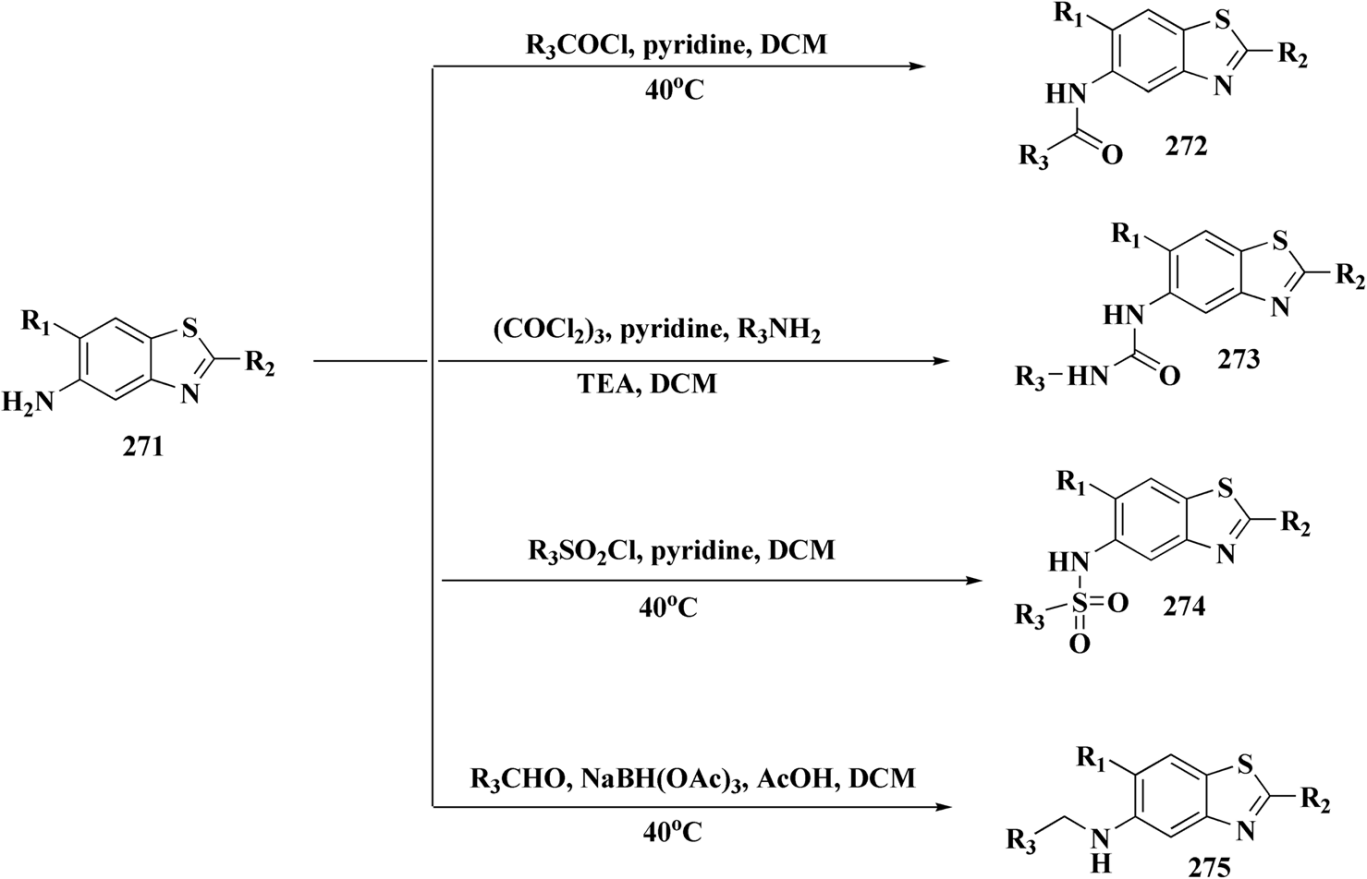
Synthesis of 2-substituted benzothiazole derivatives.

The intermediate 271 could be converted into urea derivative 273, directly be acylated or sulfonylated to access the amide 272 or sulfonamide 274. Additionally, a reductive alkylation was carried out to afford secondary amine 275 (Scheme 49).

**Fig. 30:**
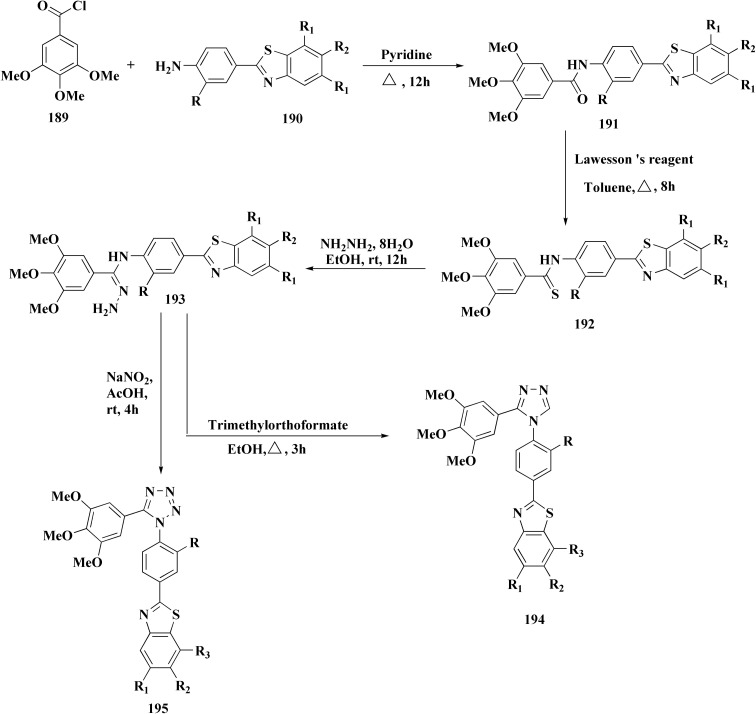
Structure–activity relationship of compound 273 & 275.

The screening of the compounds for their antitumor potency against 60 human cancer cell lines was performed. Compounds 273, 275a and 275b, indicating high activity (Fig. 30). The compound 273 afford its average 50% growth inhibition (GI_50_) at 0.38 μM.^189^

**Scheme 50:**
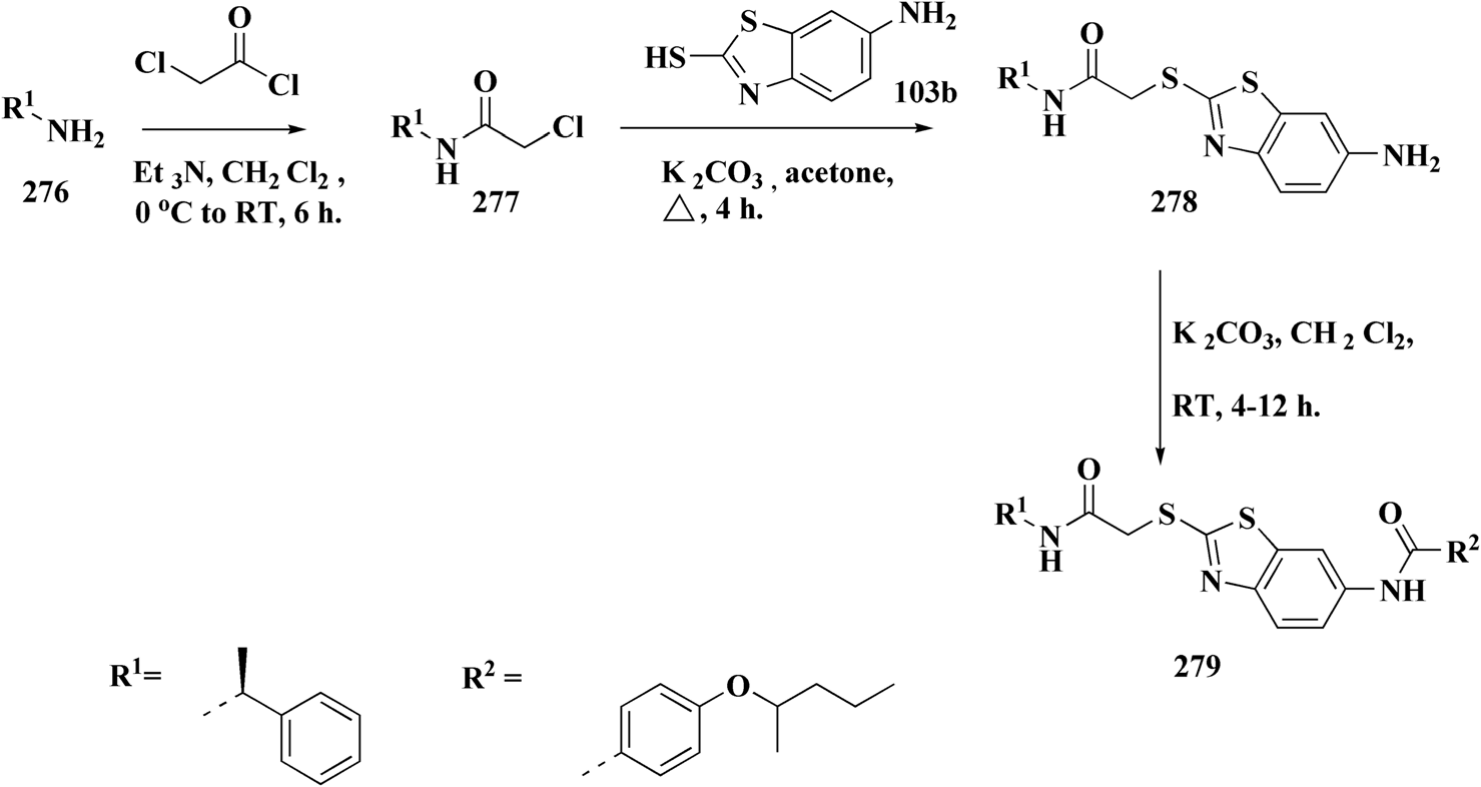
Synthesis of substituted (benzo[*d*]thiazol-6-yl)benzamides.

The synthesis of benzothiazole-based derivatives 279 is accomplished in Scheme 50. Compounds 277 were synthesized from phenylamine or benzylamine derivatives, and 2-chloroacetyl chloride. Compound 277 were reacted with 6-aminobenzothiazole-2-thiol to afford the amines 278. Compounds 279 were furnished through the reaction of compound 278 with the corresponding acyl chloride.^190^

**Scheme 51:**
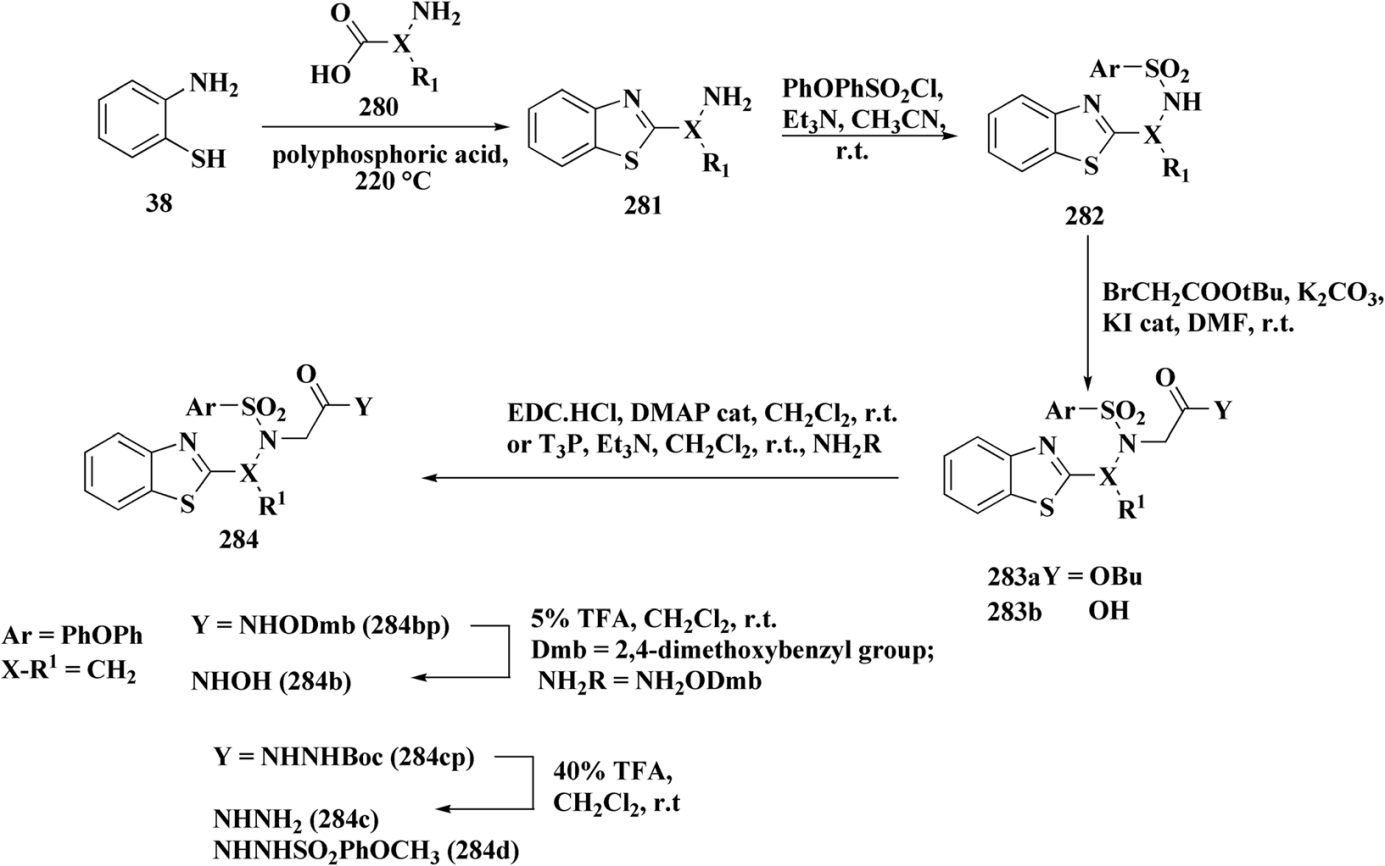
Synthesis of benzothiazole carboxylic acid analogs.

Sulfonamide moiety is considered as a key element in synthesizing a wide variety of significant heterocyles.^191–195^ Benzothiazoles containing sulfonamides were reported as shown in Scheme 56. The synthesis of the benzothiazole-amine fragments 281 from the corresponding amino acid (or ester) and *o*-aminothiophenol using the polyphosphoric acid.^196^ The arylsulfonamide analogues 282 were synthesized *via* coupling of the arylsulfonyl chloride with the BTA-amines. These sulfonamides were then allowed to react with bromoacetic acid *tert*-butyl ester to afford compounds 283a; their *tert*-butyl deprotection generated the carboxylic analogues 283b. Coupling the latter compounds with various amine-bearing derivatives. These reactions furnished the hydroxamic acids *O*-protected with 2,4-dimethoxybenzyl group (Dmb) 284, the hydrazides protected with the arylsulfonylhydrazide or *tert*-butyloxycarbonyl group (Boc). Deprotection of the latter afforded the hydroxamic acids or hydrazides (Scheme 51).^196^

The evidenced BTA-associated potency improvements on cell antiproliferactivity and enzyme inhibition in addition to the hydrolytic stability showed by the hydrazide group, propose that these novel bifunctional BTA-hydrazides could be utilized for developing novel categories of MMPIs with anticancer potency.^196^

**Fig. 31:**
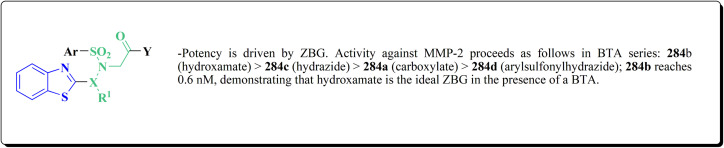
Structure–activity relationship of compound 284.

Since the trend hydroxamate > carboxylate > hydrazide > arylhydrazide indicates that the zinc-binding group (ZBG) is the main factor determining efficacy, the strongest inhibitors are hydroxamate derivatives. Although it cannot completely make up for a lower ZBG, the addition of a benzothiazole (BTA) moiety enhances enzyme binding and antiproliferative activities, especially in non-hydroxamate scaffolds (Fig. 31). Strong MMP inhibition is supported throughout the series by the aryl-sulfonyl (4-phenoxyphenylsulfonyl) motif, which fits into the S1′ pocket and forms stabilizing H-bonds with Leu191 and Ala192.

The most efficient zinc-binding moiety in this series was the hydroxamate group; compound 284b (Fig. 31), a benzothiazole–hydroxamate derivative, had significant antiproliferative action (A2780 IC_50_ ≈ 10.5 μM) in addition to sub-nanomolar inhibition of MMP-2 (IC_50_ = 0.6 nM). Even though the parent hydroxamate, *N*-hydroxy-2-(4-phenoxyphenylsulfonamido)acetamide, was still marginally more effective (0.46 nM) because of an additional stabilizing H-bond with Pro423, the benzothiazole substituent improved binding by generating more hydrophobic and π–π interactions inside the active site. Overall, the SAR shows that arylsulfonyl anchor and hydroxamate ZBG are essential for potency, while benzothiazole scaffold enhances the biological profile even more.^196^

**Scheme 52:**
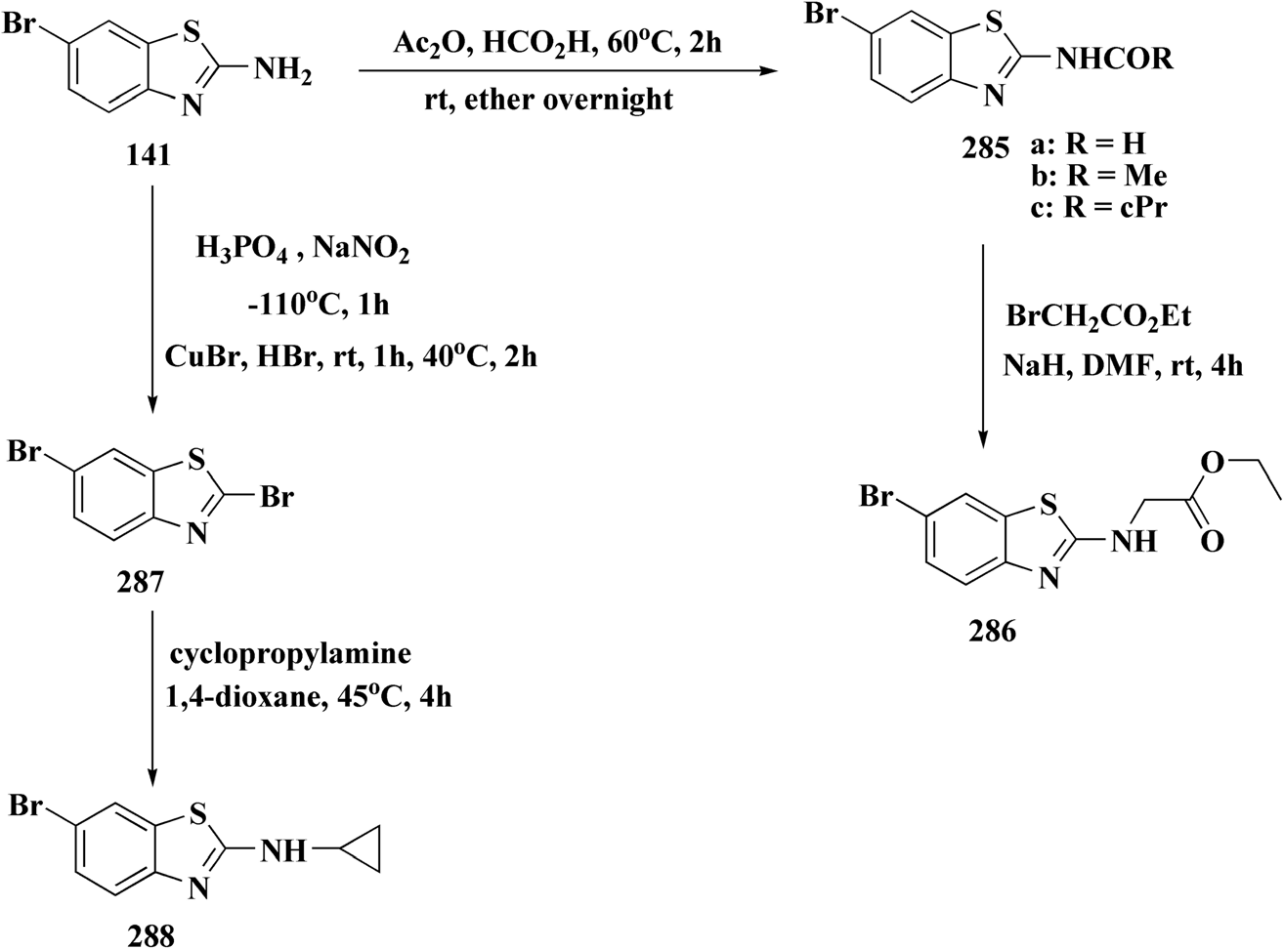
Synthesis of substituted 2-amino-benzothiazole.

**Scheme 53:**
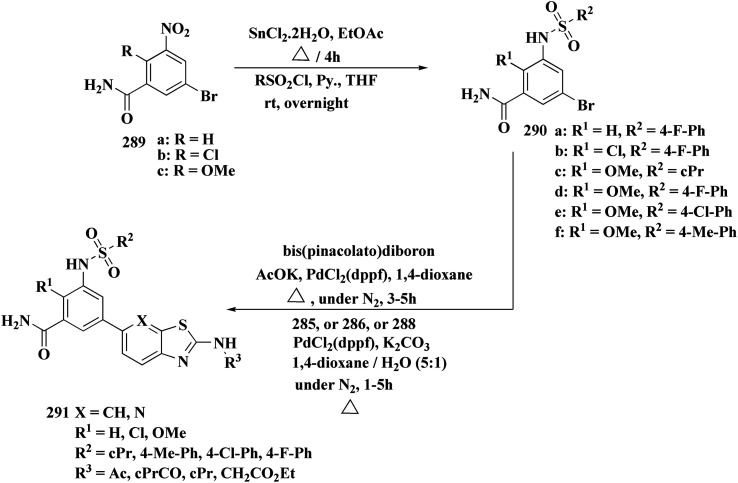
Synthesis of substituted 2-amino-benzothiazole.

The synthetic pathway of generating compounds 291 is accomplished in Schemes 52 and 53. In the presence of triethylamine or DMAP, compound 141 was acylated to afford intermediates 285. The reaction of the substituted benzothiazole 285 with ethyl 2-bromoacetate yielded compound 286 in the presence of hydridosodium. Conversion of 141 to 287 was performed through diazo-reaction and bromo-substitution. Compound 287 was transformed to 288*via* nucleophilic substitution of 287 with aminocyclopropane. The sulfonamide 290 were furnished from 5-bromobenzoic acid derivatives. Catalyzed using PdCl_2_(dppf), intermediate 290 was allowed to react with pinacol diborane to give the arylboronic esters. Without isolating the arylboronic esters, the intermediate 285, or 286, or 288, were added to the reaction mixture. The latter mixture was allowed to reflux to afford compounds 291.

**Fig. 32:**
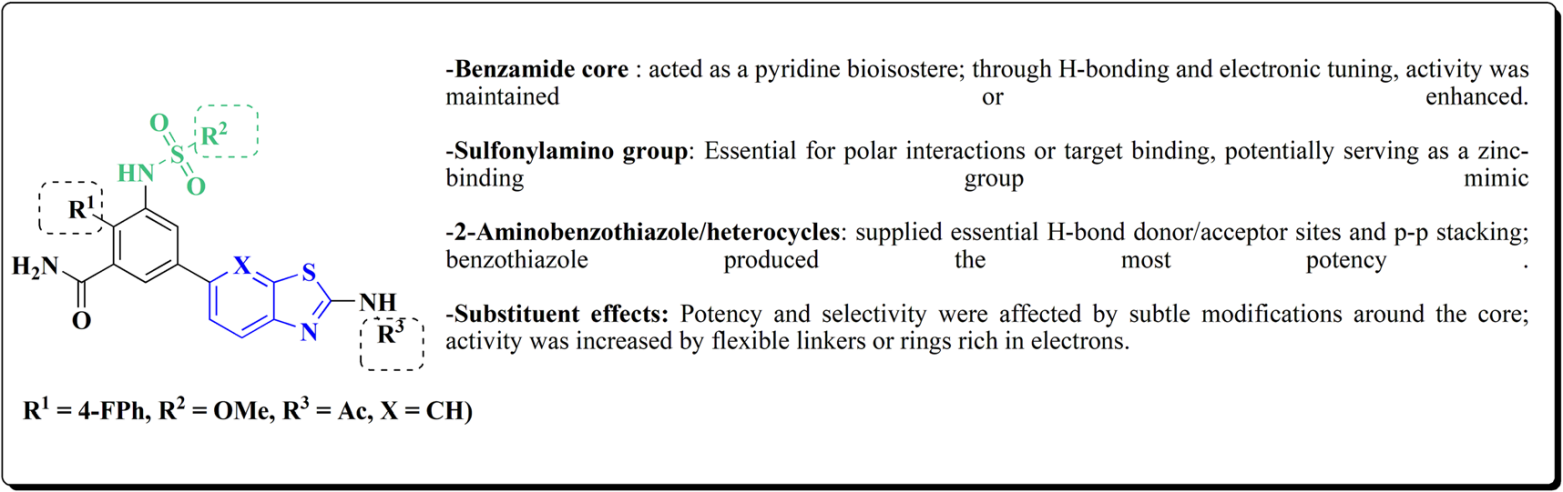
Structure–activity relationship of compound 291.

Their antiproliferative potencies were examined *in vitro via* MTT assay against human cancer cell lines comprising HCT-116, A549, U-87 MG & MCF-7. Compound 291 (R^1^ = 4-FPh, R^2^ = OMe, R^3^ = Ac, X = CH) (Fig. 32) with active anti-proliferative potency was tested for its effect on the AKT and *p*-AKT473.^197^

As dual PI3K/mTOR inhibitors, 2-substituted-3-sulfonylaminobenzamide is an effective bioisosteric substitute for pyridine. One promising lead is compound 291 (R^1^ = 4-FPh, R^2^ = OMe, R^3^ = Ac, X = CH), which has strong cytotoxicity *in vitro*, effectiveness *in vivo*, and unambiguous mechanistic confirmation. Compound 291 targets the PI3K/AKT/mTOR pathway, which is often upregulated in cancer. Western blot analysis revealed: phosphorylated AKT at Ser473 was downregulated, indicating that PI3K activation and downstream mTOR signaling should be blocked. This leads to decreased tumor cell growth, survival, and proliferation.^197^

**Scheme 54:**
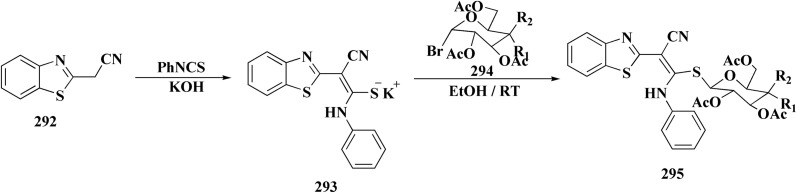
Synthesis of the *S*-glucosides 295a or *S*-galactosides 295b.

**Fig. 33:**
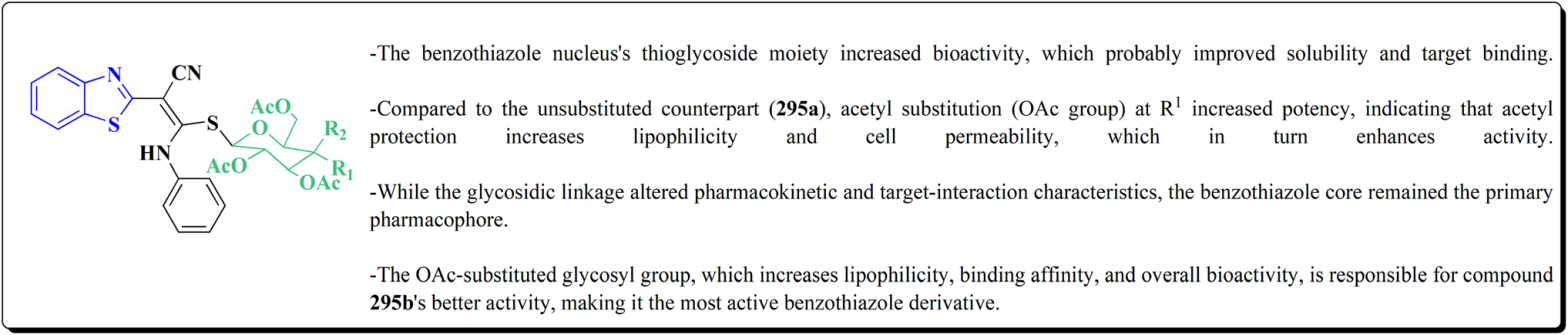
Structure–activity relationship of compound 295.

The benzothiazole acetonitrile 292 was reacted with phenylisothiocyanate to afford the corresponding thiolate salts 293. The latters was reacted with α-bromosugar 294 to yield the *S*-glucosides 295a or *S*-galactosides 295b (Scheme 54, Fig. 33).^198^

The cytotoxic activity of the compounds were assessed against MCF-7 cell lines (breast carcinoma cell lines) and demonstrated high to moderate anti-tumor activities. In addition, molecular modeling of these compounds showed that they have moderate selectivity through hydrogen bond interaction with the atypical nucleotide binding pocket in the amino terminus of HSP90 and high binding affinity through hydrophobic–hydrophobic interaction.

### 8.3. Benzothiazoles linked with amino acids

**Scheme 55:**
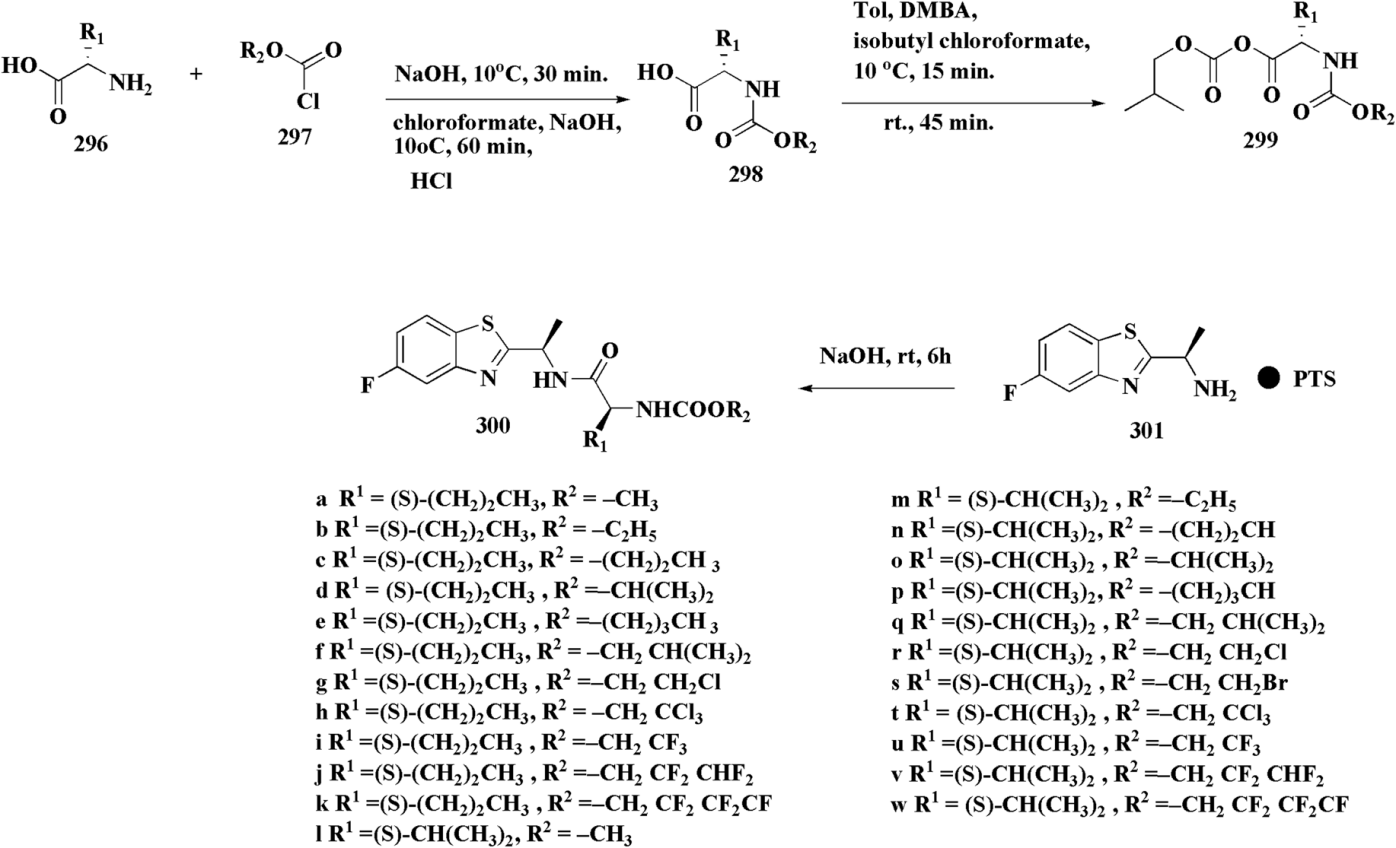
Synthesis of substituted fluorobenzothiazoles.

The synthesis of cholinesterase inhibitors based on fluorobenzothiazole is outlined in Scheme 55. The amino acid is reacted with chloroformate, and the obtained acid is then converted to a reactive derivate. This reactive intermediate reacts with the substituted ethanamine, which is released from its *p*-toluene sulphonate salt *in situ*. The targeted compound 300a–w were generated by vacuum concentration or filtration of the organic phase. The carbamates were evaluated for their ability to inhibit BChE and AChE.^199^

**Fig. 34:**
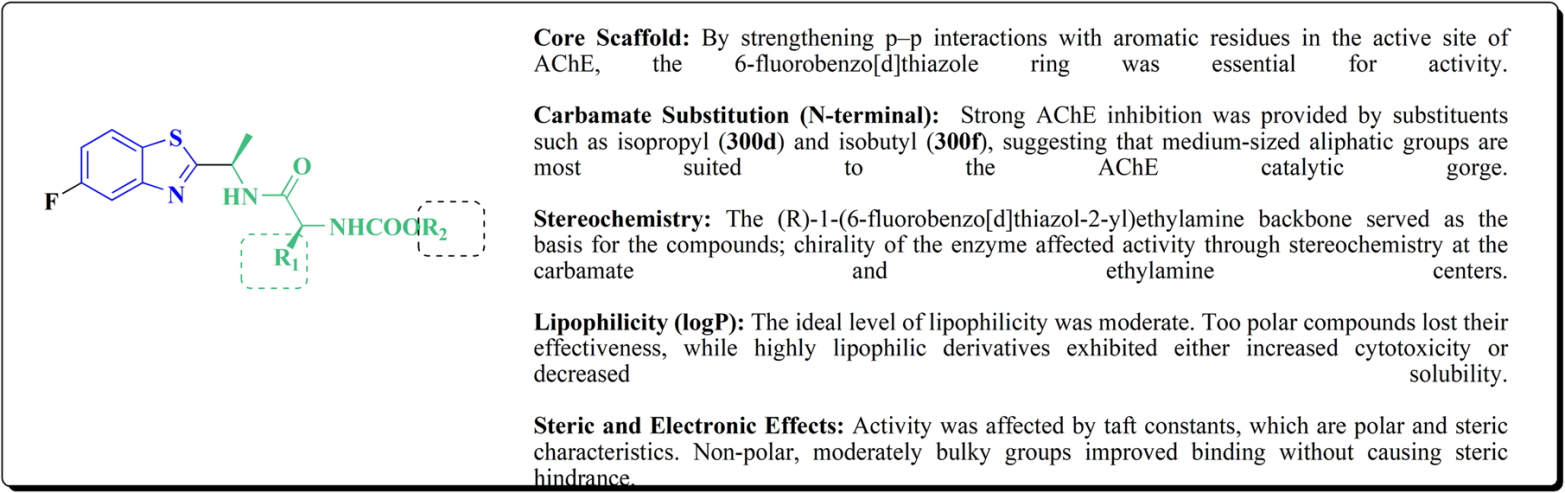
Structure–activity relationship of compound 300.

By comparing the compounds to the reference drugs galanthamine and rivastigmine, their inhibitory action against acetylcholinesterase (AChE) and butyrylcholinesterase (BChE) was assessed. Against AChE, two compounds, 300d and 300f, showed especially high potency (Fig. 34). Molecular docking studies of the most active compounds showed good π–π stacking with critical aromatic residues and appropriate orientation inside the catalytic region of AChE. Additionally, selected compounds showed acceptable cytotoxicity profiles in HepG2 and MCF-7 cell lines, indicating that they may be developed further in preclinical research.^199^

### 8.4. Benzothiazoles fused with heterocyclic compounds

#### 8.4.1. Benzothiazoles fused with six-membered ring heterocycles

**Scheme 56:**
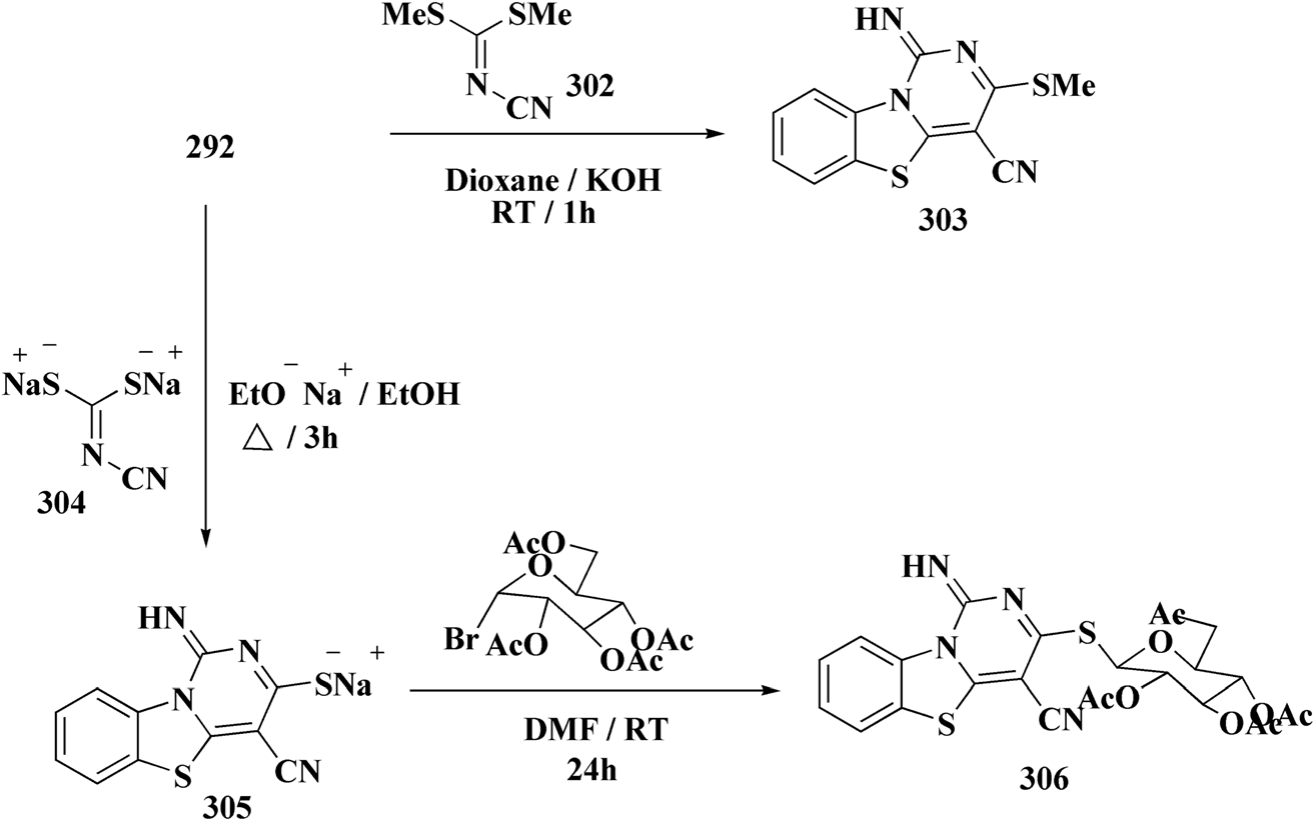
Synthesis of pyrimidobenzothiazole derivatives.

The pyrimidobenzothiazole derivative 303 was synthesized through reacting 2-cyanomethylbenzo[1,2-*b*]thiazole 292 with *S*,*S*-dimethyl *N*-cyanodithioimidocarbonate 302 (ref. 200) which is considered as a reactive reagent that we have recently used to synthesize various heterocycles.^201–205^ Sodium cyanocarbonimidodithioate salt 304 was reacted with compound 292 in the presence of sodium ethoxide to afford compound 305, and further reaction of 305 with α-bromosugar afforded compound 306 (Scheme 56).^200^

**Scheme 57:**
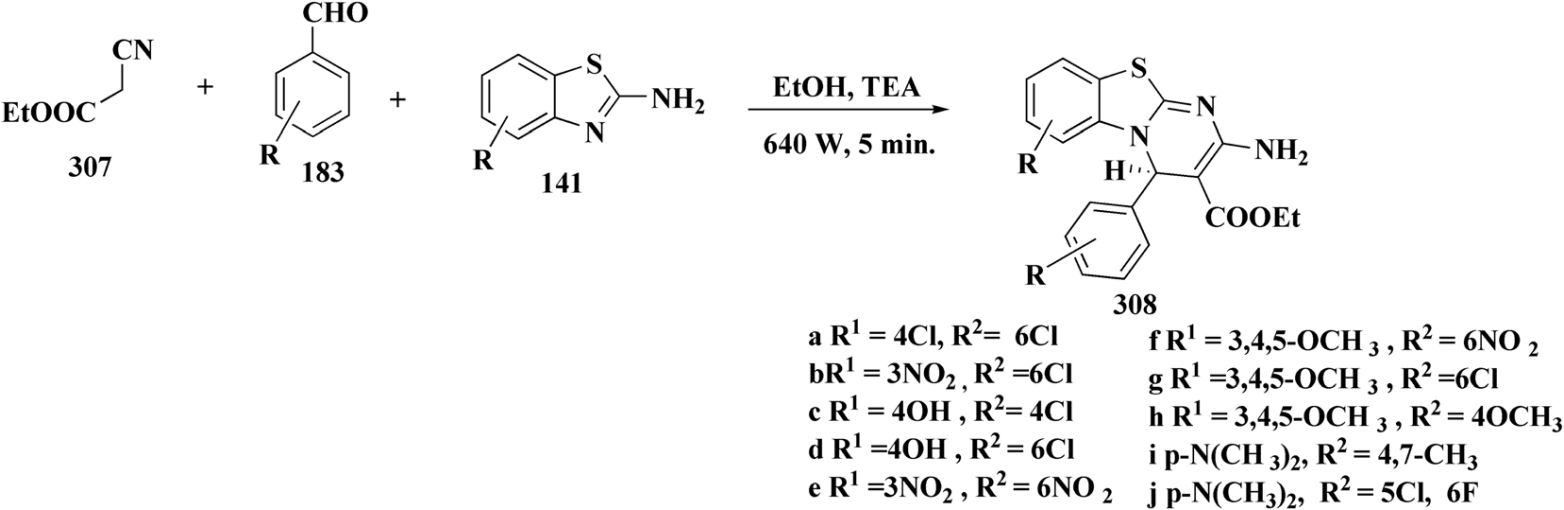
Synthesis of substituted pyrimidobenzothiazole derivatives.

Substituted pyrimidobenzothiazole derivative was synthesized *via* condensation of substituted benzothiazole, substituted benzaldehyde and ethyl cyanoacetate *via* microwave multi-component reaction (Scheme 57).

Compounds examined for *in vitro* against various human tumor cell lines, and for the *in vitro* anti-inflammatory activity. Compound 308i was found to have good antiproliferative activity and compound 308g was found to have high anti-inflammatory potency and compounds 308a, 308b, 308h also found to be effective anti-inflammatory potency.^206^

**Fig. 35:**
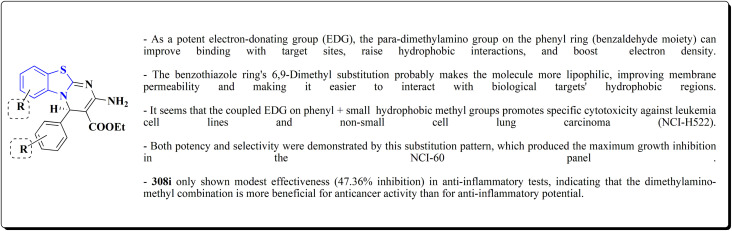
Structure–activity relationship of compound 308.

The synergistic action of electron-donating *para*-dimethylamino substitution on benzaldehyde and lipophilic methyl groups on the benzothiazole ring, which enhance potency and selectivity against NCI-H522 and leukemia cell lines, is responsible for 308i's strong anticancer activity (Fig. 35).

**Scheme 58:**
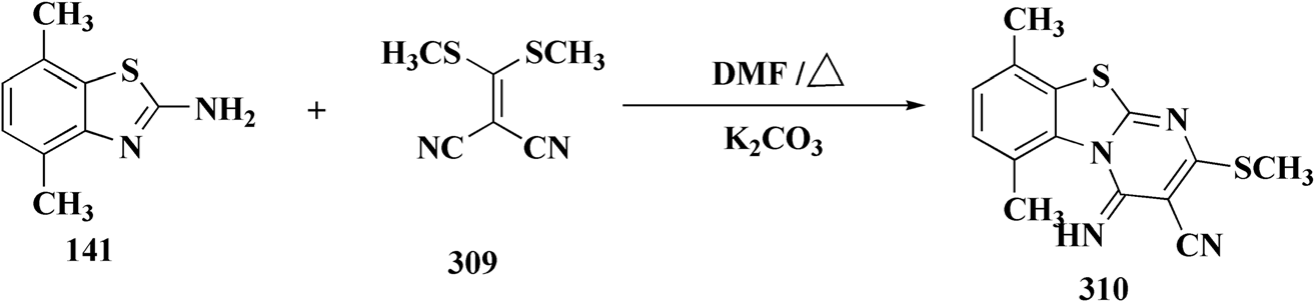
Synthesis of substituted pyrimido[2,1-*b*][1,3] benzothiazole.

**Scheme 59:**
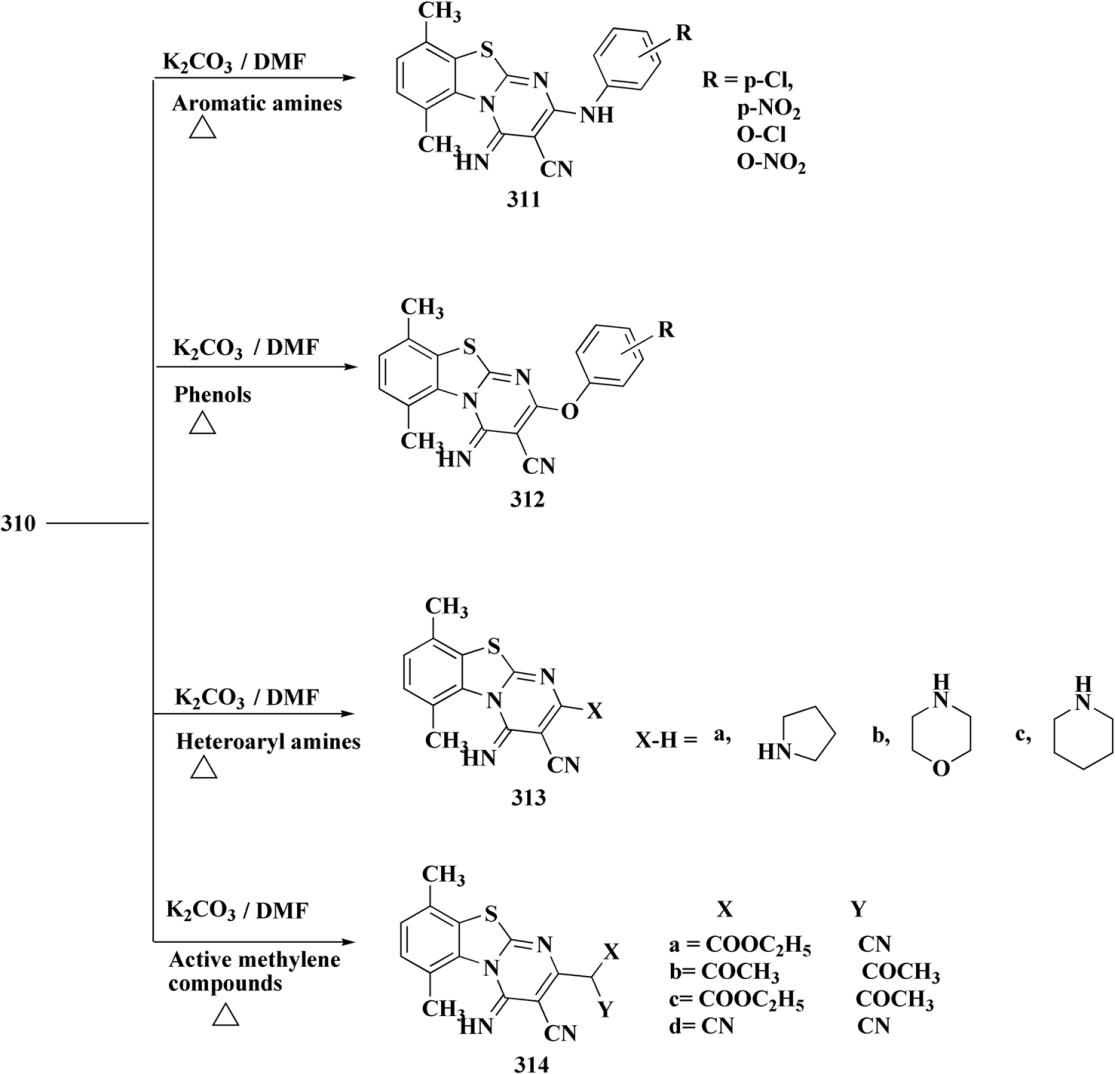
Synthesis of ssynthesis of substituted pyrimido[2,1-*b*][1,3]benzothiazole.

Along similar lines, the compound 310 has been synthesized by utilizing 2-amino 4,7-dimethyl benzothiazole 141 and by using bis-methylthio methylene malononitrile 309 ^207^ as reactive reagents. Its worthy to note that ketene dithioacetals have a cruial role in synthesizing a wide varity of bioactive heterocycles.^208–210^ Compound 310 synthesized from 2-amino 4,7-dimethyl benzothiazole 141 was reacted with bis-methylthio methylene malononitrile 309. The substituted derivatives 311–314 were furnished starting from compound 310 (Schemes 58 & 59).

**Fig. 36:**
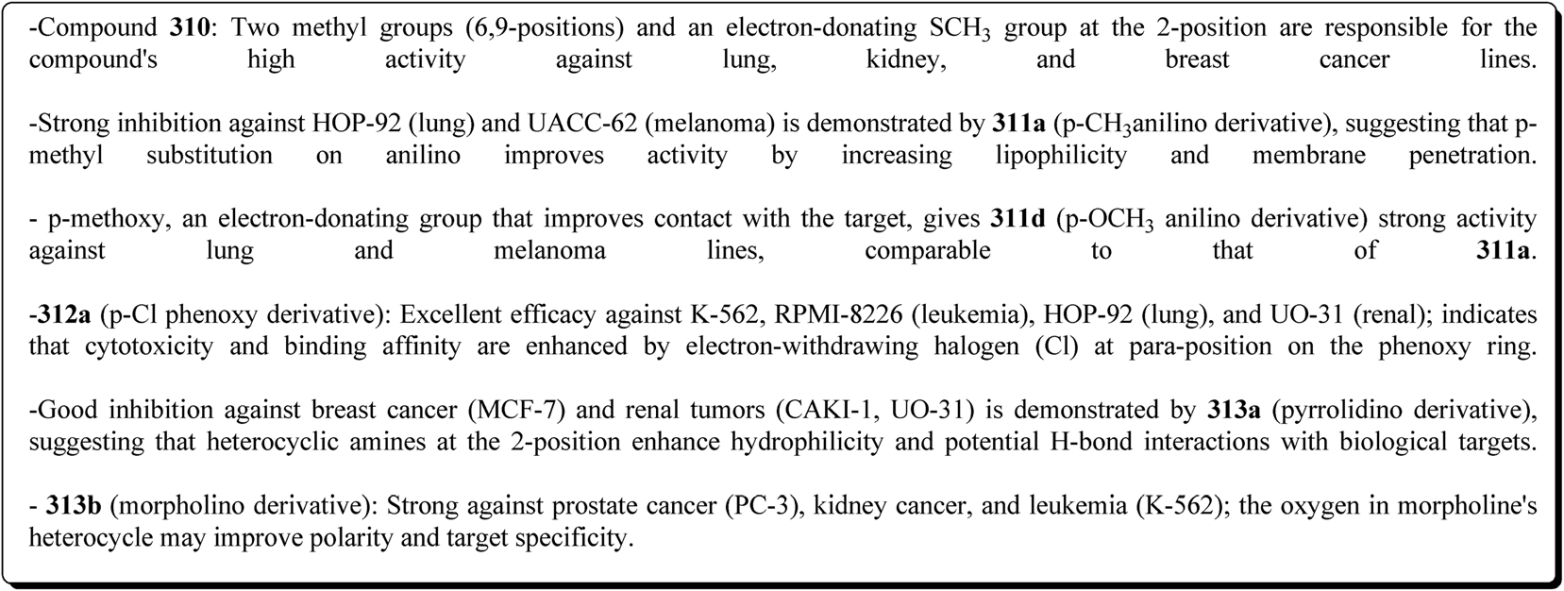
Structure–activity relationship of compounds 310–313.

Compound 310 and its selected derivatives 311–314 were screened for their *in vitro* anticancer potency. Compounds 310, 311-a, 311-d, 312-a, 313-a, 314-b displayed maximum *in vitro* anticancer activity against various cancers lines (Fig. 36).^207^

**Scheme 60:**
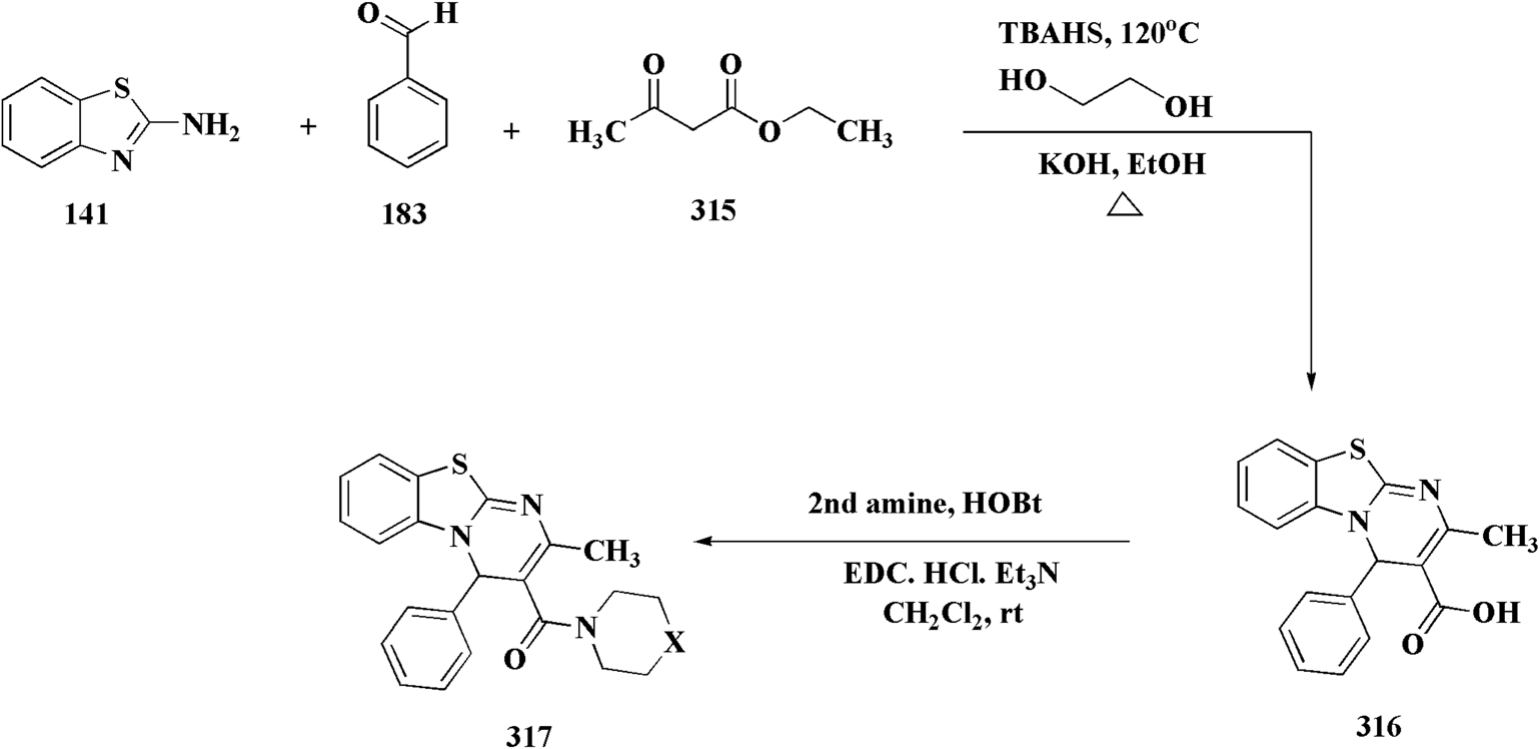
Synthesis of benzo[4,5]thiazolo[1,2-*a*]pyrimidines.

In continuation, the benzo[4,5]thiazolo[1,2-*a*]pyrimidine-3-carboxylate have been prepared as potent anti-cancer compounds. These compounds were synthesized from benzenecarboxaldehyde, 2-aminobenzothiazole and ethyl 3-oxobutanoate to afford compound 316 followed by the formation of amide through the reaction with different sec. amines (Scheme 60).

**Fig. 37:**
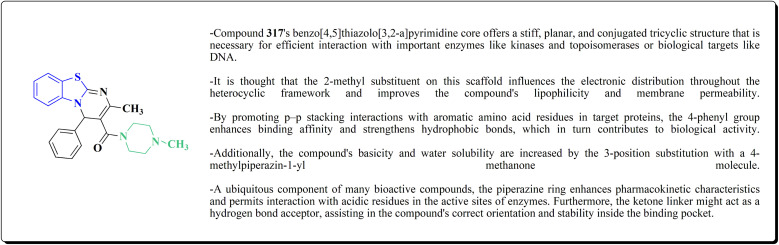
Structure–activity relationship of compound 317.

The cytotoxicity of these compounds was tested on a panel of human cancer cell lines *in vitro*. Compound 317b indicated promising cytotoxicity particularly against human breast adenocarcinoma cell lines, MDA-MB-231 & MCF-7, while compound 317a revealed promising cytotoxicity against MDA-MB-231 (Fig. 37).^211^

#### 8.4.2. Benzothiazoles fused with five-membered ring heterocycles

**Scheme 61:**
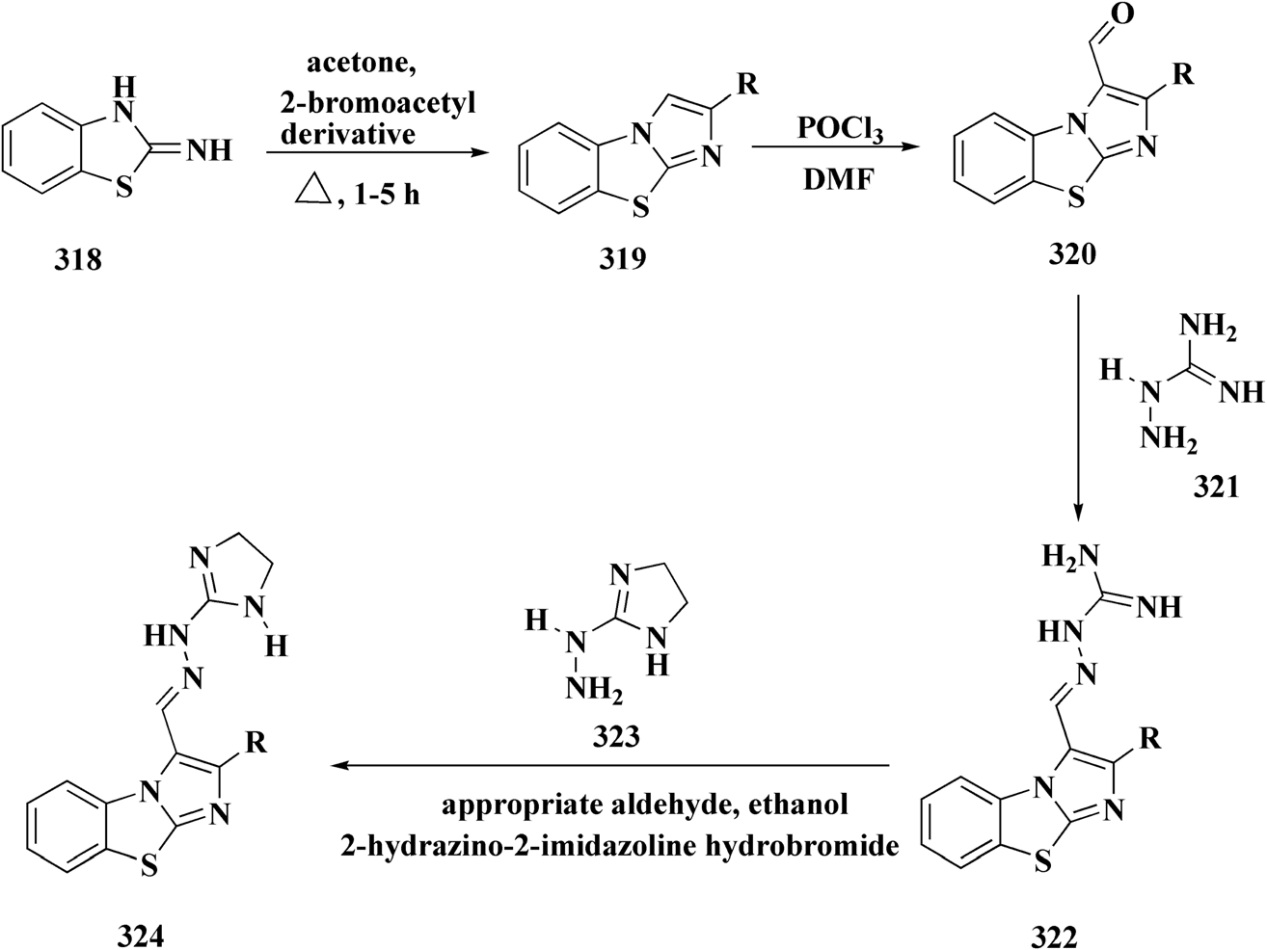
Synthesis of imidazo[2,1-*b*]thiazole guanylhydrazones.

The hydrazones 322a,b, 324a were synthesized by reaction between aminoguanidine or 2-hydrazino-2-imidazoline and an aldehyde. The aldehydes 320a,b were yielded by Vilsmeier reaction on compounds 319a,b synthesized from the bromoketones and the 2-amino-derivative (Scheme 61).

**Fig. 38:**
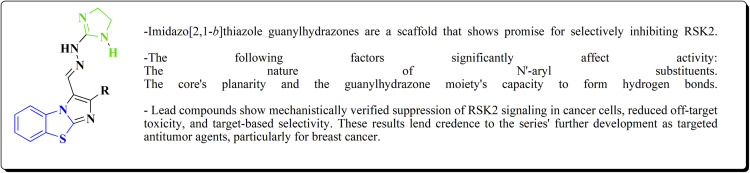
Structure–activity relationship of compound 324.

A determination is established regarding the imidazo[2,1-*b*]thiazole guanylhydrazones' potency to inhibit p90 ribosomal S6 kinase 2 (RSK2). It was found that subset of compounds shows both efficient *invitro* inhibition of tumor cell growth and RSK2 kinase activity. The compounds can affect the RSK2 target in cells (Fig. 38).^212^

**Scheme 62:**
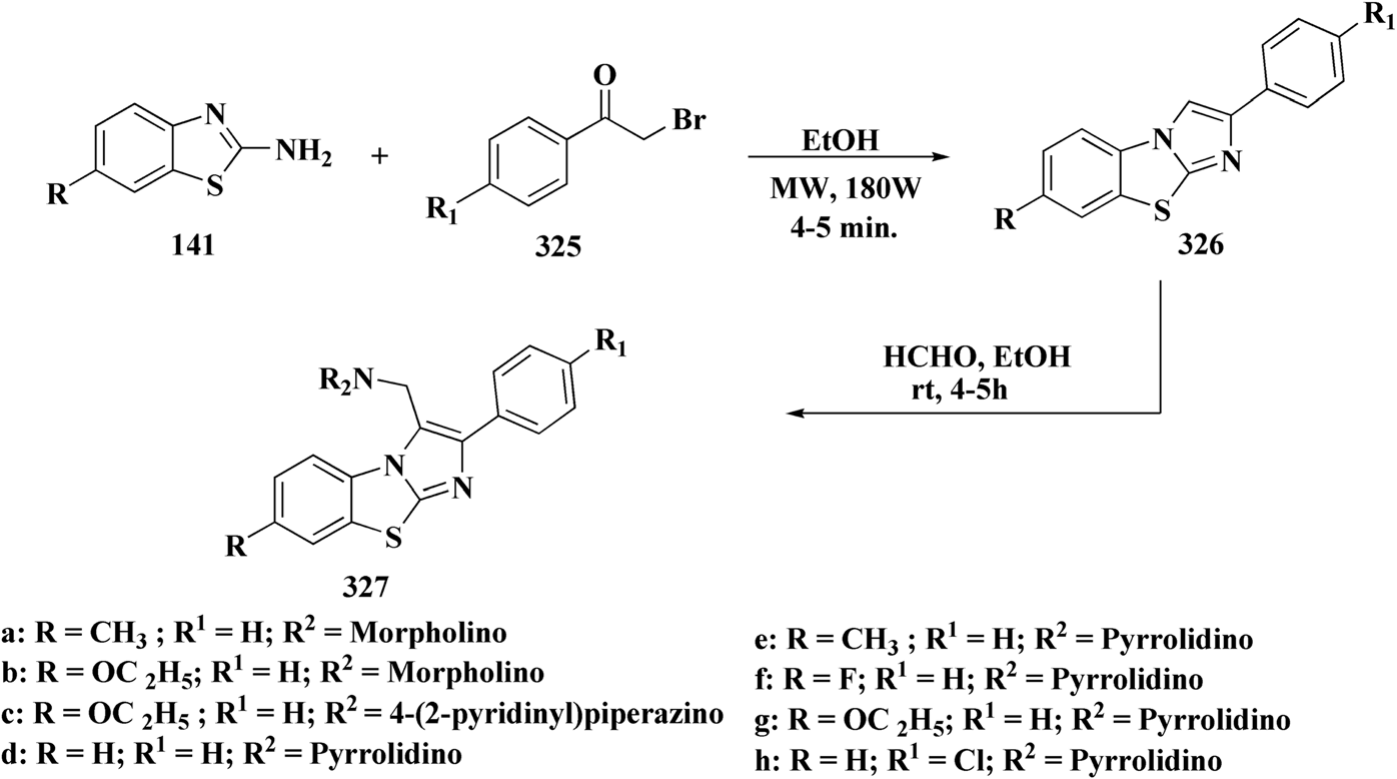
Synthesis of substituted imidazobenzothiazoles.

Substituted imidazobenzothiazole 327a–e were synthesized through the reaction of compound 141 with 2-bromoacetophenone 325 under microwave irradiations. Alkylation of compounds 326a–e through reacting with formic aldehyde and cyclic secondary amines afforded compounds 327a–h (Scheme 62).

The compounds were estimated for their anticancer potency. These compounds indicated better cytotoxic activity in MCF-7, HepG2, and HeLa cell lines. Additional mechanism aspects accountable for the anticancer activity of compounds 327c & 327f in HepG2 cell line were reported.^213^

Both 327c and 327f in the HepG2 cell line cause cell cycle arrest at the G_2_/M phase, which is accompanied by an upregulation of Chk2 and a downregulation of cyclin B, suggesting that the cell cycle is being interfered with. They cause apoptosis, which is the execution of programmed cell death, as shown by increased Caspase-3 levels.

**Fig. 39:**
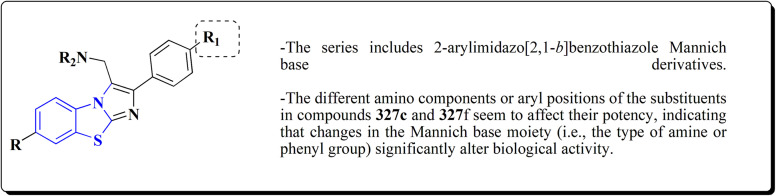
Structure–activity relationship of compound 327.

In addition, those compounds inhibit important signaling proteins linked to proliferation, including p38 MAPK, *p*-JNK, C-Jun, JunB (AP-1 factors), and PKCα, which inhibits growth and survival pathways. These findings emphasize 327f as a potent lead candidate for additional anti-cancer research (Fig. 39).^213^

**Scheme 63:**
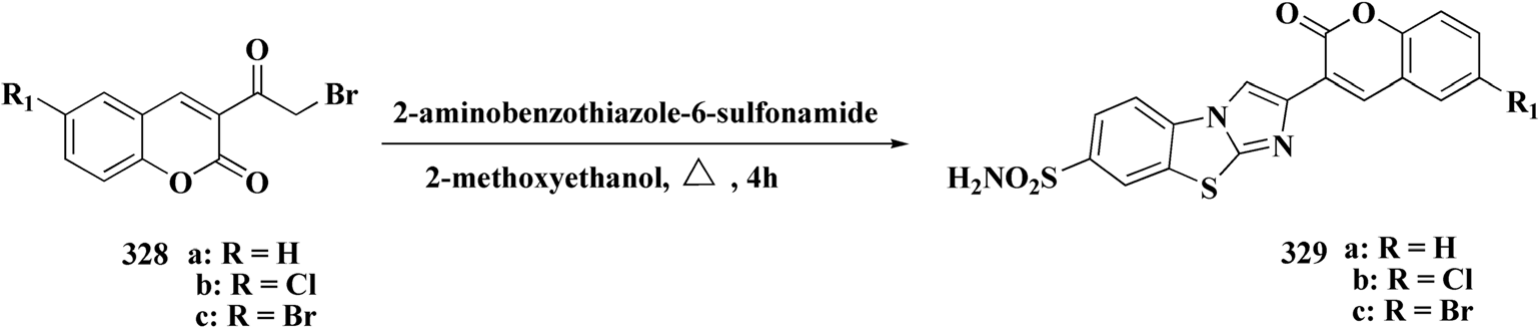
Synthesis of benothiaole–coumarin conjugates.

Heterocyclic series 329 consisting of three IBTs were prepared through the formation of 2-amino-benzothiazole-6-sulfonic acid amide from sulfanilamide and the subsequent reaction with 6-substituted-3-bromoacetylcoumarins (Scheme 63).

**Fig. 40:**
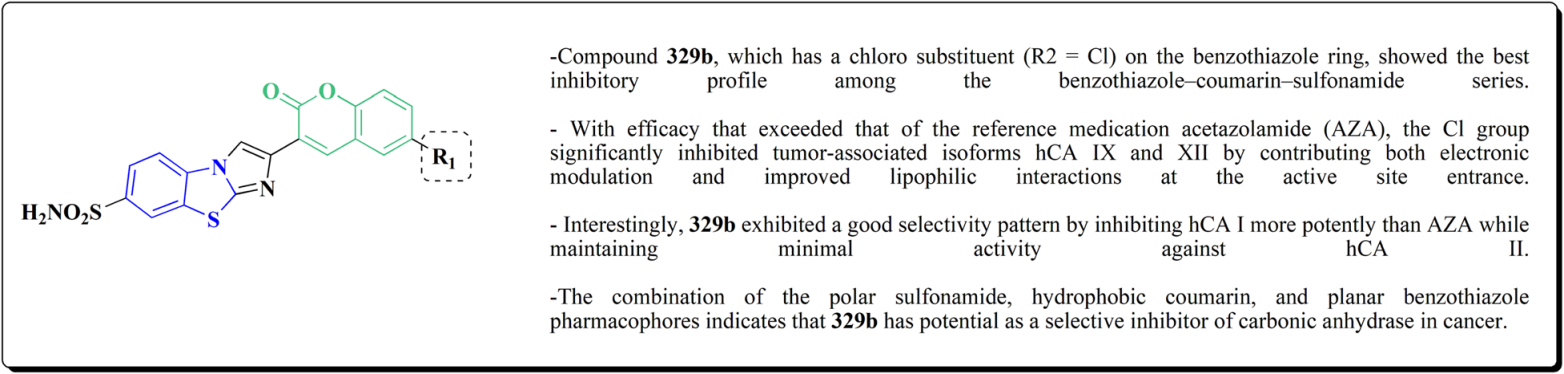
Structure–activity relationship of compound 329.

The inhibition potential of the compounds against the isoforms Hca I, II, IX & XII as selected human carbonic anhydrases was evaluated (Fig. 40). Results were compared with acetazolamide.^214^

**Scheme 64:**
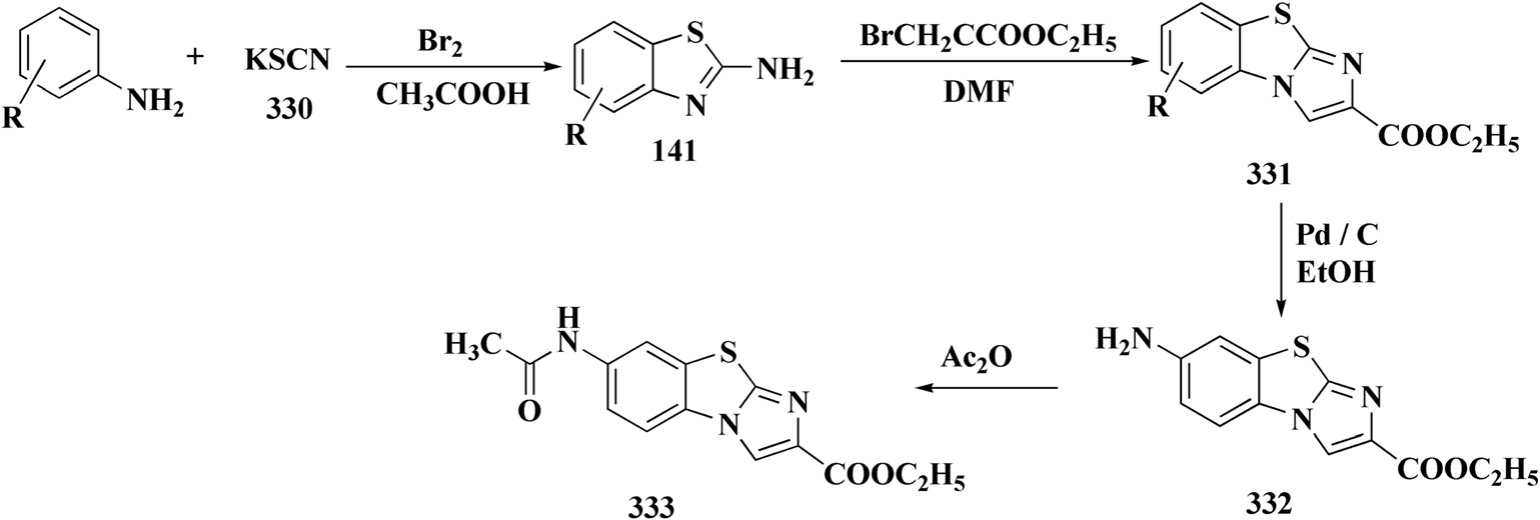
Synthesis of imidazobenzothiazoles.

Concurrently, the synthesis of the imidazobenzothiazoles 331 is outlined in Scheme 64. The 2-aminobenzothiazole was refluxed with ethyl bromopyruvate to afford hydrobromide as an intermediate which was subsequently cyclized to the targeted compounds. The thiocyanation reaction of 3,4-dichloroaniline allowed the synthesis of 5,6- and 6,7-dichloro-2-aminobenzothiazole. The amine 332 was acheived *via* catalytic hydrogenation of the nitrobenzothiazole. Treatment of 332 with acetic anhydride afforded the amide 333.

**Fig. 41:**
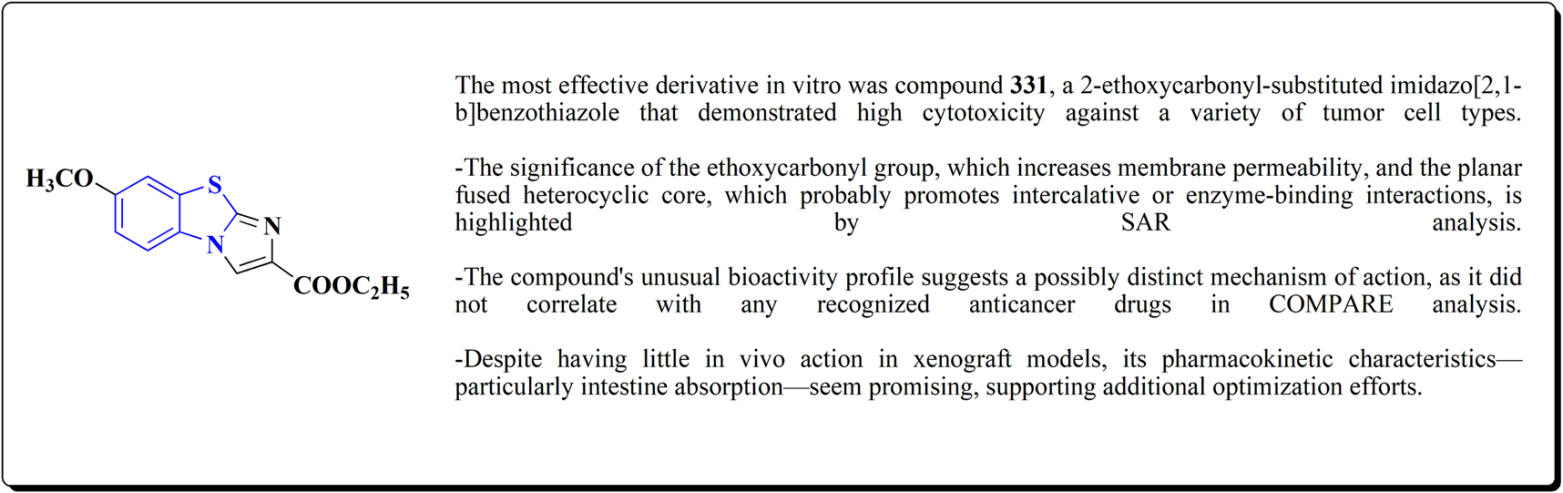
Structure–activity relationship of compound 331.

The imidazobenzothiazole compounds 331 were synthesized and their cytotoxic activity estimated for testing against tumor cell lines (Fig. 41). The most potent imidazobenzothiazole derivative was subsequently estimated as a cytotoxic agent using the hollow fiber assay.^215^

### 8.5. Benzothiazole complexes

**Scheme 65:**
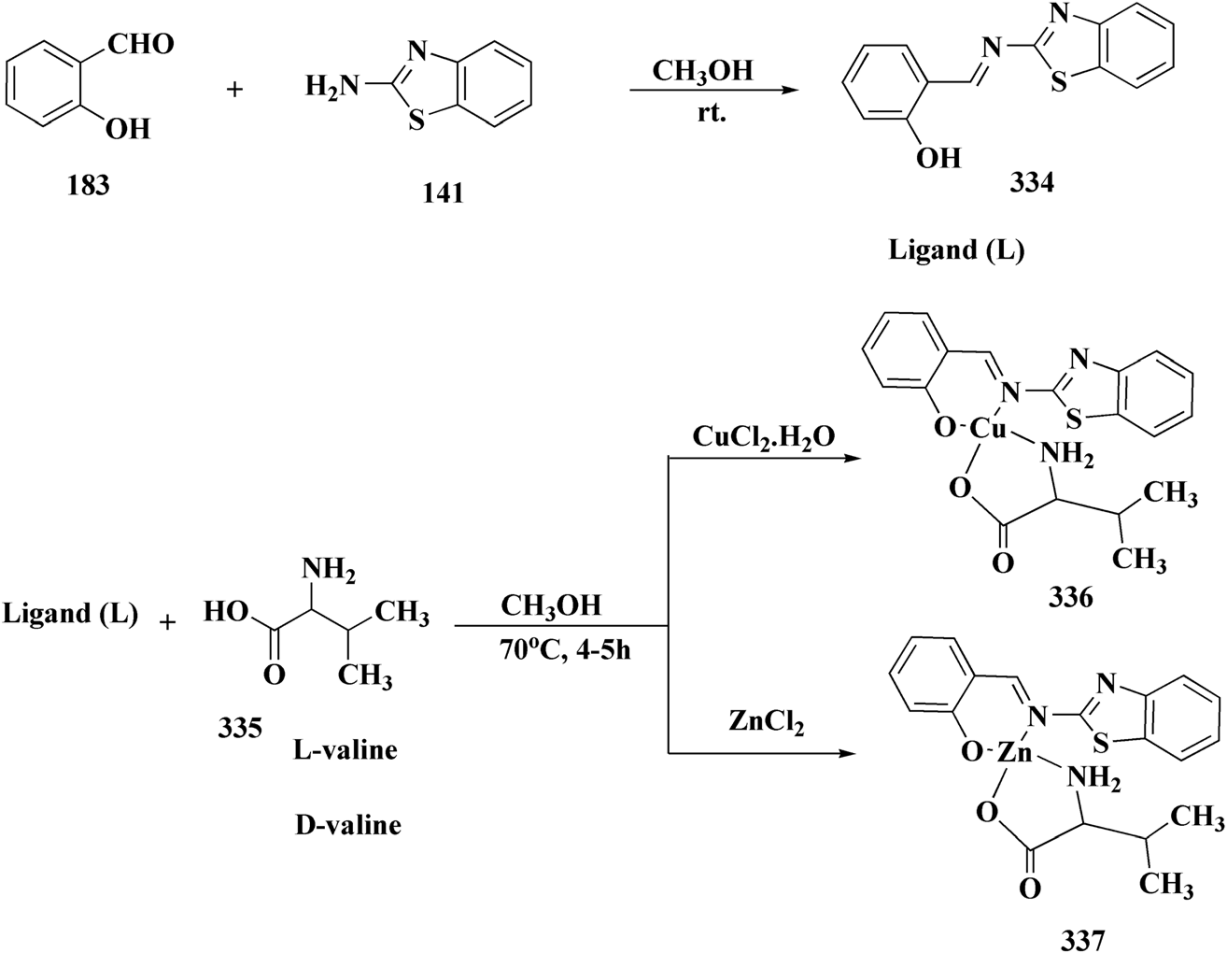
Synthesis of benzothiazole–valine complexes.

The synthesis of chiral antitumor chemotherapeutic agents of the benzothiazole Schiff base–valine complexes 336a & b and 337a & b were performed through reacting the stoichiometric amounts of the Schiff base ligand (l), Cu(ii)/Zn(ii) chloride & l-/d-valine (Scheme 65).^216^

**Fig. 42:**
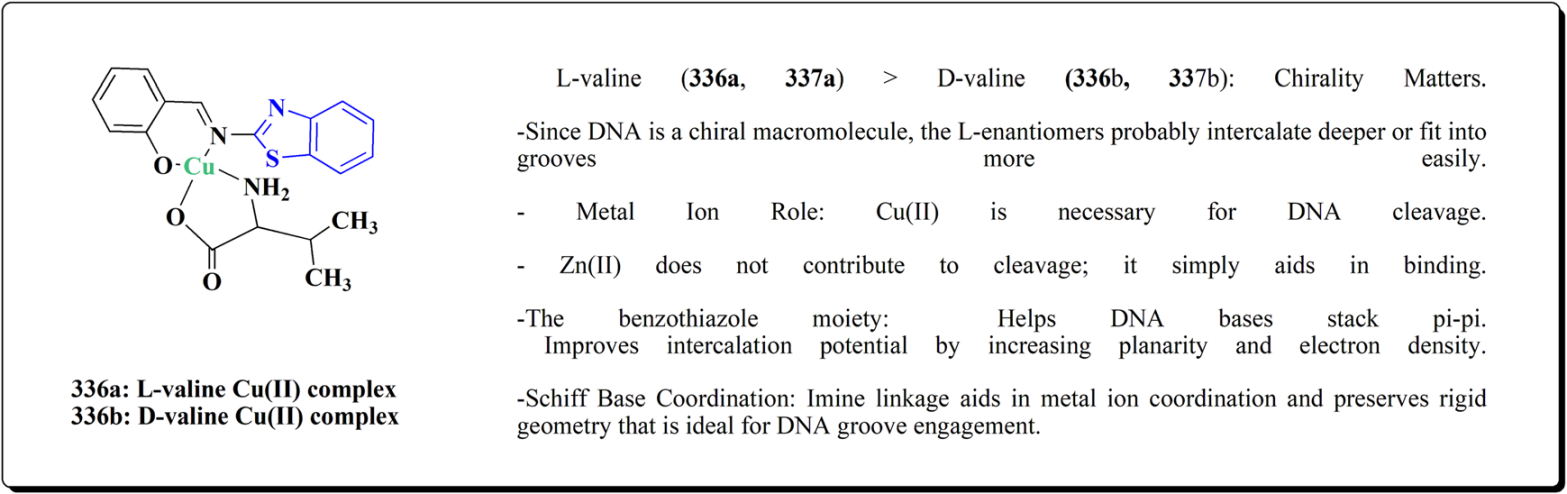
Structure–activity relationship of compound 336.

Towards DNA, the most effective and selective is 336a (Cu–l-valine complex). l-Forms are preferable, and chirality at the valine residue has a significant impact on biological behavior. Regarding DNA binding and cleavage efficacy, the SAR trend is 336a > 336b > 337a > 337b (Fig. 42). These findings encourage the development of chiral benzothiazole–metal complexes as DNA-targeted antitumor drugs, with a focus on metal redox characteristics and stereochemistry.^216^

**Scheme 66:**
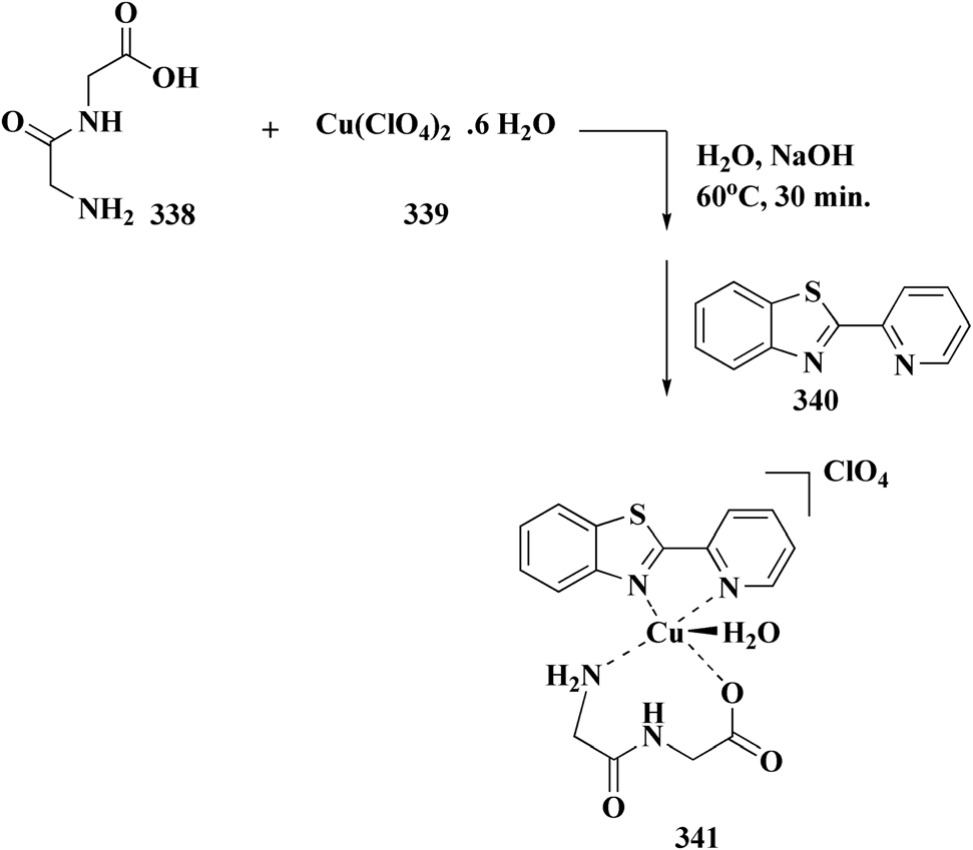
Synthesis of Cu(ii) complex 341.

In continuation, the Cu(ii) complex was synthesized as outlined in Scheme 66 through the reaction of diglycine with copper(ii) perchlorate with pbt as a secondary ligand. The complex was exposed to cytotoxicity tests *in vitro* utilizing human cancer cells lines and indicated prominent and selective cytotoxicity against HepG2 cell lines with IC_50_ value 17.78 μm.^217^

**Fig. 43:**
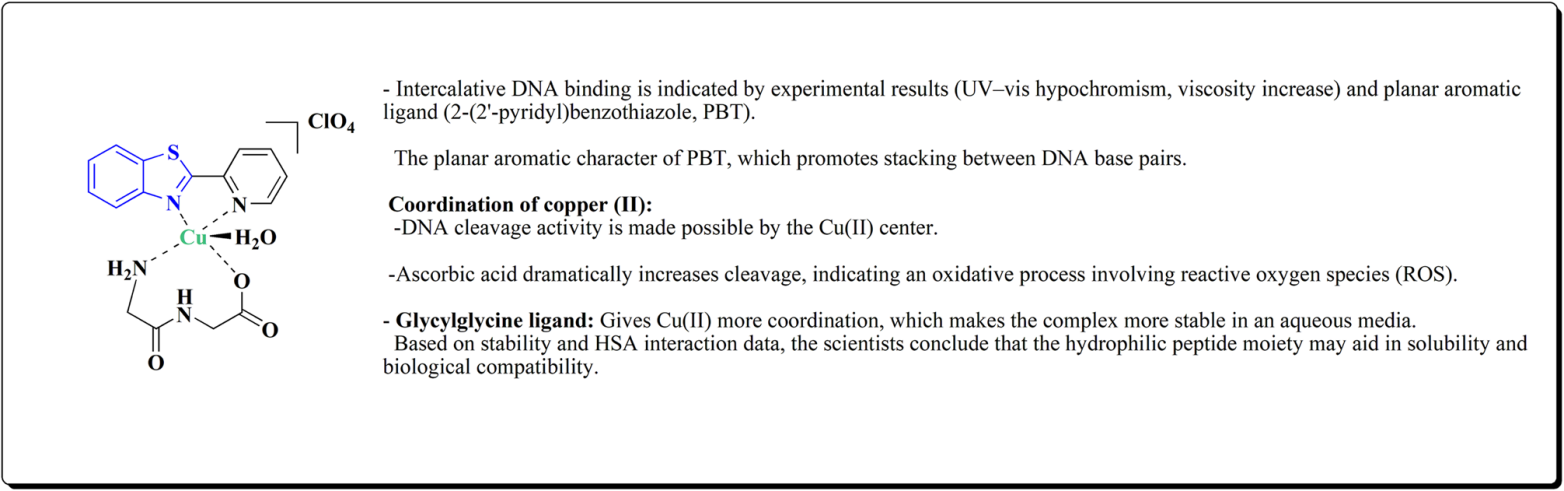
Structure–activity relationship of compound 341.

Interaction of Human Serum Albumin (HSA) parameters is reported; UV-vis shifts and fluorescence quenching demonstrate the complex's strong noncovalent bond with HSA. In biological systems, this interaction might have an impact on bioavailability and transport. The complex's quantifiable cytotoxicity is attributed to its capacity for DNA binding and cleavage (Fig. 43).^217^

## 9. Synthetic strategies for novel anti-microbial benzothiazoles

### 9.1. Benzothiazoles linked with heterocyclic compounds

#### 9.1.1. Pyrimidine–benzothiazole based analogues

**Scheme 67:**
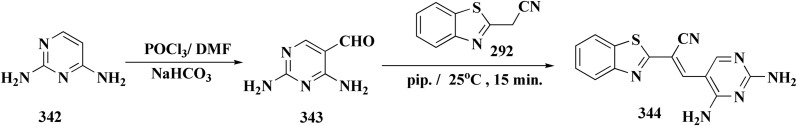
Synthesis of pyrimidine–benzothiazole conjugates.

The synthesis of compounds 344 in which a pyrimidine ring was linked to the benzothiazole moieties through an acrylonitrile bridge, was performed as outlined in Scheme 67. The synthesis of benzazole pyrimidine acrylonitriles 344 were reported. The condensation of 2-benzazole acrylonitriles 292 and pyrimidine aldehydes 343 yielded compounds 344.

**Fig. 44:**
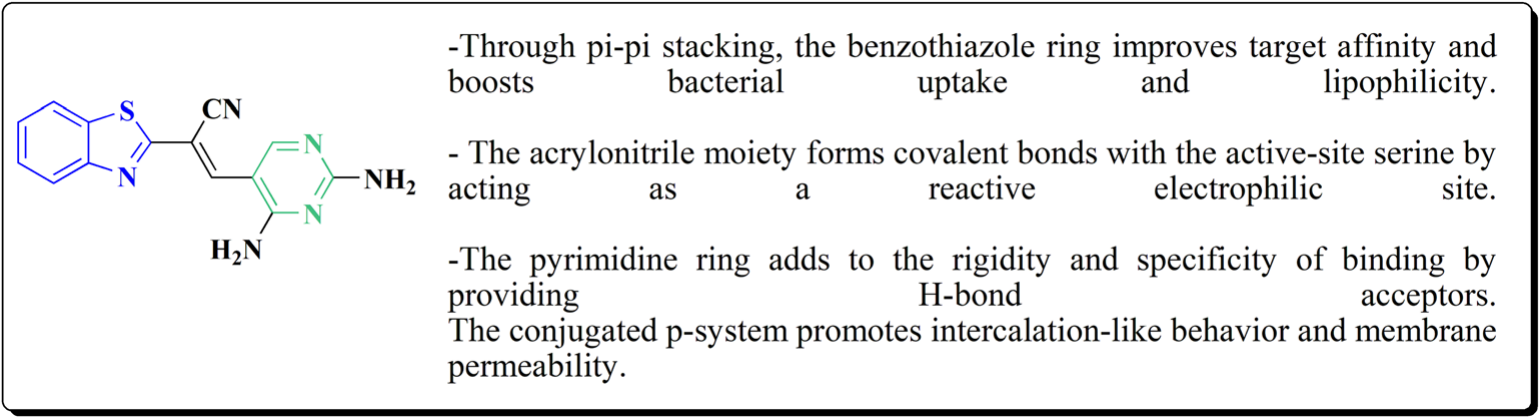
Structure–activity relationship of compound 344.

The antibacterial activity of the novel compounds was tested against bacterial strains. Compound 344 was found to display the high potency against both *Pseudomonas aeruginosa* and *Escherichia coli* as comparable to amoxicillin (Fig. 44).^218^

Compound 344 is structurally suitable for covalent inhibition and high affinity binding; it is able to target two enzymes (PBP and β-lactamase); and able to overcome resistance through a synergistic mechanism with amoxicillin 218.

**Scheme 68:**
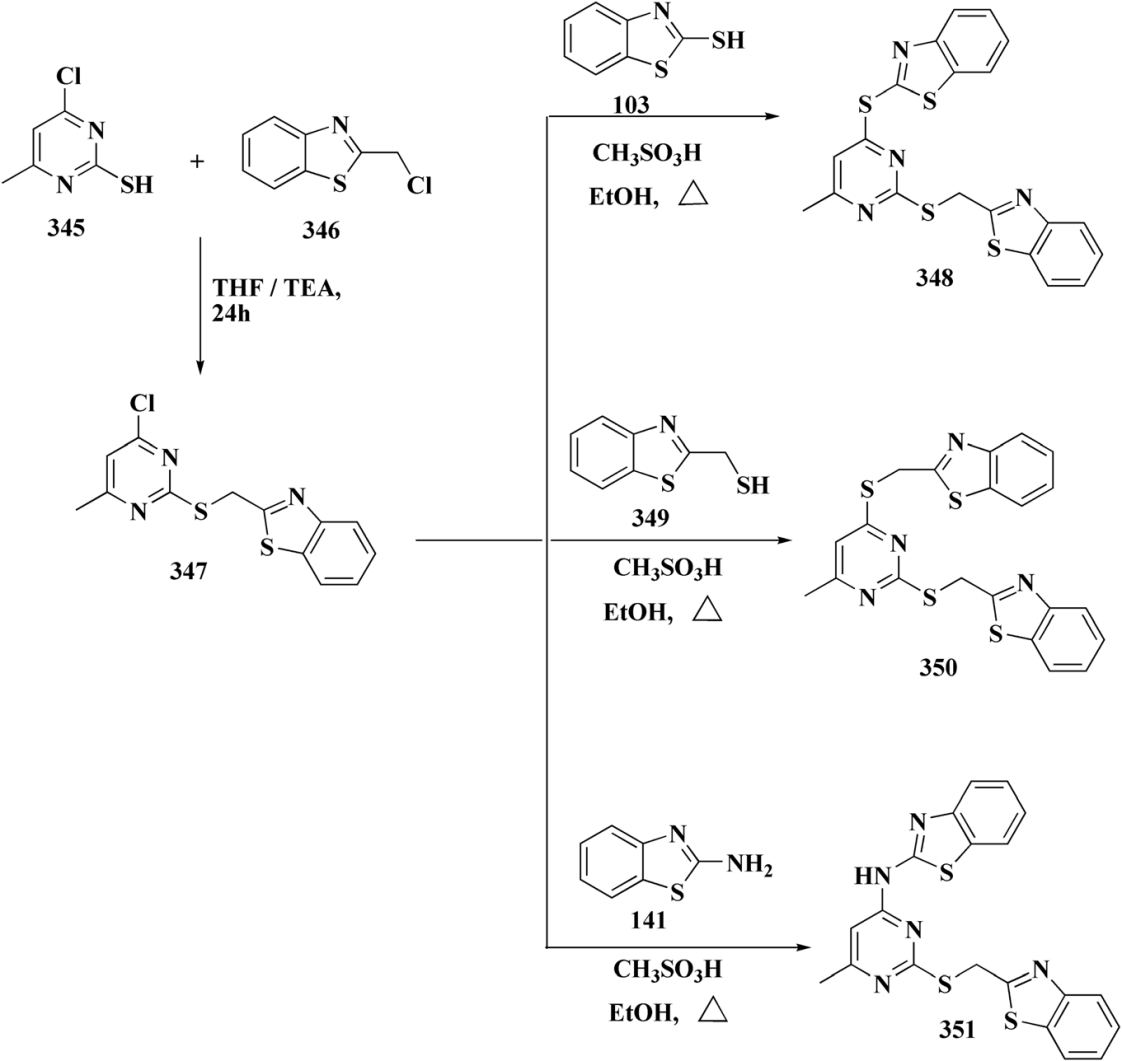
Synthesis of pyrimidine–benzothiazole conjugates.

**Fig. 45:**
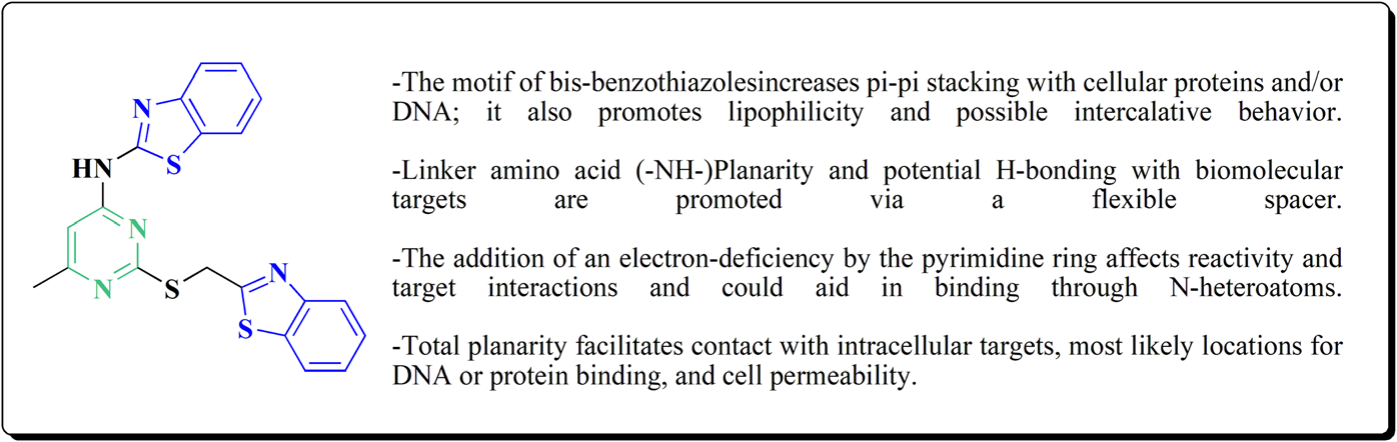
Structure–activity relationship of compound 351.

Compounds 347 were synthesized by treating 345 with 346. Similarly, the compounds 348 were synthesized by performing the reaction of 347 with 103. Compounds 350 were synthesized by treating 347 with 349. Compound 351 were furnished from the reaction of 347 with 141 (Scheme 68). The amino linked pyrimidinyl bis benzothiazole 351 displayed cytotoxic potency on A549 cells (IC_50_ value = 10.5 Mm) (Fig. 45).^219^

**Scheme 69:**
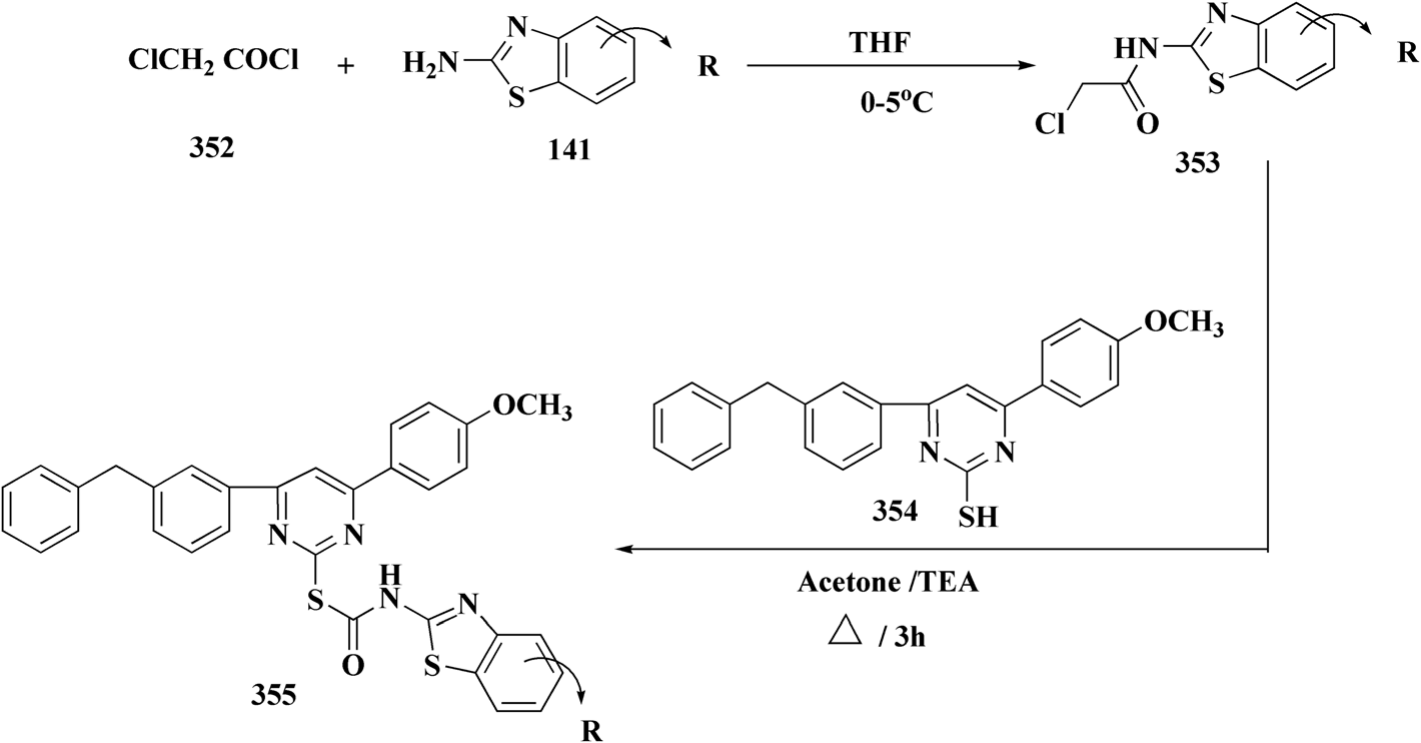
Synthesis of pyrimidine based benzothiazole derivatives.

**Fig. 46:**
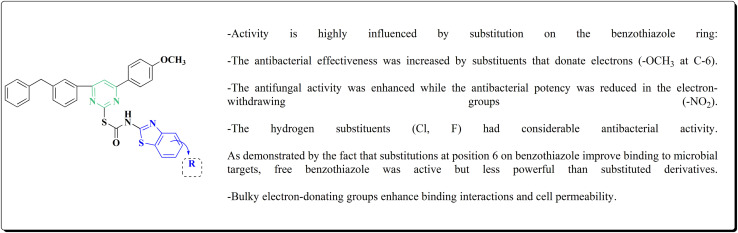
Structure–activity relationship of compound 355.

Pyrimidines are crucial core in synthesizing numerous heterocycles with wide applications.^220–223^ Pyrimidine based benzothiazole derivatives were synthesized as shown in Scheme 69. The Chalcone is generated by an aldol, condensation of *m*-phenoxy benzaldehydes with 4-methoxy acetophenone using a catalyst that is treated with thiocarbamide to furnish substituted pyrimidine. The pyrimidine treated with substituted *N*-1,3-benzothiazole-2-yl-2-chloroamide affords compound 355 (Fig. 46).^224^

The synthesized compounds screened against two Gram +ve bacteria and two Gram −ve bacteria, analyzed with chloramphenicol, ampicillin, ciprofloxacin, and norfloxacin. The synthesized compounds were screened against the anti-fungal strains *Aspergillus niger* and *Candida albicans* organisms, and were compared with griseofulvin and nystatin as standard drugs.^224^

*N*-(6-Methoxybenzo[*d*]thiazol-2-yl)-2-(4-(4-methoxyphenyl)-6-(3-phenoxyphenyl)pyrimidin-2-ylthio)acetamide indicated excellent anti-microbial potency against both Gram-positive and Gram-negative bacteria. It is comparable to ciprofloxacin for Gram-(−ve) strains. It showed less activity in anti-fungal screening, but still moderate against *Aspergillus niger*.

#### 9.1.2. Deazapyrimidine–benzothiazole based analogues

**Scheme 70:**
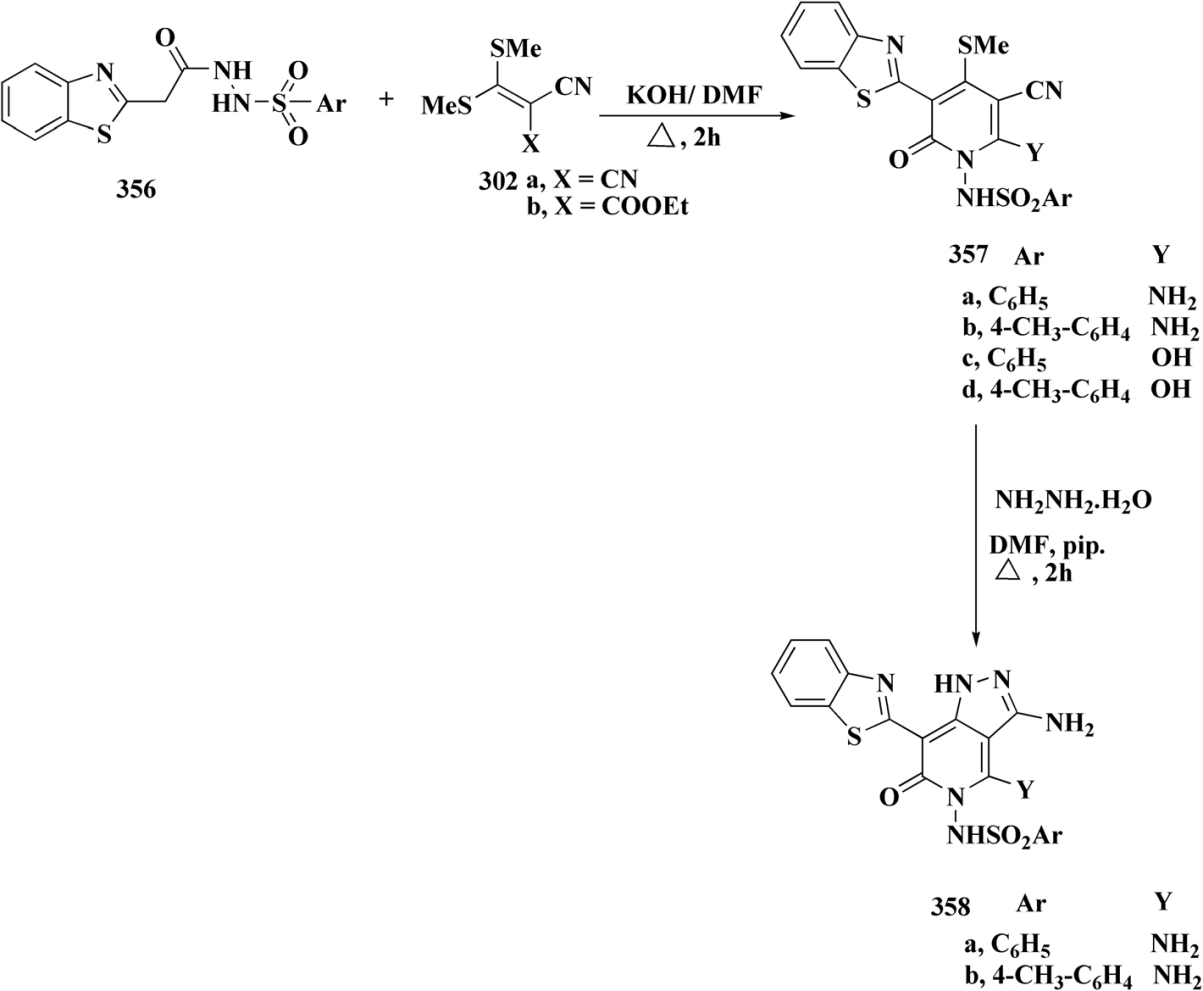
Synthesis of *N*-sulfonamide 2-pyridone derivatives.

Novel benzothiazole sulfonylhydrazide was reacted with ketene dithioacetal derivatives to synthesize a new series of *N*-sulfonamide 2-pyridone derivatives (Scheme 70). These substances were tested for their *in vitro* antimicrobial efficiency and capacity to concurrently inhibit both targets. They were synthesized to combine dual inhibitory action against both DHPS and DHFR enzymes within a single molecule.

**Fig. 47:**
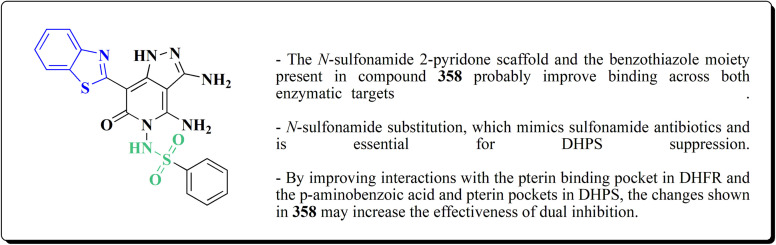
Structure–activity relationship of compound 358.

Five compounds – 356b, 357a, 357b, 358a, and 358b—of the synthesized *N*-sulfonamide 2-pyridone derivatives showed significant antibacterial activity against the tested strains of bacteria and fungi. The MTT assay was used to further assess these compounds' cytotoxicity on the human dermal fibroblast cell line HFB4. With IC_50_ values of 2.76 and 0.20 μg mL^−1^, respectively, compound 358a was shown to be the most potent dual inhibitor of DHPS and DHFR in *in vitro* enzyme inhibition tests. According to molecular docking studies, 358a efficiently resides in the pterin binding pocket of DHFR as well as the *p*-aminobenzoic acid and pterin binding sites of DHPS. These results validate that the most effective dual DHPS/DHFR inhibitor in this series is 358a (Fig. 47). By reacting newly synthesized benzothiazole sulfonylhydrazide compounds with ketene dithioacetal derivatives, the novel *N*-sulfonamide 2-pyridone derivatives containing a benzothiazole moiety were synthesized.^225^

The most effective dual inhibitor of both the DHPS and DHFR enzymes among these was compound 358, which had unusually low IC_50_ values of 2.76 μg mL^−1^ for DHPS and 0.20 μg mL^−1^ for DHFR.

Compound 358 efficiently occupies crucial binding sites, according to docking analyses: DHPS – it interacts with the binding pockets of pterin and *p*-aminobenzoic acid. The pterin binding pocket is where DHFR binds. Dual-site occupancy is suggested by these interactions, improving inhibitory efficacy and specificity. From a structural standpoint, the benzothiazole and *N*-sulfonamide 2-pyridone core probably provide for flexible fitting in the enzyme active sites, promoting robust binding interactions.^225^

**Scheme 71:**
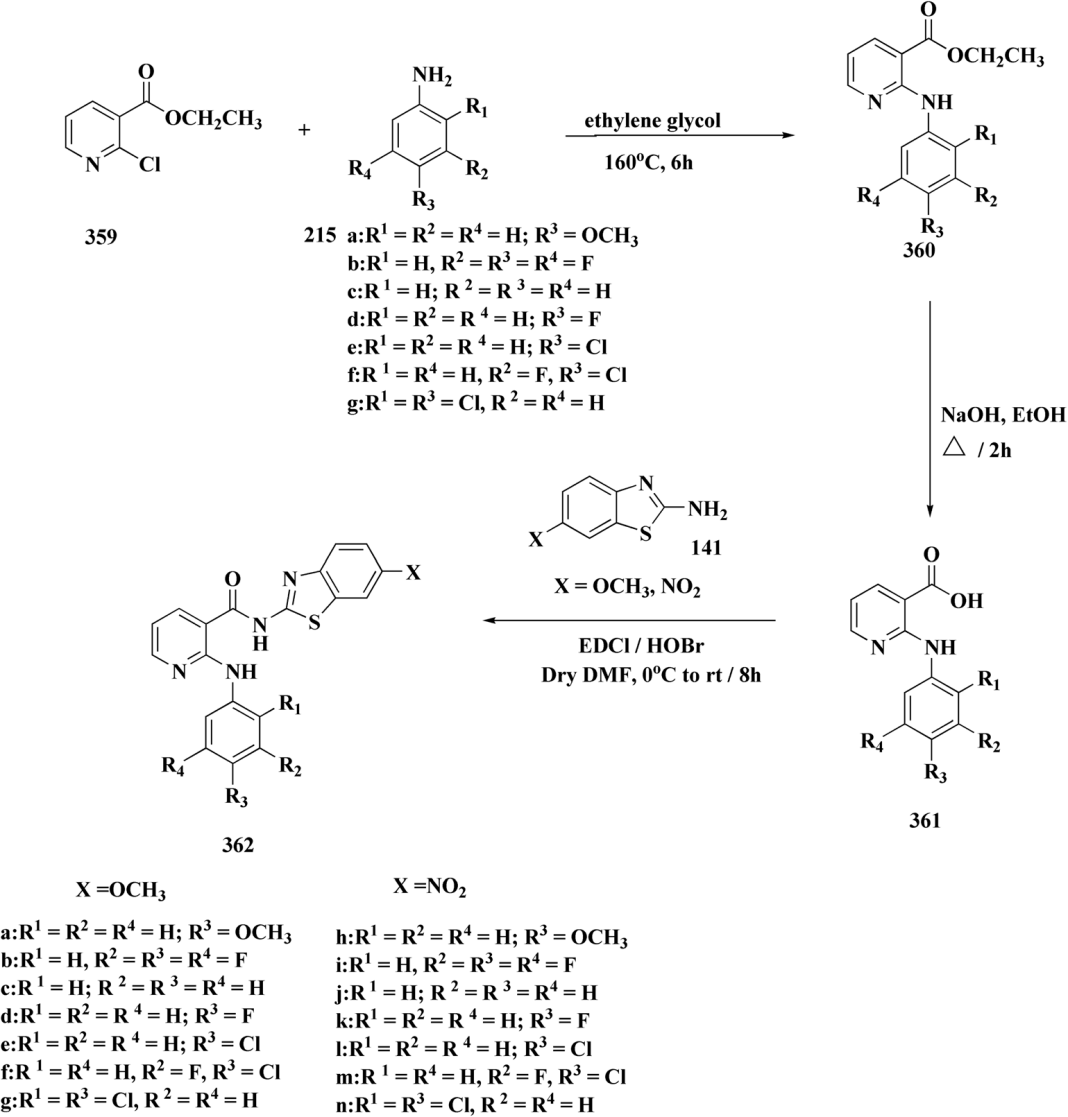
Synthesis of Synthesis of pyridine based benzothiazole derivatives.

2-Chloropyridine-3-carboxylic acid 359 and the anilines 215a–g were reacted under reflux in ethane-1,2-diol to yield compound 360a–g. Further treatment of the latter with sodium hydroxide solution give the 2-anilino nicotinic acids 361a–g. The synthesis of compounds 362a–n was performed by reacting 6-substituted 2-amino benzothiazoles with 2-anilino nicotinic acids (Scheme 71).^226^

**Fig. 48:**
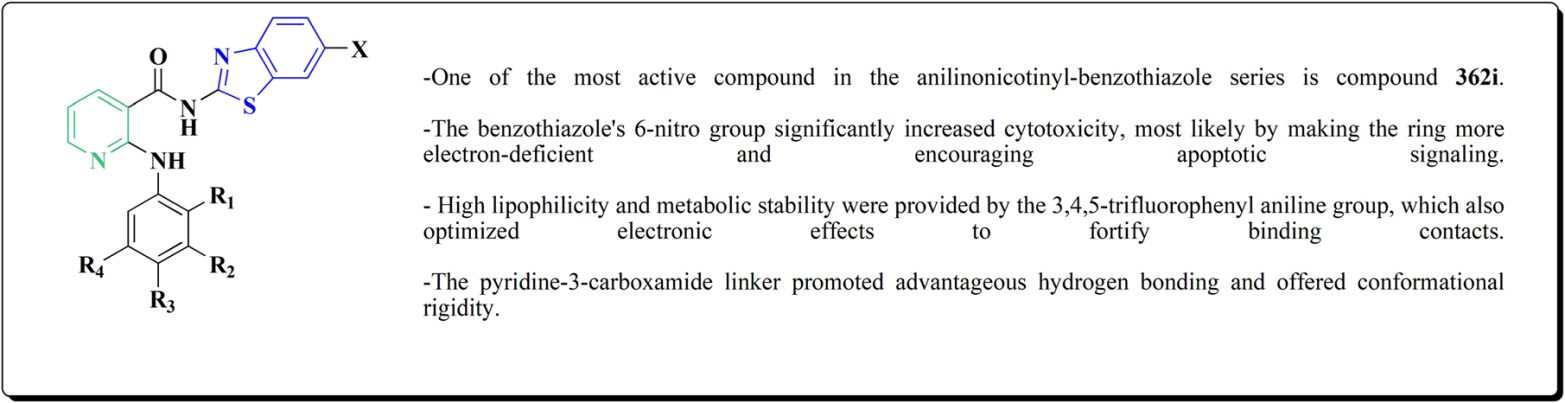
Structure–activity relationship of compound 362.

The compounds were estimated for their antiproliferative activity. Compounds (362h–k & 362n) have displayed potent cytotoxicity against human leukemia HL-60 cell lines. All these compounds were examined for their effects on the cell cycle perturbations and induction of apoptosis. Subsequently, the mechanism of cell death was analysed. The cytotoxicity of 362i (Fig. 48) correlated with induction of apoptosis, caspases activation and DNA damage and thus showing the apoptotic pathway of anticancer effect of these compounds.^226^

In terms of mechanism, 362i caused apoptosis through the extrinsic and intrinsic (mitochondrial) pathways. Nuclear DNA damage was the main driver for the dose-dependent activation of caspases-3, -8, and -9, which suggested dual apoptotic pathway engagement. Additionally, without influencing Bcl-2 expression, the drug increased loss of mitochondrial membrane potential, ruling out direct Bcl-2 regulation as a mechanism. Crucially, there was no rise in nitric oxide levels, indicating different processes that trigger apoptosis. Fluorescence microscopy morphological study verified the characteristic apoptotic alterations, such as decreased cell size, smoothing of the plasma membrane, and widespread blebbing.^226^

**Scheme 72:**
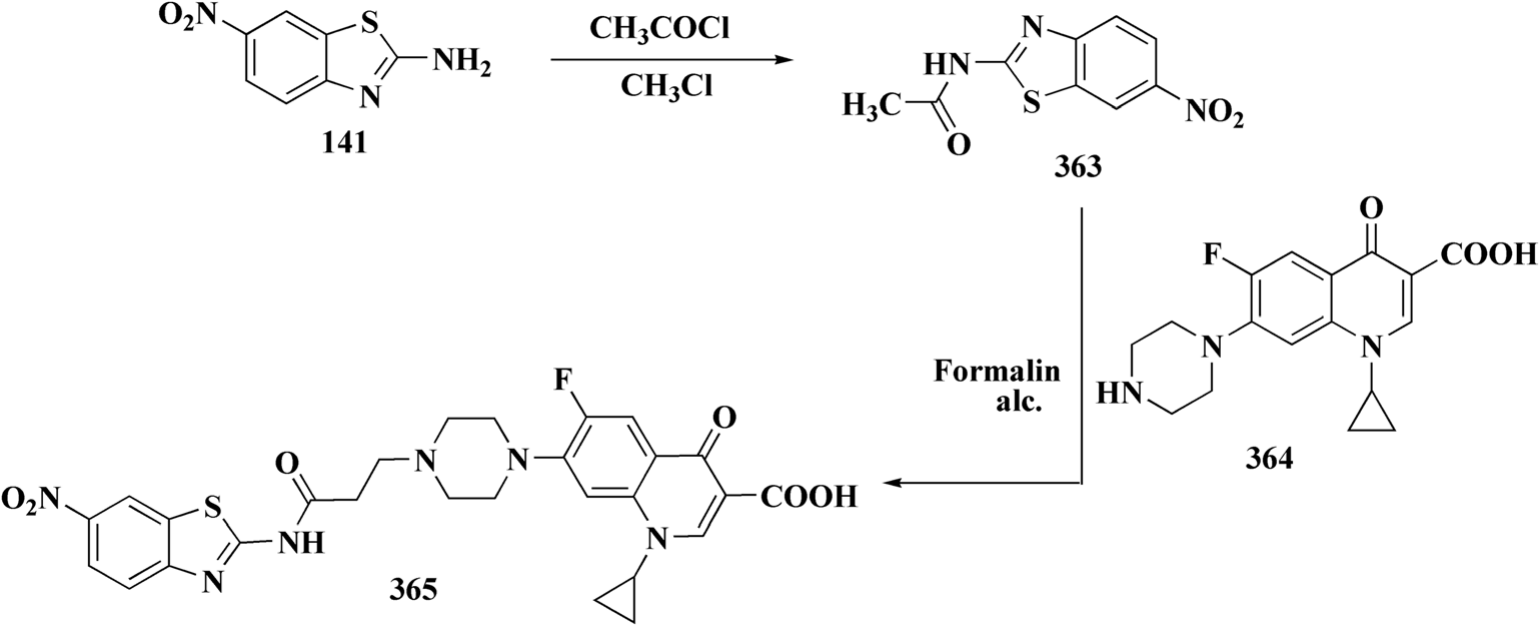
Synthesis of *N*-Mannich base pro-drugs of norfloxacin.

In another approach, the benzothiazoles 334 were acetylated *via* refluxing them with acetyl chloride to afford acetylated benzothiazoles 335. *N*-Mannich bases of the anti-bacterial agents 337 were prepared through refluxing compound 335, antibacterial agents 336, and formalin (Scheme 72). The compounds have been screened for antihelmintic activity and cytotoxic activity against human lung cancer cell lines A-549. Prodrug 337 (GI_50_ value 28.8) has been proved most potent among all the synthesized prodrugs.^227^

**Fig. 49:**
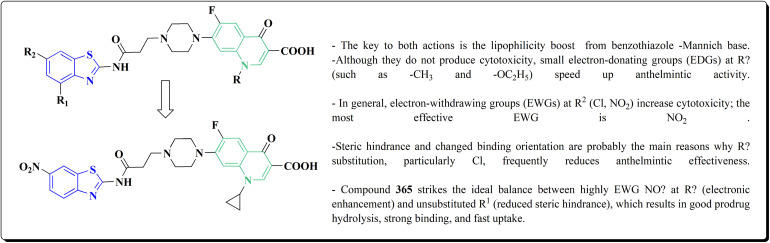
Structure–activity relationship of compound 365.

Compound 365 is a derivative of benzothiazole and norfloxacin *N*-Mannich base. The benzothiazole ring in 365 (Fig. 49) has a strong electron-withdrawing NO_2_ group at position 6 (R_2_) and is unsubstituted at R_1_.

The norfloxacin amine and the benzothiazole carrier are joined by the *N*-Mannich base. This raises the amine's lipophilicity and makes passive diffusion across membranes easier by lowering its p*K*_a_ by about three units. Under physiological conditions, the parent drug (norfloxacin) and the benzothiazole moiety are released when the Mannich base hydrolyzes (pH-dependent).

Prior to hydrolysis, the intact benzothiazole–fluoroquinolone combination may interact with targets such as cancer cells or parasites. The benzothiazole fragment can offer further bioactivity (benzothiazoles are known anticancer/antimicrobial scaffolds), while hydrolysis releases norfloxacin, which maintains antimicrobial/anthelmintic activity.

In terms of cytotoxicity, the enhanced lipophilicity improves cellular absorption in A-549 cells; the strong growth inhibition may be explained by the NO_2_ group increasing enzyme inhibition or DNA intercalation potential.^227^

**Scheme 73:**
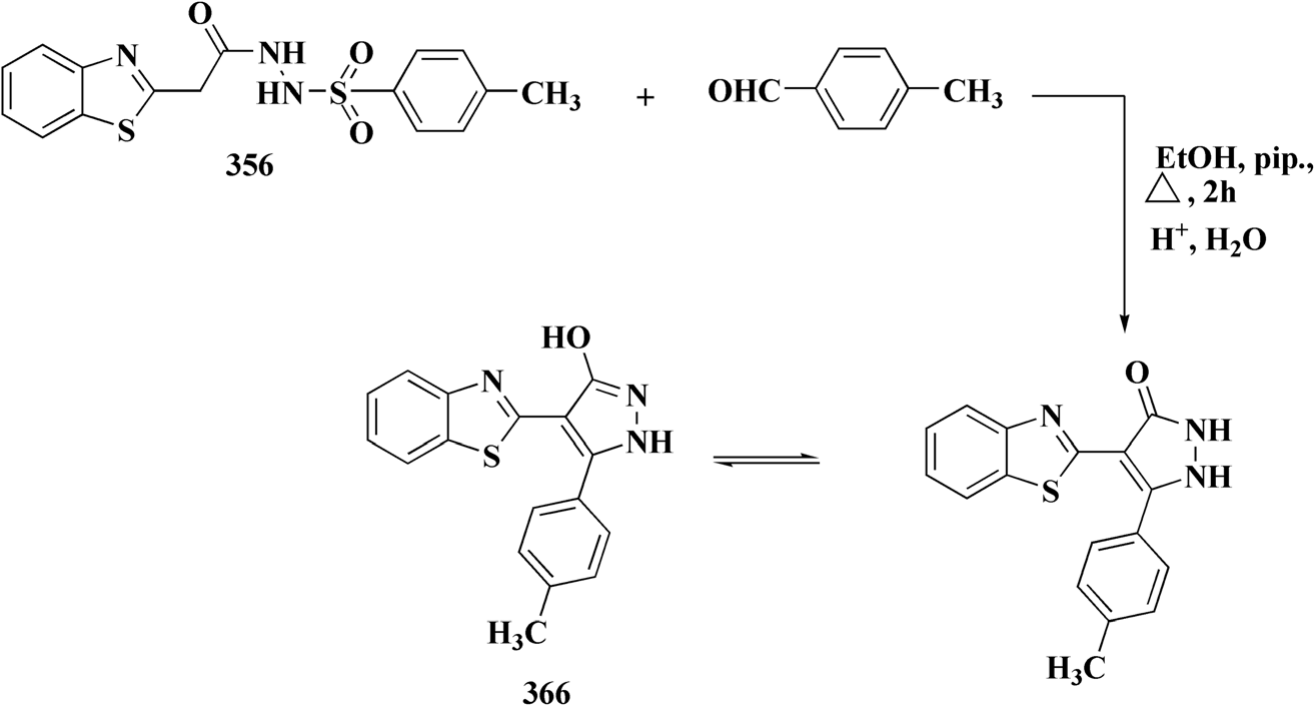
Synthesis of substituted 4-(benzo[*d*]thiazole-2-yl)-1*H*-pyrazol-3(2*H*)-one.

The ability of a number of recently synthesized benzothiazole derivatives to inhibit the dihydropteroate synthase (DHPS) enzyme and exhibit antibacterial activity was assessed. Benzothiazole *N*-arylsulfonylhydrazones were synthesized by reacting with a variety of partners, such as arylaldehydes, diazonium salts of arylamine derivatives, *N*-aryl-2-cyano-3-(dimethylamino)acrylamide, and *N*-aryl-3-(dimethylamino)prop-2-en-1-one (Scheme 73). With minimum inhibitory concentrations (MICs) ranging from 0.025 to 2.609 mM, antimicrobial screening showed that many of the synthesized compounds demonstrated substantial action, especially against *Staphylococcus aureus*. With a MIC of 0.025 mM, compound 366 was the most potent of them all, outperforming common antibiotics like ampicillin and sulfadiazine.^228^

**Fig. 50:**
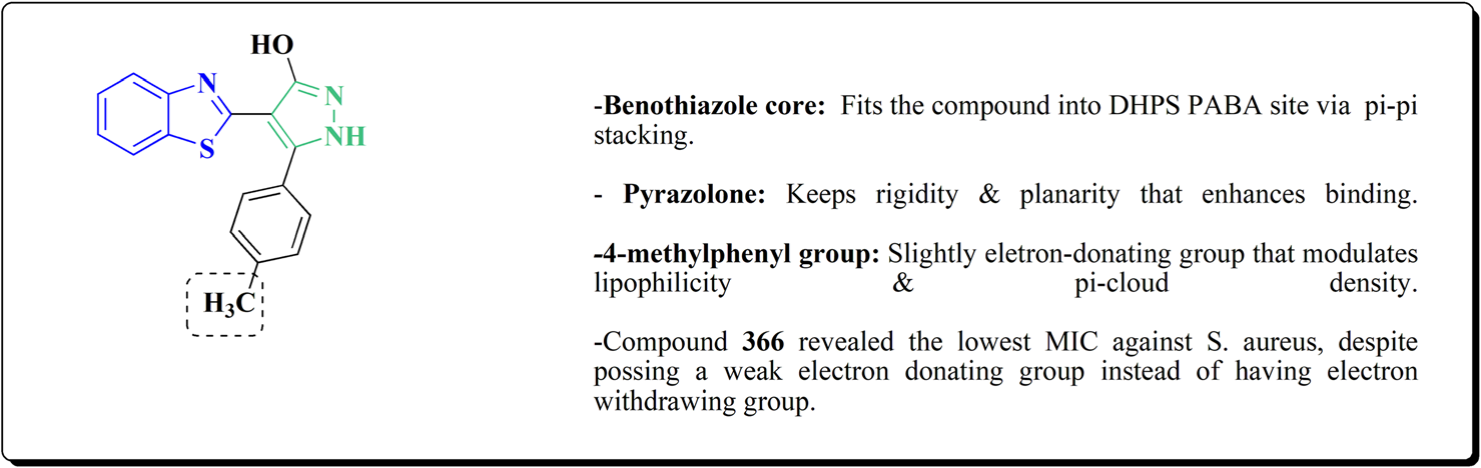
Structure–activity relationship of compound 366.

The benzothiazole–pyrazolone hybrids exert their antibacterial activity primarily through inhibition of the dihydropteroate synthase (DHPS) enzyme, a key player in the folate biosynthesis pathway in bacteria. These compounds mimic the natural substrate *para*-aminobenzoic acid (PABA) and competitively bind to the PABA-binding pocket of DHPS, thereby blocking the formation of dihydropteroate and ultimately halting DNA synthesis. The planar benzothiazole and pyrazolone moieties facilitate strong π–π stacking interactions with aromatic residues within the active site, while the sulfonylhydrazone linker provides critical hydrogen bonding interactions. Specifically, compound 366 (Fig. 50), which features a *para*-methyl-substituted phenyl group, benefits from optimal hydrophobic interactions and enhanced membrane permeability. Despite lacking a strong electron-withdrawing group, its structural balance allows efficient binding within the DHPS active site, leading to superior antibacterial potency compared to standard drugs.

#### 9.1.3. Triazole–benzothiazole based analogues

**Scheme 74:**
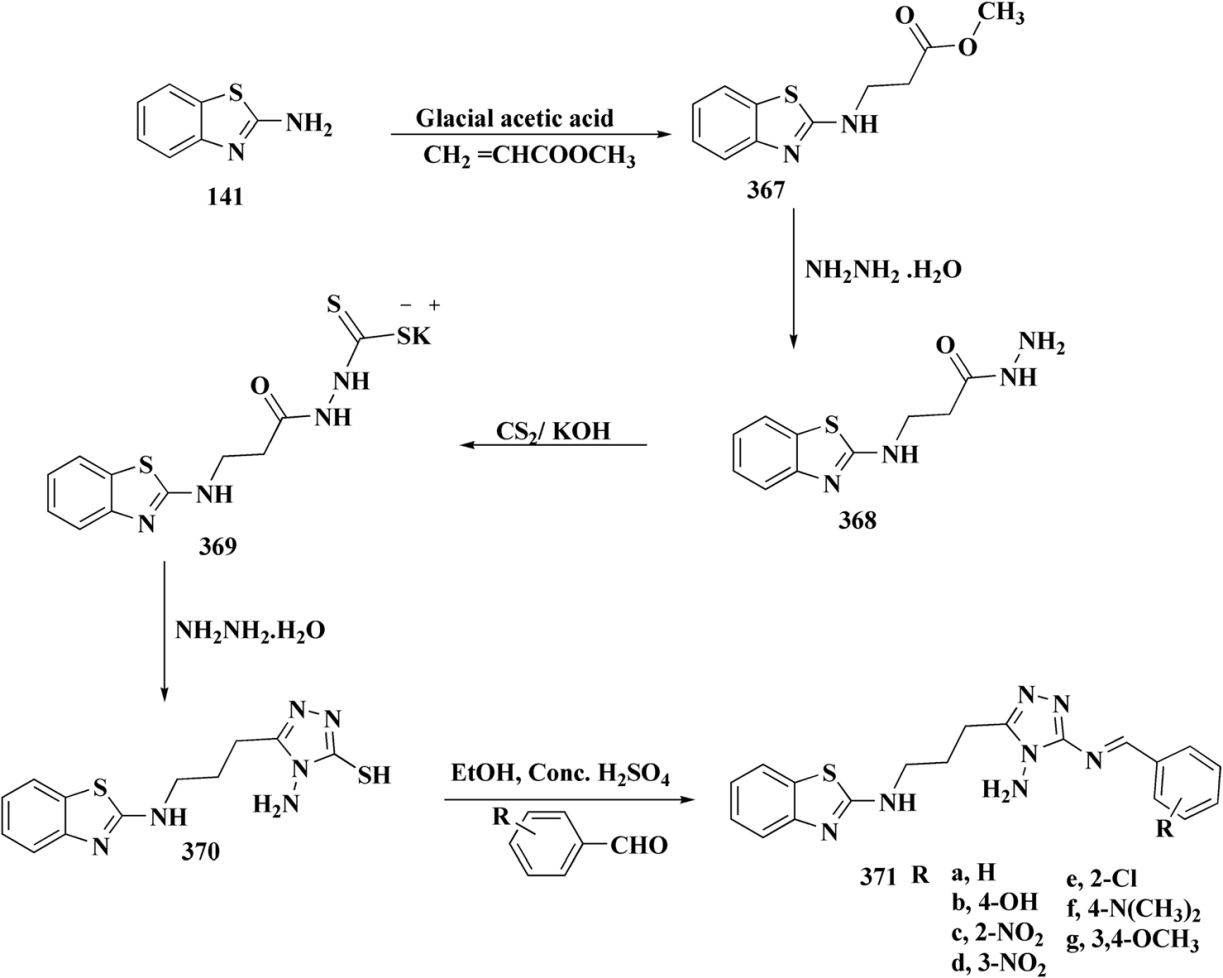
Synthesis of 1,2,4-triazoles benothiazole conjugates.

The reaction of compound 370 with aromatic aldehydes yielded the 1,2,4-triazoles 371a–g (Scheme 74).^231^ Triazoles showed potency in a variety of synthesized compounds.^229,230^ The synthesized compounds were screened for their antimicrobial activity.^231^

It was suggested that the *para*-substitution increases π-delocalization by enabling better electrical conjugation between the aromatic ring and the azomethine (–CH=N–) connection. Stronger π–π stacking or hydrophobic interactions with microbial enzymes or DNA may be made possible by this increased planarity. The arylidene ring was twisted out of plane by *o*-substitution (371c: 2-NO_2_; 371e: 2-Cl), which decreased conjugation and binding affinity.

**Fig. 51:**
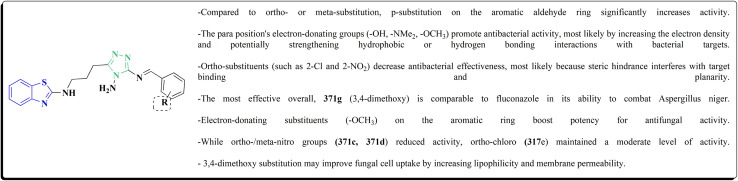
Structure–activity relationship of compound 371.

By increasing the electron density on the aromatic ring, electron-donating groups (EDGs) like –OH (371b), –NMe_2_ (371f), and –OCH_3_ (371g) can: –strengthen donor/acceptor hydrogen-bond interactions with microbial biomolecules. –May increase lipophilicity, which would facilitate penetration of cell walls and membranes. –Nitro (371c, 371d) and other electron-withdrawing groups (EWGs) reduce electron density and typically diminish activity, potentially as a result of less interaction with π-rich or nucleophilic sites in the microbial target (Fig. 51).

In addition, the benzothiazole–triazole scaffold has key role: the benzothiazole moiety is recognized for its ability to intercalate DNA or attach to grooves in microorganisms. It facilitates hydrogen bonding and π-interactions for enzyme inhibition.

On the other hand the ring 1,2,4-triazole has the ability to bind with cytochrome P450 enzymes' heme iron, which is crucial for antifungal action. Also it promotes hydrogen bonding and could help microbial metalloenzyme chelation. Compound 371g has most potency against the anti-fungal activity. Possibly as a result of the methoxy groups' improved lipophilicity, which facilitates the penetration through fungal cell membranes, 3,4-dimethoxy substitution enhanced efficacy against Candida albicans and *A. niger*. Additionally, they might line up to better fit the hydrophobic regions of fungal enzyme targets, such as sterol biosynthesis enzymes.

- The presence of *para*-electron-donating substituents in the arylidene ring maximizes activity by maintaining conjugation and planarity with the benzothiazole–triazole core, which improves target binding and microbial penetration. This is disrupted by *ortho*-substitution, and the interaction potential is decreased by electron-withdrawing groups.^231^

**Scheme 75:**
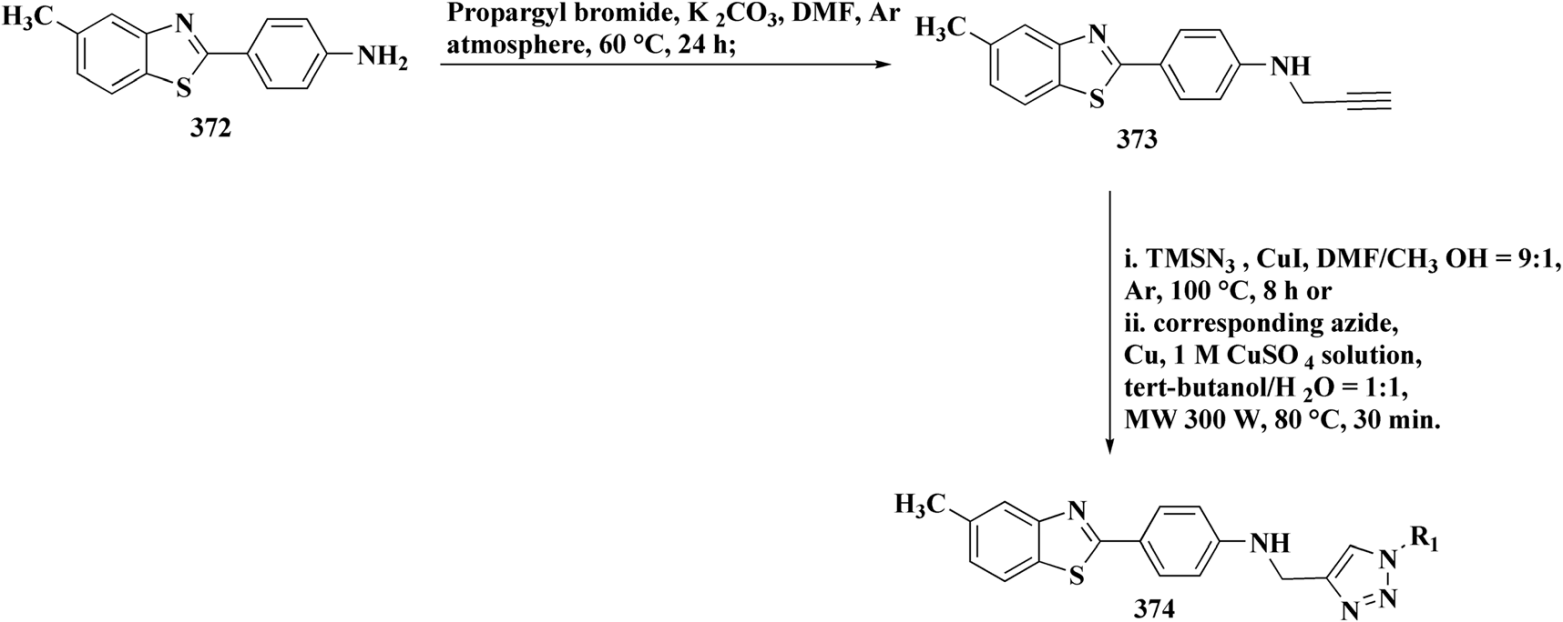
Synthesis of 1,2,3-triazole based bis-heterocycles.

The halogen-substituted aromatic azide precursors essential for the synthesis of 1,2,3-triazole based bis-heterocycles 374 were prepared from the corresponding arylamines. 1,2,3-Triazole-benzofused heterocycle conjugates 374 were accessed by copper(i)-catalyzed click chemistry using microwave irradiation. The 1,2,3-triazole-tethered benzofused heterocycle–coumarin chimeras were synthesized from terminal alkynes and coumarin azides (Schemes 75).

The antibacterial activities of the compounds were evaluated against selected Gram (+ve) and Gram (−ve) bacteria. Hybridization aspect utilizing Cu(i)-catalyzed click reaction using microwave irradiation was assumed in synthesizing the triazole tethered heterocycles 374.^232^

**Fig. 52:**
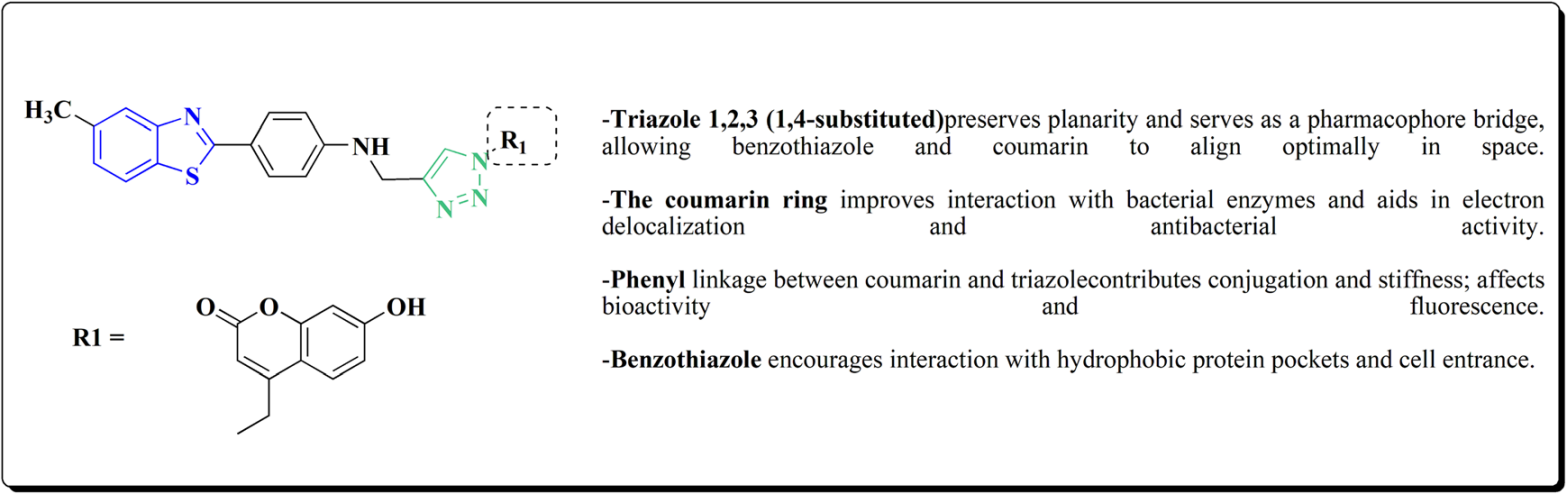
Structure–activity relationship of compound 374.

Benzothiazole, triazole, and the coumarin motifs were fused to generate a unique design (compound 374; Fig. 52) with specific anti-*M. catarrhalis* activity; the combined scaffold provides a distinct interaction profile than single motifs. This is why hybridization was important.

The active substances in this block were 7-substituted coumarins; the coumarin substitution pattern had a significant impact on both biological activity and fluorescence. The coumarin fragment's function was reported to affect the antibacterial potency. Rather than having broad Gram-negative/Gram-positive coverage, the activity was selective for the picky Gram-negative *M. catarrhalis*, indicating a particular target or uptake/penetration characteristic unique to this species.^232^

**Scheme 76:**
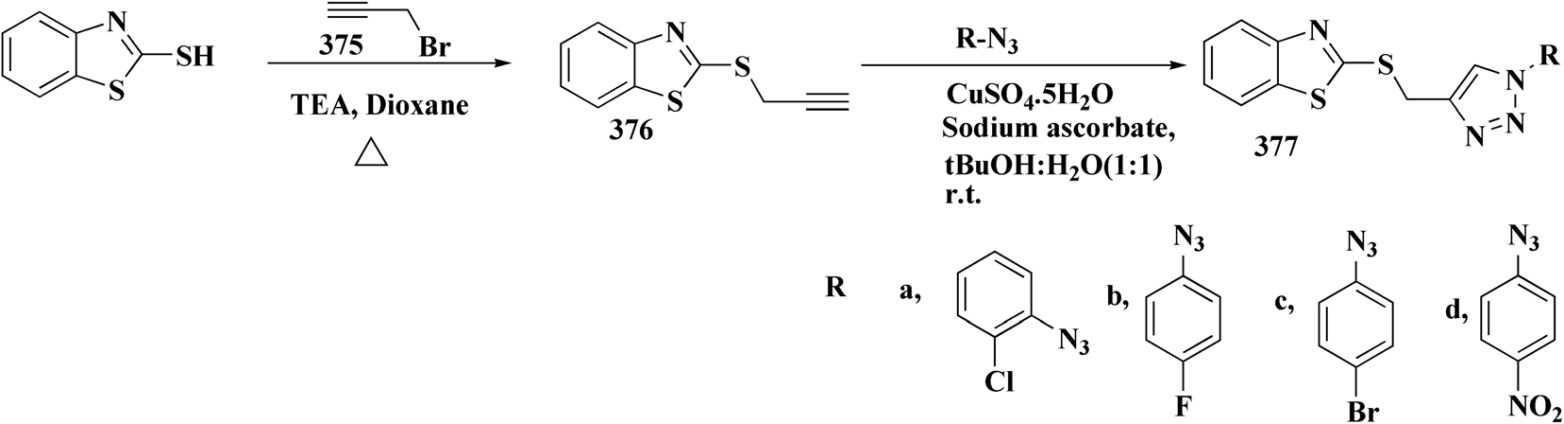
Synthesis of 1,2,3-triazole based benzothiazoles.

Bis-heterocycles were accessed *via* the cycloaddition reaction of compound 376 with various azides. 2-Mercaptobenzothiazole was reacted under reflux with propargyl bromide in dry THF to afford compound 376. The azides were allowed to react with propargylated 2-mercaptobenzothiazole (376) under click chemistry conditions to afford compound 377. Aromatic azides with different substituents were reacted with compound 376 (Scheme 76).

**Fig. 53:**
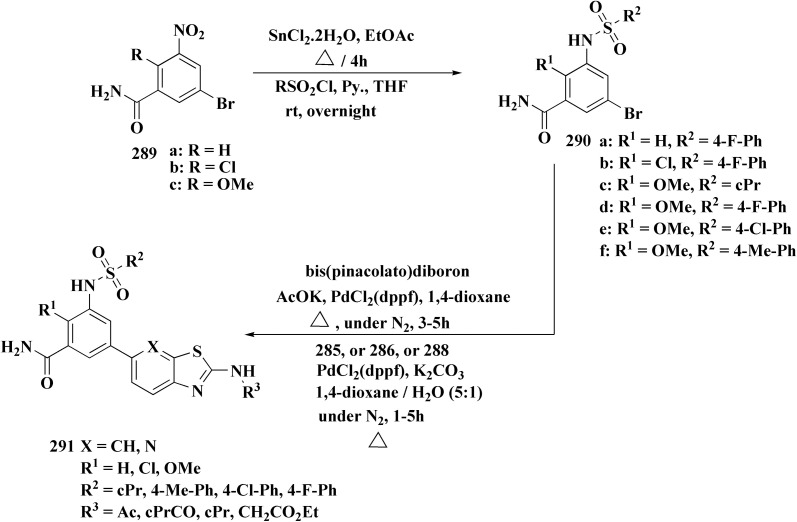
Structure–activity relationship of compound 377.

The synthesized compounds have been examined for their anti-inflammatory potency *via* the utilization biochemical cyclooxygenase (COX) activity assays and carrageen an-induced hind paw edema. Compound 377d (Fig. 53) displayed a potent selective COX-2 inhibition with COX-2/COX-1 ratio of 0.44. Results from carrageenan-induced hind paw edema indicated that compounds 377a, 377b, 377c and 377d have significant anti-inflammatory activity comparable to Ibuprofen. Crucially, none developed stomach ulcers, indicating perhaps better gastro-safety in comparison to standard NSAIDs.^233^

**Scheme 77:**
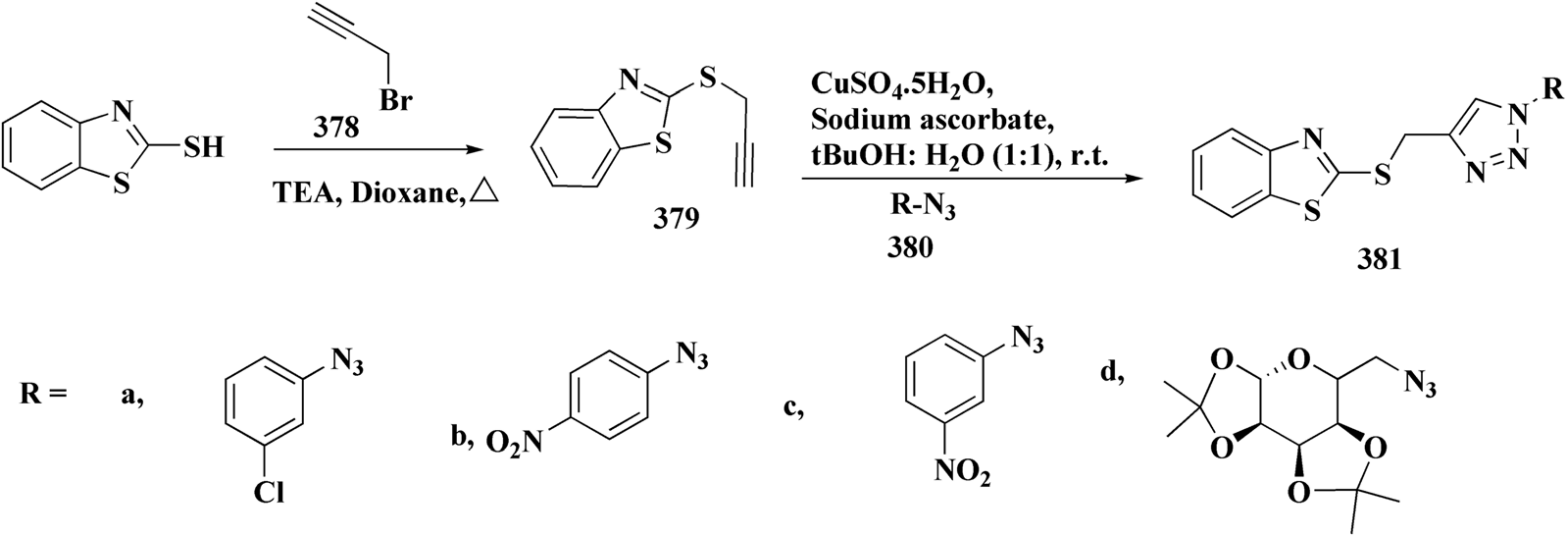
Synthesis of benzothiazole-1,2,3-triazole conjugates.

**Fig. 54:**
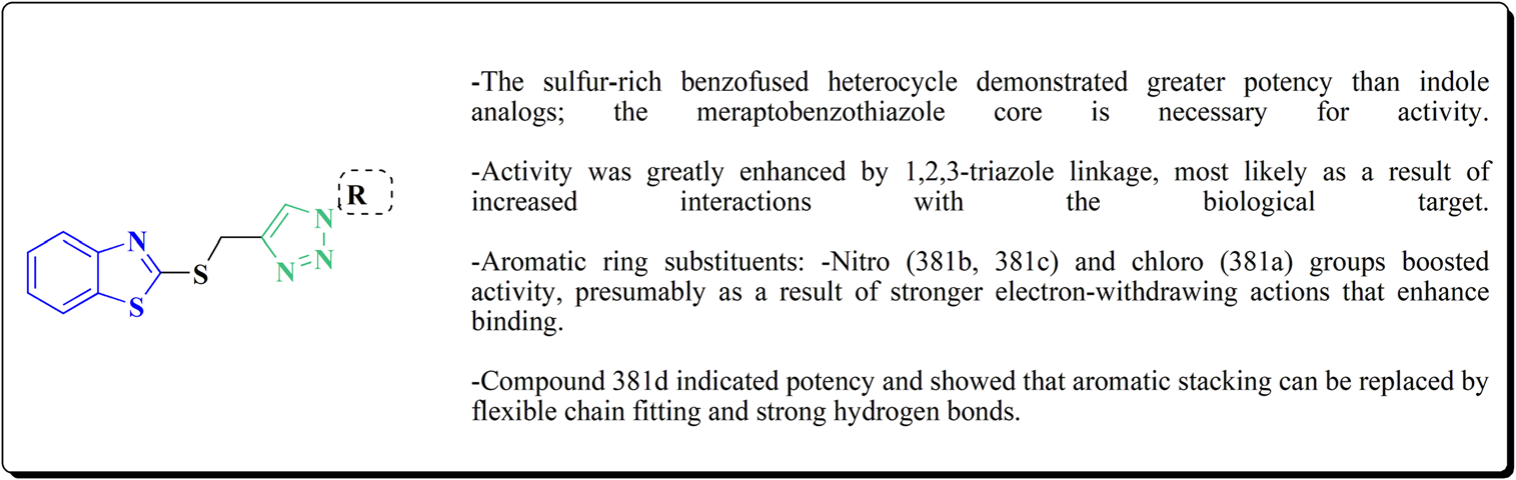
Structure–activity relationship of compound 381.

The 1,2,3-triazole conjugates of 2-mercaptobenzothiazole was synthesized by an azide–alkyne cycloaddition catalyzed by copper(i) (also known as “click chemistry”) as depicted in Scheme 84. First, in the presence of triethylamine in dioxane under reflux, 2-mercaptobenzothiazole was alkylated with propargyl bromide to get the propargyl derivative 379. Separately, other substituted benzyl halides or similar precursors reacted with sodium azide to yield the respective azides 380. In order to synthesize the 1,4-disubstituted 1,2,3-triazole ring that connects the benzothiazole core and the substituted side chain, the alkyne intermediate 379 and the azides 380 were subsequently exposed to click conditions, which included CuSO_4_·5H_2_O and sodium ascorbate in a *t*-BuOH/H_2_O (1 : 1) combination at room temperature. The target bis-heterocyclic compounds 381 were obtained in high isolated yields (87–97%) using this one-pot, regioselective method (Scheme 77; Fig. 54).

The compounds were screened against *Mycobacterium tuberculosis* H37Rv strain for their antitubercular activity.^234^

**Scheme 78:**
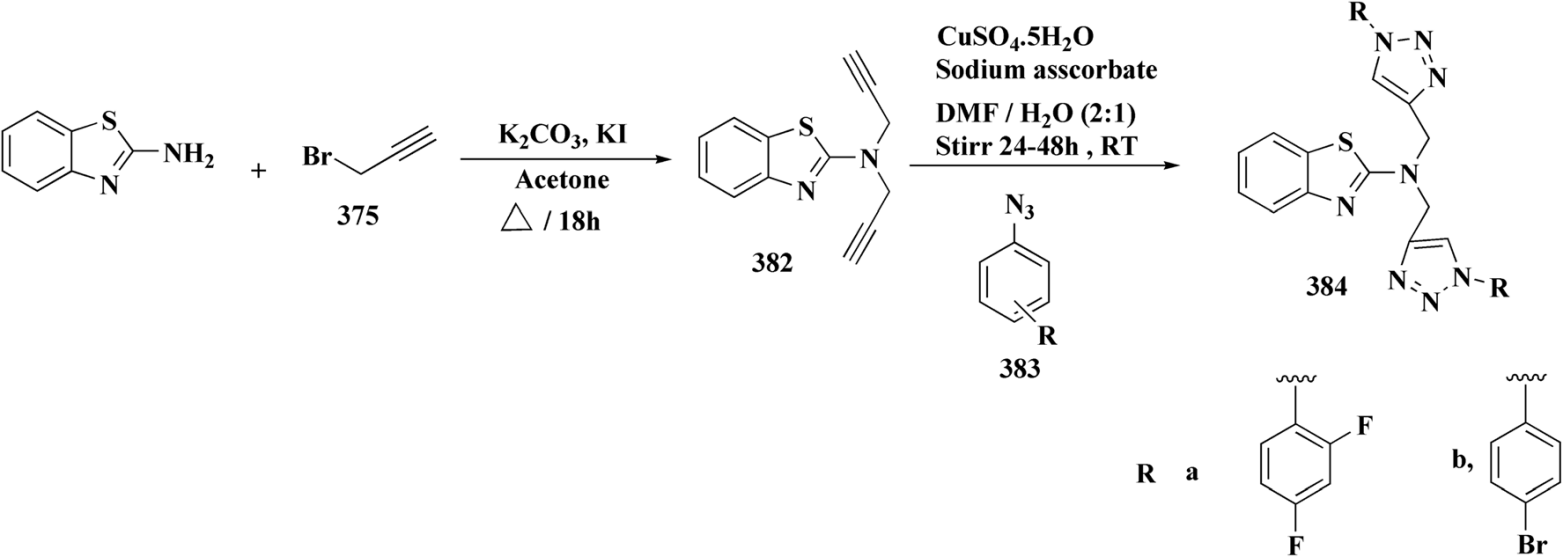
Synthesis of substituted aryl azides.

Dialkyne substituted benzo[*d*]thiazol-2-amine was reacted with different substituted aryl azides to furnish compounds 384 by click chemistry (Scheme 78). These compounds were screened for their antibacterial activity. The compound 384a indicated maximum activity against all Gram (+ve)/Gram (−ve) bacterial strains with MIC value 3.12 mg mL^−1^, which is two-fold more potent comparable to ciprofloxacin (MIC 6.25 mg mL^−1^). Compound 384b was found to be the most potent against all fungal strains with MIC value ranging from 1.56 mg mL^−1^ to 12.5 mg mL^−1^.^235^

**Fig. 55:**
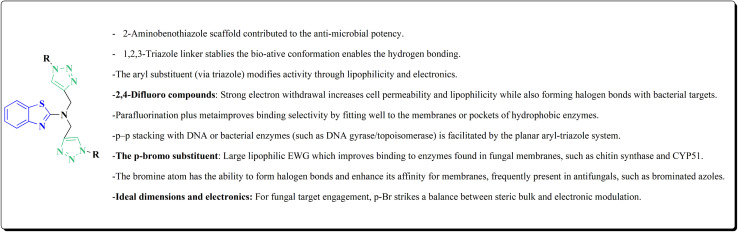
Structure–activity relationship of compound 384.

Fluorine atoms may interact favorably with the active regions of enzymes and enhance membrane penetration. The difluoroaryl group's hydrophobic and polar interactions are probably what give compound 384a its enhanced activity (Fig. 55). Compound 384b most likely interacts with biosynthetic enzymes or fungal membrane proteins, and because of its size and polarizability, the bromine improves binding and selectivity.

Because of its fluorine atoms, 384a probably targets bacterial enzymes like DNA gyrase and MurB and more easily passes through Gram(−) membranes. Bromine increases the binding affinity and selectivity of 384b, which most likely binds to fungal cytochrome enzymes or cell wall synthesis machinery.^235^

#### 9.1.4. Thiazole–benzothiazole based analogues

The reaction of 4,5-dichloro-1,2,3-dithiazolium chloride (Appel's salt) with *ortho*-halogenated anilines, aminophenols, and aminopyridines afforded the aryliminodithiazoles. Copper(i)-mediated or nucleophilic-assisted cyclization of aryliminodithiazoles generated cyano-functionalized benzothiazoles. The latter were reacted with substituted thiazol-2-amines to afford novel polyaromatic carboximidamides.

**Scheme 79:**
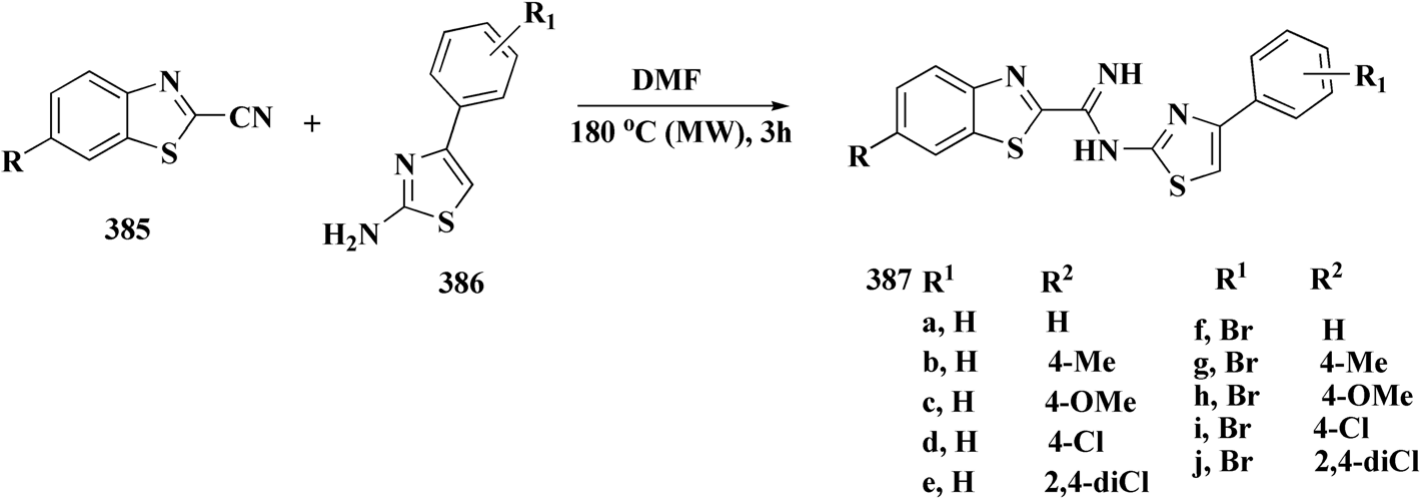
Synthesis of benzothiazole–thiazole conjugates.

**Fig. 56:**
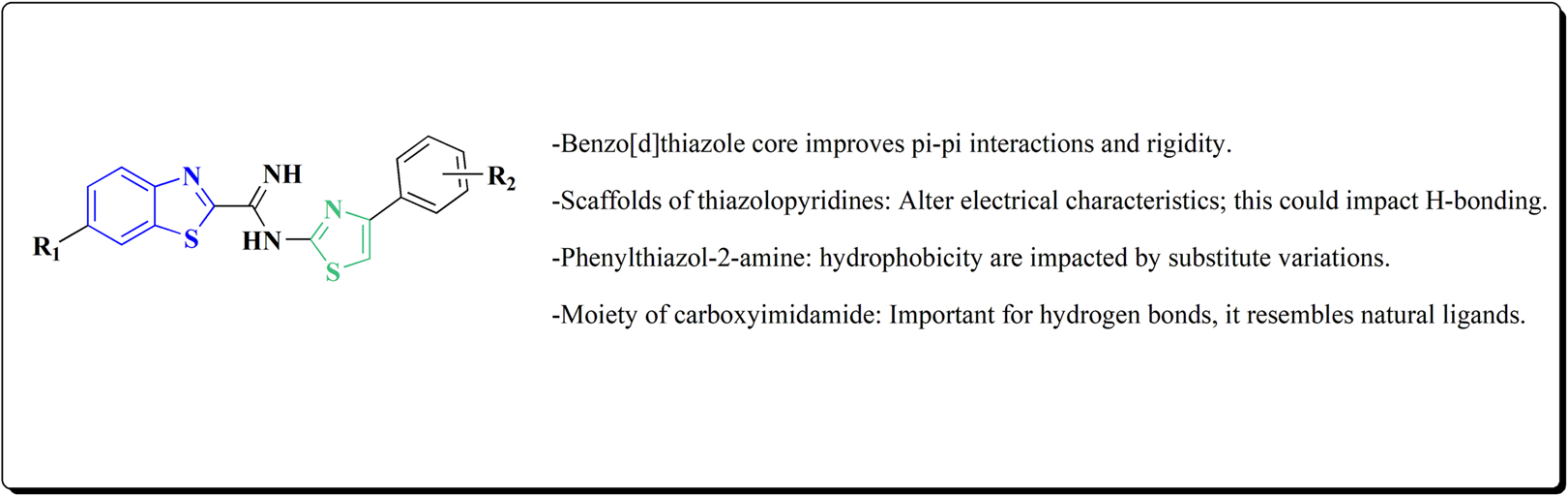
Structure–activity relationship of compound 387.

Microwave irradiation of the carbonitrile and the amine in dimethylformamide afforded the substituted benzo[*d*]thiazole-2-carboximidamides 387 (Scheme 79; Fig. 56).^236^

**Scheme 80:**
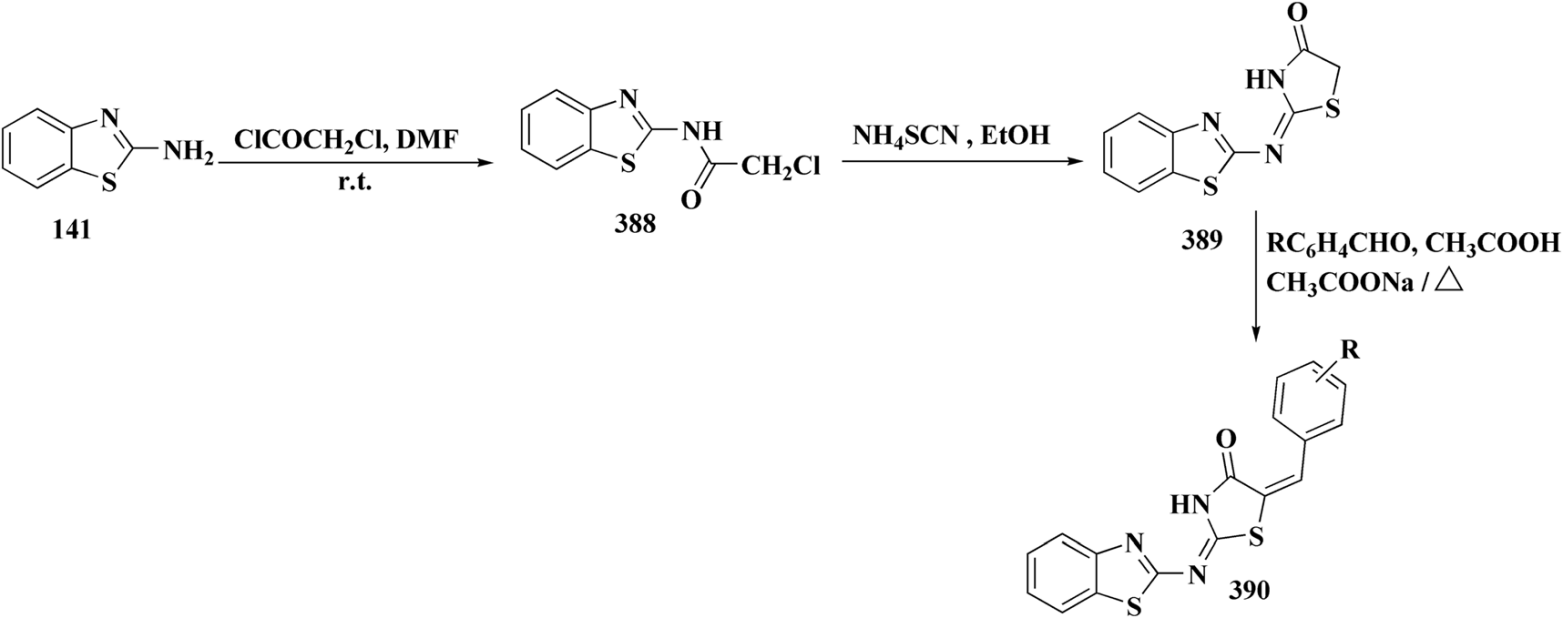
Synthesis of 2-(benzothiazolylimino) thiazolidin-4-ones.

**Fig. 57:**
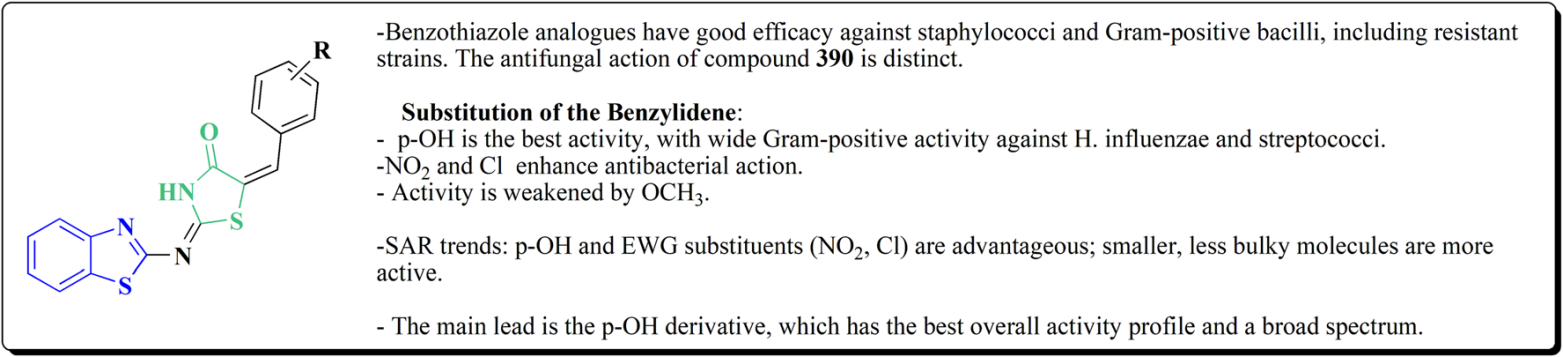
Structure–activity relationship of compound 390.

The 2-(benzothiazolylimino) thiazolidin-4-one 390 was achieved in Scheme 87. The synthetic procedures were preformed by reacting chloroacetylchloride with the appropriate amine. Cyclisation of 2-chloro-*N*-(etheroaryl)acetamide, yielded the 2-(heteroarylimino)thiazolidin-4-ones 389. Compounds 390 were accessed by refluxing the latter intermediates with aldehydes (Scheme 80; Fig. 57).

The compounds were assayed *in vitro* for their antimicrobial potency. Most of the benzothiazole analogues exhibit good inhibition of the growth of Gram (+ve) staphylococci and bacilli. Among the synthesized analogues a few derivatives indicate a selective and strong activity against bacilli.^237^

**Scheme 81:**
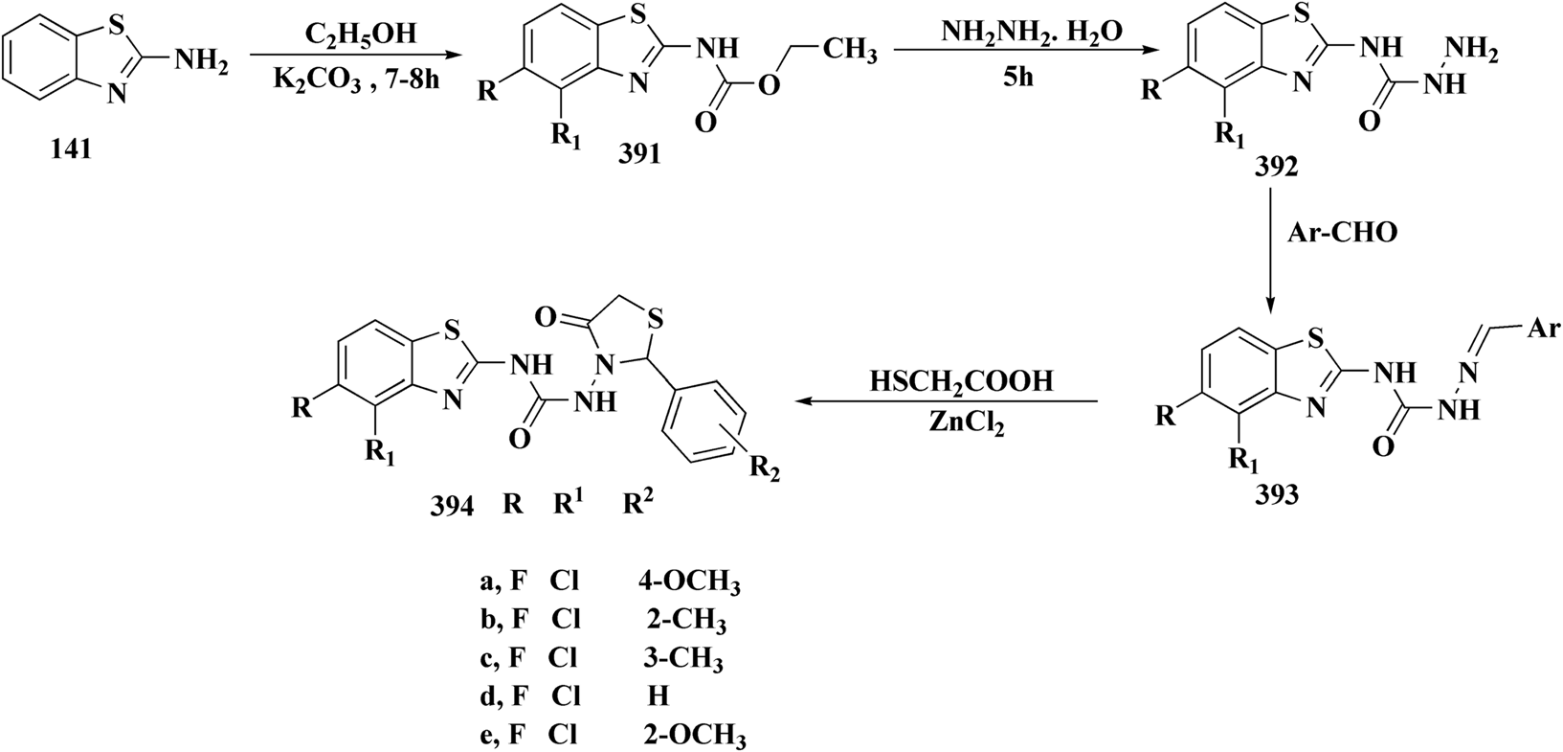
Synthesis of benzothiazolyl ureas.

The preparation of the benzothiazolyl ureas 394 were accessed by reaction with the semicarbazon 393 with DMF, thioglycolic acid and zinc chloride (Scheme 81).

The synthesized compounds indicated good to moderate anthelmintic potency against *Perituma posthuma*. The results of the ureas derivatives revealed anthelmintic activity against Albendazole.^238^

**Fig. 58:**
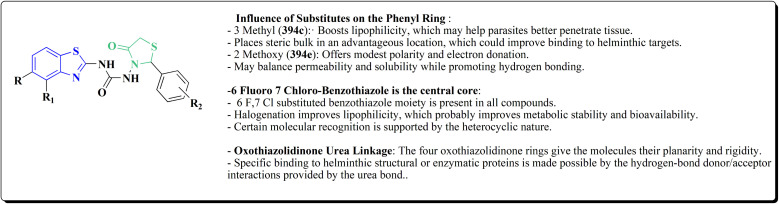
Structure–activity relationship of compound 394.

Analogues 394c and 394e (Fig. 58) were the most effective anthelmintic compounds against Perituma posthuma, outperforming other compounds in the series when compared to albendazole.

The uptake of parasite tissue is improved by lipophilicity, which is boosted by F, Cl, and methyl. Methoxy and other substituents that provide electrons may improve interactions with helminth targets. Planar arrangement and structural rigidity facilitate efficient target engagement in worm metabolic pathways.The most effective anthelmintic analogue is TH18, which has a 3 methyl phenyl group. Better hydrophilic balance and virtually equal activity are provided by TH20 (with a 2 methoxy substituent).These emphasizes how crucial strategically positioned lipophilic or H-bonding substituents are for this scaffold's best performance.^238^

#### 9.1.5. Imidazole–benzothiazole based analogues

**Scheme 82:**
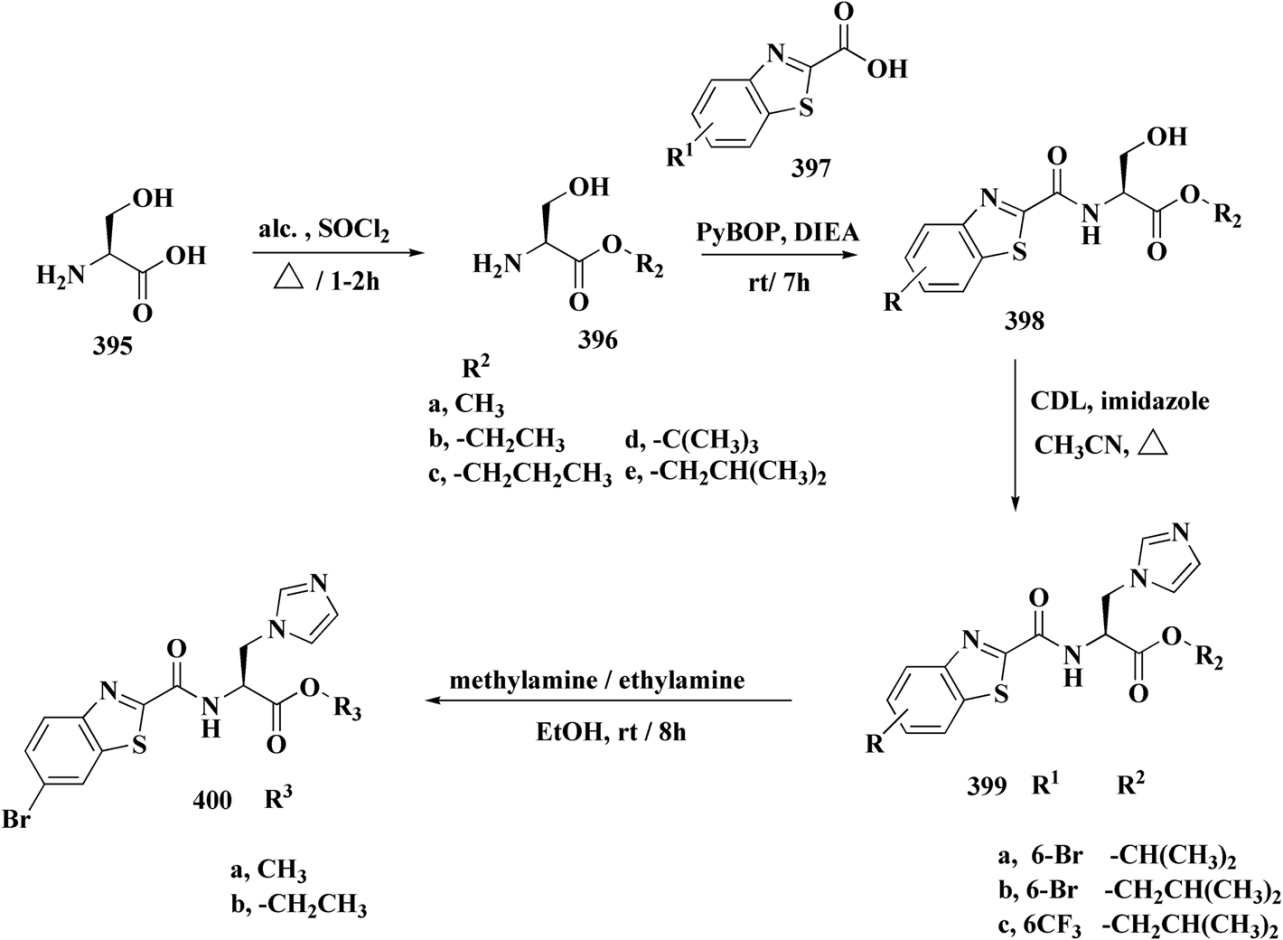
Synthesis of substituted benzo[*d*]thiazole-2-carboxamide.

l-Serine 395 was dissolved in alcohol and refluxed with thionyl chloride to yield the serine esters 396a–e. Compounds 396 were then treated with the intermediates 397 to afford compounds 398. The desired compounds 399 were accessed through the introduction of an imidazole group. Compounds 400a–b were prepared from 399*via* an aminolysis reaction (Scheme 82).

**Fig. 59:**
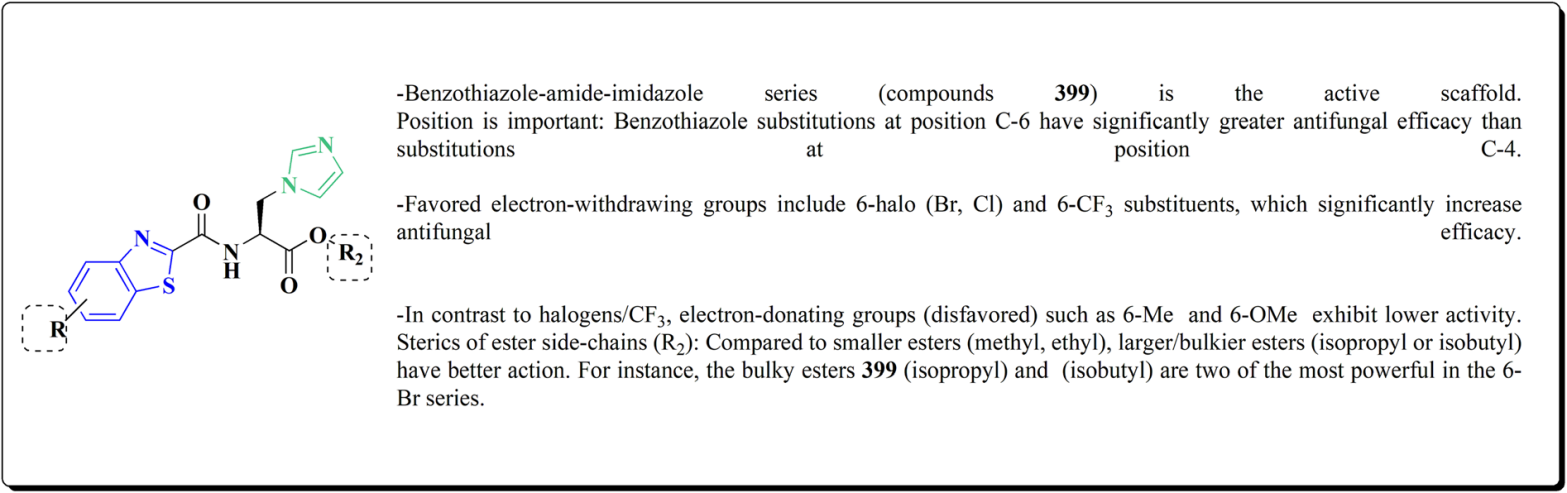
Structure–activity relationship of compound 399.

The antifungal activity of these compounds was tested *in vitro*, and their SARs were estimated. The synthesized compounds indicated excellent inhibitory activity against *Cryptococcus neoformans* and *Candida albicans*. The most active compounds 399a, b, and c (Fig. 59) revealed potent activity, with MIC values ranging from 0.125 to 2 μg mL^−1^.^239^

The experimental CYP51 inhibition was performed and a significant drop in ergosterol and an accumulation of lanosterol and eburicol, the same sterol-pattern generated by fluconazole (a known CYP51 inhibitor), were observed in GC-MS sterol profiling following *C. albicans* treatment with 399b (R^1^ = 6-Br; R^2^ = (–CH_2_CH(CH_3_)_2_). This study clearly shows that fungal lanosterol 14α-demethylase (CYP51) is inhibited by 399b.

*In silico* docking/binding hypothesis shows that fungal CYP51 docking (CDOCKER) reveals: –(azole-like coordination) the heme iron is coordinated by the imidazole. –A hydrophobic pocket accommodates the alkyl ester (isobutyl) (contacts Ile139, Met313), and hydrophobic/vdW interactions are formed when the benzothiazole tail extends into the enzyme channel (His381, Phe241, Pro238, Leu95, Ala69). These interactions explain (a) why an imidazole is necessary, (b) why large hydrophobic ester groups are advantageous, and (c) why potency is increased by lipophilic electron-withdrawing C-6 substituents.

Activity against fluconazole-resistant strains: fluconazole is inert (>64 μg mL^−1^), whereas 399b maintains modest activity against two fluconazole-resistant *C. albicans* strains (MICs 8–16 μg mL^−1^). This suggests that 399b may overcome some resistance mechanisms and indicates partial (not full) cross-resistance.^239^

**Scheme 83:**
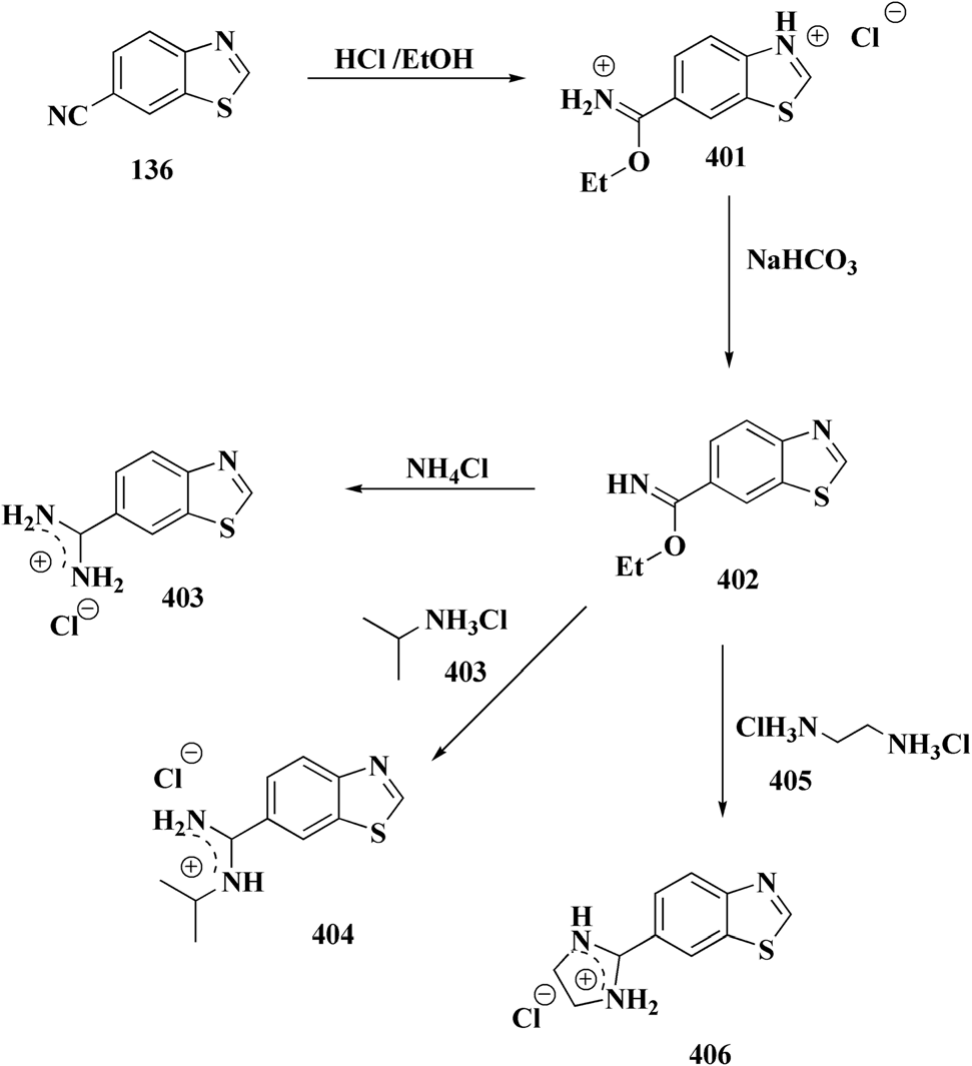
Synthesis of substituted benzothiazoles.

New amidino, 2-imidazolinyl, *N*-isopropylamidino-substituted benzothiazoles and 2-aminothiophenoles were synthesized *via* the Pinner reaction is shown in Scheme 83. The new ring opening of benzothiazole with ethylenediamine and ammonia was occurred. The ring opening with ethylenediamine is appropriate to compounds having amonolytically and hydrolytically unstable substituents.^240^

**Scheme 84:**
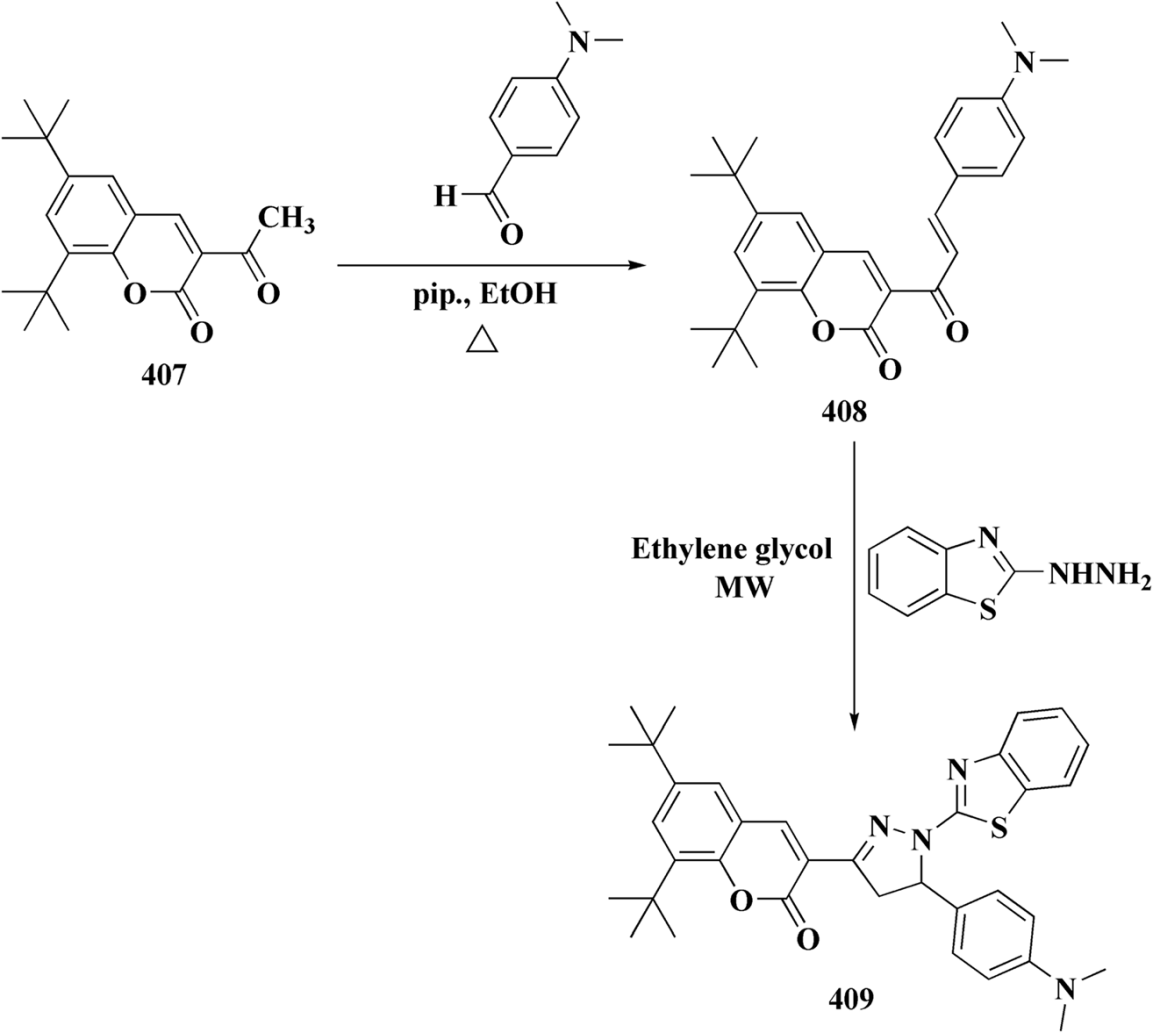
Synthesis of substituted benzothizole 409.

Heterocycle-based chromophores comprising pyrazoline scaflods were synthesized using microwave irradiation (Scheme 84). The heterocyclic chromophores were fluorescent, with some examples emitting blue light (410–460 nm) whilst others emitted green light (529 nm). The absorption maxima of the chromophores were found to vary from 349 to 463 nm conditional on the degree of conjugation.^241^

These compounds' emission and absorption spectra were recorded in a solution of chloroform. New heterocyclic chromophores were discovered to have fluorescene in solution. Green light was emitted by compounds 409 (529 nm). These heterocyclic chromophores may be used as optical materials in a variety of applications, including biosensors, fluorescent probes in biological applications, OLED materials, and two-photon absorption materials.

#### 9.1.6. Oxadiazole–benzothiazole based analogues

**Scheme 85:**
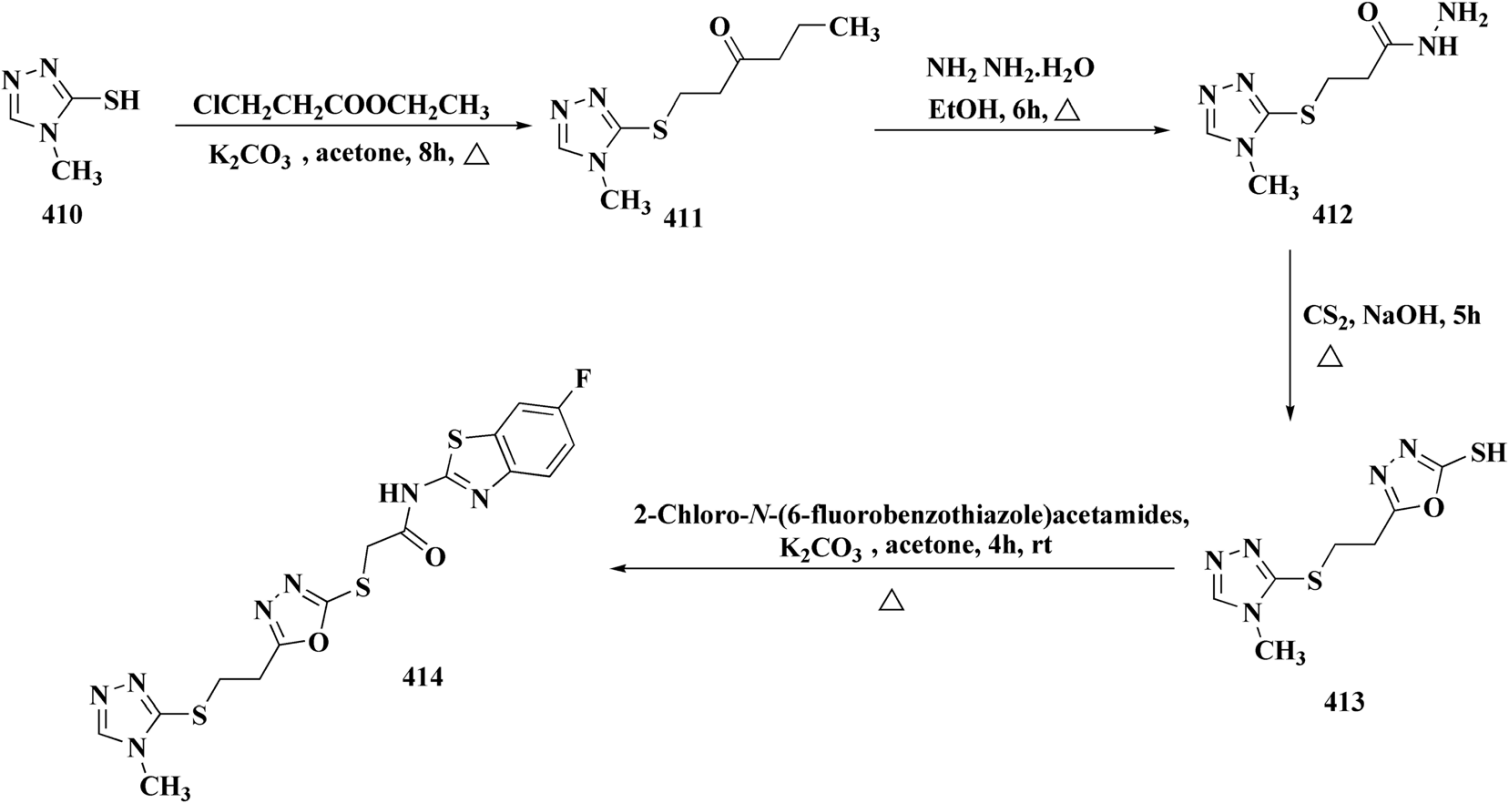
Synthesis of triazole-oxadiazole benzothiazole conjugates.

Novel triazole–oxadiazole compounds were prepared as shown in scheme. 4-Methyl-4*H*-1,2,4-triazole-3-thiol was reacted with 3-chloropropionic acid ethyl ester to afford ethyl 3-[(4-methyl-4*H*-1,2,4-triazol)thio]propionate 411. The ester group of compound 411 was hydrazinated to afford the hydrazide 412. The intermediate 413 was acheived *via* ring-closure reaction of compound 412 with carbon disulfide (Scheme 85).

**Fig. 60:**
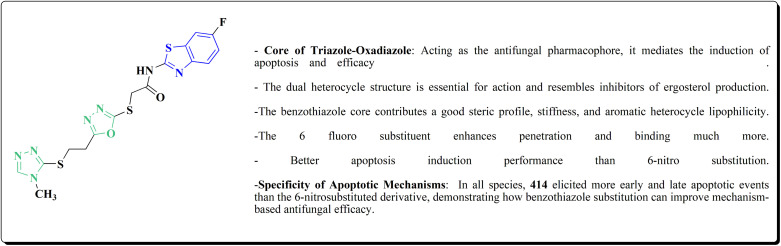
Structure–activity relationship of compound 414.

The *in vitro* apoptotic and antifungal activity were tested. The compounds 414 (Fig. 60) were detected as the most potent compounds against *C. glabrata* and *C. albicans*.^242^

**Scheme 86:**
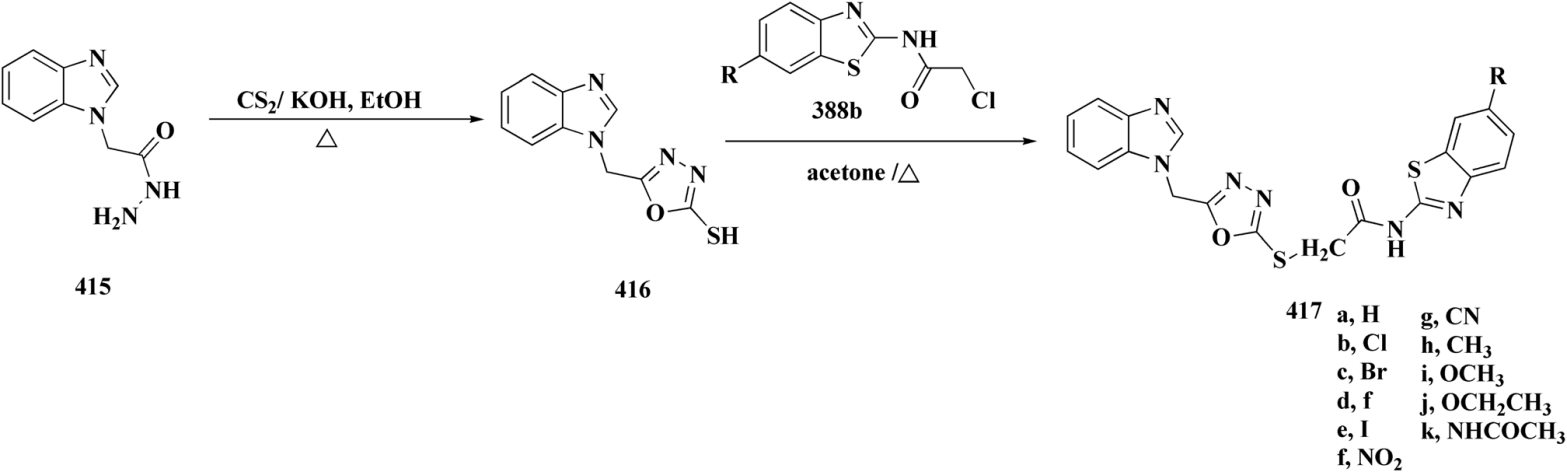
Synthesis of 2-amino-6-substituted benzothiazoles.

**Fig. 61:**
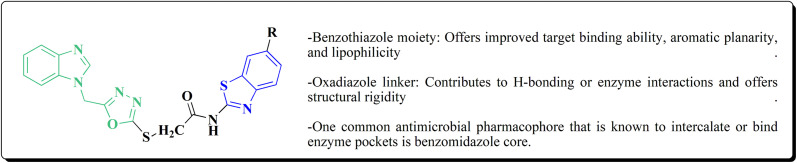
Structure–activity relationship of compound 417.

The 2-amino-6-substituted benzothiazoles were transformed to the chloroacetamides 412 using chloroacetyl chloride. The acetamide derivatives 412 were then condensed to the oxadiazole-2-thiol 416 to yield 417 (Scheme 86; Fig. 61).

Upon biological screening, it was detected that the majority of the compounds were found to have a remarkable broad spectrum antitubercular (6.25–25 mg mL^−1^ of MIC) and antimicrobial (3.12–25 mg mL^−1^ of MIC) potential.^243^

**Scheme 87:**
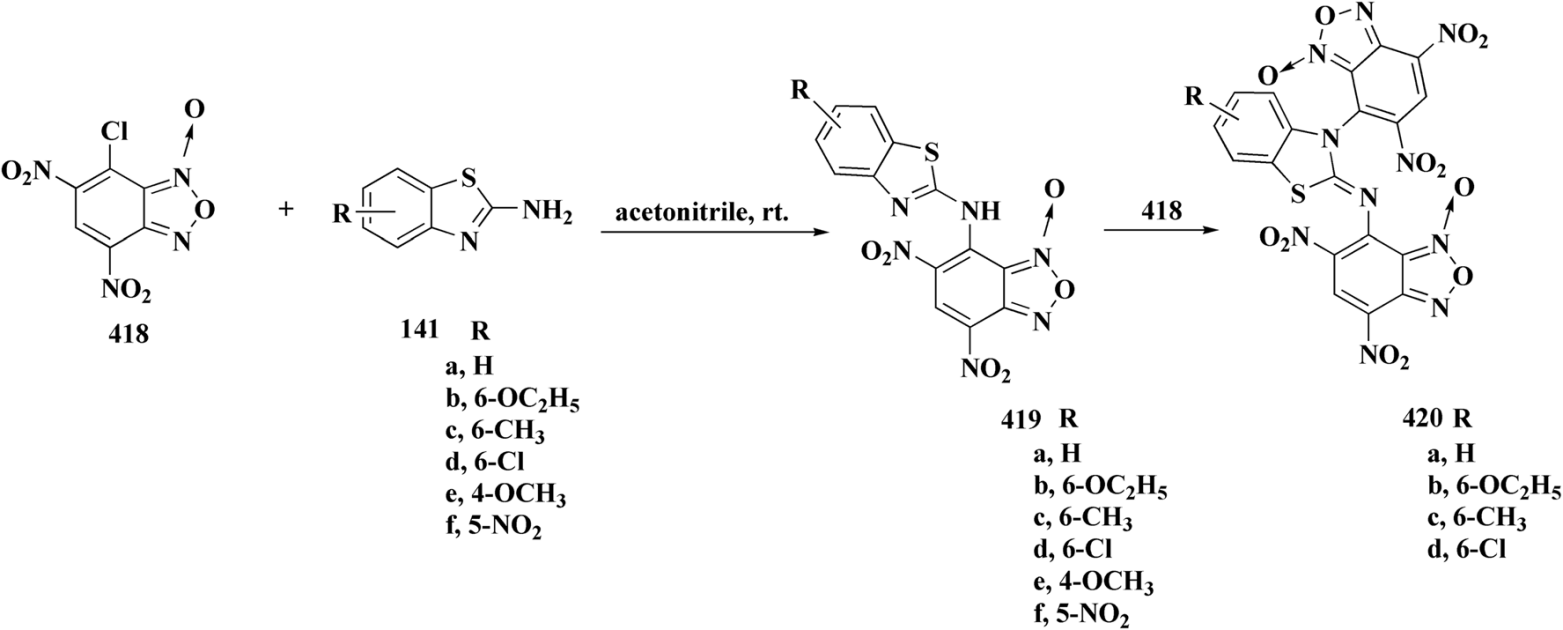
Synthesis of 2-aminobenzothiazole-benzofuroxan conjugates.

**Fig. 62:**
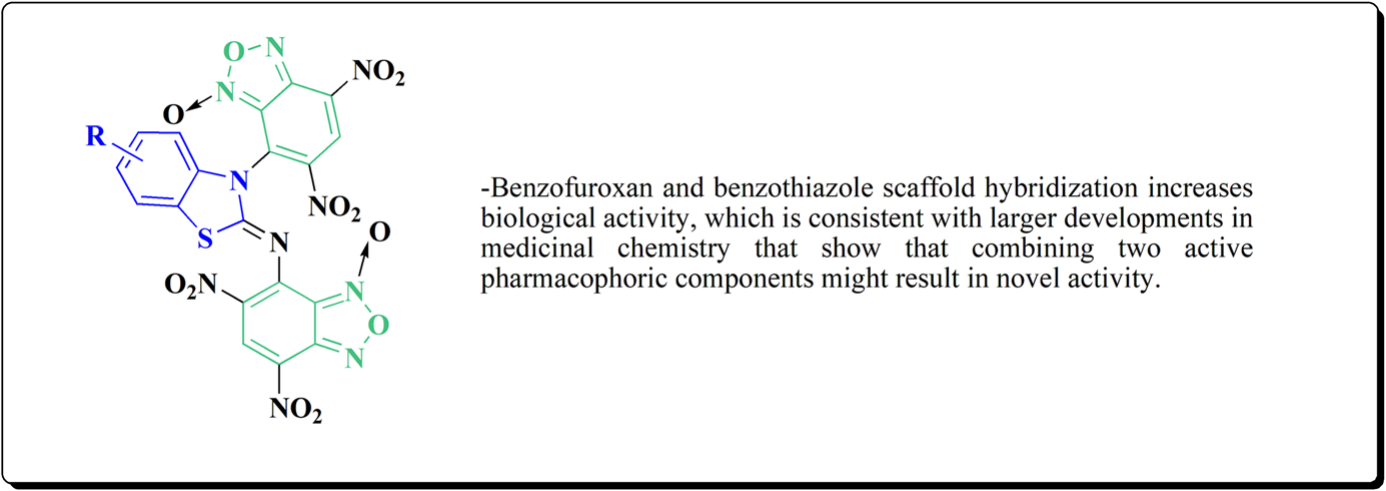
Structure–activity relationship of compound 420.

A mixture of mono-adducts 419a–d and di-adducts 420a–d were accessed from the reaction of benzofuroxan 418 and 2-aminobenzothiazole derivatives (Scheme 87; Fig. 62).^244^

#### 9.1.7. Pyrrole–benzothiazole based analogues

**Scheme 88:**
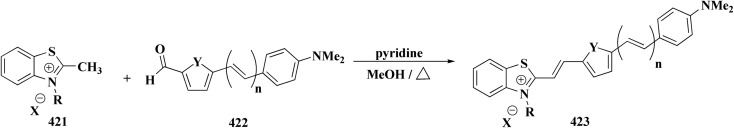
Synthesis of benzothiazolium derivatives.

New derivatives containing an electron-donating *N*,*N*-dimethylaminophenyl ring connected to an electron-withdrawing benzothiazole or benzothiazolium moiety *via* a heteroaryl system and up to two ethenylene groups have been prepared. Benzothiazolium derivatives 423 were acheived from carbaldehydes 322 and compounds 421 (Scheme 88).

The antimicrobial activities of the compounds were estimated against unicellular organisms. The 3-alkyl-benzothiazolium salts exhibited high toxicity against different tested microbes.^245^

**Fig. 63:**
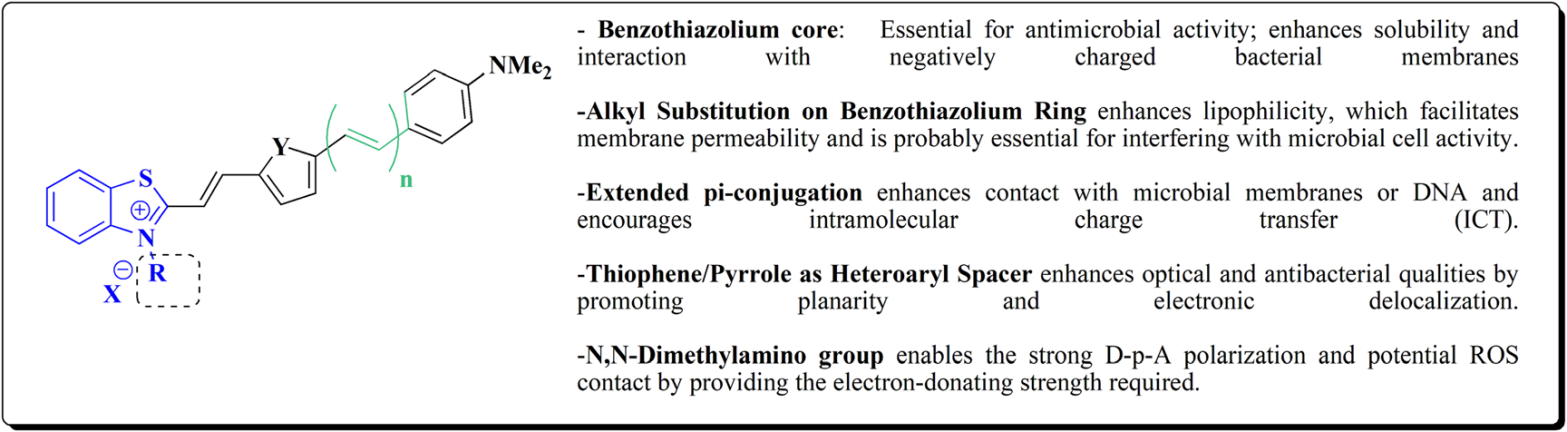
Structure–activity relationship of compound 423.

The antimicrobial potency is due to the cationic charge of the benzothiazolium core that disrupts the cell membranes of the bacteria. Also, the lipophilic alkyl chain enhances cellular uptake. The D–π–A conjugation facilitates the charge transfer, and cause interference with the microbial redox systems (Fig. 63).

### 9.2. Benzothiazoles linked with substituted aromatics

**Scheme 89:**
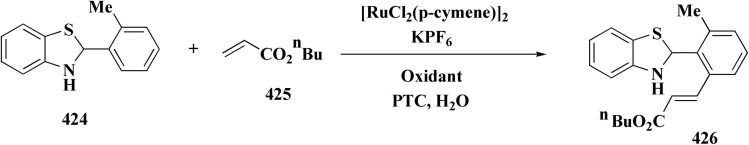
Synthesis of 2-arylbenzo[*d*]thiazoles.

**Fig. 64:**
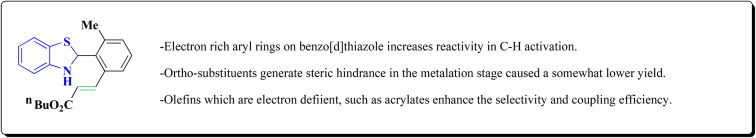
Structure–activity relationship of compound 426.

An effective Ru-catalyzed *ortho*-oxidative alkenylation of 2-arylbenzo[*d*]thiazoles *via* twofold C–H bond functionalization in aqueous solution of anionic surfactant sodium dodecylbenzenesulfonate (SDBS) has been developed using activated olefins as coupling partner (Scheme 89; Fig. 64).^246^

**Scheme 90:**
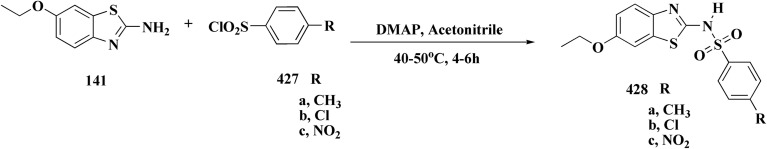
Synthesis of benzothiazole sulfonamides.

**Fig. 65:**
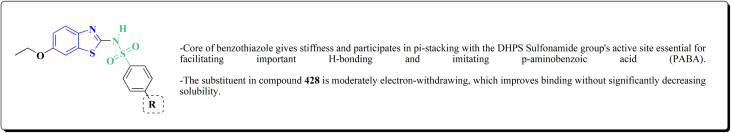
Structure–activity relationship of compound 428.

The sulfonamide has been prepared (Scheme 90; Fig. 65) and screened for antibacterial activity. It was found to be highly potent (MIC value 50–3.1 μg mL^−1^) against various human pathogens, and most effective against *E. coli*.^247^

Compound 428 exhibits broad-spectrum antibacterial activity, particularly against Gram-negative bacteria. Compound 428's SAR pattern demonstrates support for enhancing binding by using electron-withdrawing *para*-substituents on the sulfonyl phenyl group while maintaining the benzothiazole ring.^247^

**Scheme 91:**
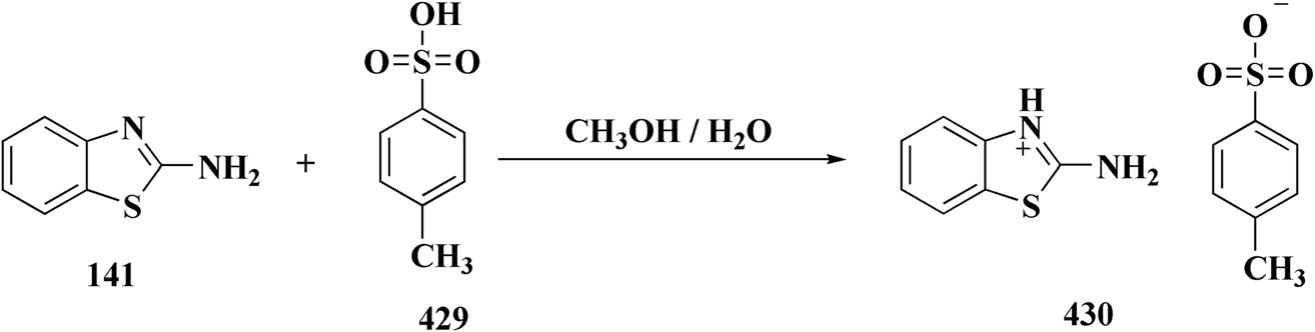
Synthesis of 2-aminobenzothiazolium-4-methylbenzenesulphonate.

**Fig. 66:**
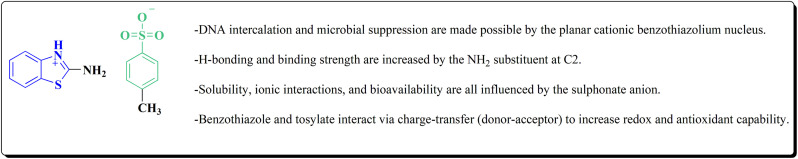
Structure–activity relationship of compound 430.

2-Aminobenzothiazolium-4-methylbenzenesulphonate (ABPTS) was synthesized as accomplished in Scheme 91. The antifungal and antibacterial activities of synthesized complex were examined against different fungi and bacteria strains. The free radical scavenging activity of the complex has been identified against ABTS, DPPH, and OH radicals (Fig. 66).^248^

Proteus sp., *P. aeruginosa*, *S. aureus*, *Aspergillus flavus*, *A. fumigatus*, and other bacteria are significantly inhibited by ABPTS.

Regarding the binding of DNA, the hypochromism was evident in the absorption titration using CT-DNA; the binding constant, *K*_b_ = 3.6 × 10^4^ M^−1^ as intercalative binding.

Dose-dependent cleavage of supercoiled pBR322 DNA into linear and nicked forms was demonstrated by agarose gel electrophoresis, which is compatible with intercalative/oxidative DNA disruption. Concerning the activity of antioxidants: ABPTS efficiently scavenged DPPH, hydroxyl, and ABTS radicals; its redox activity was confirmed by published IC_50_ values and comparison to ascorbic acid.^248^

**Scheme 92:**
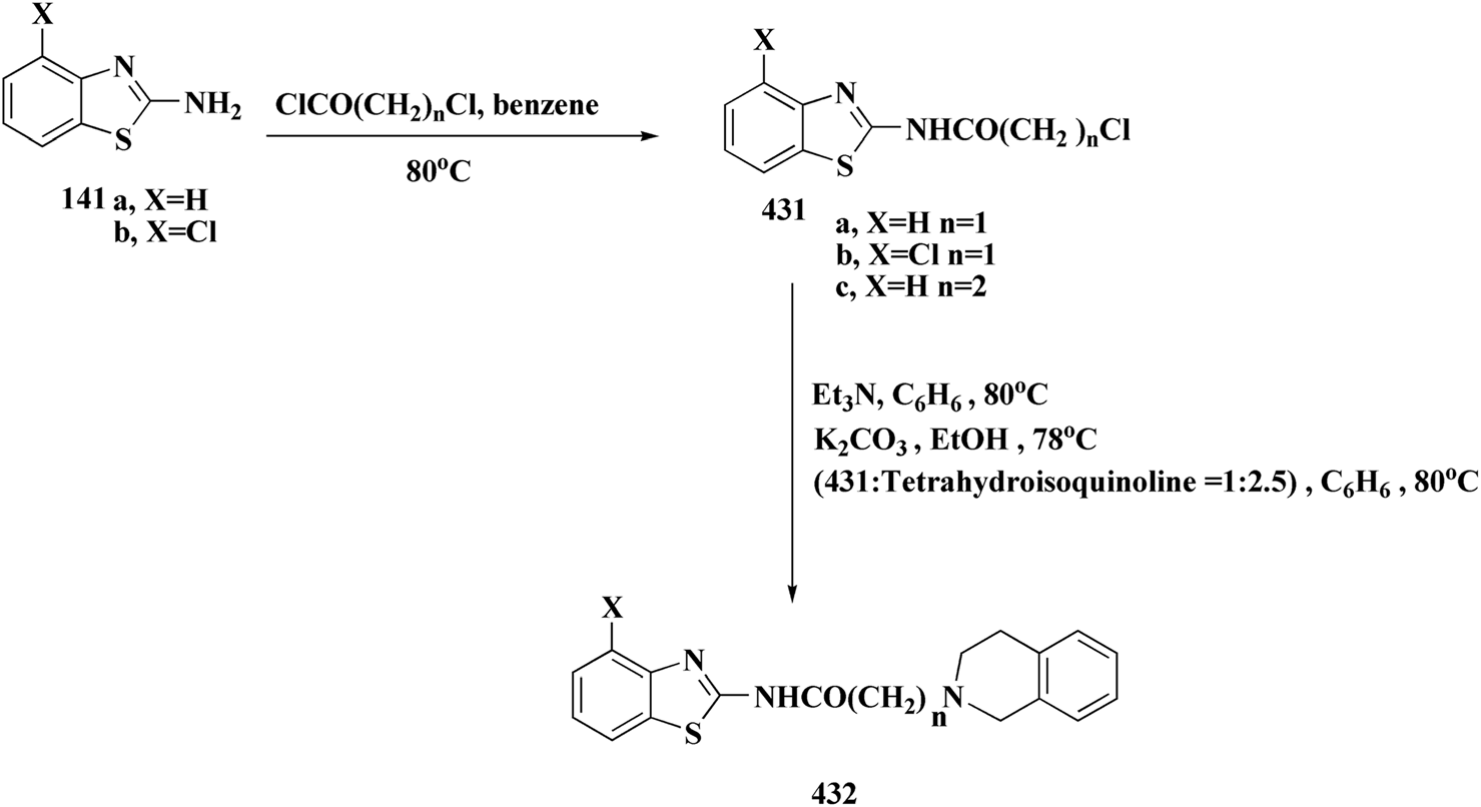
Synthesis of dihydroisoquinolin-2(1*H*)-yl]alkanamides.

The synthesis of compounds 432 is accomplished in Scheme 85. The strategy for the synthesis of dihydroisoquinolin-2(1*H*)-yl]alkanamides 432 involved the synthesis of the propanamido intermediates 431a–c, which were utilized as alkylating agents in reaction with 1,2,3,4-tetrahydroisoquinoline to give the targeted compounds (Scheme 92). The compounds were found to be potent in psychotropic, anti-inflammatory and cytotoxicity *in vitro* screening. They have obvious sedative action, show high antiinflammatory potency, have selective cytotoxic effects and nitric oxide (NO) induction ability regarding tumour cell lines. Some of the compounds synthesized exhibit antimicrobial activity.^249^

**Fig. 67:**
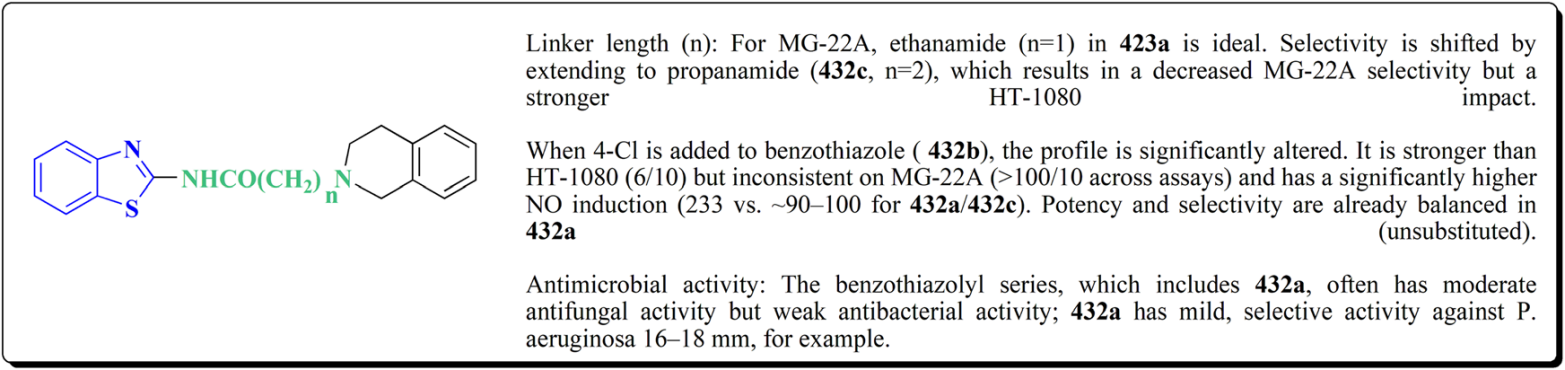
Structure–activity relationship of compound 432.

The compounds was synthesized by using an amide spacer to join a 3,4-dihydroisoquinoline fragment, which is present in a number of biologically active alkaloids, with a 1,3-benzothiazole moiety, which is known for its anticancer and antibacterial properties. The goal of this design was to generate a single structure that combined the pharmacological potential of both heterocyclic systems (Fig. 67).

The most effective motif for MG-22A in terms of tumor cytotoxicity is benzothiazole linked to a short linker (432a); ring substituents (432b) change selectivity and NO signaling, while lengthening the linker (432c) changes potency toward HT-1080 and antifungal properties (Fig. 66).^249^

**Scheme 93:**
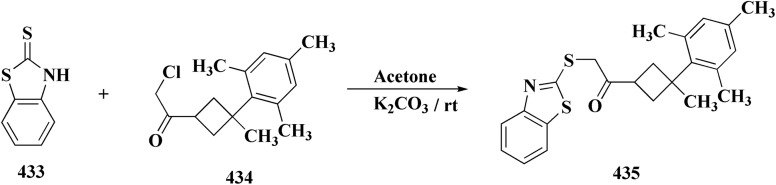
Synthesis of 2-(benzo[*d*]thiazol-2-ylthio)-1-((1*S*,3*S*)-3-mesityl-3-methylcyclobutyl) ethan-1-one.

**Fig. 68:**
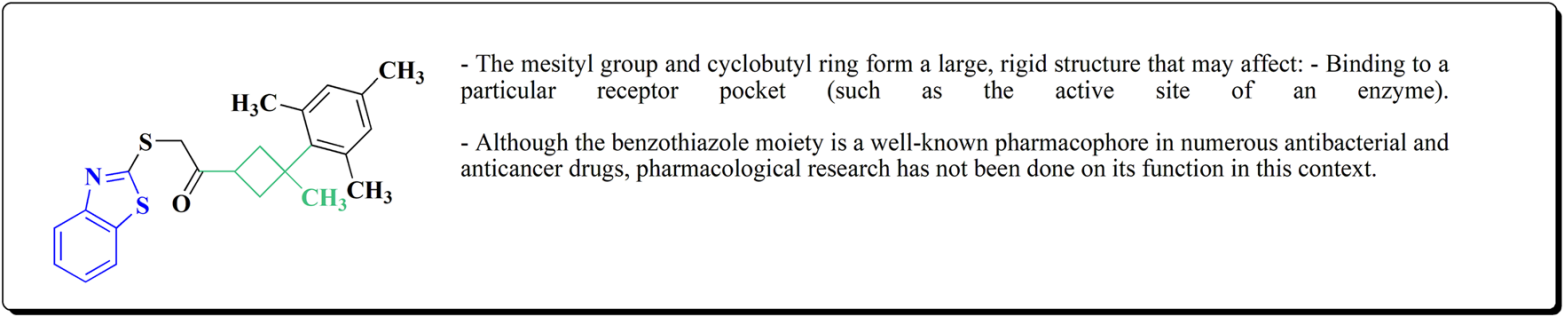
Structure–activity relationship of compound 435.

2-(Benzo[*d*]thiazol-2-ylthio)-1-((1*S*,3*S*)-3-mesityl-3-methylcyclobutyl) ethan-1-one was synthesized as shown in Scheme 93 (Fig. 68).^250^

**Scheme 94:**
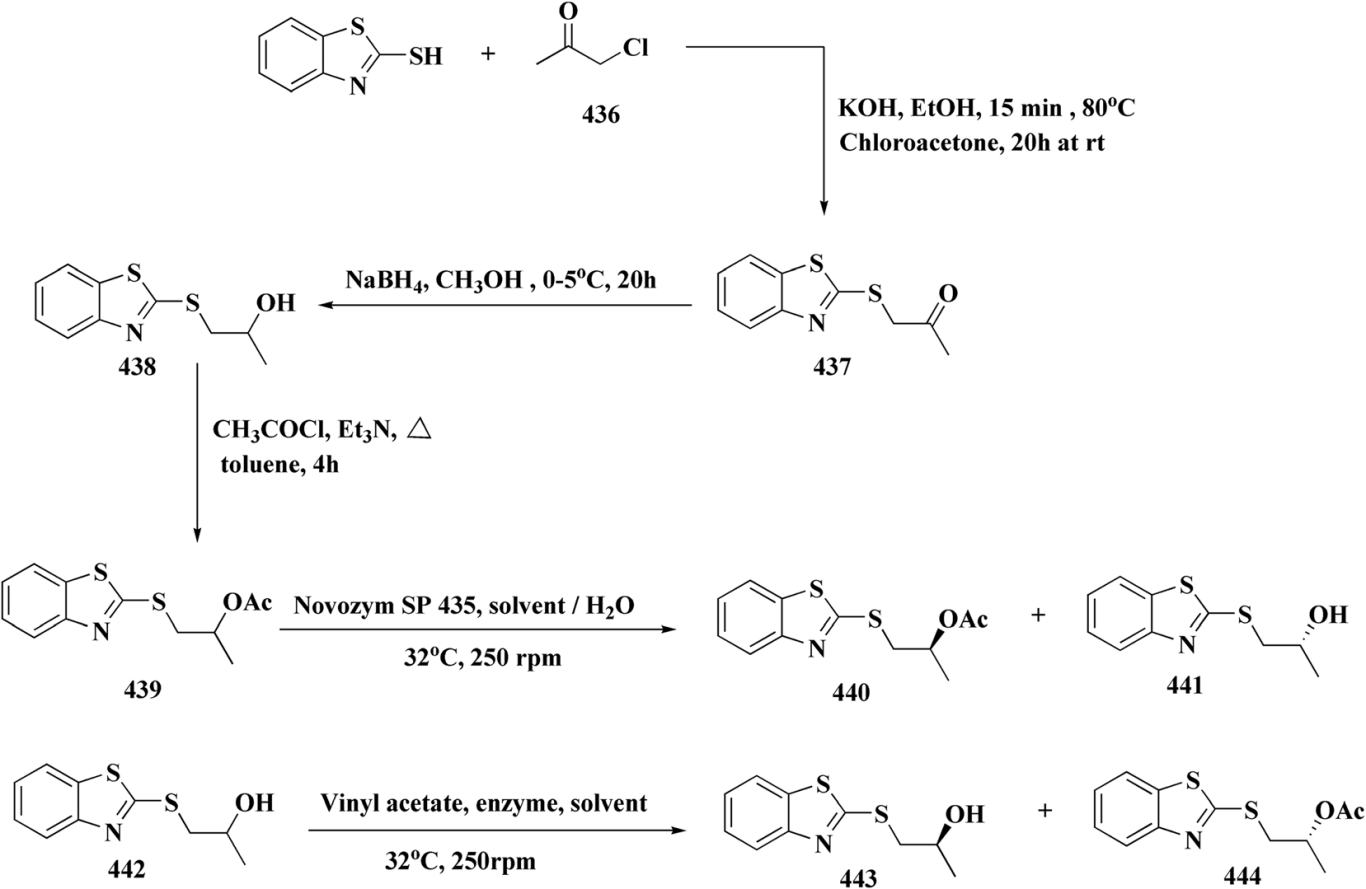
Synthesis of 1-(1,3-benzothiazol-2-ylsulfanyl)propan-2-ol.

The reaction of chloroacetone 436 with potassium hydroxide activated 1,3-benzothiazol-2-thiolate afforded the ketone 437. The reduction of the latter generated 1-(1,3-benzothiazol-2-ylsulfanyl)propan-2-ol. In order to synthesize an analytical standard of racemic resolution product, racemic alcohol was esterified with acetylchloride. This procedure yielded racemic acetyl ester (Scheme 94).

**Fig. 69:**
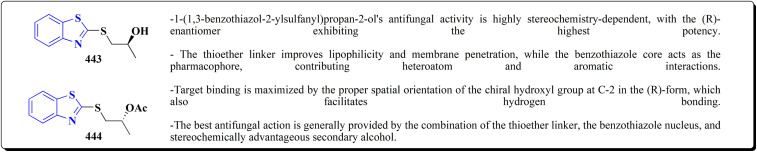
Structure–activity relationship of compound 443 & 444.

Chemoenzymatic synthesis of the two enantiomers of 1-(1,3-benzothiazol-2-ylsulfanyl)propan-2-ol was performed (Fig. 69). Different lipase preparations were examined as bio-catalysts in the kinetic resolution process of desired compound *via* enantioselective transesterification and/or hydrolysis. It was discovered that CAL-B (Novozym 435) was the ideal catalyst. It seemed that the lipase-mediated hydrolysis method was better than the transesterification procedure.^251^

### 9.3. Cyclotriphosphazene–benzothiazole based analogues

**Scheme 95:**
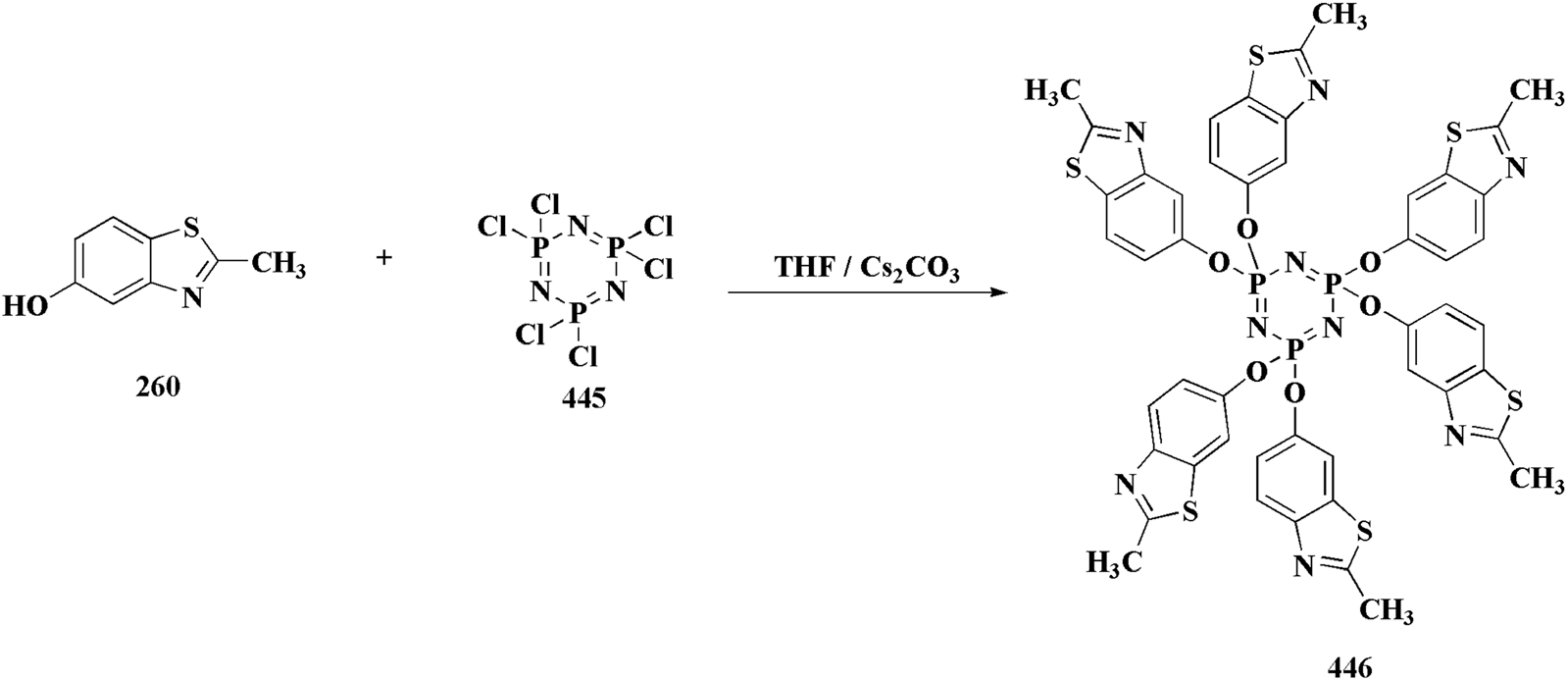
Synthesis of cyclotriphosphazene derivatives.

The cyclotriphosphazene derivatives 446 were prepared from the reactions of hexachlorocyclotriphosphazatriene 445 with 5-hydroxy-2-methylbenzothiazole 260 (Scheme 95). The fluorescence assets of these cyclophosphazene analogs 446 were evaluated in tetrahydrofuran solution.^252^

**Fig. 70:**
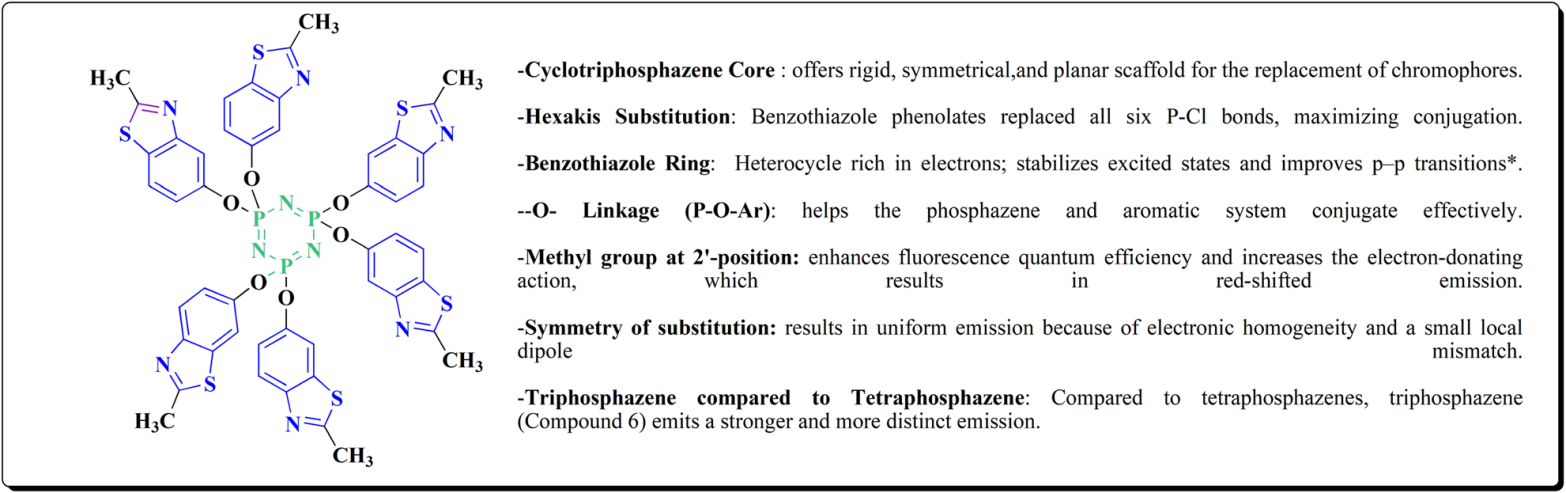
Structure–activity relationship of compound 446.

In comparison to the thiadiazole analogues, the benzothiazole-substituted cyclotriphosphazene based on triphosphazene core showed a noteworthy emission maximum and increased intensity of fluorescence emission. This implies that benzothiazole rings' π-rich aromaticity and advantageous electronicle interactions boost photoluminescence (Fig. 70).

Benzothiazole scaffold reduces non-radiative decay and produced a stronger emission, probably as a result of improved π-conjugation. Compared to tetraphosphazene analogues, triphosphazene cores tended to produce more distinct emission peaks, maybe as a result of tighter, symmetrical replacement that improved photophysical uniformity.

All results are measured in THF; aggregation or solvent–solute interactions may cause emission behavior to change in polar *versus* nonpolar solvents.^252^

**Scheme 96:**
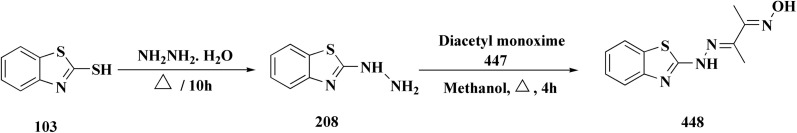
Synthesis substituted benzothiazol-2-yl hydrazine 448.

**Fig. 71:**
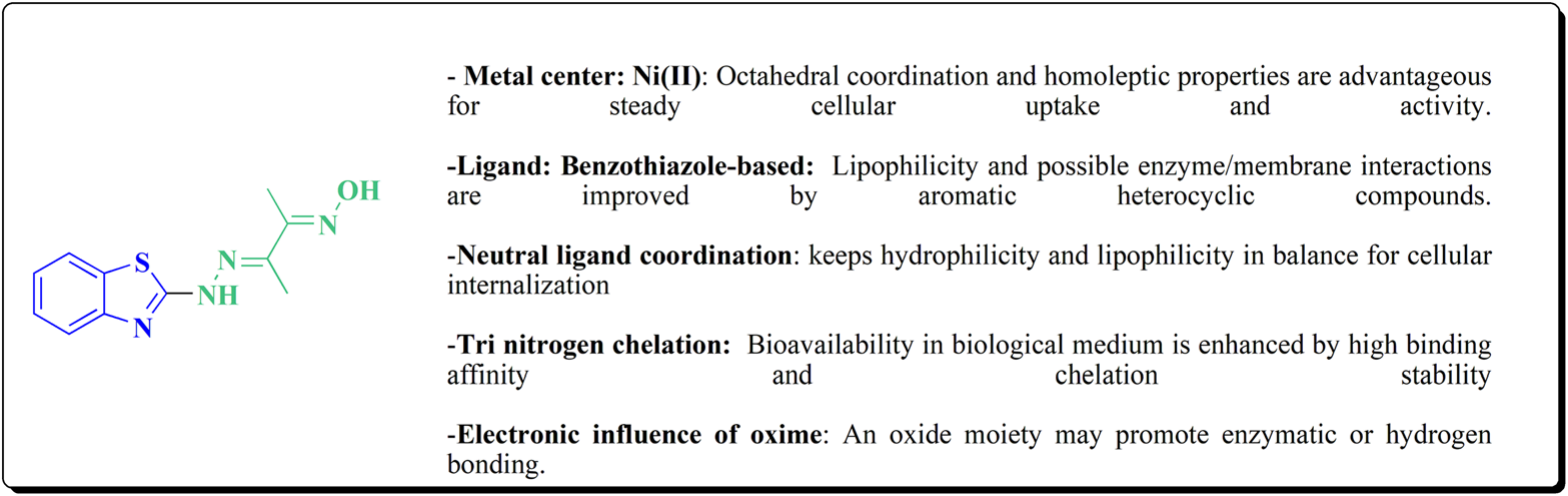
Structure–activity relationship of compound 448.

The 3-(hydroxyimino)-2-butanone-2-(1*H*-benzimidazol-2-yl)hydrazone and 3-(hydroxyimino)-2-butanone-2-(1*H*-benzothiazol-2-yl)hydrazine are synthesized *via* condensing 2-hydrazino-benzothiazole/benzimidazole with diacetyl monoxime (Scheme 96). The complexes have been investigated for their NCI-60 Human Cancer Cell Line anticancer screening. Sequently, the nickel complex of benzothiazole core is utilized for one dose growth inhibition screening. The complex has indicated highest growth inhibition over a Non-Small Cell Lung Cancer cell line EKVX. Additionally, the compounds were screened for their antifungal and antibacterial activities. The complexes have indicated promising antimicrobial activities (Fig. 71).^253^

### 9.4. Benzothiazoles fused with heterocyclic compounds

#### 9.4.1. Benzothiazoles fused with six-membered ring heterocycles

**Scheme 97:**
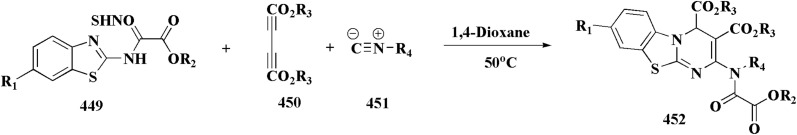
Synthesis of functionalized benzo[4,5]thiazolo[1,2-*a*]pyrimidines 452.

The synthesis of the tricyclic fused pyrimidine derivatives was shown in Scheme 90. A three-component cyclization using *N*-(benzothiazol-2-yl)amide, isocyanides and dialkyl acetylenedicarboxylates generating functionalized benzo[4,5]thiazolo[1,2-*a*]pyrimidine was reported (Scheme 97).^254^

**Fig. 72:**
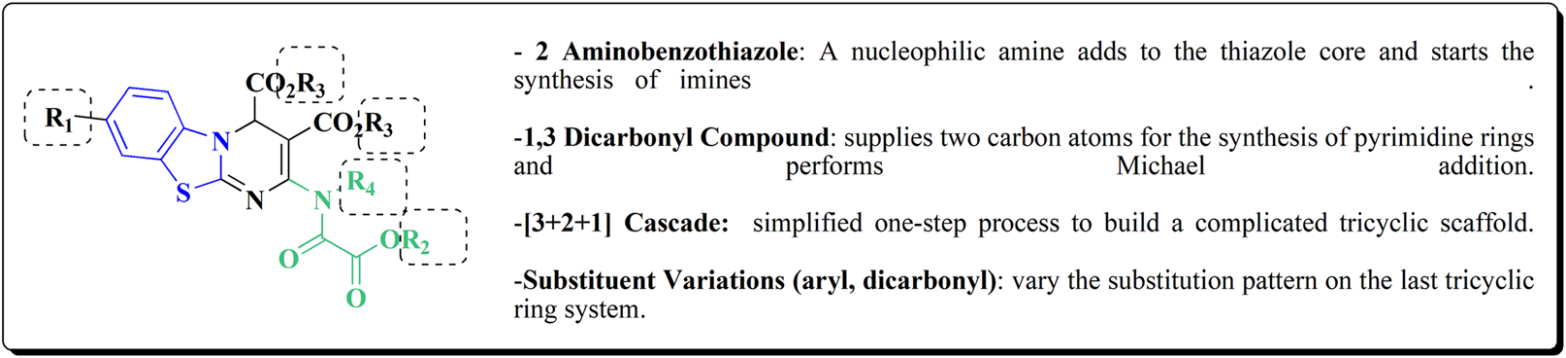
Structure of compound 452.

The choice of aryl glyoxal or 1,3 dicarbonyl components results in structural variation; replacement affects the final tricycle's yield and electronic nature. The structural insights provide helpful SAR-style reasoning for synthetic application, notwithstanding the lack of bioactivity reported (Fig. 72).^254^

**Scheme 98:**
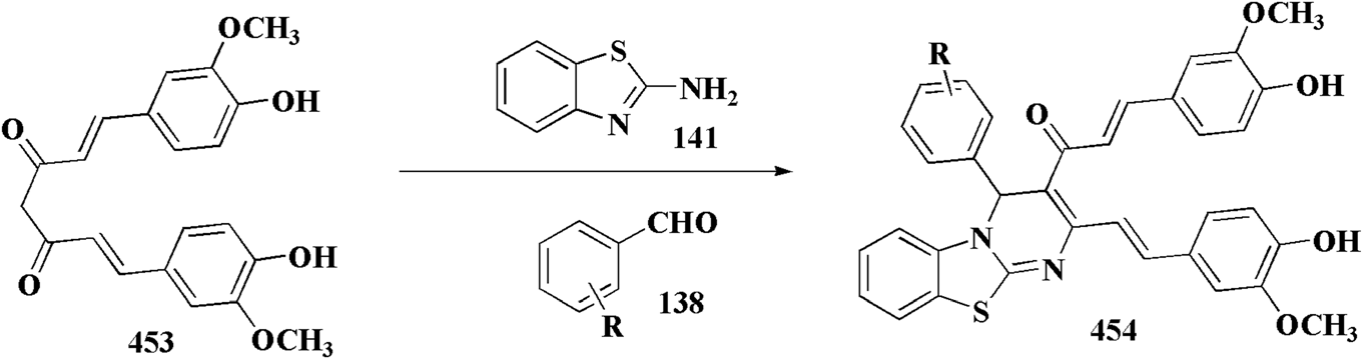
Synthesis of pyrimido[2,1-*b*]benzothiazole derivatives.

Pyrimido[2,1-*b*]benzothiazole 454 derivatives of curcumin have been prepared under solvent and solvent free conditions in microwave (Scheme 98). The synthesized compounds were tested for their anti-fungal activity against fungi and anti-bacterial activity against gram (+ve) and gram (−ve) bacteria.^255^

**Fig. 73:**
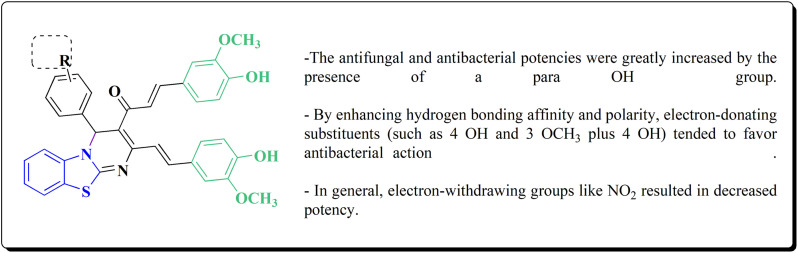
Structure–activity relationship of compound 454.

In microbial cells, membrane insertion and potential enzyme target binding are most likely caused by the benzothiazole–pyrimidine tricyclic core. The planar heterocycle and electronic distribution of the scaffold may facilitate interactions with nucleic acids or active site pockets in bacteria or fungi; the *para* OH substituent may allow for improved hydrogen bonding to microbial enzymes or cell wall/membrane components (Fig. 73).

#### 9.4.2. Benzothiazoles fused with five-membered ring heterocycles

**Scheme 99:**
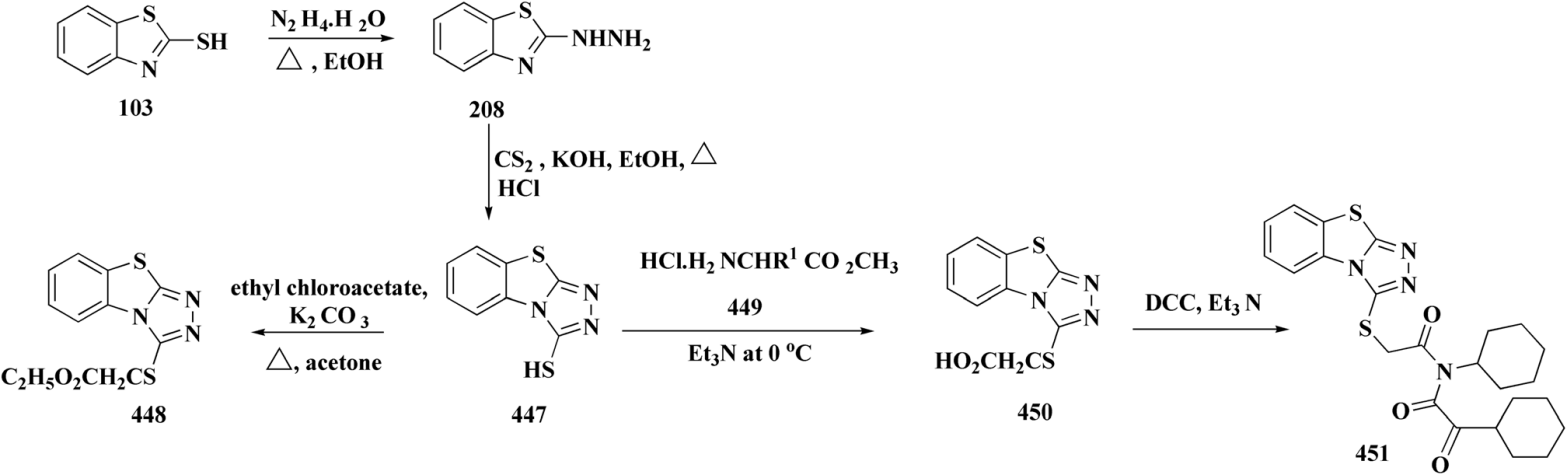
Synthesis of *s*-triazolobenzothiazolylthioacetyl/propionyl amino acid derivatives.

*s*-Triazolobenzothiazolylthioacetyl/propionyl amino acid derivatives were prepared as outlined in Scheme 99. The synthesized compounds were screened for their anti-fungal activity against *Candida albicans* and *Aspergillus flavus*. Compounds 447 were found to have high activity against *C. albicans* as compared to fluconazole at 100 mg mL^−1^.^256^

**Fig. 74:**
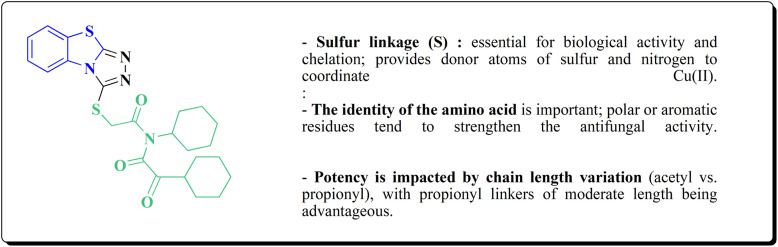
Structure–activity relationship of compound 451.

The triazolobenzothiazole scaffold targets fungal melanin production or membrane integrity by imitating the well-known fungicide tricyclazole. The amino acid moiety and S Linker may improve target selectivity and cellular absorption. Selective binding to fungal enzymes or cell wall constituents may be facilitated by fluorency or polar side chains. These compounds present possible triazole-drug analogs for the development of antifungal agents (Fig. 74).^256^

**Scheme 100:**
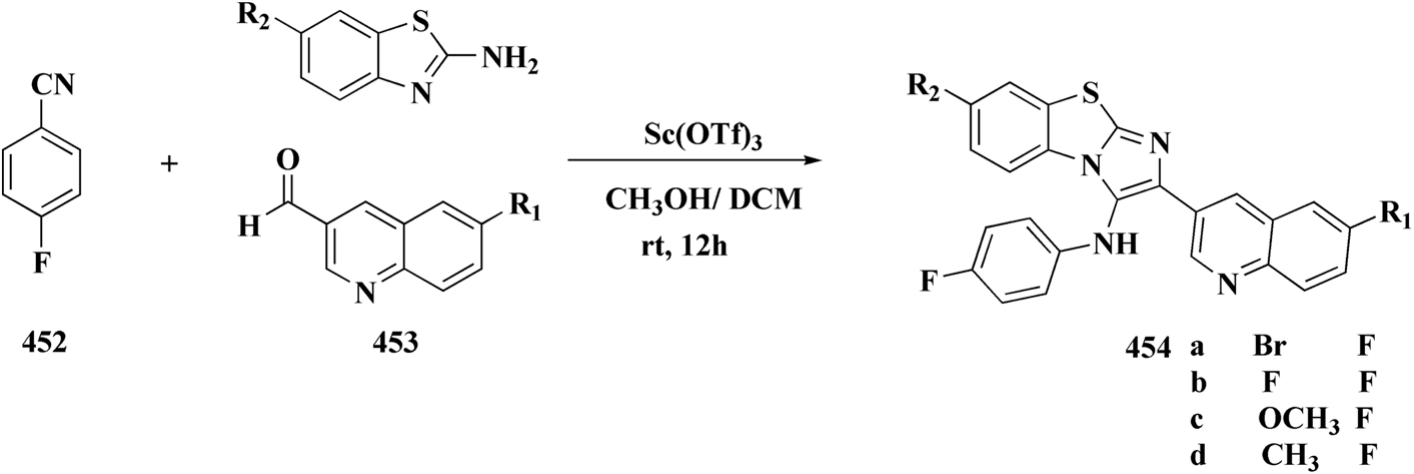
Synthesis of imidazobenzothiazole–quinoline conjugates.

**Scheme 101:**
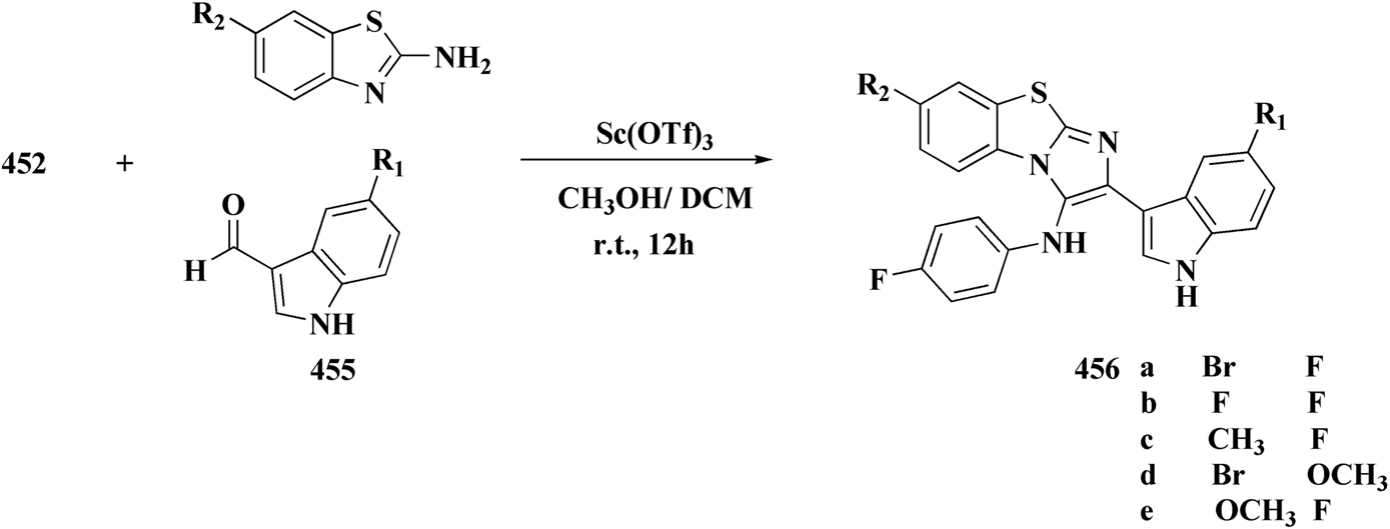
Synthesis of synthesis of imidazobenzothiazole–indole conjugates.

Compound were reacted with quinoline carboxaldehyde derivatives 453 followed by reaction with the fluorophenyl isocyanide 452 to afford the polycyclic compounds 454. The reaction of aminobenzothiazole with indole carbaldehyde 445 followed by the addition of isocyanide 452, afforded compounds 456 (Schemes 100 & 101).

**Fig. 75:**
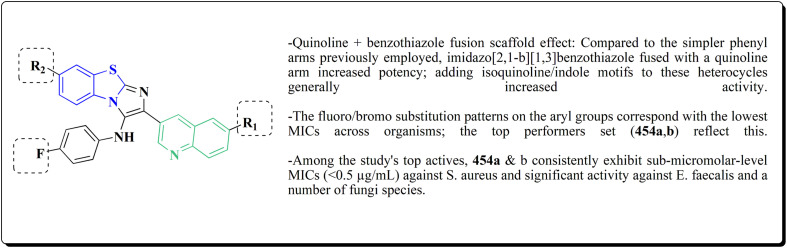
Structure–activity relationship of compound 454.

**Fig. 76:**
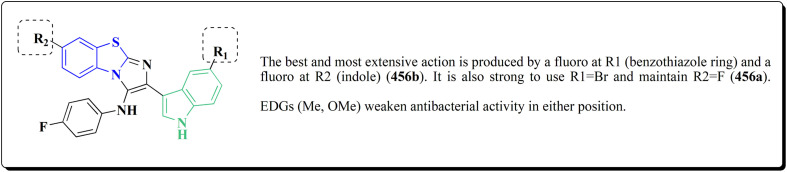
Structure–activity relationship of compound 456.

Their anti-microbial potencies were estimated against several Gram (+ve), Gram (−ve) bacteria and fungi. Compounds 454a, 454b, 456a and 456b showed strong inhibition of the estimated fungal and bacterial strains comparable to control antibiotics cefixime and amoxicillin and the anti-fungal agent fluconazole (Fig. 75 and 76).^257^

**Scheme 102:**
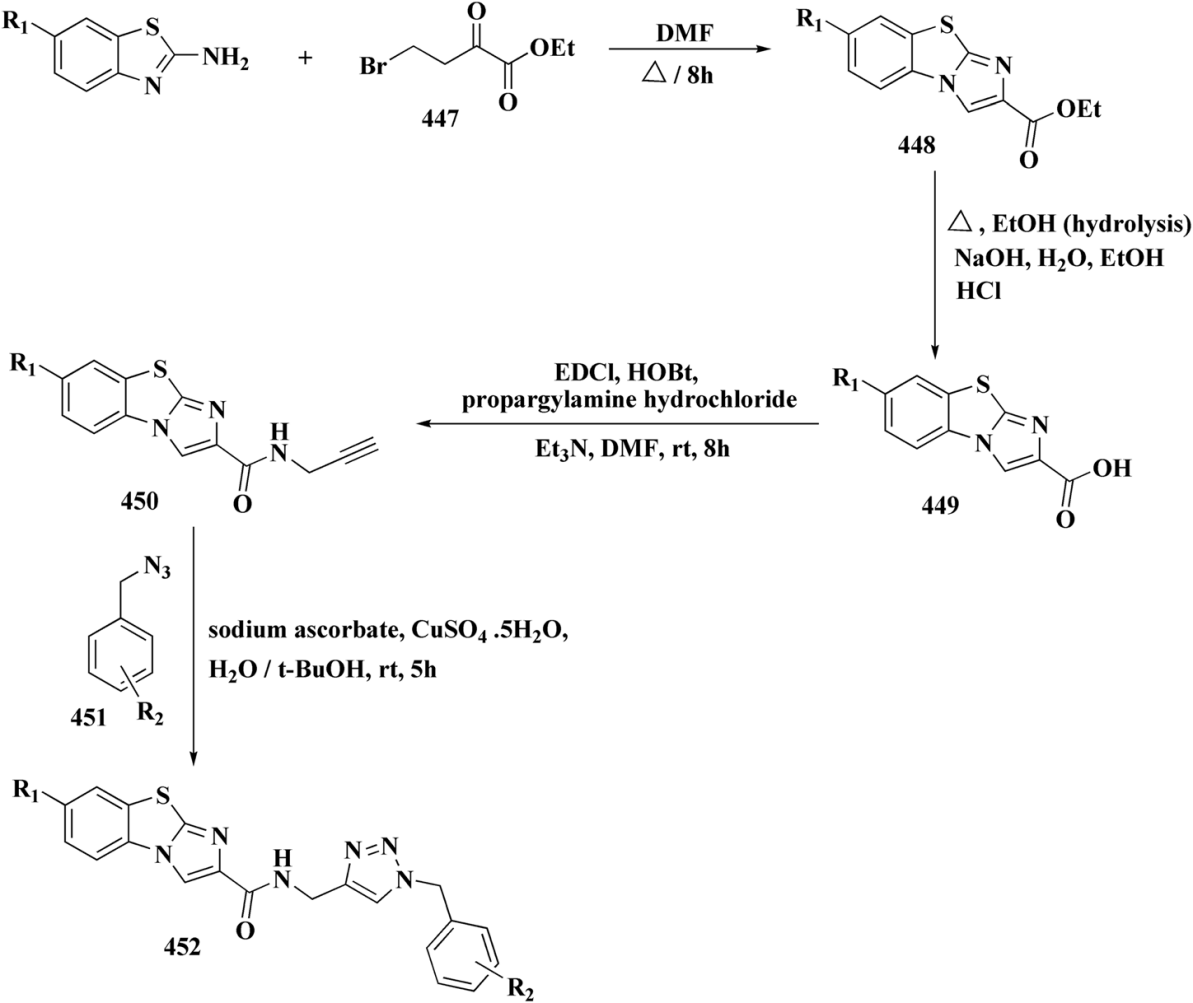
Synthesis of 1,2,3-triazolo linked benzo-imidazothiazole conjugates.

The 1,2,3-triazolo linked benzo-imidazothiazole conjugates 452 were prepared as outlined in Scheme 102.^258^ Refluxing 2-aminobenzothiazoles derivatives with the ethylbromopyruvate 447 in DMF generated compound 448, which upon sodium hydroxide mediated ester hydrolysis afforded compound 449. These were reacted with propargylamine hydrochloride under nitrogen atmosphere to access the terminal alkynes 450. The targeted compound 452 was afforded by exposing benzo-imidazothiazole terminal alkynes 450 to the benzyl azides 451.

Compounds 452 tested for their cytotoxic potency against some human cancer cell lines. Preliminary results showed that compounds 452 (R^1^ = H, R^2^ = 4-F) and 452 (R^1^ = Me, R^2^ = 4-F) displayed effective antiproliferative effect against human breast cancer cells (MCF-7) with IC_50_ values of 0.60 and 0.78 μM.^258^

**Fig. 77:**
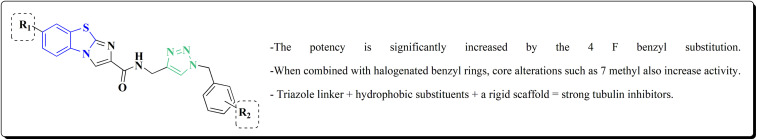
Structure–activity relationship of compound 452.

The assessed cytotoxic activity of a novel class of 1,2,3-triazolo linked benzo[*d*]imidazo[2,1-*b*]thiazole conjugates was reported. The human breast cancer cell line (MCF-7) was significantly cytotoxically affected by conjugates 452 (R^1^ = H, R^2^ = 4-F) and 452 (R^1^ = Me, R^2^ = 4-F) (Fig. 77). Based on these scaffolds, the SAR offered a useful insight for generating better leads. These conjugates result in cell cycle arrest at the G_2_/M phase, according to the flow cytometric study. The existence of elevated tubulin and cyclin B1 protein levels in the soluble portion of cells is in good agreement with the suppression of tubulin polymerization. Additionally, they successfully prevent microtubule assembly and interfere with the architecture of microtubules in breast cancer cells. These conjugates bind at the tubulin's colchicine site, according to molecular docking studies. Annexin V FITC test and mitochondrial membrane potential are linked to the induction of apoptosis. These conjugates may therefore be regarded as promising scaffolds that aid in the discovery of novel leads for breast cancer chemotherapy.^258^

### 9.5. Benzothiazole complexes

**Scheme 103:**
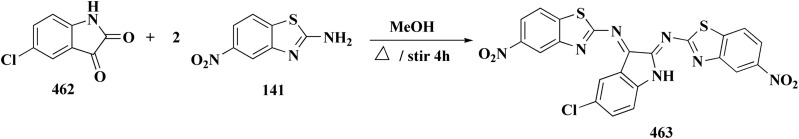
Synthesis of indoline-2,3-diylidene-benzothiazole conjugates.

New bioactive 5-chloro isatin based Schiff base ligands 463 derived from 5-nitrobenzo[*d*]thiazol-2-amine and 5-nitrothiazol-2-amine and their metal complexes have been prepared (Scheme 103).

**Fig. 78:**
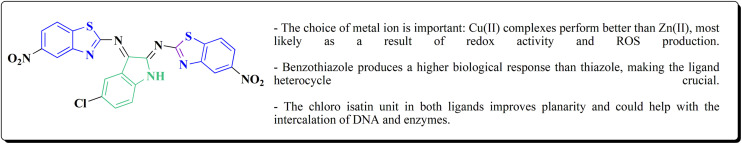
Structure–activity relationship of compound 463.

*In vitro* antibacterial investigations were performed against various bacterial strains and scavenging activity against standard control at several concenterations unfolded pronounced anti-bacterial and radical scavenging activities of the metal complexes comparable to free ligands. Additionaly, *in vitro* cytotoxicity of ligands and its metal complexes was screened on MCF7, HepG2, and HeLa cell lines and normal cells (peripheral blood mononuclear cells, PBMC). The antiproliferative outcomes showed that metal complexes display superior activity comparable to free ligands 463 where metal complexes of 5-chloro isatin-linked benzothiazole motif 463 are showed potency as chemotherapeutic agents (Fig. 78).^259^

The Cu(ii) core and the benzothiazole-containing Schiff-ligand, supported by planar isatin architecture, are necessary for optimal activity.^259^

**Scheme 104:**
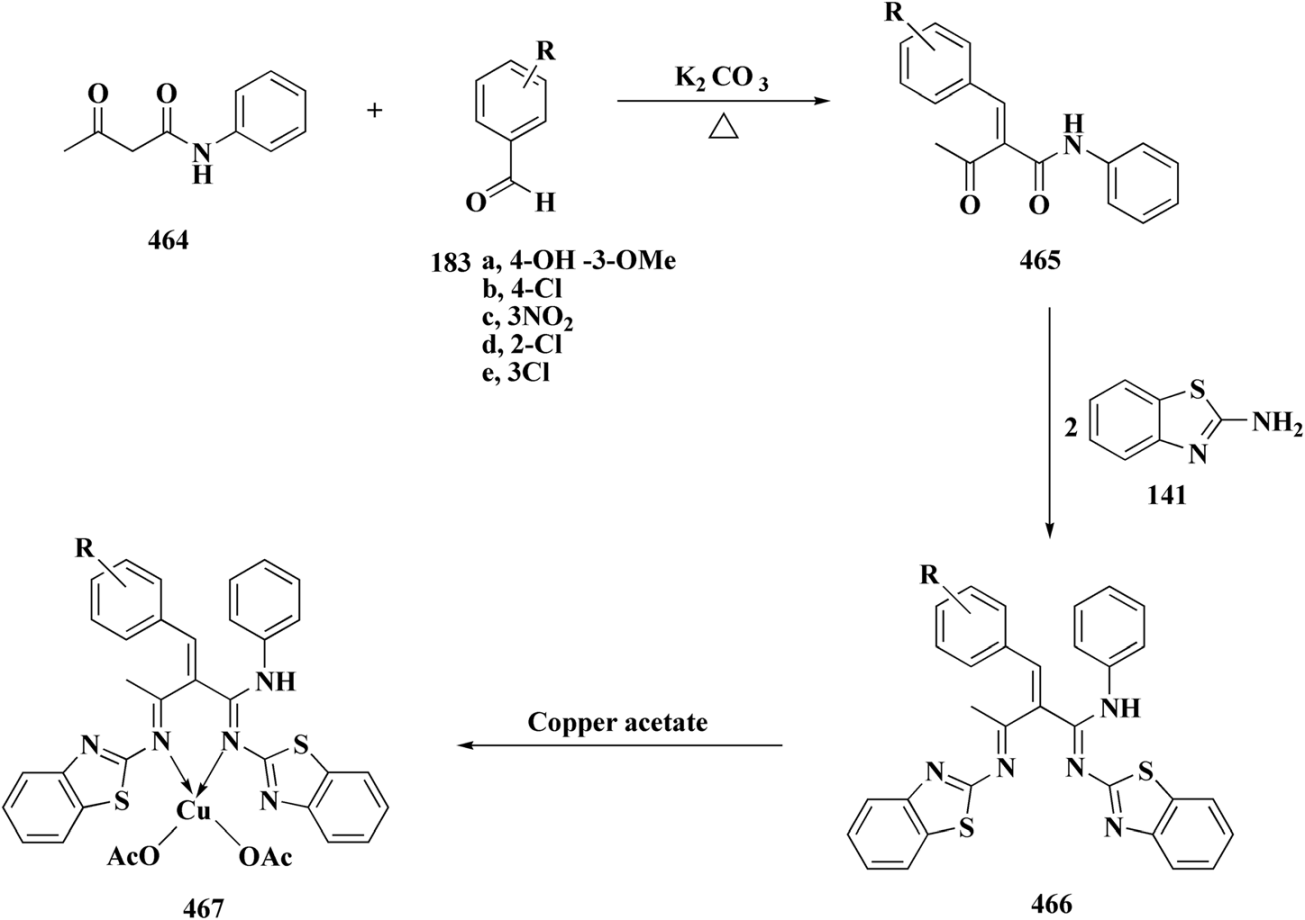
Synthesis of copper complexes of 2-aminobenzothiazole derivatives.

New copper complexes of 2-aminobenzothiazole derivatives were prepared *via* the condensation of 2-aminobenzothiazole and knoevenagal condensate acetoacetanilide (Scheme 104).^260^

By condensation of the Knoevenagel products with two aminobenzothiazole, followed by a reaction with Cu(ii) ions, a series of copper(ii) complexes were synthesized.

**Fig. 79:**
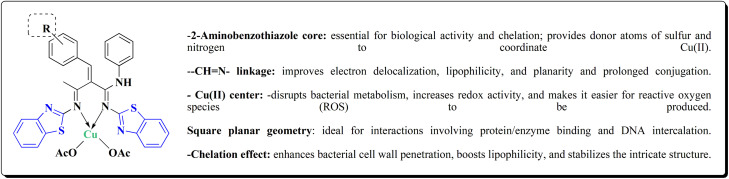
Structure–activity relationship of compound 467.

A variety of Gram-positive and Gram-negative microorganisms were screened for using antibacterial agents. When compared to their free ligands, all Cu complexes demonstrated increased antibacterial activity. For bioactivity, Cu(ii)–ligand chelation is essential (Fig. 79). The *para*-position of a methyl group improves electron delocalization throughout the π-system, ligand basicity, and Cu(ii) binding.^260^

**Scheme 105:**
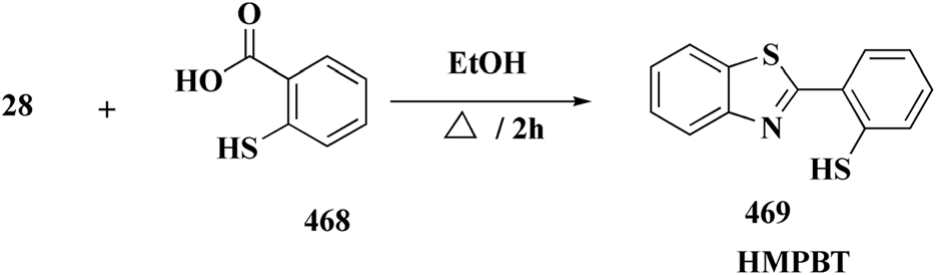
Synthesis of (HMPBT) ligand.

Novel ligand (HMPBT) ligand is synthesized through the reaction of 2-mercaptoaniline with 2-mercaptobenzoic acid (Scheme 105). The estimated metal complexes and ligand were screened for their *in vitro* anti-microbial potencies against various kinds of bacterial and fungal strains. The results assert on the examined compounds as a highly promising fungicides and bactericides.^261^

**Fig. 80:**
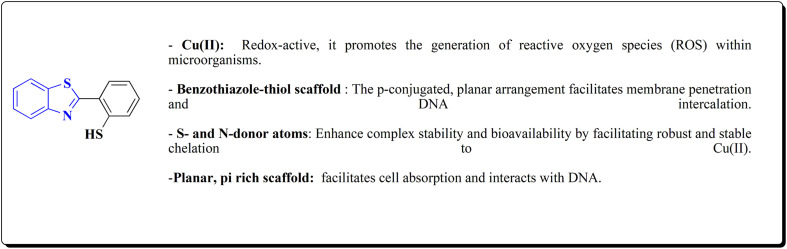
Structure–activity relationship of compound 469.

Direct DNA interference and oxidative stress are probably both involved in the mechanism. The 2-(2′-mercaptophenyl)benzothiazole copper(ii) complex had the most potent antibacterial action against *B. subtilis*, *C. albicans*, *S. aureus*, and *E. coli* (Fig. 80). Viscosity, gel electrophoresis, and UV-Vis titration studies demonstrated a significant DNA-binding affinity.

Several processes are responsible for these complexes' antimirobial action. While the Cu(ii) center contributes to oxidative stress by producing reactive oxygen species (ROS), which causes oxidative damage of proteins, lipids, and DNA, the lipophilic aspect of the ligand structure encourages rupture of microbial membranes. Additionally, increased cellular uptake and improved bioavailability are made possible by a synergistic interaction between the metal ion and the ligand.

An intercalative binding mode was suggested by the hypochromic effect and minor red shift observed upon DNA addition in the UV-Vis absorption titration. Viscosity experiments, which showed that a rise in DNA viscosity verified base pair insertion, provided more evidence for this. DNA cleavage was directly demonstrated by gel electrophoresis, with Cu(ii)-complex showing especially strong action.^261^

**Scheme 106:**
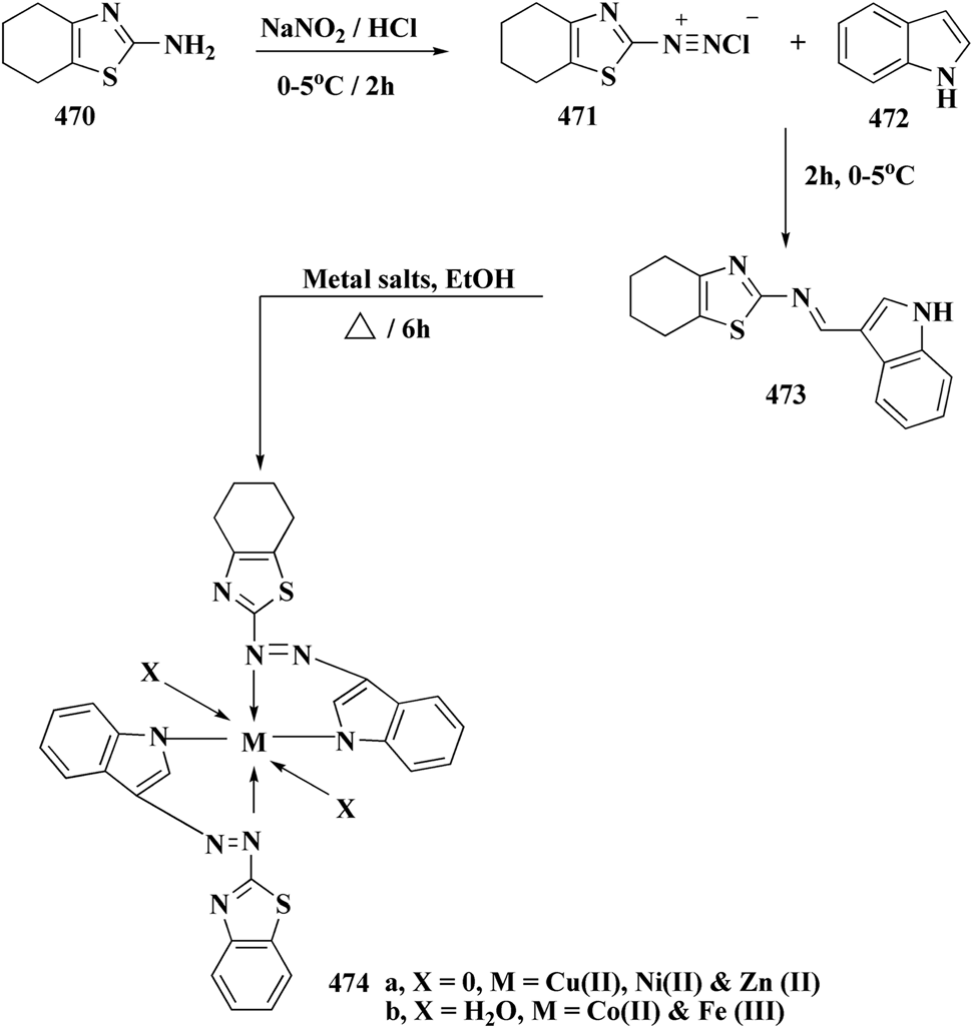
Synthesis of 2-(1*H*-indol-3-yldiazenyl)-4,5,6,7-tetrahydro-1,3-benzothiazole complexes.

The bioactive metal complexes were prepared from the tetrahydro-1,3-benzothiazole (Scheme 106). The synthesized compounds displayed antimicrobial potency against tested pathogens. The antimycobacterial potency of the synthesized compounds was studied and all the metal chelates showed higher activity than the free ligand.^262^

**Fig. 81:**
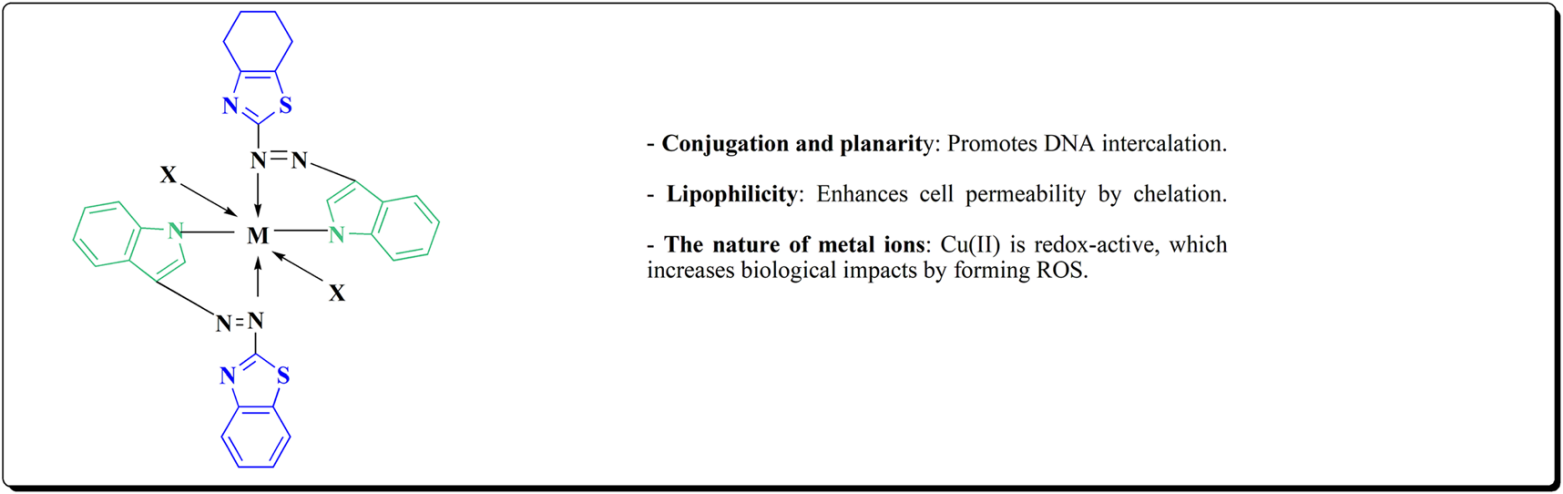
Structure–activity relationship of compound 474.

The azo-linked indole–benzothiazoline ligand and its metal(ii) complexes (Co(ii), Ni(ii), Cu(ii), and Zn(ii)) are the main subjects of the investigation. Important mechanistic details and findings: including the mode of ligand binding in which the ligand forms a bidentate chelate ring with the metal ions by coordinating with azo nitrogen (–N=N–) and thiazole nitrogen. Coordination was indicated by FTIR spectra, which verified shifts in *v*(N=N) and the disappearance/shift of NH and C=N peaks (Fig. 81).

The Cu(ii) complex's capacity to produce reactive oxygen species (ROS) through redox cycling (Cu^2+^/Cu^+^) was responsible for the whole DNA cleavage. The mechanism most likely uses superoxide or hydroxyl radicals to cause oxidative DNA scission.

The antimicrobial mechanism can be explained by the chelation theory, often known as Tweedy's theory, provided an explanation for the metal complexes' increased activity in comparison to the free ligand: chelation increases lipophilicity by decreasing metal ion polarity, which improves complexes' membrane permeability and provides improved intracellular access to biological targets like enzymes or DNA.^262^

**Scheme 107:**
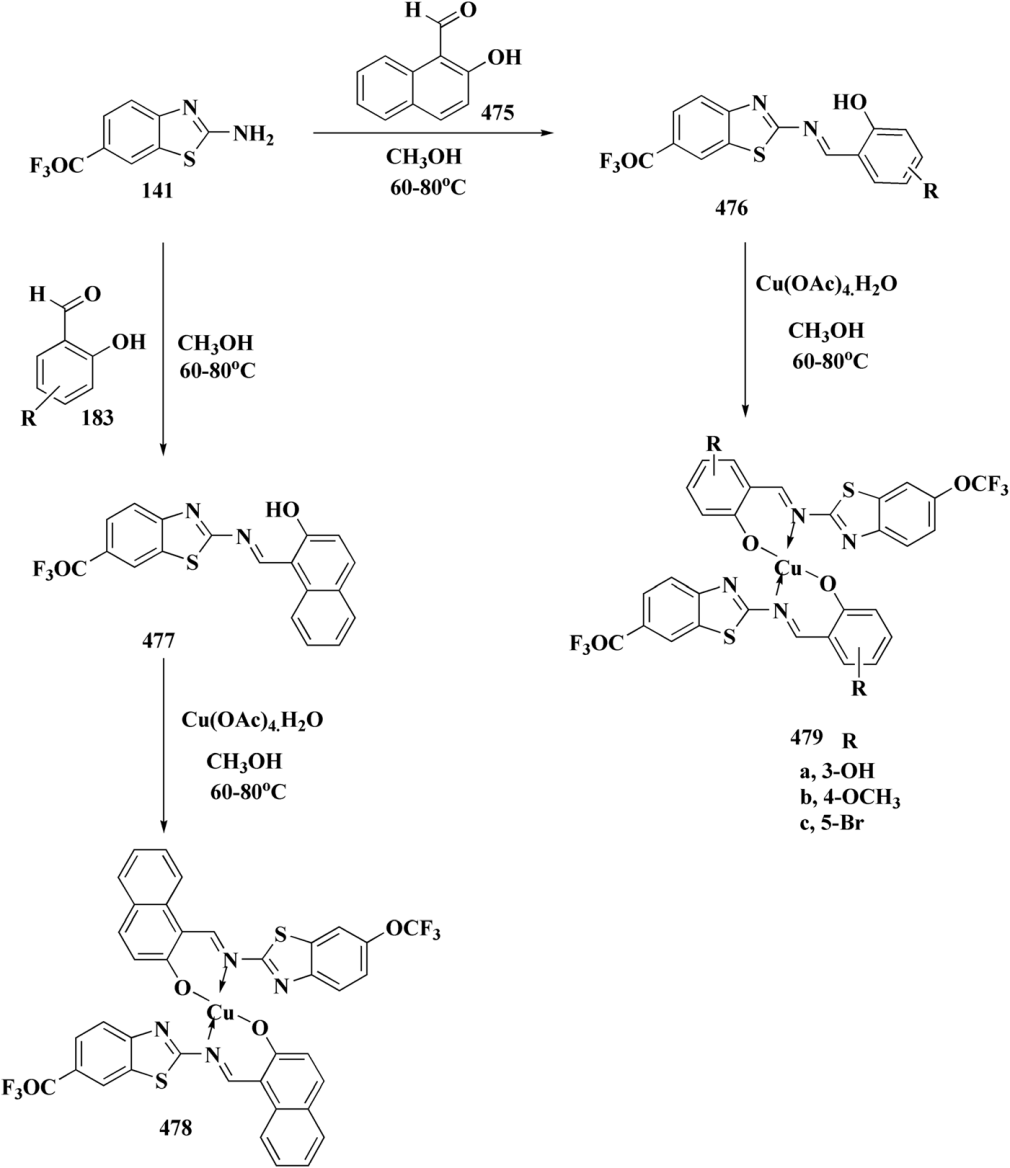
Synthesis of benzothiazole-based phthalonitrile analogs & tetra substituted metal free phthalocyanines.

**Fig. 82:**
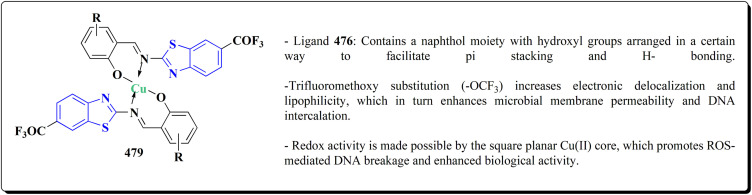
Structure–activity relationship of compound 479.

The Schiff base ligands, and their binary copper(ii) complexes 478 & 479 have been prepared (Scheme 107). After screening the ligands and their Cu(ii) complexes against bacterial strains for anti-microbial potency and it was observed that all Cu(ii) complexes are more active than corresponding ligands (Fig. 82).^263^

The most active complex in terms of DNA binding and cleavage, as well as antibacterial activity, was complex 479 (Cu(L^2^)_2_). Viscosity measurements, fluorescence titration, and UV-Vis all confirm intercalative binding to CT-DNA, while ifs strongest π–π stacking interactions with base pairs, as evidenced by the highest binding constant and cleavage efficiency among the four complexes. Compared to the ligand and the other complexes, complex 479 shown higher potency against bacterial (*e.g.*, *E. coli*, *P. aeruginosa*, *B. amyloliquefaciens*, *S. aureus*) and fungal (*S. rolfsii*, *M. phaseolina*) strains.^263^

**Scheme 108:**
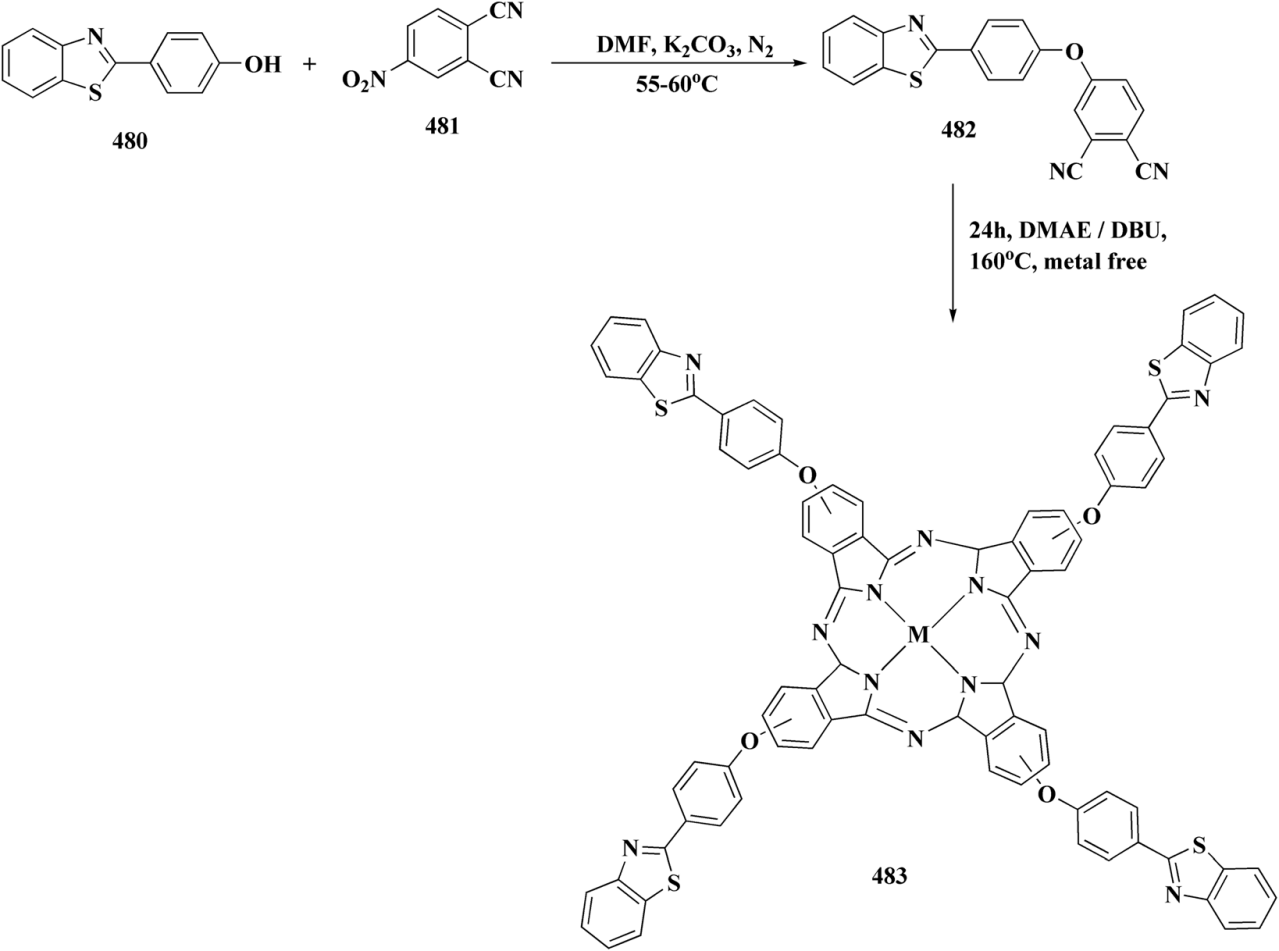
Synthesis of metal-free & metallophthalocyanines.

Benzothiazole-based phthalonitrile analog and its peripherally tetra substituted metal free phthalocyanines have been prepared (Scheme 108). Their fluorescence quenching properties by the addition of 1,4-benzoquinone was also investigated.

The synthesis of 4-(1,3-benzothiazol-2-yl)phenoxy substituted phthalonitrile 482 and its target metal-free and metallophthalocyanines is indicated.^264^

Each tetrakis substituted phthalocyanine had a rigid benzothiazole moiety connected by a 1,4 benzene spacer. This design reduces aggregation and promotes orderly self-assembly behavior by providing steric impediment while maintaining adequate stiffness. The benzothiazole units have two functions: they are directing groups for supramolecular organization and electronic modulators (*via* S and N atoms).

The compounds' red/near-IR fluorescence emissions ranged from mild to strong; the benzothiazole substituents' steric blocking results in less π stacking, which improves the fluorescence quantum yield. The Zn(ii) complex was the most appropriate for optical and photophysical applications due to its intense fluorescence, high quantum yield, and decreased aggregation. The Cu(ii) complex was not the best for fluorescence-based applications, but it probably showed the strongest photochemical quenching (because of paramagnetism).

By preventing planar π–π stacking, the benzothiazole arms preserve monomeric absorption bands and molecular separation. The benzothiazole spacer modifies electron distribution, improving photophysical characteristics including excited-state lifetime and absorption cross-section *via* prolonged conjugation. Self-assembly took place in thin films or aggregates, enabling ordered nano-systems with desired optical properties.

Benzothiazole–phthalocyanine hybrids show promise for photonic applications such as optical limiting and nonlinear absorbers; thin-film optoelectronics, where performance is improved by ordered stacking; and possible scaffolds for supramolecular structures or nanoparticle conjugation.^264^

**Scheme 109:**
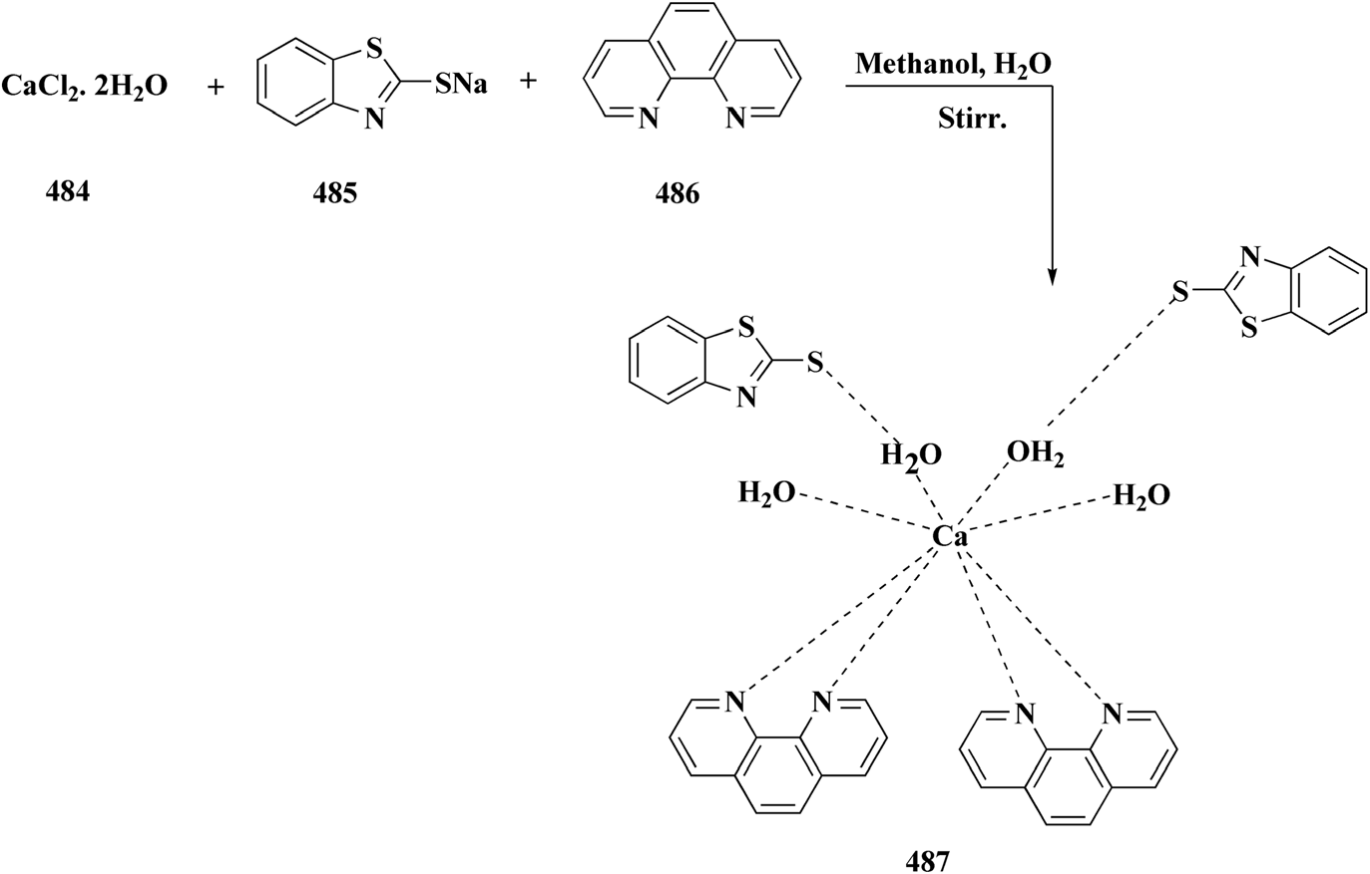
Synthesis of Ca-complex 487.

A novel Ca-complex has been prepared through the reaction of sodium 2-mercaptobenzothiazole, 1,10-phenanthroline and calcium chloride (Scheme 109). The complex was evaluated against various bacterial strains. The complex exhibited good anti-bacterial activity against *Acinetobacter baumanni* and remarkable anti-bacterial activity against *Pseudomonas aeruginosa* as compared to levofloxacin 265.

**Fig. 83:**
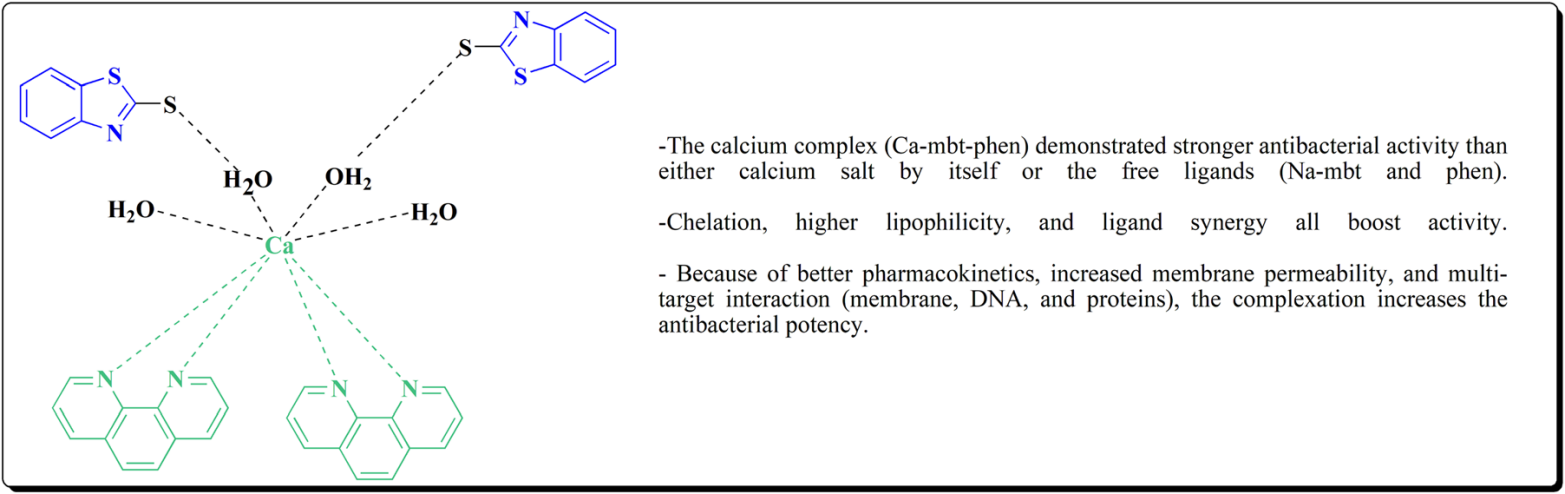
Structure–activity relationship of compound 487.

The synthesis of a new calcium(ii) compound and its biological assessment was acccomplished using the sodium 2-mercaptobenzothiazole (Na-mbt), is a bidentate ligand that coordinates *via* nitrogen and sulfur, and 1,10-phenanthroline (phen) which is a planar aromatic bi-dentate ligand with the ability to intercalate DNA and promote biological activity. Mechanism of action most likely comprises enzyme inhibition (mbt ligand), membrane rupture, and DNA binding (phen). The [Ca(mbt)(phen)] complex is the most active molecule because of its optimal metal–ligand geometry and dual ligand effects (Fig. 83).

In order to examine the impact of nanoparticle morphology on photodynamic treatment (PDT) performance, a study presents the synthesis of glycosylated zinc phthalocyanine (ZnPc) coupled to gold nanoparticles (AuNPs) with a variety of geometries (spherical, rod-shaped, and star-shaped).

**Scheme 110:**
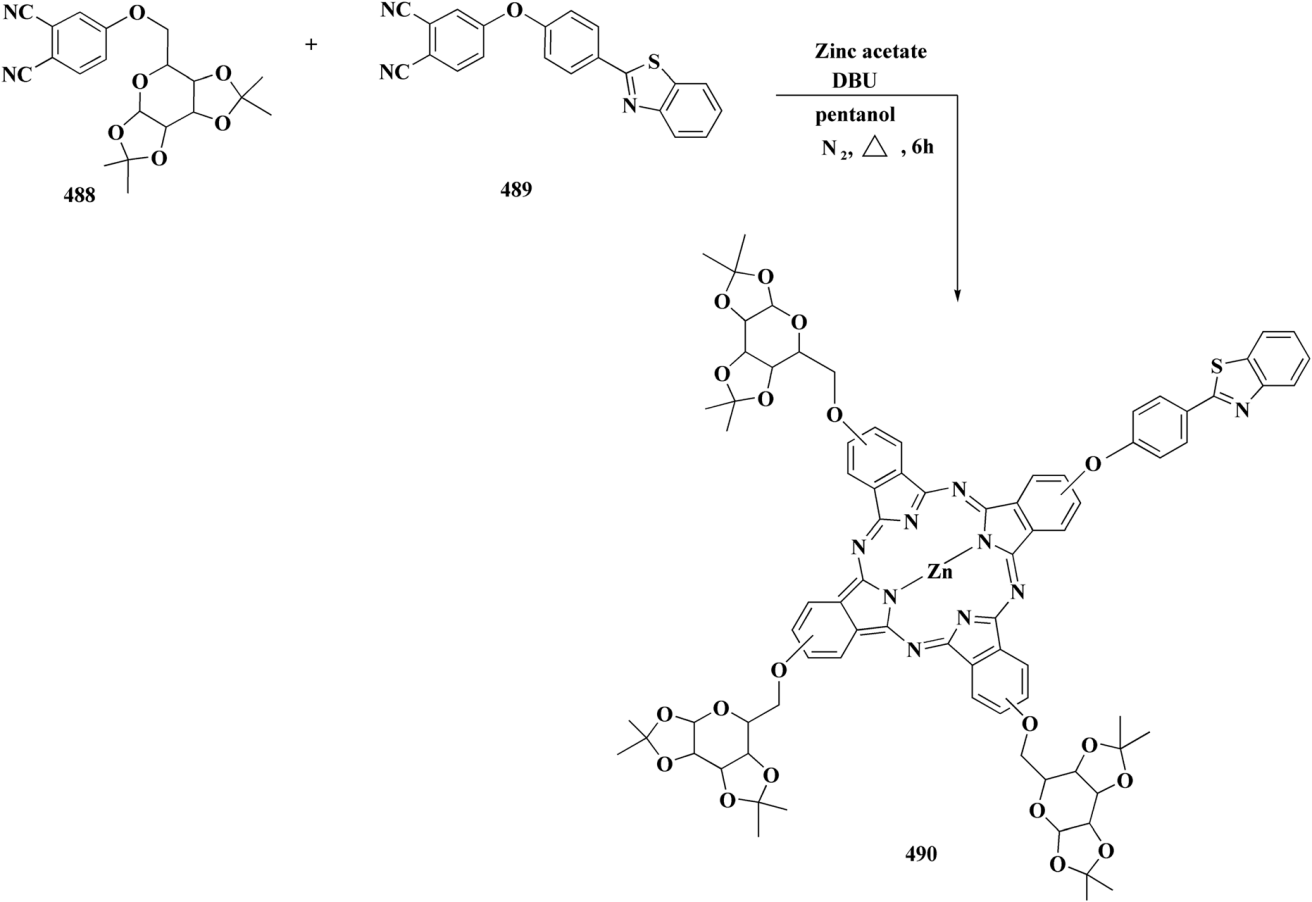
Synthesis of the complex 490.

**Scheme 111:**
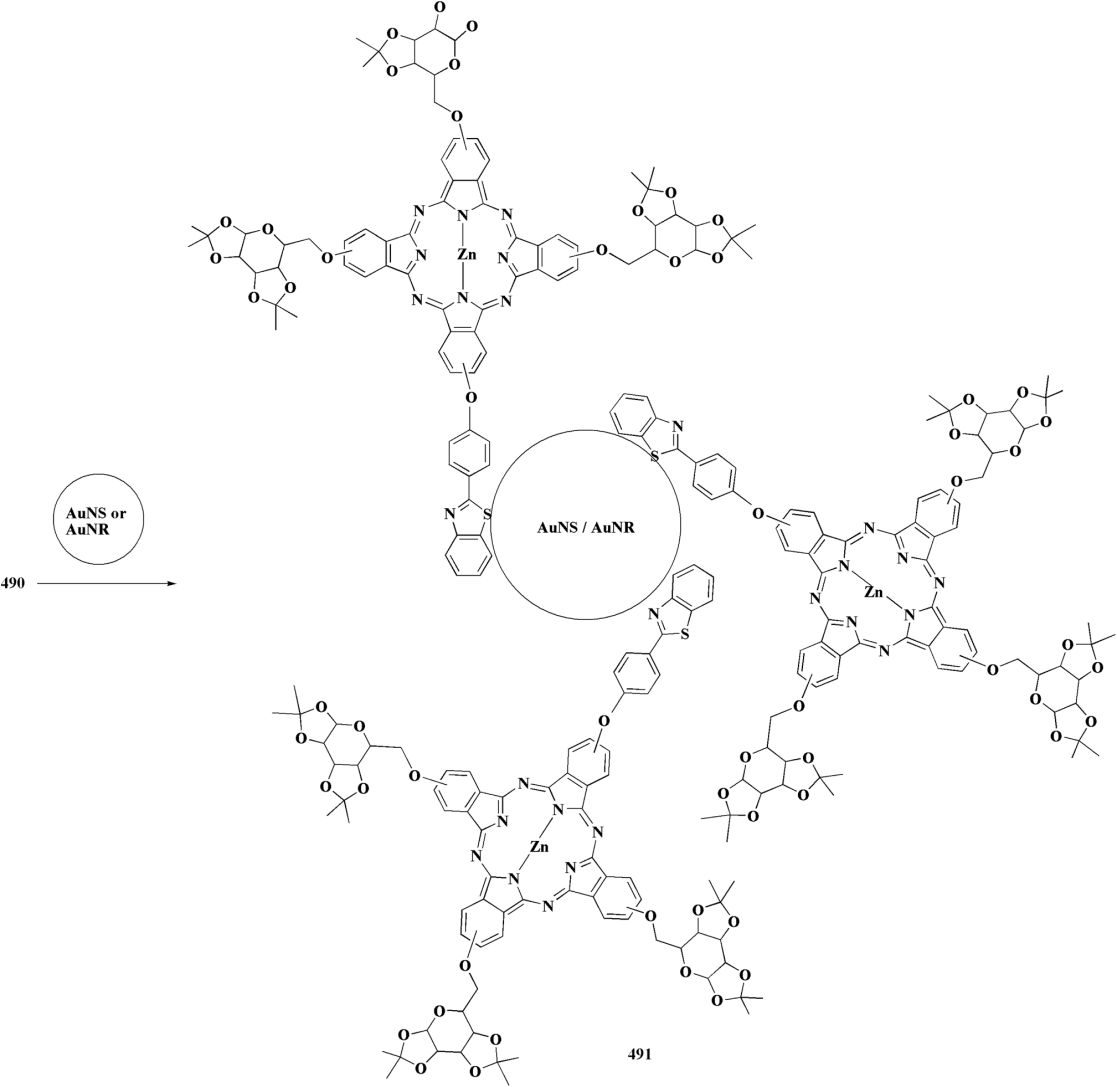
Synthesis of gold nanospheres (AuNS) and nanorods (AuNR).

**Fig. 84:**
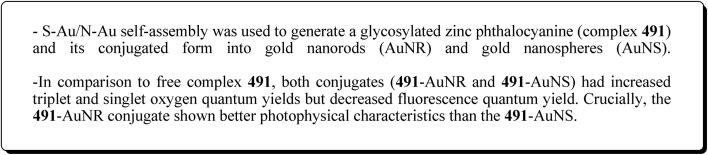
Structure–activity relationship of compound 491.

The synthesis of complex 490 and its linkage to gold nanoparticles (AuNPs) of various shapes *via* S–Au/N–Au self-assembly was accomplished in Schemes 110 & 111. The conjugates of complex 490 with both gold nanospheres (AuNS) and nanorods (AuNR), exhibited decreased fluorescence quantum yield with corresponding enhanced singlet and triplet quantum yields comparable to complex 490 alone, however 491-AuNR indicated enhanced behavior than 491-AuNS. Complex 491 revealed comparatively low *in vitro* dark cytotoxicity against the epithelial breast cancer cells with cell survival greater than eightyfive percent at conc. ≤160 μg mL^−1^ (Fig. 84).^266^

Cell survival >85% at doses <160 μg mL^−1^ indicated that free complex 491 exhibited comparatively low dark cytotoxicity toward epithelial breast cancer cells. However, its PDT efficacy was decreased, most likely as a result of aggregation. In contrast, at doses ≥40 μg mL^−1^, 491-AuNR reached 50% cell viability. For 491-AuNS to have the same impact, larger amounts (≥80 μg mL^−1^) were needed. Since nanorods absorb more light at 680 nm than nanospheres do, photothermal effects have been identified as the reason for the higher activity of 491-AuNR.^266^

## 10. Synthetic strategies for novel anti-viral benzothiazoles

### 10.1. Benzothiazoles linked with heterocyclic compounds

**Scheme 112:**
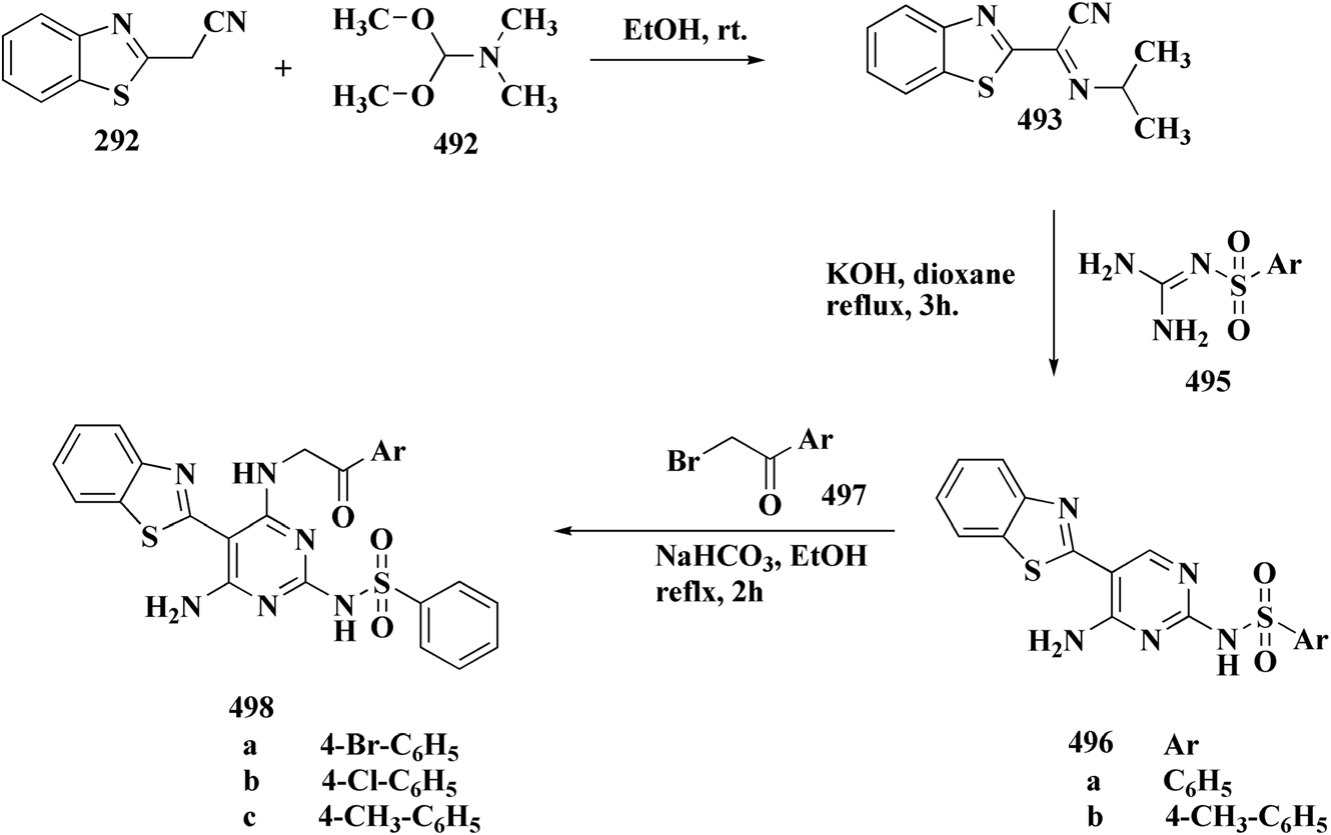
Synthesis of substituted (pyrimidin-2-yl)benzenesulfonamide.

The antiviral activity of a novel series of substituted 2-pyrimidylbenzothiazoles with either sulfonamide groups or an amino substituent at the C2 position of the pyrimidine ring was assessed. The core ring system was synthesized by reacting guanidine or *N*-arylsulfonated guanidine with different ylidene benzothiazole derivatives by a Michael addition process (Scheme 112).

A plaque reduction assay against a panel of viruses, including HSV-1, CBV4, HAV (HM175 strain), HCVcc (genotype 4), and HAdV7, was used to evaluate the synthetic compounds' antiviral activity. Notably, compounds 498a & 498b showed considerable action against HSV-1, with selectivity index (SI), IC_50_, and CC_50_ values higher than those of acyclovir, and viral inhibition rates ranging from 70% to 90%.

**Fig. 85:**
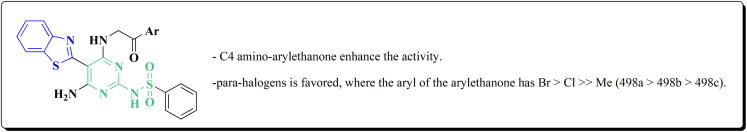
Structure–activity relationship of compound 498.

The promise of compound 498a as a prospective lead antiviral drug was highlighted by its remarkable broad-spectrum antiviral efficacy against all five tested viruses. Additionally, with IC_50_ values ranging from 4.87 to 10.47 μg mL^−1^, the strongest anti-HSV-1 compounds—498a, & 498b also inhibited Hsp90α. Curiously, these compounds increased antiviral potency when combined with acyclovir, lowering IC_50_ values compared to acyclovir alone. Molecular docking studies demonstrated that compounds 498a & 498b have favorable binding interactions within the Hsp90α active site, confirming their dual function as Hsp90α inhibitors and antiviral agents (Fig. 85).^267^

**Scheme 113:**
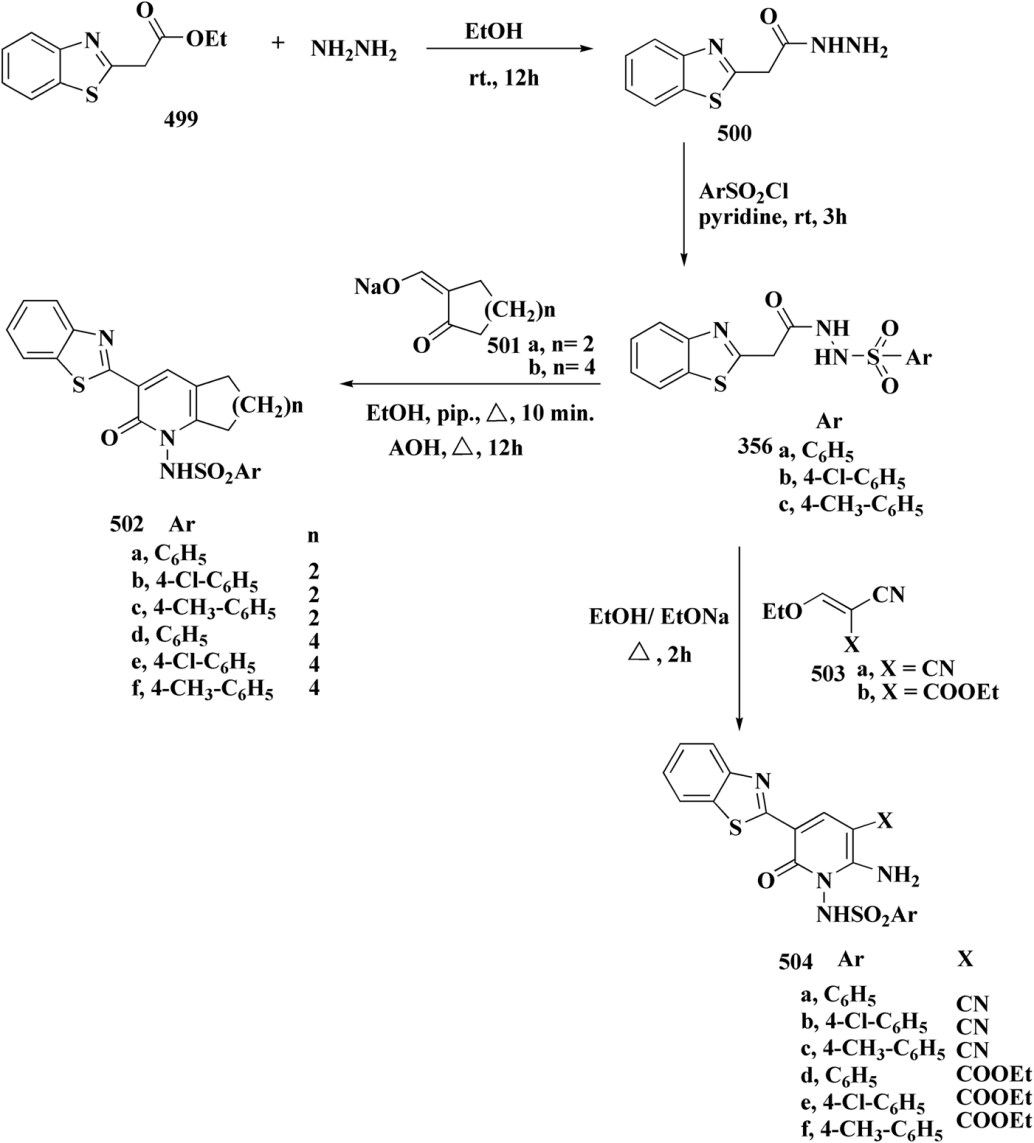
Synthesis of arylsulfonamides.

By reacting benzothiazole sulfonylhydrazide with the sodium salts of hydroxymethylene cycloalkanones, unsaturated ketones, and ethoxymethylene analogues, a new series of benzothiazole-linked *N* sulfonamide 2-pyridone derivatives was synthesized (Scheme 113). Five compounds—502a, 502c, 502e, 502f, and 504a—achieved above 50% viral suppression in *in vitro* antiviral studies against HSV 1, HAV HM175, HCVcc genotype 4a, CBV4, and HAdV7. The selectivity indices and CC_50_ and IC_50_ values for these lead compounds were computed.

**Fig. 86:**
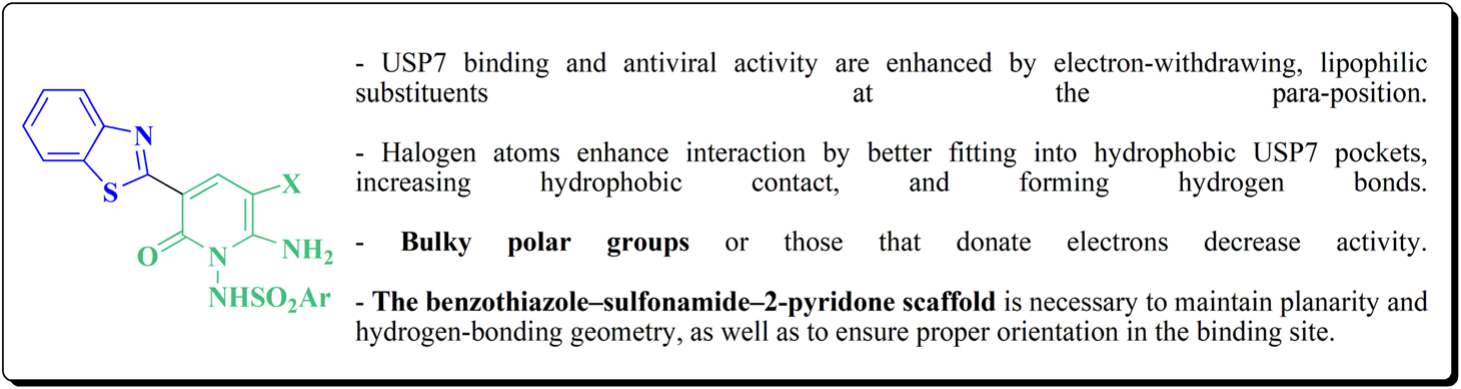
Structure–activity relationship of compound 504.

According to an *in silico* assessment of physicochemical properties, all five candidates had plausible cell-permeability and good oral bioavailability. The most effective HSV 1 inhibitors among them were 502e and 504a, which also showed detectable inhibition of the USP7 enzyme. Both 502e and 504a occupy the USP7 binding pocket and interact strongly with important active-site residues, as further demonstrated by molecular docking (Fig. 86).^268^

Regarding the enzyme inhibition of USP7, the USP7 contributes to the stabilization of viral proteins and the inhibition of p53. Reactivation of host antiviral defenses (*via* p53 reactivation), decreased viral protein stability, and impaired viral DNA replication in infected cells are the results of its inhibition. The compounds form hydrogen bonds and π–π stacking with key residues (*e.g.*, Asp295, Gly277, Tyr465) when they bind to the catalytic domain of USP7. Preventing the deubiquitination process, which causes important proteins that support the virus to be broken down by proteases.^268^

**Scheme 114:**
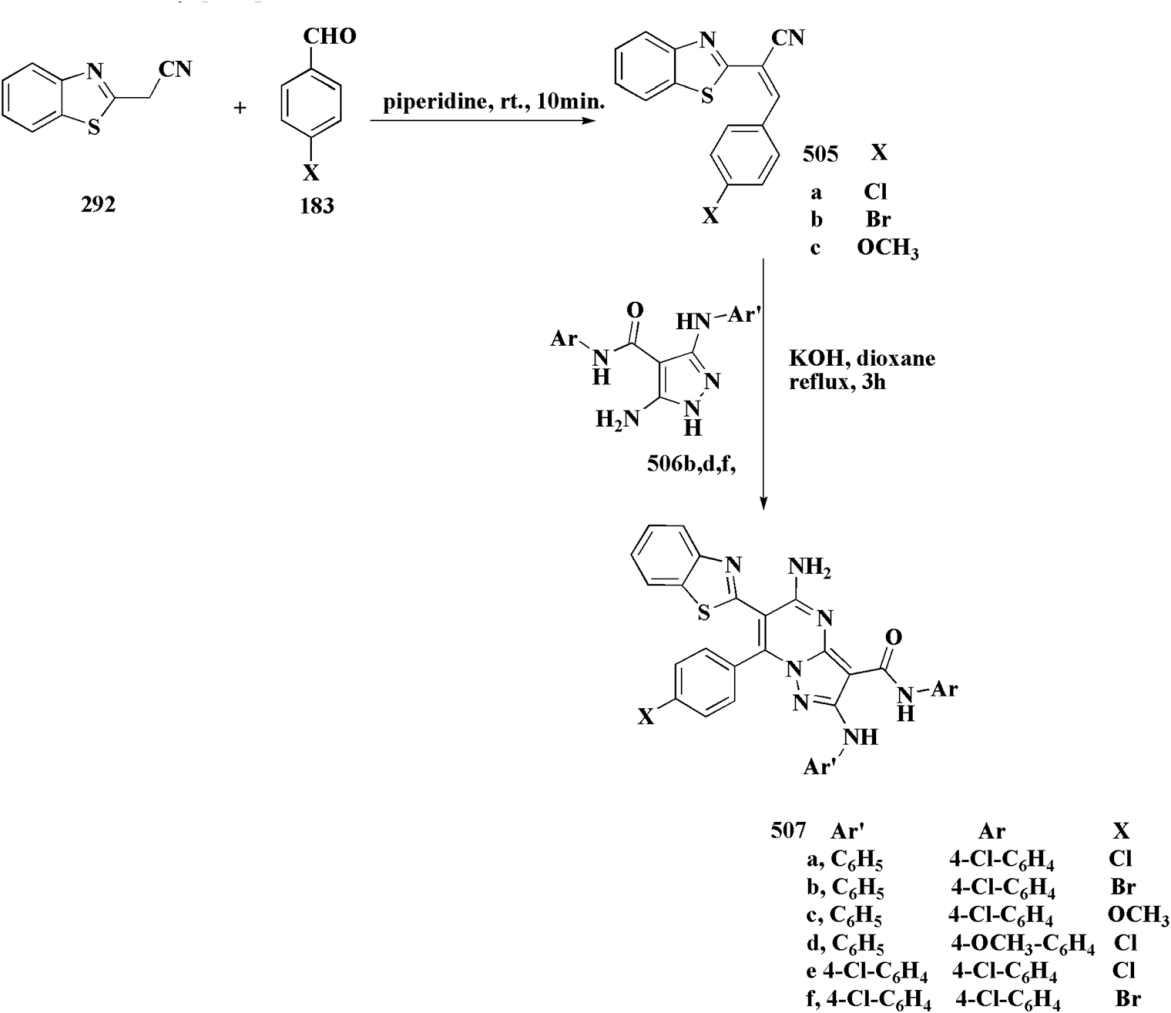
Synthesis of substituted pyrazolopyrimidines.

By reacting pyrazole carboxamide intermediates with different benzothiazole derivatives, new benzothiazolyl–pyrazopyrimidine carboxamide compounds were synthesized (Scheme 114). The antiviral activity of these compounds against the H5N1 virus that causes avian influenza was then assessed. Although compound 507f had the most CDK9 inhibitory activity (0.062 μmol μL^−1^) of all of them, its antiviral efficacy was rather modest, only attaining 40% virus suppression. On the other hand, compound 507b showed the largest antiviral efficacy with 71.6% inhibition, although having the least CDK9 inhibition (0.955 μmol μL^−1^). This suggests that while 507b most likely uses a mechanism that is independent of CDK9 to produce its antiviral effects, 507f may predominantly function as a CDK9 inhibitor with negligible antiviral qualities. With similar CDK9 inhibitory activity (0.143 and 0.144 μmol μL^−1^, respectively), compounds 507a and 507d demonstrated moderate antiviral effects of 71.67% and 61.67%. All of these results point to the possibility that the investigated compounds' antiviral effects are mediated by other routes or mechanisms of action, as there is no discernible relationship between CDK9 inhibition and antiviral activity.^269^

**Scheme 115:**
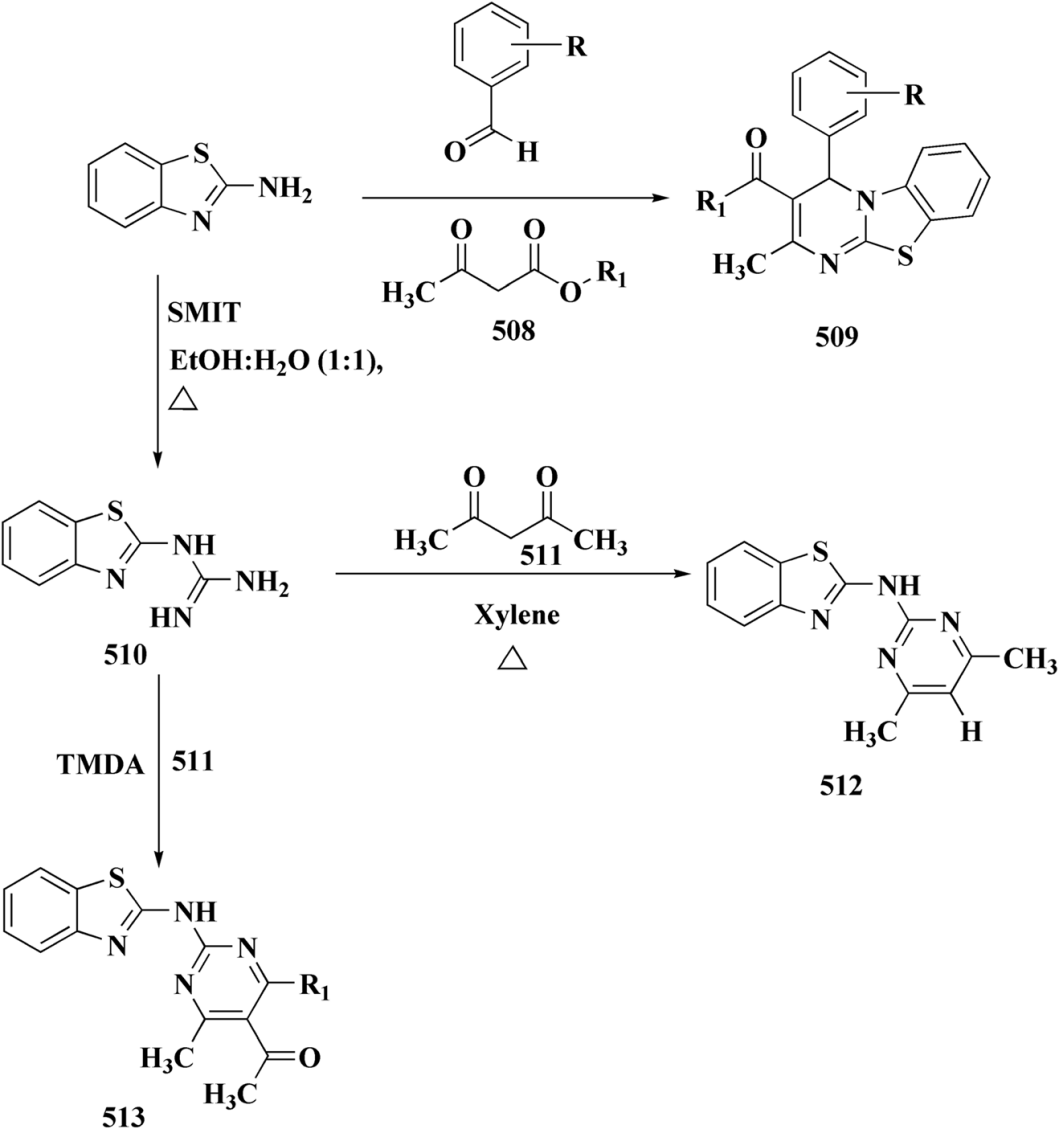
Synthesis of pyrimidobenzothiazole-3-carboxylate derivatives and substituted aminobenzothiazole.

2-Aminobenzothiazole was reacted with acetoacetic acid methyl ester or ethyl 3-oxobutyrate 508 and several substituted arylcarbaldehydes to furnish the benzothiazole-3-carboxylates 509 (Scheme 115).

**Scheme 116:**
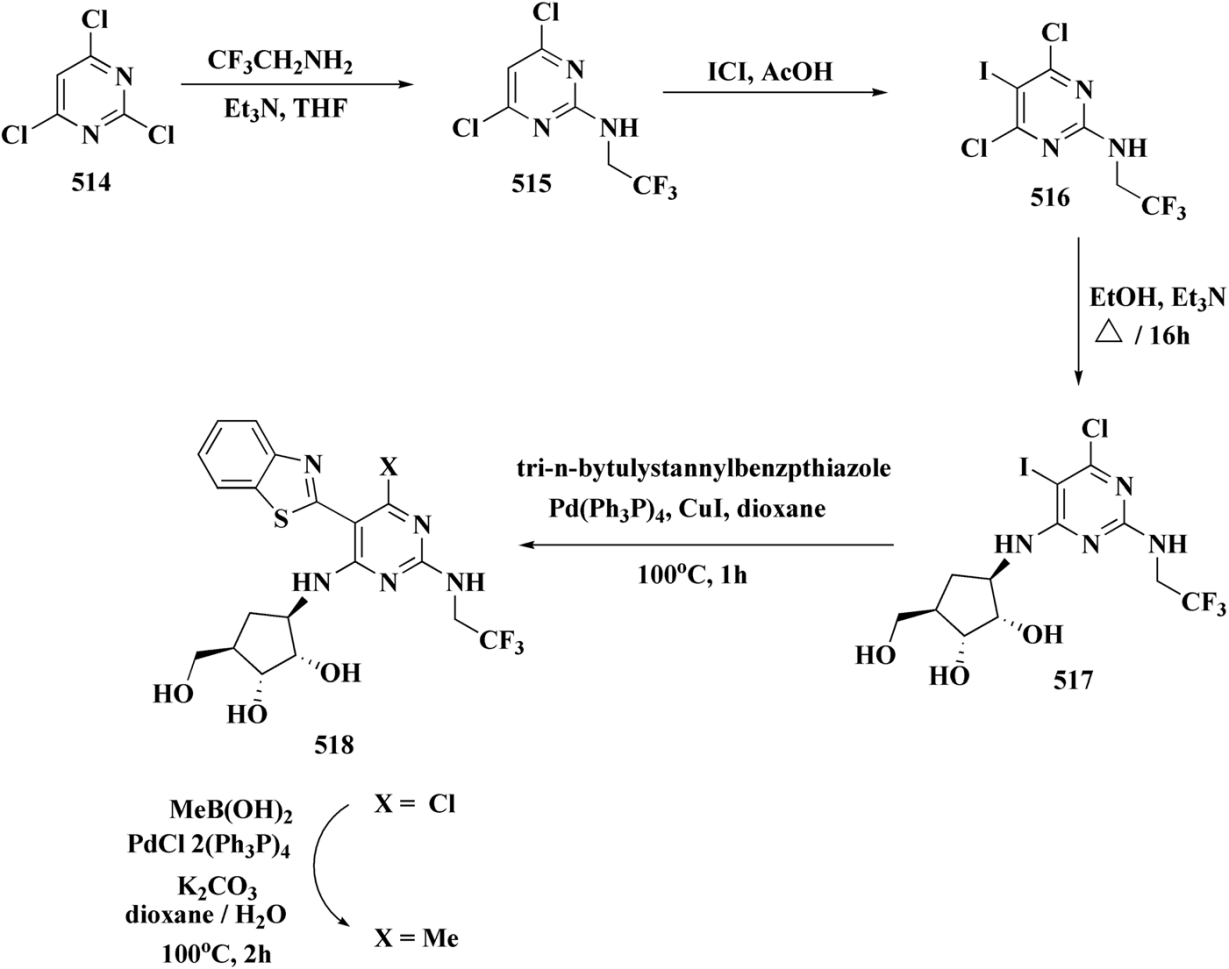
Synthesis of benzothiazole substituted pyrimidines.

Benzothiazolyl guanidine was treated with 511 to provide compound 512. The benzothiazolamine was also furnished from compound 510 as depicted in Scheme 116.

**Fig. 87:**
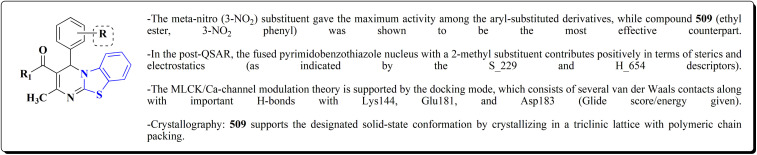
Structure–activity relationship of compound 509.

The benzothiazole-3-carboxylate 509 (R = 3-NO_2_, R^1^ = C_2_H_5_) (Fig. 87) displayed IC_50_ on MLCK assay of 2.1 ± 1.7 lM with selectivity of L-type calcium channels and comparable to nifedipine.^270^

The primary drivers of compound 509's activity are (1) an ethyl carboxylate arm (the ethyl ester at C-3), which increases lipophilicity and provides a favorable PK profile, and (2) a 3-nitro (*meta*-NO_2_) substituent on the phenyl ring, which the authors determined to be the most advantageous aryl substitution. These characteristics, along with the 2-methyl pyrimidobenzothiazole core (positive steric/electrostatic contributions in the 3D-QSAR), allow for selective L-type Ca^2+^-channel modulation and MLCK inhibition (IC_50_ = 2.1 ± 1.7 μM) that is comparable to nifedipine.^270^

**Fig. 88:**
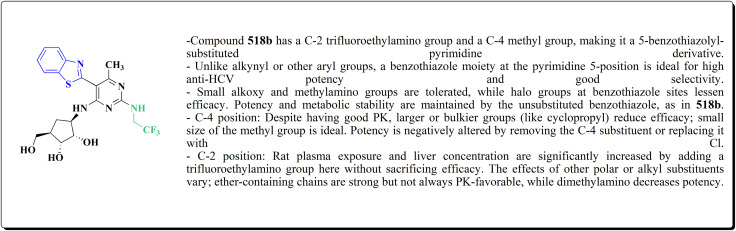
Structure–activity relationship of compound 518.

It was discovered that the best substituent at the pyrimidine 5-position was a benzothiazole moiety. As a result of potential reactivity concern, the 4-chloro residue was replaced by a methyl group with roughly loss in activity and improved rat *in vivo* profile. Wide-ranging estimations at the C-2 position leads to identification of compound 518b (Scheme 116; Fig. 88) that indicated very good replicon activity, rodent plasma/target organ concentration and selectivity. Inhibitor 518b also showed oral bioavailability and good plasma levels in dogs, whereas monkey exposure was relatively low.^271^

The hepatitis C virus (HCV) replication complex, generally known as the replicase, may be inhibited by the carbanucleoside-like pyrimidines, according to previous target engagement studies. RT-PCR was used to detect the suppression of viral RNA replication in HCV genotype 1b subgenomic replicon tests, which showed the antiviral activity. Although no specific enzymatic target was identified in this study, previous research suggests that the replicase complex is the site of action.^271^

**Scheme 117:**
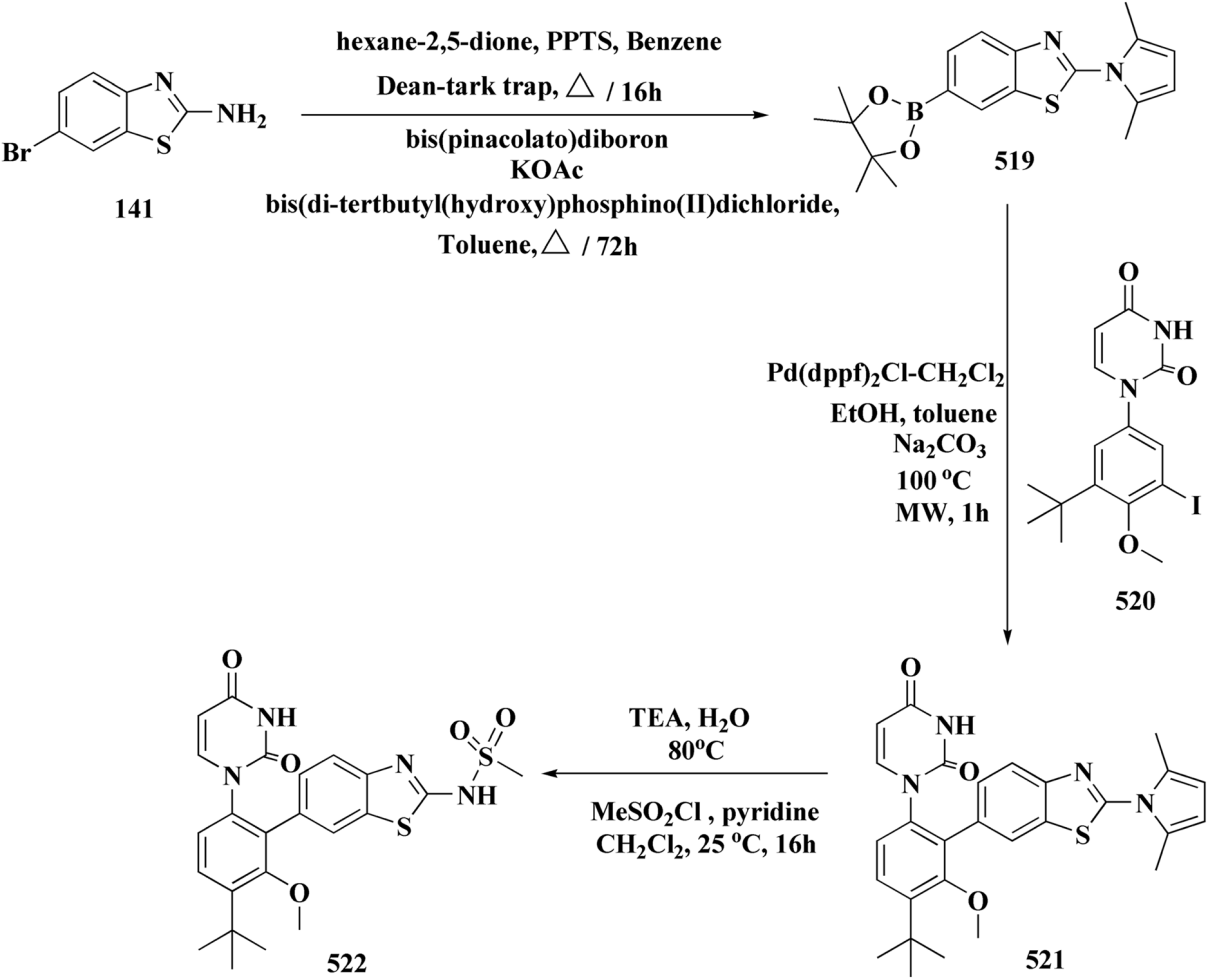
Synthesis of uracil–benzothiazole conjugates.

The sulfonamide inhibitors 522 was synthesized as depicted in Scheme 117.

**Fig. 89:**
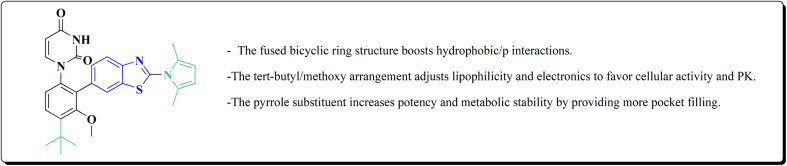
Structure–activity relationship of compound 521.

**Scheme 118:**
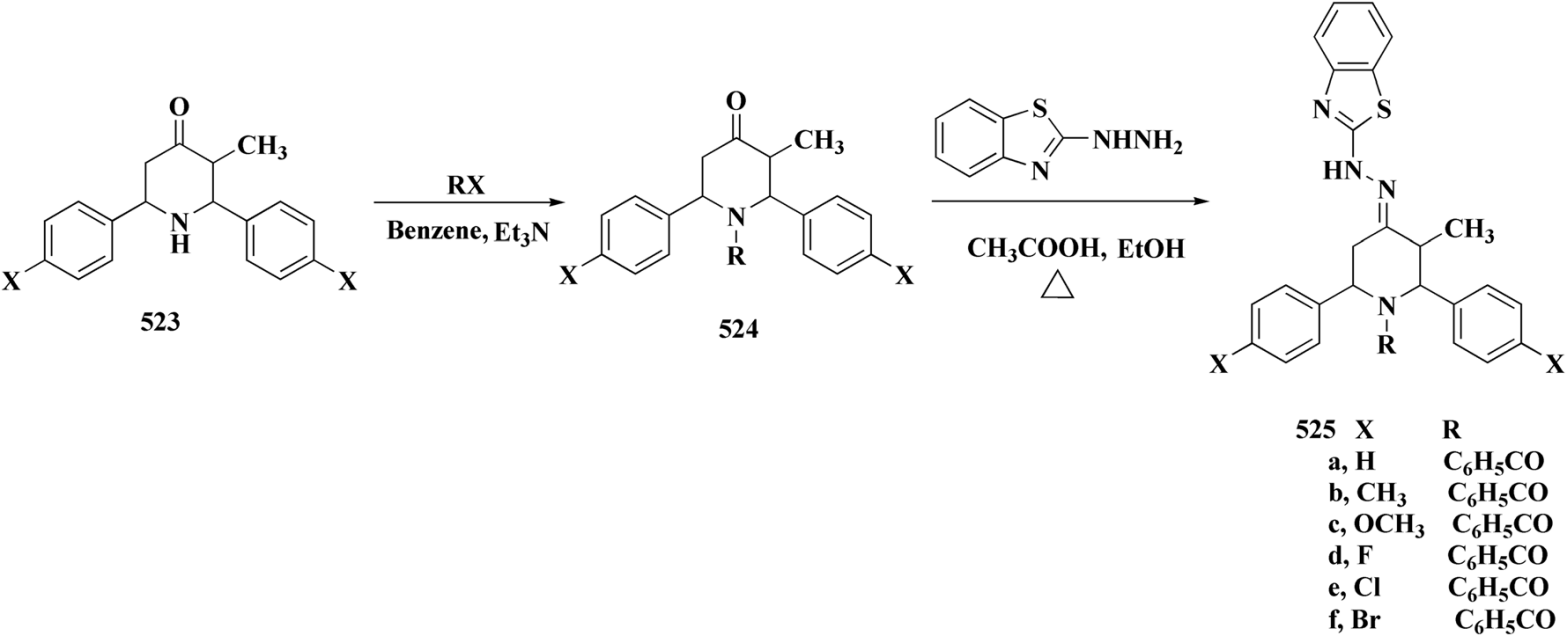
Synthesis of (*E*)-(4-(2-(benzo[*d*]thiazol-2yl)hydrazono)methyl-2,6-diphenylpiperidin-1-yl)(phenyl)methanone.

The synthesis and SAR of aryl uracil series for HCV NS5B inhibition is reported. Various analogs exhibit replicon cell culture activities along with excellent rat pharmacokinetic values (Fig. 89).^272^

(*E*)-(4-(2-(Benzo[*d*]thiazol-2yl)hydrazono)methyl-2,6-diphenylpiperidin-1-yl)(phenyl)methanone [EPHDPM] and its derivatives were prepared (Scheme 118). The reported EPHDPM molecule utilized as an active NLO material since it has high μB_0_ value.^273^

**Scheme 119:**
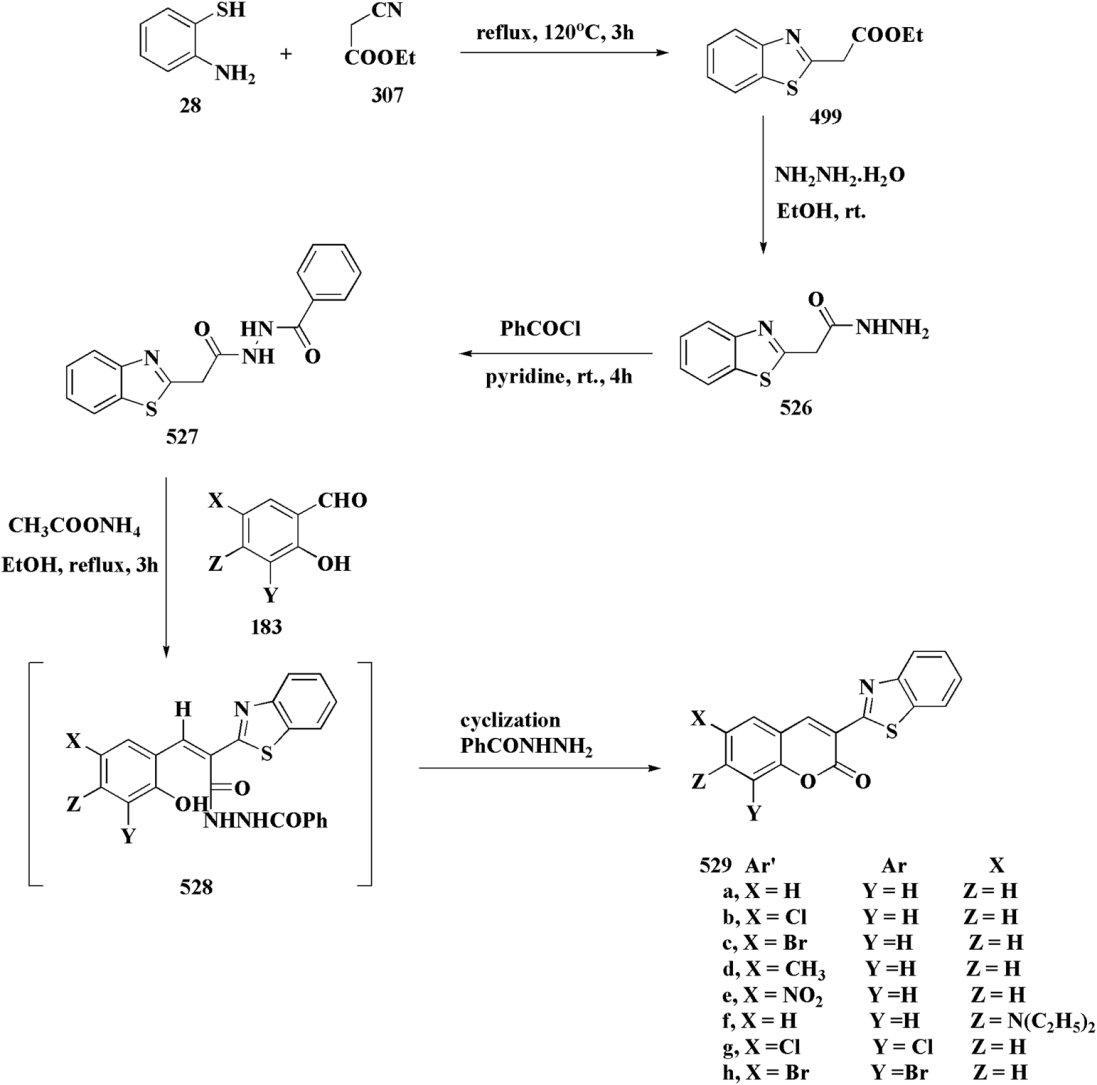
Synthesis of 3-(benzo[*d*]thiazol-2-yl)-2*H*-chromen-2-one derivatives.

**Fig. 90:**
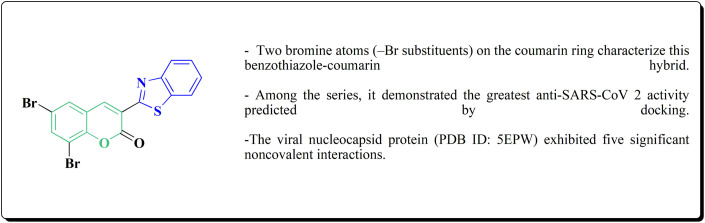
Structure–activity relationship of compound 529.

A set of novel 3-(benzo[*d*]thiazol-2-yl)-2*H*-chromen-2-one derivatives (529a–h) were designed and synthesized (Scheme 119). The emission efficiency gradually decreased as electron-withdrawing substituents were added from the parent molecule 529a to the dibromo derivative 529h, according to fluorescence measurements. Positive binding interactions were predicted for each molecule using molecular docking and virtual screening against the human coronavirus NL63 nucleocapsid protein (PDB ID: 5epw). The most effective anti-COVID-19 candidate among the series was 529h, which formed five important interactions inside the binding pocket. It was determined that the two bromine atoms in 529h were essential to its increased activity (Fig. 90).^274^

**Scheme 120:**
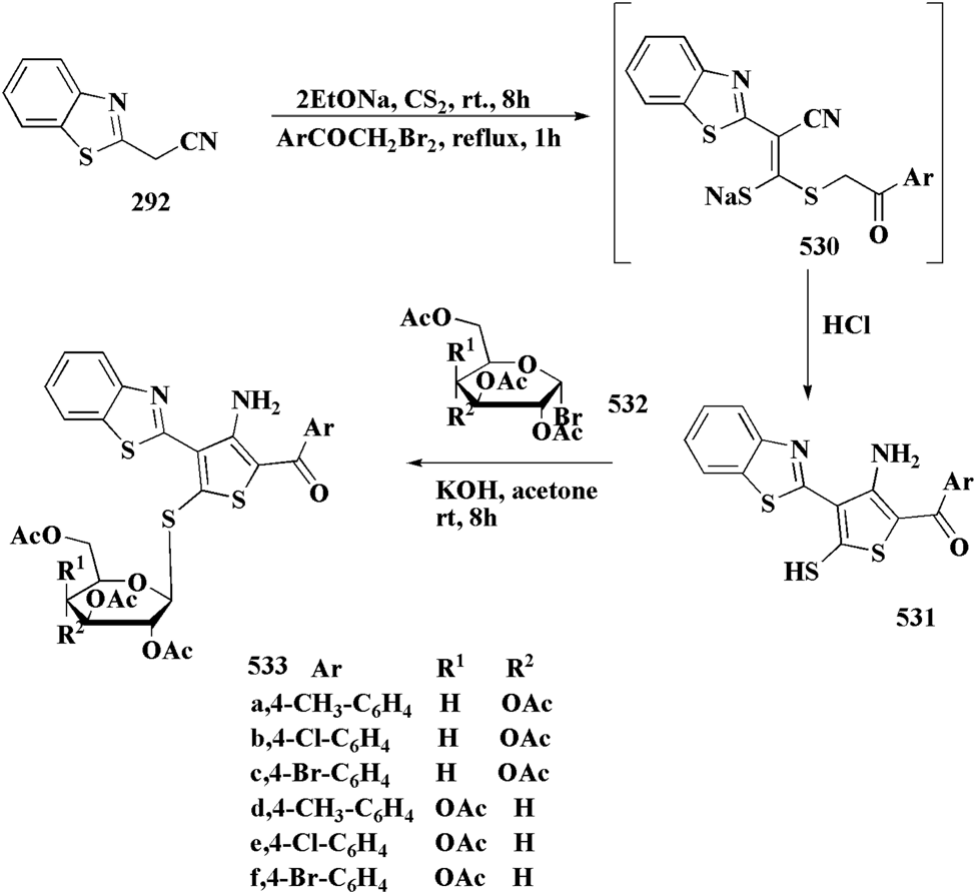
Synthesis of benzothiazole-2-thiophene *S*-glycosides.

By reacting novel 5-mercaptothiophen compounds replaced with the benzothiazole moiety and then coupling with different halo sugar derivatives, new benzothiazole-2-thiophene *S*-glycoside derivatives were synthesized (Scheme 120). The antiviral activity of the novel compounds was evaluated against HSV-1, HAV HM 175, COB4, HAdV7, and the ED-43/SG-Feo (VYG) replicon of HCV genotype 4a. With a viral reduction of over 50%, two compounds showed significant antiviral effectiveness against the CBV4, HSV-1, and HCVcc viruses. The most effective compounds against HCVcc viruses, compounds 533c, was evaluated against the NS3/4A protease and their actions were contrasted with those of the reference medication, sovaldi. The compound was been shown to be the most effective against HSV-1. Further analysis of the produced compounds' anticancer properties revealed that two of them, 533a and 533c, inhibit the majority of cancer kinds, but 533d and 533f only inhibited three and two cell lines, respectively.^275^

**Fig. 91:**
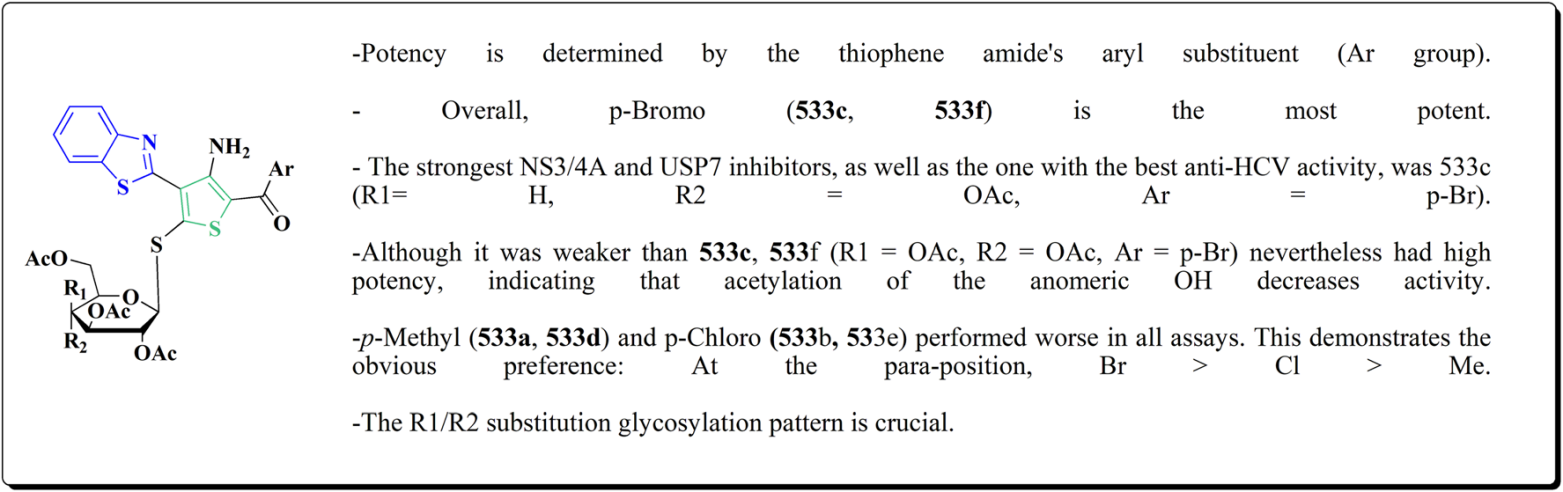
Structure–activity relationship of compound 533.

In comparison to their fully acetylated counterparts (533f) or other substitution patterns, compounds having R_1_ = H and R_2_ = OAc (*e.g.*, 533c) consistently performed better. This trend implies that the equilibrium between solubility and lipophilicity is optimized by a free anomeric OH (R_1_ = H) with per-*O*-acetylation elsewhere, improving bioactivity (Fig. 91).

Comparing series 533 compounds with intact acetates (533a–f) to their deacetylated counterparts in series 9, the former were noticeably more active. This emphasizes how crucial acetyl groups are for both cell permeability and antiviral activity.

In terms of NS3 protease inhibition and anti-HCV activity, 533c stood out. Additionally, it demonstrated a high level of inhibition against USP7, making it the series' dual-active lead. The SAR is extremely substitution-sensitive, as seen by the low activity of other 533-series analogs.^275^

**Scheme 121:**
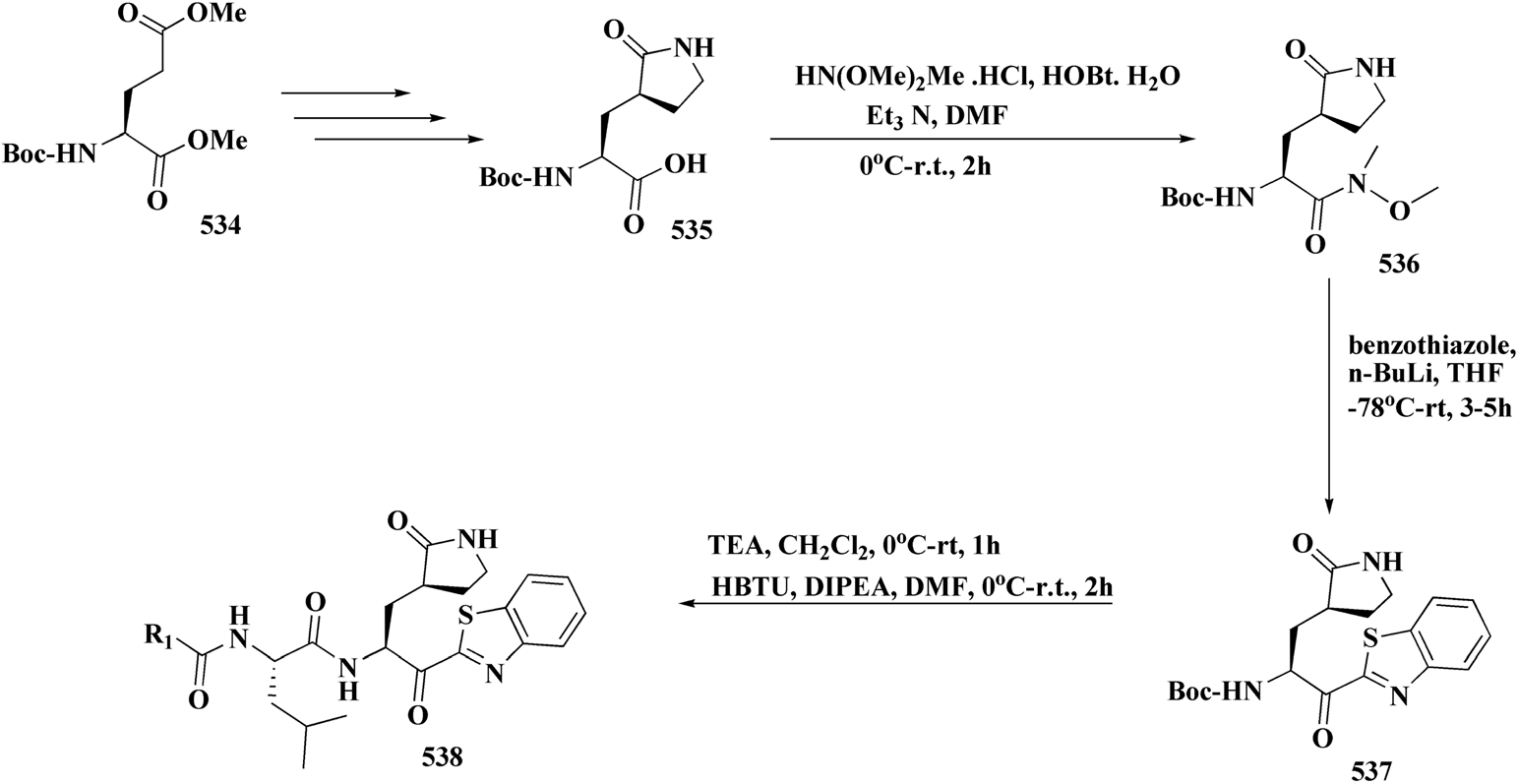
Synthesis of 2-substituted benzothiazole.

The l-glutamic acid ester 534 was transformed to the *g*-lactam-acid 535 through treating with cyanomethyl bromide, followed by reduction with PtO_2_, cyclization, and then hydrolysis (Scheme 121). Subsequent coupling of 535 to *N*-methoxymethylamine utilizing the EDC–HOBt procedure yielded the Weinreb amide 536. The latter compound was then reacted with benzothiazole to give compound 537, which was then followed by deprotection and then coupling to the peptides 534 in the existence of HBTU and DIPEA to yield compounds 538.

**Fig. 92:**
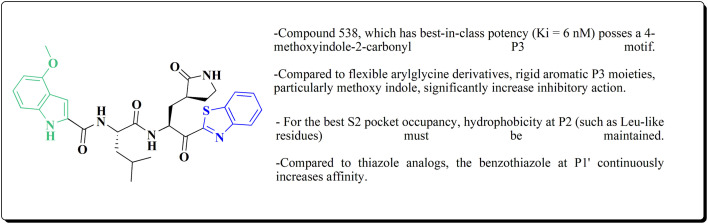
Structure–activity relationship of compound 538.

The synthesis of dipeptide-type inhibitors with new P3 scaffolds that exhibit inhibitory potency against SARS-CoV 3CLpro was reported. Compound 538 exhibited the most active inhibitory potency, with a *K*_i_ value of 0.006 mM (Fig. 92).^276^

By closely resembling the substrate P1′ side chain and slipping into the S1′ pocket, the benzothiazole at the P1′ location increases binding affinity. The S1 pocket's hydrogen bonding is mediated by the γ lactam at P1. The hydrophobic S2 subsite contains the Leu or hydrophobic residue at P2. The stiff indole 2 carbonyl at P3 (in compound 538) helps to achieve the ideal stiffness and binding geometry by forming a stable H bond with Glu166.^276^

**Scheme 122:**
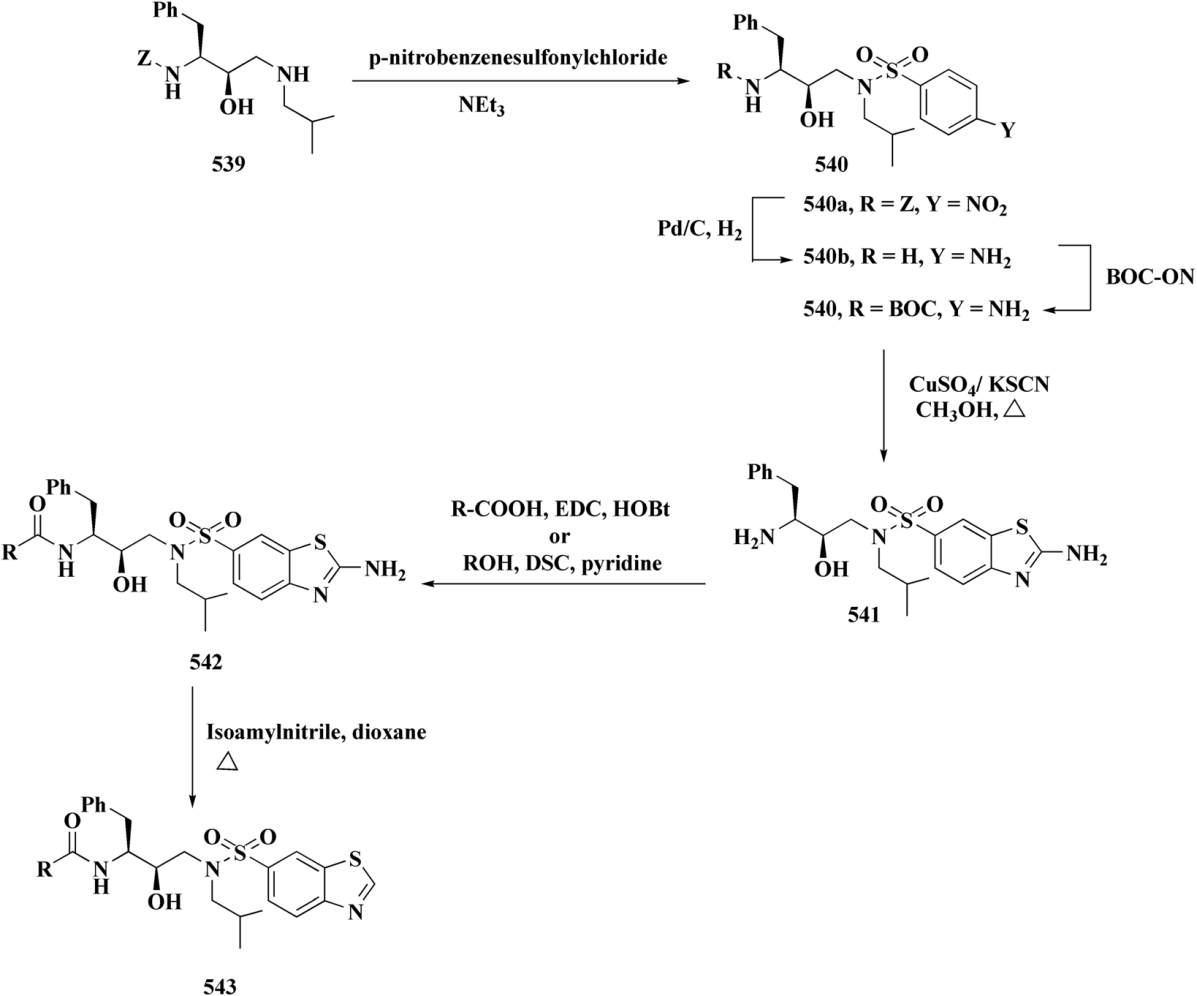
Synthesis of benzothiazole-6-sulfonic acid derivatives.

**Fig. 93:**
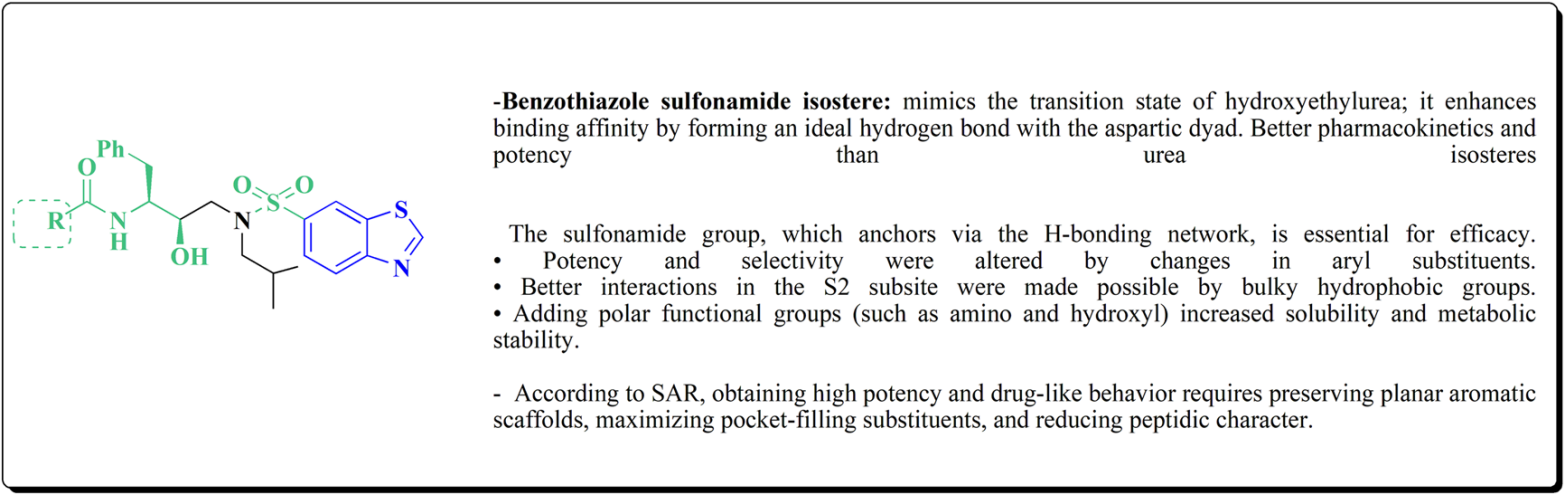
Structure–activity relationship of compound 543.

The preparation of benzothiazole-6-sulfonic acid was developed starting from sulfanilamide as depicted in Scheme 122 (Fig. 93). The replacement of *t*-butylurea scaffold by benzothiazolesulfonamide afforded HIV inhibitors with enhanced activity and antiviral potencies. Some of the compounds have indicated good oral bioavailability and half-life in rats.^277^

In order to successfully simulate the transition-state hydroxyl, the sulfonamide nitrogen and nearby oxygen atoms establish crucial hydrogen interactions with the catalytic Asp25/Asp25′ residues in the protease active site. With flap areas and side chains such as Ile50 and Pro81, the benzothiazole ring improves hydrophobic and aromatic interactions. The whole scaffold effectively prevents the proteolytic cleavage of Gag-Pol precursors by supporting tight, reversible occupancy of the active site. These compounds bind directly to the catalytic aspartyl residues of HIV-1 protease to function as competitive active-site inhibitors.

Hydrogen bonding with the catalytic Asp25/Asp25′ dyad is one of the key interactions. The interactions with S1/S2 subsites that are hydrophobic. Through π–π stacking and dipole interactions, the benzothiazolesulfonamide moiety improves binding affinity and produces a stiff scaffold.

### 10.2. Benzothiazoles fused with heterocyclic compounds

**Scheme 123:**
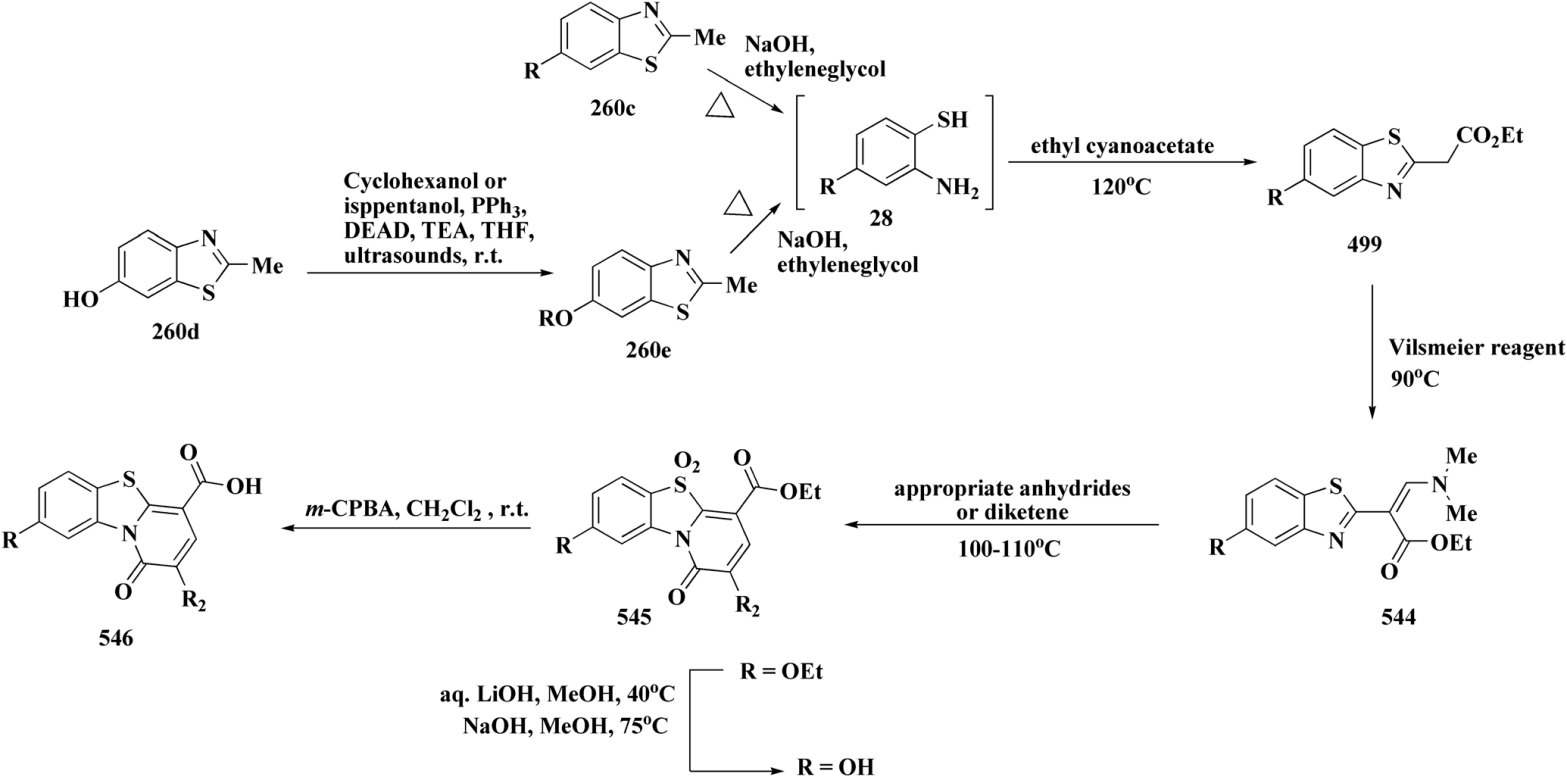
Synthesis of pyridobenzothiazole-4-carboxylate derivatives.

**Fig. 94:**
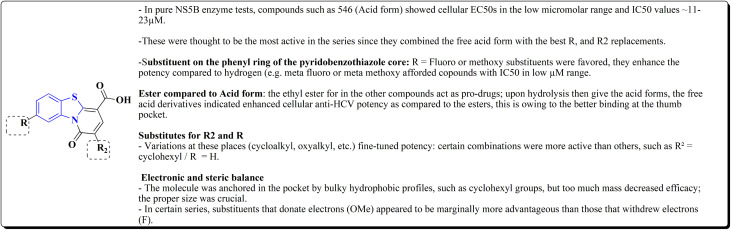
Structure–activity relationship of compound.

The synthesis and in cellulo anti-HCV & *in vitro* anti-NS5B evaluation of pyridobenzothiazole-4-carboxylate derivatives are reported (Scheme 123; Fig. 94).^278^

These compounds attach to the HCV NS5B polymerase thumb domain allosteric location. The four carboxylate derivatives (as ester, amide, or sulfonamide) point toward solvent-exposed regions, affecting solubility and binding orientation; the pyridobenzothiazole core offers a rigid, planar scaffold that fits into the hydrophobic thumb pocket; potential π–π and hydrophobic interactions with residues in the thumb region.

The NS5B polymerase is inhibited by these non-nucleoside allosteric pyrido[2,1-*b*][1,3]benzothiazole 4 carboxylate derivatives. They don't bind at the active site or mimic nucleotides. Rather, they attach to an allosteric site in the thumb domain, changing the structure of the enzyme and preventing it from functioning.

According to molecular modeling and pharmacological research, the substances bind to the thumb domain and disrupt the conformational dynamics necessary for RNA elongation.^278^

## 11. Conclusion

Synthetic approaches of novel targeting therapeutical benzothiazoles were provided. Various benzothiazole-based compounds have emerged as significant structures, they are viable bioactive agents that are interested in advancing the research of the therapeutic arena. Several benzothiazole-based drugs were accomplished. Up-to-date synthetic strategies of anti-neurodegenerative, anti-inflammatory, antitumor, anti-microbial, and anti-viral benzothiazoles are depicted. The biological evaluation of the new synthesized compounds is also emphasized. The pharmacological potency and selectivity of benzothiazoles have been further improved by structural alterations and hybridization with other pharmacophores. Numerous medications, both licensed and under research, as well as novel molecules, have shown encouraging preclinical and clinical results. To effectively transform these heterocycles into medicinal medicines, more research is needed to understand their structure–activity correlations (SAR), molecular processes, and *in vivo* efficacy. The synthesis of safer and more effective benzothiazole-based medications will be further accelerated by the combination of computational tools, tailored delivery methods, and green synthesis methodologies.

## Conflicts of interest

There are no conflicts to declare.

## Data Availability

No primary research results, software or code have been included and no new data were generated or analysed as part of this review.
